# Assessing spatial transmission risk of respiratory infectious diseases across cities of different socioeconomic tiers in China: A modelling study

**DOI:** 10.1371/journal.pmed.1005172

**Published:** 2026-07-20

**Authors:** Wenjie Li, Wei Yang, Yang Liu, Ye Yao

**Affiliations:** 1 Research Institute of Intelligent Complex Systems, Fudan University, Shanghai, China; 2 Department of Biostatistics, School of Public Health, Fudan University, Shanghai, China; 3 PATH, London, United Kingdom; 4 Centre for Mathematical Modelling of Infectious Diseases, London School of Hygiene & Tropical Medicine, London, United Kingdom; 5 Department of Infectious Disease Epidemiology, Faculty of Epidemiology and Population Health, London School of Hygiene & Tropical Medicine, London, United Kingdom; 6 Shanghai Institute of Infectious Disease and Biosecurity, Fudan University, Shanghai, China; 7 Key Laboratory of Public Health Safety of Ministry of Education, School of Public Health, Fudan University, Shanghai, China; Institut Pasteur, FRANCE

## Abstract

**Background:**

Understanding how respiratory infectious diseases spread across cities of different socioeconomic tiers is crucial for regionally targeted interventions. However, most spatial prediction frameworks neglect the combined influence of urban hierarchy and human mobility in shaping transmission risk.

**Methods and findings:**

We integrated large-scale intercity mobility data into an agent-based branching process model to simulate the spatial diffusion of respiratory pathogens across mainland China. Three COVID-19 outbreaks were used for validation: the Omicron outbreak in Shanghai, the Delta outbreak in Nanjing, and a multi-provincial Delta outbreak in northwestern China. We applied the framework to model the spread of SARS-CoV-2 (Omicron variant) and Influenza A to quantify tier-specific transmission risks. Tiers denote a hierarchical classification of Chinese cities based on concentration of commercial resources, transportation hub centrality, etc., ranging from super-tier metropolises to lower-tier cities. Predicted first arrival times showed strong agreement with observed data (*r* = 0.68 and 0.76), and mobility-based predictions more accurately identified outbreak origins than distance-based approaches. Markedly different tier-dependent diffusion patterns were observed across pathogens. Influenza A exhibited stable and stratified diffusion, with transmission confined mainly within the same or adjacent urban tiers and limited cross-tier seeding. In contrast, SARS-CoV-2 (Omicron) initially concentrated in super-tier and tier-1 cities but rapidly spread to lower-tier cities, producing a pronounced hierarchical pattern of spread that quickly diminished tier-level differences in transmission risk. Across pathogens, higher-tier cities consistently faced greater early importation risk; however, this disparity persisted for Influenza A but was rapidly attenuated for Omicron due to its high transmissibility and fast spatial expansion. A key limitation is that the model was validated against city-level first arrival times rather than full epidemic dynamics, and was parameterised using mobility data from China’s “dynamic zero-COVID” period, which may limit direct quantitative generalisability to other settings.

**Conclusions:**

Spatial transmission risk reflects an interaction between pathogen-specific transmissibility and the hierarchical organisation of China’s urban mobility system. These findings indicate that surveillance and response strategies effective for less transmissible pathogens may be insufficient for highly transmissible variants. Mobility-informed, tier-specific risk assessment may help inform early warning and support more adaptive public health responses. Because the framework relies on routinely available mobility data and minimal pathogen-specific inputs, it may provide a scalable approach for epidemic preparedness, including potential future emerging respiratory threats (Disease X).

## Author summary

**Why was this study done?:**

- Human mobility plays a key role in how infectious diseases spread between cities, but the distribution of early transmission risk within a country remains incompletely understood.
- Most existing studies rely on indirect or aggregated data, making it difficult to directly assess how well models capture the early spatial spread of infections across cities.
- While respiratory pathogens differ in their transmission characteristics, there remains limited empirical evidence on how these differences translate into distinct spatial spread patterns within the same mobility system.

**What did the researchers do and find?:**

- We developed a model that combines human mobility data with disease transmission to simulate how infections spread between cities.
- Using detailed data from COVID-19 outbreaks in China, we validated the model against observed spread patterns and showed that it can reproduce the timing of infection first appearing in different cities.
- We found that transmission risk is structured by city hierarchy and that different pathogens (e.g., COVID-19 variants versus influenza) spread across the same mobility network in markedly different ways.

**What do these findings mean?:**

- Early transmission risk is not evenly distributed: some cities play a disproportionately important role in driving spread.
- Public health responses may need to be tailored to both city characteristics and pathogen type, rather than to a uniform strategy.
- These results are based on data from a specific setting with unusually detailed surveillance and may not fully generalise to all contexts.

## Abstract 3

In a modelling study, Wenjie Li and colleagues investigate how respiratory infectious diseases spread across cities of different socioeconomic tiers by simulating the spatial diffusion of respiratory pathogens across mainland China.

## Introduction

Pandemics of respiratory infectious diseases continue to challenge global health security in an increasingly interconnected world [1–3]. Effective prevention and control hinge on the ability to anticipate when and where infections will spread, so that interventions can be deployed with precision rather than uniformly across regions. Such targeted prevention and control, the spatial counterpart of risk-based population strategies, is essential for minimising unwarranted disruptions while containing outbreaks [4,5]. The success of spatially targeted interventions relies critically on the ability to generate accurate early predictions of disease diffusion across urban networks. However, accurately anticipating the spatial trajectory of spread remains challenging in real-world settings [6,7].

Traditional approaches to modelling intercity spread have often relied on geographical distance as a proxy for connectivity, assuming that shorter distances correspond to higher transmission risk [8,9]. Nevertheless, in the context of modern transportation systems and dense travel networks, geographical closeness no longer adequately represents the actual flow of people and pathogens [10]. Empirical evidence increasingly shows that mobility-based connectivity, derived from real population movements, better explains observed diffusion patterns than static distance measures [11–13]. The use of large-scale mobility data thus provides a data-driven foundation for improving the accuracy of spatiotemporal predictions, particularly during the early stages of emerging outbreaks when rapid assessment is critical for decision-making.

At the same time, effective spatial targeting of interventions also requires an understanding of how disease diffusion patterns differ across pathogens. Fast-spreading viruses such as SARS-CoV-2 may rapidly traverse intercity mobility networks, necessitating early interregional coordination and broad geographical alert zones [14–16]. In contrast, pathogens with lower transmissibility, such as seasonal influenza, may exhibit more stratified or locally contained spatial trajectories [17,18], enabling more focussed and efficient control measures. In countries with substantial socioeconomic heterogeneity and hierarchical urban systems, as characterised, for example, by Brazil’s IBGE Regiões de Influência das Cidades [19], Indonesia’s BPS Human Development Index-based classification [20], India’s Ministry of Finance tier classification [21], and the United States’ Brookings Metro Monitor rankings [22], integrating mobility-informed risk assessment into epidemic preparedness frameworks provides a practical pathway toward achieving precision and efficiency in outbreak response.

A central limitation in validating mobility-driven models of respiratory disease spread is the difficulty of directly observing intercity infector-infectee relationships, such that validation typically relies on statistically inferred transmission patterns. Here, we leverage a uniquely detailed dataset from three COVID-19 outbreaks in China to enable validation against directly observed transmission events. More broadly, this study makes three contributions. First, it provides high-fidelity empirical validation of mobility-driven diffusion models using directly observed transmission events, strengthening the empirical grounding of such approaches. Second, it quantifies how early transmission risk is structured across urban hierarchies within a country, generating sub-national, tier-stratified evidence to inform regionally differentiated public health strategies. Third, it explicitly incorporates a direct empirical comparison of how a shared mobility network shapes spatial diffusion across respiratory pathogens with contrasting transmission characteristics, thereby informing an important and previously unresolved policy question of whether internal movement restrictions and other regionally targeted measures are likely to exert comparable effects across different respiratory pathogens.

To address these challenges, this study introduces a mobility-informed analytical framework to understand and predict the spatial diffusion of respiratory infectious diseases. By integrating large-scale intercity movement data into an agent-based branching process framework, we aim to capture how real human mobility shapes epidemic trajectories. Using SARS-CoV-2 and Influenza A as representative pathogens, we further assess the spatial heterogeneity of transmission risks across urban tiers, providing insights for early warning and targeted intervention. This framework lays the foundation for scalable, data-driven approaches to epidemic preparedness and response. Specifically, we asked whether intercity human mobility alone can reproduce and predict the early city-level spatial spread of respiratory pathogens in China, and whether pathogens with contrasting transmissibility (SARS-CoV-2 Omicron versus Influenza A) generate systematically different tier-stratified transmission risks across the same mobility network.

## Methods

### Mobility data

Baidu is one of China’s largest technology companies. Baidu’s Baidu Maps application provides location-based services to over 1.1 billion mobile device users in mainland China. After anonymisation and aggregation, these data are converted into a daily city-level migration index, which is publicly available on Baidu’s platform, Baidu "Qianxi" (which means "migration" in Chinese) [23]. We extracted the migration index for 366 cities in mainland China between 1 January 2021 and 5 April 2022. For each city and day, we obtained both the outbound migration scale index and the proportion of outflow directed to each destination city. We combined these two components to estimate city-to-city mobility flows and then normalised these flows to derive travel probabilities between origin-destination pairs (Method C in S1 Appendix). These probabilities were assembled into city-to-city mobility transition matrices, which were used to parameterise intercity movement in the transmission model. We constrained our analysis to this period to capture population mobility patterns during the routine response phase of the ‘dynamic zero-COVID’ strategy, which represents a scenario of heightened alertness but relatively stable intercity travel compared to city-wide lockdowns.

### Epidemiological data

Data on daily new infections during three COVID-19 outbreaks in 2021 and 2022 in mainland China were collected from the official press releases of the National Health Commission of China. In the context of this study, we define the start of an outbreak as the detection of the first case, and the end of an outbreak as the last confirmed case or the time of substantial public health and social measures (PHSM) introduction. Using these definitions, the three outbreaks lasted from 1 March to 5 April 2022, 20 July to 5 August 2021, and 17 October to 3 November 2021 (Methods A and B in S1 Appendix, Tables E–G in S1 Appendix). The first cases of these outbreaks were detected in Shanghai, Nanjing, and northwestern China, respectively. These three outbreaks involved 121, 28, and 26 cities overall.

### Agent-based branching process model

**Fig 1:**
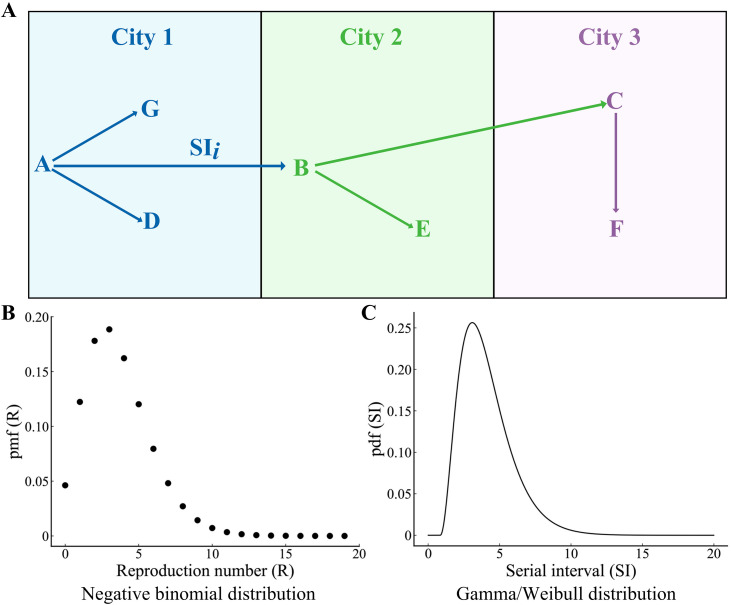
Example of the agent-based branching process model. **(A)** A conceptual diagram of the branching process model; **(B)** the probability mass function (pmf) capturing the uncertainty around the reproduction number; **(C)** the probability density function (pdf) capturing the uncertainty around the serial interval. In **(A)**, person A infected three individuals, B, D, and G. This level of transmissibility, in implementation, is drawn from the probability distribution in **(B)**. Individuals D and G do not travel and, therefore, would be reported in city 1 as new infections. Individual B, however, travelled to city 2 because on one of the days before symptom onset, they were sampled to travel based on the migration index from city 1 to city 2. The total number of days before symptom onset will be randomly sampled from the probability distribution presented in **(C)**. In this example, the first arrival time of City 2 corresponded to the serial interval of individual B, as B represented the first introduction into City 2. Similarly, the first arrival time of City 3 was determined by the sum of the serial intervals of individuals B and C, since the infection chain A → B → C constituted the earliest seeding pathway to City 3.

We developed an agent-based branching process model on the city level to capture the spatial propagation of COVID-19 outbreaks (Fig 1). In the agent-based branching process model, the number of secondary infections generated by an infected individual followed a negative binomial distribution with a mean equal to the reproduction number (*R*_0_), capturing individual heterogeneity in transmissibility. The infection time for each potential secondary case was drawn from the serial interval (SI) distribution (Method E in S1 Appendix), Gamma for SARS-CoV-2 and Weibull for seasonal influenza A (Table 1). Human mobility was incorporated through a sigmoid function describing the probability of travel as a function of the local daily migration index (Method D in S1 Appendix). When travel occurred, the destination city was selected based on the observed proportions of outbound travellers, as determined from Baidu Migration Index data (Method C in S1 Appendix). An example of this process is illustrated in Fig 1: an infected individual A in City 1 generated three secondary infections (B, D, and G); B then travelled to City 2 and infected two additional cases (C and E); and C travelled to City 3 and produced one further infection (F). This example shows how infection events and travel behaviour jointly shape the spatial spread of infection.

**Table 1:** Parameter values for the simulation model.

Pathogen/parameter	Value used	References
**SARS-CoV-2 (Omicron BA.2)**		
$R_{e}$ (Effective reproduction number)	Negative binomial distribution mean = 3.40, overdispersion (k = 0.33)	Cai and colleagues [24]; Guo and colleagues [25]
Serial interval	Gamma distribution mean = 2.72 days, shape = 3.25, scale = 0.84	Mefsin and colleagues [26]
**SARS-CoV-2 (Delta)**		
$R_{e}$ (Effective reproduction number)	Negative binomial distribution mean = 3.20, overdispersion (k = 0.85)	Zhang and colleagues [27]; Ryu and colleagues [28]
Serial interval	Gamma distribution mean = 3.59 days, shape = 2.06, scale = 1.74	Xu and colleagues [29]
**Seasonal Influenza A**		
$R_{\text{0}}$ (Basic reproduction number)	Negative binomial distribution mean = 1.50, overdispersion (k = 8.09)	Petersen and colleagues [30]; Hébert-Dufresne and colleagues [31]
Serial interval	Weibull distribution mean = 3.60 days, α = 4.10, β = 2.30	Cowling and colleagues [32]

Parameter values for SARS-CoV-2 (Omicron variant) and seasonal Influenza A were used in the agent-based branching process simulations. For Omicron, the effective reproduction number ($R_{e}$) was drawn from a negative binomial distribution (mean = 3.40, overdispersion k = 0.33), and the serial interval from a gamma distribution (mean = 2.72 days, shape = 3.25, scale = 0.84). For Influenza A, the basic reproduction number ($R_{\text{0}}$) followed a negative binomial distribution (mean = 1.50, overdispersion k = 8.09), and the serial interval followed a Weibull distribution (mean = 3.60 days, *α* = 4.10, *β* = 2.30). Parameter sources are shown in the References column.

For clarity, the main steps of the spatial agent-based branching process are summarised in the following pseudocode.

**Algorithm: Spatial agent-based branching process simulation**

**Input**: seed city o, initial infections N_0_, time window W, offspring distribution, serial interval distribution, mobility matrix P

**Output**: first arrival time FA(c) for each city c

Initialise N_0_ infections in city o on day 1. Set FA(o) = 1 and FA(c) = undefined for all other cities. Add initial infections to queue Q

**while** Q is not empty **do**

 Remove one infected individual i from Q

 Draw the number of secondary infections r

 **for** each secondary infection **do**

  Draw serial interval Δ

  Set t = day(i) + round(Δ)

  Assign destination city c according to P

  **if** FA(c) is undefined **then** FA(c) = t

  **if** t ≤ W **then** add offspring (t, c) to Q

 **end for**

**end while**

**Return** FA(c)

Repeat over stochastic simulations and summarise city-specific first arrival times

### Simulated first arrival time

To quantify the spatiotemporal dynamics of disease spread, we simulated the first arrival time in each city using the agent-based branching process model described above. The first arrival time was defined as the elapsed time between the onset of the outbreak in the source city and the first infection in the destination city. During each simulation, all infection and travel events were recorded. When an infected individual travelled from their original city to another city, the infection time of that individual determined the first arrival time of the destination city. If multiple infected individuals arrived in the same city, the earliest infection time among them was defined as that city’s first arrival time. A simple example was illustrated in Fig 1. To ensure that the simulated first arrival times reflect spatial diffusion driven purely by population mobility rather than disease-specific transmission parameters, this component of the simulation omitted pathogen-specific values of reproduction number (*R*_0_) and SI, allowing infections to propagate solely through mobility processes. To provide a more comprehensive validation framework, we additionally conducted an extended validation analysis that incorporated outbreak-specific reproduction numbers and serial intervals, with the results presented in Figs A and B in S1 Appendix. Each outbreak-origin scenario was simulated 10,000 times to account for stochastic variation in transmission and travel processes (Method F in S1 Appendix). For each simulation, we recorded the first arrival time for every city that was seeded within the simulation horizon. Cities that were not seeded during the simulation period were classified as not reached. For each city, the predicted first arrival time used in the main analysis was summarised as the mean across simulations, which provides a stable estimate of expected arrival timing under stochastic variability. Model performance was evaluated by comparing the observed first arrival times with the mean predicted first arrival times using Pearson correlation, mean absolute error (MAE), and root mean square error (RMSE).

First arrival time was chosen as the primary validation target because it matches the policy frame our evidence is intended to inform: in China, responses to respiratory epidemics are typically enacted at the city level and initiated by a local trigger, so the operationally meaningful question for city-level practitioners is whether an outbreak has arrived—not what proportion of the country is affected or how fast it is spreading spatially. First arrival timing also more directly reflects intercity diffusion on the mobility network and is less confounded by city-specific differences in prior infection, vaccination, local interventions, and climatic conditions than metrics based on peak incidence, cumulative incidence, epidemic intensity, or final attack rate.

### Model parameters and scenarios

We first used observed COVID-19 outbreaks to validate the model’s ability to reproduce real-world spatial diffusion patterns. We then applied the validated framework to Influenza A as a hypothetical scenario to illustrate its use in comparing spatial transmission risk across urban tiers under a different respiratory infection context. We conducted a risk assessment by simulating the agent-based branching process model to explore two scenarios involving two pathogens: SARS-CoV-2 (Omicron) and Influenza A. The parameters we used for these pathogens are presented in Table 1. We assumed there were 100 cases introduced initially and ran the simulation for 21 days. We defined reproduction numbers according to their epidemiological interpretation:$R_{\text{0}}$ denotes the basic reproduction number in a fully susceptible population without interventions, whereas $R_{e}$ denotes the effective reproduction number under prevailing epidemiological and control conditions. For the COVID-19 Omicron validation analyses, we therefore referred to *R*_e_. For the hypothetical Influenza A simulations, the reproduction parameter represents an assumed baseline transmissibility parameter analogous to $R_{\text{0}}$ under the modelled conditions.

For the baseline analyses, serial interval distributions were parameterised using literature-based point estimates. In each simulation, however, the serial interval for each transmission event was randomly drawn from the specified distribution. Uncertainty in the serial interval distribution parameters themselves was examined separately in sensitivity analyses, including the joint uncertainty analysis, in which alternative parameter combinations were sampled over plausible ranges.

### Identifying the origin

We initiated outbreaks in each of the 366 prefectures under consideration at the beginning of each outbreak, thereby obtaining 366 sets of 366 predicted arrival times. We compared the simulated first arrival time pattern generated by each candidate origin with the observed first arrival time pattern across affected cities. This comparison was performed using the Pearson correlation between the logarithm of the predicted first arrival time ($\text{lg}T_{pre}$) and the logarithm of the observed first arrival time ($\text{lg}T_{obs}$). We used logarithmically transformed first arrival times for the mobility-based analyses because the relationship between the diffusion time (t) and diffusion distance (D) follows a power-law form $t\;\sim \;D^{\alpha }$. In the origin-identification analysis, the Pearson correlation on log-transformed arrival times was used primarily to compare the similarity between the observed and predicted arrival time patterns across cities and to preserve information on their relative temporal spacing (Method I in S1 Appendix). The prefecture with the highest Pearson correlation coefficient for each outbreak was identified as its origin. The origins of the Shanghai and Nanjing outbreaks are known through outbreak investigation efforts; therefore, these outbreaks are used to validate this approach (Method K in S1 Appendix).

We compared this approach to using geodesic distance to identify outbreak origins. The geodesic distance between a pair of cities was obtained using the Haversine formula, calculated from the latitude and longitude of the two cities. In the context of SARS-CoV-2 transmission, we do not know the functional relationship between diffusion distance and time. Thus, we used the Spearman correlation between geodesic distance and raw observed arrival times to approximate the association between predicted and observed arrival times (Method J in S1 Appendix).

### Risk potential assessment

Using the validated agent-based branching process model, we simulated the propagation of two pathogens in mainland China, using their $R_{\text{0}}$ and serial intervals. We assumed 100 initial cases at the origin and simulated the outbreak for 7, 14, and 21 days. We introduce $N_{i}^{\text{risk}}$ to quantify the potential scope of an outbreak, which denotes the number of people at risk. The entire population of a city is considered at risk once the pathogen has been transmitted to that city. Therefore, we have the following relationship:


$$\begin{array}{c} N_{i}^{\text{risk}}=\sum_{j}{\text{Pop}}_{j}a_{ij} \end{array}$$ (1)

where ${\text{Pop}}_{j}$ is the number of residents of city j; $a_{ij}$ is a binary variable indicating if city *j* has received infectious individuals from city i. Alternative definitions of $N_{i}^{\text{risk}}$ based on importation thresholds are detailed in Method O in S1 Appendix.

For each origin city, the risk metric $N_{i}^{\text{risk}}$ was estimated as the mean across 10,000 stochastic simulations. Destination cities that were not seeded within the simulation horizon in a given simulation were not counted as contributing to transmission risk in that replicate. We assumed 100 initial cases as a standardised seeding condition for the risk simulations to enable stable and comparable estimates of relative transmission risk across cities; sensitivity analyses using alternative initial case numbers showed similar overall patterns.

### Prefecture-level population and tier classification

A prefecture is a second-level administrative unit in mainland China, below the province (i.e., the first-level administrative unit) and county levels (i.e., the third-level administrative unit). Prefecture-level population data were collected from the Seventh National Population Census of China in 2021 [33]. The concept of "prefecture tier" offers valuable insights into the business appeal, economic vitality, and development potential of different cities in mainland China. In this study, we rely on prefecture-tier classification when discussing the geographic propagation of infectious disease transmission. The prefecture tier classification is based on the "City Commercial Charm Ranking", published annually by the Yicai Media Group [34]. Beijing, Shanghai, Guangzhou and Shenzhen are categorised as "super-tier" cities. There were 15, 30, 70, 90, and 128 cities in tiers one through five, respectively. More details can be found in Figs CC and DD, Tables H–M in S1 Appendix.

Using prefecture tier, we could analyse the effect of cities in different prefecture tiers and the impact percentage (*K)* of a prefecture tier within the same or on a different prefecture tier:


$$\begin{array}{c} \begin{array}{c} K_{m\to n,\;t}=\frac{\sum_{i\in m,\;j\in n}a_{ij,t}}{\sum_{i\in m,\;j\in C}a_{ij,t}} \end{array} \end{array}$$ (2)

where *m* and *n* are different prefecture tiers; *t* is the elapsed time; C is the set of all 366 prefectures included in our study. For example, $K_{\text{1}\to \text{3}}=\text{6}\text{0}\%$ means 60% of the outgoing mobility pathways that may involve an infectious individual if the outbreak initiated in a tier-1 city are directed at tier-3 cities.

### Sensitivity and uncertainty analyses

We assessed robustness through one-way sensitivity analyses (Method M in S1 Appendix) and an additional joint probabilistic uncertainty analysis (Method N in S1 Appendix). In the latter, key parameters governing the reproduction number distribution, serial interval distribution, and migration probabilities were jointly sampled using Latin hypercube sampling to evaluate whether the main spatial risk patterns were robust to simultaneous uncertainty in model inputs.

### Use of AI tools

During the preparation of this work, the authors used ChatGPT to assist with language editing of the manuscript text. All AI-assisted content was reviewed, verified, and edited by the authors. The authors take full responsibility for the content of the manuscript.

## Results

### Model performance in real-world outbreaks

**Fig 2:**
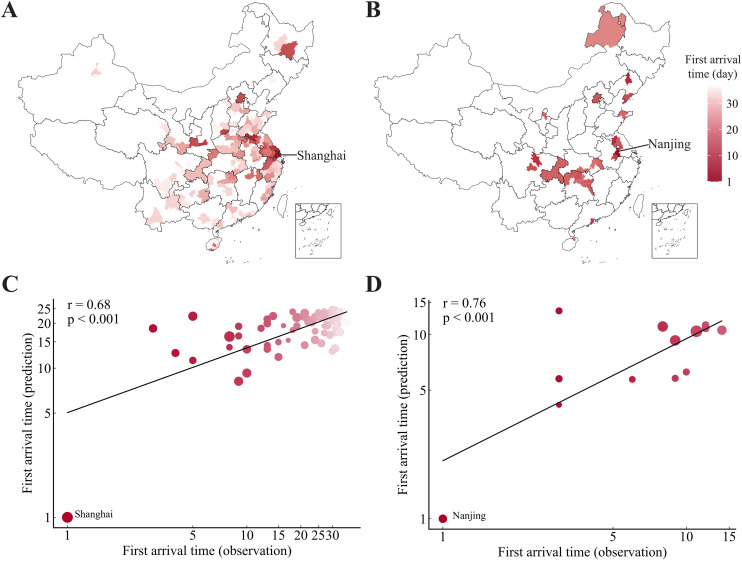
Observed and predicted first arrival times during two SARS-CoV-2 outbreaks in China. **(A)** Spatial distribution of cities affected during the Shanghai outbreak (March 1–April 5, 2022). **(B)** Spatial distribution of cities affected during the Nanjing outbreak (July 20–August 5, 2021). Darker shading indicates shorter intervals between the first reported case in the origin city and subsequent detection in other cities. **(C)** Comparison between observed and mean predicted first arrival times for 79 cities affected by the Shanghai outbreak. Each dot represents a city; symbol size reflects its population. **(D)** Comparison between observed and mean predicted first arrival times for 13 cities affected by the Nanjing outbreak. Strong positive correlations were observed for both Shanghai (Pearson correlation test, *r* = 0.68, 95% CI [0.54,0.79], *p* < 0.001) and Nanjing (Pearson correlation test, *r* = 0.76, 95% CI [0.35,0.92], *p* < 0.001) outbreaks, showing that our modelling captured the main spatiotemporal diffusion pattern of outbreaks. The basemap shapefiles were downloaded from the Chinese Resource and Environmental Science Data Platform (http://www.resdc.cn/, https://doi.org/10.12078/2023010102).

We evaluated the transmission patterns of two well-documented outbreaks of respiratory infectious diseases in China: the Omicron outbreak in Shanghai (1 March–5 April 2022) and the Delta outbreak in Nanjing (20 July–5 August 2021). Extensive nucleic acid amplification testing (NAAT) during both events enabled the precise identification of the first confirmed case in each affected city. Observed first arrival times ($T_{\text{obs}}$) were recorded for all cities involved (Fig 2A and 2B). The Omicron outbreak that originated in Shanghai spread rapidly, reaching 121 cities across almost all provinces within 36 days, whereas the Delta outbreak from Nanjing extended to 28 cities over 17 days. To assess whether the analytical framework could reproduce these observed dynamics, predicted first arrival times ($T_{\text{pre}}$) were derived from spatial diffusion estimates. Predicted and observed timings showed strong agreement in both Shanghai (*r* = 0.68, 95% CI [0.54,0.79], *p* < 0.001) and Nanjing (*r* = 0.76, 95% CI [0.35,0.92], *p* < 0.001) outbreaks (Fig 2C and 2D). These significant and high correlations suggest that the modelling accurately reflected the spatiotemporal progression of outbreaks, supported by the availability of detailed case data. To complement the correlation-based assessment, absolute prediction error was additionally evaluated using the MAE and RMSE, with detailed results provided in Fig A in S1 Appendix. The consistency between correlation and error-based metrics suggests that the model captures both the relative ordering and the magnitude of arrival times without substantial systematic bias. Further validation was presented in Methods G and H, Results C–E in S1 Appendix.

### Origin identification based on correlation analysis

**Fig 3:**
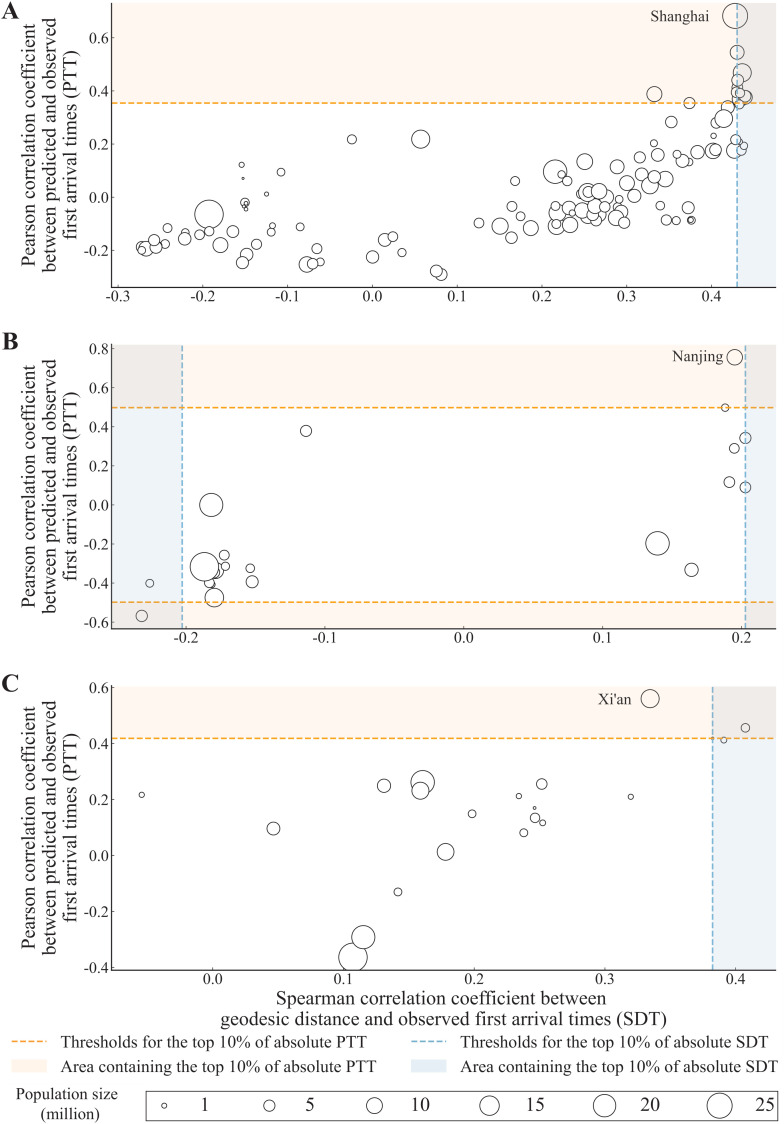
Identification of outbreak origins based on correlations between predicted and observed arrival times and by geodesic distance. Correlations between observed and predicted first arrival times were compared with correlations between observed arrival times and geodesic distance for the outbreaks in Shanghai **(A)**, Nanjing **(B)**, and northwestern China **(C)**. Each dot represents a candidate city, and the symbol size corresponds to the resident population. Dashed lines indicate thresholds for the top 10% of absolute correlation coefficients based on predicted arrival times (orange) or geodesic distance (blue). Shaded areas highlight cities within these top 10% thresholds. Shanghai showed the highest correlation (Pearson correlation test, *r* = 0.68, 95% CI [0.54,0.79], *p* < 0.001), consistent with its role as the origin of the Omicron outbreak. Nanjing was correctly identified as the origin of the Delta outbreak (Pearson correlation test, *r* = 0.76, 95% CI [0.35,0.92], *p* < 0.001), and Xi’an was identified as the most probable origin of the northwestern China outbreak (Pearson correlation test, *r* = 0.56, 95% CI [0.013,0.85], *p* = 0.046). Overall, the model-data correlation-based approach provided a more reliable identification of outbreak origins than the distance-based method.

Building on the strong correlations between predicted and observed first arrival times, we further examined whether this analytical approach could identify the origin of each outbreak. For each of the 366 cities in China, simulated diffusion patterns were generated assuming that the outbreak began there, and the correlation between the predicted and observed first arrival times was calculated. The city with the highest correlation was inferred as the most probable origin for each epidemic. As expected, Shanghai showed the strongest correlation (*r* = 0.68, 95% CI [0.54,0.79], *p* < 0.001) among all candidate cities, consistent with the known origin of the Omicron outbreak (Fig 3A). Similarly, Nanjing, the known source of the Delta outbreak, also demonstrated the highest correlation (*r* = 0.76, 95% CI [0.35,0.92], *p* < 0.001) (Fig 3B). Encouraged by these results, we applied the same analysis to a subsequent outbreak in northwestern China, where the first reported cases had travel histories spanning multiple provinces. Despite this complexity, the analysis identified Xi’an as the most probable origin (*r* = 0.56, 95% CI [0.013,0.85], *p* = 0.046) (Fig 3C), consistent with epidemiological reports from that period. This finding indicates that the approach can reasonably infer likely outbreak origins even in settings of uncertain transmission pathways. For comparison, we also applied a geodesic distance-based method to identify possible origins. When cities with the top 10% of absolute correlation coefficients were selected, neither Shanghai nor Nanjing appeared as origins. From the correlation-based ranking, Shanghai and Nanjing emerged as the most likely outbreak origins, although the differences between the top-ranked candidate cities were not always statistically distinguishable (Result A in S1 Appendix). The geodesic distance approach produced weaker and less distinguishable correlations (around *r* = 0.43, for the Shanghai outbreak, all p > 0.05 for Nanjing), making it difficult to pinpoint the source (Fig F in S1 Appendix). In contrast, the model-data correlation approach accurately identified the true origins of the Shanghai and Nanjing outbreaks, indicating stable performance across distinct epidemic settings.

### Risk assessment of respiratory infectious disease transmission

**Fig 4:**
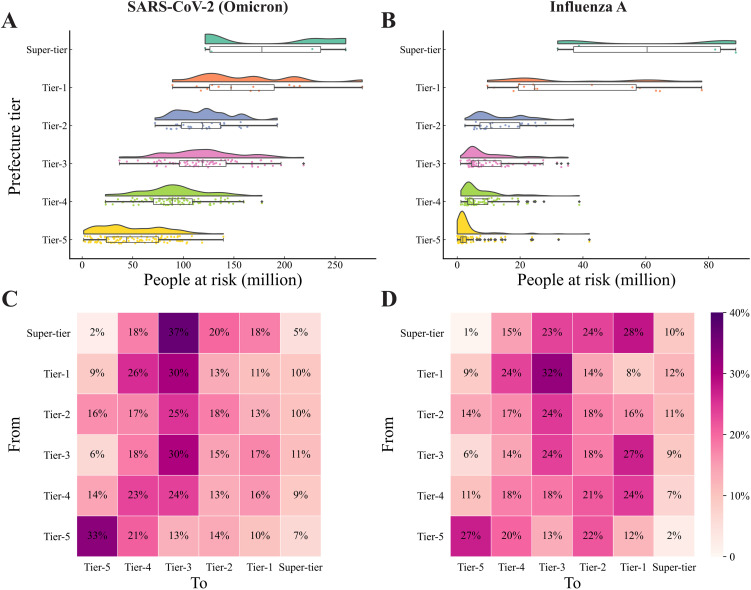
Epidemic risk by prefecture tier for SARS-CoV-2 and Influenza A transmission with 14 days. **(A, B)** Epidemic risk of cities in each prefecture tier for Omicron (A) and Influenza A (B) transmission, assuming 100 initial cases and a 14-day simulation period. Raincloud plots show the distribution of city-level epidemic risk within each tier. Significant differences in risk were observed across tiers for both pathogens (Kruskal–Wallis test, *p* < 0.001). **(C, D)** Heatmaps showing the probability of transmission between prefecture tiers for Omicron **(C)** and Influenza A **(D)**. Darker colours indicate a higher proportion of affected cities in the corresponding tier, illustrating patterns of within-tier and cross-tier spread.

We estimated the potential transmission risk of respiratory infectious diseases across cities in mainland China by applying an agent-based branching-process framework to simulate the early spread of SARS-CoV-2 (Omicron variant) and Influenza A. For each city, the simulation was initiated with 100 seed infections and run for 7, 14, and 21 days, using disease-specific basic reproduction numbers ($R_{\text{0}}$) and serial intervals (Table 1). A risk index ($R_{i}$) was used to quantify the potential impact of each origin city in generating secondary spread, and results were aggregated at the prefecture-tier level, a classification reflecting the economic vitality and development potential of Chinese cities. Within 14 days, substantial differences in epidemic risk were observed among prefecture tiers for both Omicron and Influenza A (*p* < 0.001; Fig 4A and 4B). Differences in $N_{i}^{\text{risk}}$ across prefecture tiers were highly significant in Kruskal–Wallis tests and remained robust in permutation-based analyses (Method P and Result H in S1 Appendix). This pattern was consistent at 7 and 21 days (Figs I and J in S1 Appendix), with the highest transmission risk observed in super-tier cities. For Omicron, risk differentials between higher and lower tiers were most pronounced during the initial 7 days and gradually narrowed over time, with risk distributions across city tiers rapidly converging and overlapping as outbreaks progressed. In contrast, Influenza A showed a more stable stratified pattern, with limited overlap between higher and lower tiers throughout the simulation period. Analyses with alternative initial case numbers (Figs N and O in S1 Appendix), migration probabilities (Result F in S1 Appendix) and importation thresholds (Result G in S1 Appendix) yielded comparable results, supporting the robustness of these findings. The robustness of these tier-specific spatial risk patterns was further supported by a joint probabilistic uncertainty analysis that simultaneously varied key epidemiological and migration parameters, with qualitatively consistent results across sampled parameter sets (Result I in S1 Appendix).

Using prefecture-tier classification, we further assessed inter-tier transmission patterns. At day 7, both SARS-CoV-2 Omicron and influenza A showed similar early patterns: most risk was directed to cities within the same tier or to neighbouring tiers (Fig I in S1 Appendix), indicating that initial spread remained concentrated among comparable socioeconomic groups. From day 14 onward, the two pathogens diverged (Fig 4C and 4D). Omicron displayed a progressive shift in the distribution of epidemic risk. As transmission progressed, Omicron showed a clear redistribution of risk from higher-tier cities toward lower-tier cities, with tier 3 becoming the predominant recipient by day 21 (Fig J in S1 Appendix). In contrast, influenza A retained a more tier-bound structure throughout the observation window. Even at day 21, risk remained primarily concentrated in the same-tier or adjacent-tier cities (Fig J in S1 Appendix). These findings indicate that although both pathogens begin with locally stratified spread, Omicron evolves toward a pattern that bridges the socioeconomic hierarchy. In contrast, influenza A remains restricted within the structure defined by urban hierarchy.

## Discussion

In this study, we integrated large-scale human mobility data with an agent-based branching process model to characterise the spatial diffusion of respiratory infectious diseases across mainland China. Analyses of three COVID-19 outbreaks and simulation-based risk assessments for SARS-CoV-2 (Omicron variant) and influenza A showed that mobility patterns alone capture much of the early spatial spread. The strong agreement between predicted and observed first arrival times, together with accurate identification of outbreak origins, supports the empirical plausibility of a mobility-driven modelling approach. Building on this validated framework, our risk assessment revealed systematic differences in transmission potential across China’s urban tiers, with distinct vulnerability profiles for the two pathogens. These findings demonstrate that human mobility and the hierarchical structure of urban areas jointly shape spatial transmission risk.

Our findings yield two major insights into the propagation of respiratory infectious diseases. First, city-level introduction times during outbreaks could be largely reconstructed using mobility data alone, without calibrating to outbreak-specific epidemiological parameters. This consistency across distinct events, including the Delta and Omicron outbreaks, suggests that routine human mobility exerts a substantial influence on early spatial diffusion [35–38], often playing a more decisive role than pathogen-specific characteristics in shaping the timing and ordering of introductions, particularly in settings where high-resolution mobility data accurately reflect population movements. Second, the model-based correlation approach markedly outperformed distance-based methods for origin identification. Geodesic distance, though widely used as a proxy for spatial connectivity, could not distinguish true outbreak origins from other candidate cities because it fails to reflect directional and heterogeneous travel flows [10,37]. In contrast, our mobility-informed framework incorporates both directionality and travel volume, yielding a more realistic representation of early transmission pathways. Collectively, these results reinforce the growing evidence that high-resolution mobility data enhances epidemic situational awareness and supports rapid response efforts.

The risk assessment analysis revealed distinct spatial diffusion patterns for respiratory infectious diseases like SARS-CoV-2 and influenza A, reflecting their contrasting epidemiological characteristics. Influenza A exhibited a stable and stratified diffusion profile, with transmission largely confined within similar or adjacent urban tiers. This pattern suggests that pathogens with relatively lower transmissibility remain strongly shaped by structural features of the mobility network, leading to slower and more tier-bound spatial expansion [39,40]. In contrast, SARS-CoV-2 (Omicron variant) demonstrated a markedly different pattern: epidemic risk initially concentrated in super-tier and higher-tier cities but rapidly expanded toward lower-tier cities, showing a pronounced downward hierarchical spread. This accelerated cross-tier propagation reflects Omicron’s high transmissibility and its ability to overcome socioeconomic and structural barriers that typically constrain mobility-driven diffusion [39,41]. These contrasting diffusion profiles indicate that spatial transmission risk reflects an interaction between pathogen characteristics and the socioeconomic hierarchy of cities [42–44]. Notably, the rapid cross-tier spread of SARS-CoV-2 (Omicron) suggests that surveillance systems concentrated in major metropolitan areas may be insufficient to capture early signals during highly transmissible outbreaks. This insight supports the development of pathogen-specific and tier-wide surveillance strategies.

Our study provides a flexible and scalable analytical framework with several methodological strengths. First, the model is parsimonious and relies solely on routinely collected mobility data, without requiring outbreak-specific epidemiological curve fitting. This makes the framework readily applicable during emerging respiratory infectious disease outbreaks and supports real-time risk stratification when information is limited. Its ability to accurately identify outbreak origins across multiple epidemiological contexts further highlights the model’s utility for real-time outbreak investigation, particularly when early transmission pathways are uncertain. Second, the validation benefited from the unusually comprehensive outbreak data collected during China’s stringent COVID-19 control period, when systematic testing and timely reporting ensured near-exhaustive case identification [45–47]. This high-quality dataset enabled rigorous validation of spatial diffusion pathways and strengthened confidence in the model’s robustness.

Our modelling framework builds on established work on epidemic spread in metapopulation systems and mobility networks. Early theory showed that heterogeneous coupling and host movement can govern invasion across connected subpopulations, while subsequent work examined arrival time distributions and multiscale mobility effects [48–50]. GLEAM further extended this tradition into a global epidemic-simulation platform [51]. We therefore do not consider the general use of mobility-coupled branching-process or metapopulation models to be novel. Instead, our contribution lies in adapting this modelling tradition to a specific empirical and inferential setting: early city-level spread within China, using daily intercity mobility data for 366 cities and validation against observed first arrival times from multiple outbreaks. In this context, our framework is designed not for general epidemic forecasting but for reconstructing first arrival patterns, supporting origin inference, and comparing how pathogens with different transmission characteristics propagate across the urban hierarchy. We view this as a mobility-coupled stochastic diffusion model, with value in its empirical grounding, interpretability, and direct validation against observed spatial spread [52].

Real-time or near-real-time mobility data have been used for more than a decade to study the spatial spread of infectious diseases. Early studies showed that mobile phone-derived movement data could identify sources and sinks of malaria parasite importation and improve the prediction of the geographic spread and timing of dengue epidemics, demonstrating that human mobility can provide epidemiologically meaningful information beyond geographic distance alone [53,54]. During the COVID-19 pandemic, aggregated mobility data were further recognised as valuable for monitoring behavioural change and assessing intervention effects in near-real time [55]. Methodological work has also highlighted the importance of explicitly representing mobility networks for epidemic simulation [56]. In China, Kraemer and colleagues used Baidu mobility data to show that movement out of Wuhan strongly explained the early spatial dissemination of pre-Alpha SARS-CoV-2 across China, although this relationship weakened after major control measures were introduced; our findings are consistent with this broader conclusion that mobility structure is highly informative for early geographic spread, but extend it by showing that, under the later dynamic zero-COVID period, realised diffusion patterns differed substantially between Delta and Omicron despite being embedded in the same intercity mobility system [35]. Our interpretation is also consistent with the broader conclusion of Perofsky and colleagues, who showed that the mobility measures most closely associated with respiratory virus transmission can vary markedly across pathogens and over time. However, that study focussed on multiple local mobility indicators within Seattle, whereas we analysed intercity flows across mainland China. Both studies suggest that the epidemiological role of mobility is not fixed but depends on pathogen characteristics, epidemic context, and spatial scale [57]. Taken together, these findings suggest that mobility is a crucial determinant of early spread, but that its form and magnitude of epidemiological relevance vary by pathogen and scale.

The key assumptions underlying our model are supported by different levels of empirical evidence. The assumption that intercity human mobility is a primary driver of early city-level spatial spread has a strong empirical basis, supported by extensive prior work using mobile-phone and platform-based mobility data, as well as by our own validation against observed first-arrival times across multiple outbreaks. Representing transmission as a branching process with a negative binomial offspring distribution is also well supported, as it is a standard, empirically grounded approach for capturing individual-level heterogeneity and superspreading. The use of the serial interval as the temporal scale for comparing arrival times is justified by our validation target, which is based on the timing of detected cases rather than unobserved infection events. By contrast, two assumptions rest on a more approximate basis and are therefore treated as sensitivity targets rather than fixed assumptions: the sigmoid travel-probability function anchored to an estimated baseline daily migration probability for Shanghai, which is an empirically informed approximation rather than a directly observed quantity; and the prefecture-tier classification, which captures commercial and functional urban hierarchy rather than epidemiological vulnerability per se. Across these assumptions, our sensitivity and uncertainty analyses suggest that assumptions with weaker empirical support may influence the magnitude of estimates but do not materially alter the qualitative cross-tier conclusions.

Our findings carry several important policy implications. Mobility-informed early warning systems could help identify cities at the highest risk of receiving infected individuals, enabling rapid deployment of testing resources, travel advisories, or targeted surveillance. Differences in diffusion trajectories between pathogens also underscore the need for pathogen-specific preparedness strategies: highly transmissible pathogens may require broader geographic alert zones in the early phase of spread, whereas pathogens with more stratified diffusion may benefit from tier-focussed interventions. Beyond pathogen-specific responses, mobility-based risk assessment offers a practical foundation for strengthening multi-level surveillance and situational awareness within China’s public health system. The ability to quantify how infections propagate through the urban network can inform more adaptive allocation of diagnostic capacity, risk communication, and inter-provincial coordination. Furthermore, because the framework depends primarily on mobility patterns and requires only minimal pathogen-specific inputs (such as an approximate reproduction number and serial interval), it can provide an early, coarse assessment of spatial transmission risk even for newly emerging pathogens, including potential Disease X, when epidemiological information is scarce and rapid situational awareness is essential.

Our findings should be interpreted as reflecting comparative early spatial diffusion potential, rather than comparative local epidemic severity after introduction. We focussed on first arrival timing because it is more directly linked to the mobility network, transmission generation characteristics, and stochastic seeding process during the earliest phase of spread, and is therefore less sensitive than outcomes such as peak incidence, cumulative incidence, epidemic intensity, or final attack rate to city-specific heterogeneity in prior infection, vaccination, climatic forcing, and the timing and intensity of local interventions. These factors remain important for epidemic growth after introduction. Available evidence suggests that widespread prior SARS-CoV-2 infection in China remained limited overall before late 2022, although sporadic outbreaks could still have generated some local heterogeneity in infection-acquired immunity. Likewise, COVID-19 vaccine uptake in China was high during the study period, but harmonised city-specific vaccination data were not available across all study locations and time periods. The comparison between the Delta outbreaks and the Shanghai Omicron outbreak should also be interpreted within the broader dynamic zero-COVID framework, rather than as a contrast between intensive control for Delta and broadly abandoned control for Omicron. Influenza circulation in China was also substantially perturbed during the COVID period, with delayed resurgence and altered seasonal patterns, suggesting that the immunological landscape was unlikely to represent a typical pre-pandemic seasonal steady state. Taken together, these considerations suggest that incorporating the infection landscape, vaccination landscape, climatic drivers, and heterogeneous interventions would be essential for studies of peak size, epidemic intensity, or final attack rate, but is less critical for the arrival time-based question addressed here.

We did not explicitly model city-specific prior infection, vaccination coverage, time-varying intervention intensity, or climatic forcing because our objective was to study early intercity diffusion and first arrival timing rather than local epidemic burden after introduction. If the framework were extended to outcomes such as peak incidence, cumulative incidence, epidemic intensity, or final attack rate, these factors would need to be incorporated explicitly. They could alter the relative ranking of cities and pathogens. An exploratory post-arrival local transmission analysis was therefore added in Method Q, Result J in S1 Appendix to illustrate how these factors could influence local epidemic trajectories once introduction had occurred.

It is important to acknowledge several limitations in this study. First, mobility data derived from Baidu users may not fully capture population movement across all demographic groups [58,59], particularly older adults or individuals with limited smartphone access. Although Baidu Migration aggregates movement information from more than 1.1 billion active users (covering over 75% of the national population), sampling bias may still influence estimated travel probabilities [60]. Future work could incorporate complementary mobility sources (e.g., telecommunications or transport networks) to strengthen generalisability [61]. Second, the model focuses on early-stage diffusion and does not account for behavioural adaptations, increasing stringency of public health and social measures, or pathogen evolution, all of which may alter transmission dynamics in later stages of an outbreak [62–64]. Third, only first arrival times could be reliably extracted for validation, as other epidemiological indicators, such as outbreak magnitude, were affected by non-pharmaceutical interventions (NPIs) and therefore incomplete. Nonetheless, the model’s strong and consistent performance across multiple outbreak contexts supports its robustness and external validity. The mobility data used in the main analysis were derived from China’s "dynamic zero-COVID" period and therefore reflect a policy-conditioned mobility regime. In supplementary analyses (Method L and Result B in S1 Appendix), however, intercity mobility matrices were highly similar across the Nanjing Delta outbreak, the Shanghai Omicron outbreak, the full year 2021, and the full year 2023, consistent with prior evidence that China’s intercity transportation network exhibits structural stability even following large shocks such as the initial COVID-19 outbreak [58]. And repeating the risk assessment using post-zero-COVID mobility data from 2023 yielded qualitatively consistent results. In particular, Omicron continued to show faster and broader cross-tier dissemination, whereas influenza A remained more stratified within the urban hierarchy. These findings suggest that the main qualitative conclusions are not solely specific to the mobility conditions of 2021–2022. We therefore interpret China as a large-scale case study and the present analysis as a proof-of-concept for a transferable framework, rather than as implying direct quantitative generalisability of the China-specific estimates. Additionally, although the prefecture-tier classification captures broad differences in urban scale and connectivity, it is based on commercial and functional characteristics and may not fully capture epidemiological vulnerability, which depends on additional factors such as demographic structure, healthcare capacity, housing conditions, socioeconomic context, and local public health response capacity. However, because our analysis focuses on mobility-driven early spatial diffusion, the use of this classification is unlikely to materially affect the main conclusions regarding cross-tier differences in transmission risk.

In conclusion, this study presents a practical, robust framework for simulating the complex spatial dynamics of epidemic spread using publicly available transportation network data. The model’s successful application to real-world outbreaks demonstrates its practical utility for capturing large-scale transmission processes of respiratory infectious diseases. By integrating human mobility and socioeconomic structure, the framework enables spatiotemporal risk assessment and early warning at both national and subnational scales. These features make it a valuable decision-support tool for guiding data-driven, region-specific public health interventions. Beyond its immediate applicability, this approach also provides a methodological foundation for developing integrated, mobility-informed surveillance systems to enhance preparedness and resilience against future emerging infectious disease threats.

## Supporting information

S1 Appendix Supplementary material for “Assessing spatial transmission risk of respiratory infectious diseases across cities of different socioeconomic tiers in China: a modelling study”. (DOCX)

## Abbreviations

MAE: mean absolute error
NAAT: nucleic acid amplification testing
NPIs: non-pharmaceutical interventions
PHSM: public health and social measures
RMSE: root mean square error
SI: serial interval
