# Supplementary material for: Assessing spatial transmission risk of respiratory infectious diseases across cities of different socioeconomic tiers in China: A modelling study
Source: PLoS Med. 2026 Jul 20;23(7):e1005172. doi: 10.1371/journal.pmed.1005172 (PMC13384292; doi:10.1371/journal.pmed.1005172)
Supplement: S1 Appendix — (DOCX) [file pmed.1005172.s001.docx]

**Supplementary material for ‘Assessing spatial transmission risk of respiratory infectious diseases across cities of different socioeconomic tiers in China: a modelling study’**

Wenjie Li^1,2^, Wei Yang^1*^, Yang Liu^3,4,5&^, Ye Yao^2,6,7&*^

^1^ Research Institute of Intelligent Complex Systems, Institute of Science and Technology, Fudan University, Shanghai, China.

^2^ Department of Biostatistics, School of Public Health, Fudan University, Shanghai, China.

^3^ PATH, London, United Kingdom.

^4^ Centre for Mathematical Modelling of Infectious Diseases, London School of Hygiene & Tropical Medicine, London, United Kingdom.

^5^ Department of Infectious Disease Epidemiology, Faculty of Epidemiology and Population Health, London School of Hygiene & Tropical Medicine, London, United Kingdom.

^6^ Shanghai Institute of Infectious Disease and Biosecurity, Fudan University, Shanghai, China.

^7^ Key Laboratory of Public Health Safety of Ministry of Education, School of Public Health, Fudan University, Shanghai, China.

& Equally contributed last author

*yangwei@fudan.edu.cn; yyao@fudan.edu.cn.

**Table of contents**

[Supplementary Methods 3](#_Toc233829387)

[Method A. Observed outbreak scenarios for model validation 3](#_Toc233829388)

[Method B. First confirmed case collection 3](#_Toc233829389)

[Method C. Human mobility data 3](#_Toc233829390)

[Method D. Migration probability 4](#_Toc233829391)

[Method E. Serial interval 4](#_Toc233829392)

[Method F. Stochastic simulation procedure 5](#_Toc233829393)

[Method G. Validation against cumulative affected cities and temporal growth of spatial spread 5](#_Toc233829394)

[Method H. Arrival time distribution and mixed-effects modelling 5](#_Toc233829395)

[Method I. Origin identification using log-transformed Pearson correlation 6](#_Toc233829396)

[Method J. Spearman correlation in geodesic distance analysis 7](#_Toc233829397)

[Method K. Additional uncertainty analyses for origin identification 7](#_Toc233829398)

[Method L. Robustness to an alternative post-zero-COVID mobility regime 8](#_Toc233829399)

[Method M. Sensitivity analysis 8](#_Toc233829400)

[Method N. Joint probabilistic uncertainty analysis 9](#_Toc233829401)

[Method O. Alternative definitions of $N_{i}^{\mathrm{risk}}$ based on importation thresholds 9](#_Toc233829402)

[Method P. Permutation-based inference for tier-level comparisons 10](#_Toc233829403)

[Method Q. Exploratory post-arrival local transmission analysis 10](#_Toc233829404)

[Supplementary Results 12](#_Toc233829405)

[Result A. Uncertainty analyses for origin identification 12](#_Toc233829406)

[Result B. Robustness of tier-specific diffusion patterns under 2023 mobility conditions 12](#_Toc233829407)

[Result C. Validation against cumulative affected cities and temporal growth of spatial spread 12](#_Toc233829408)

[Result D. Arrival time distributions 13](#_Toc233829409)

[Result E. Mixed-effects model 13](#_Toc233829410)

[Result F. Cross-tier comparisons under alternative baseline migration probabilities for Shanghai 13](#_Toc233829411)

[Result G. Sensitivity of $N_{i}^{\mathrm{risk}}$ to alternative importation thresholds and initial seeding sizes 14](#_Toc233829412)

[Result H. Permutation-based assessment of cross-tier differences in $N_{i}^{\mathrm{risk}}$ 14](#_Toc233829413)

[Result I. Robustness of tier-specific risk patterns under joint parameter uncertainty 15](#_Toc233829414)

[Result J. Exploratory post-arrival local transmission analysis 15](#_Toc233829415)

[Supplementary Figures 16](#_Toc233829416)

[Supplementary Tables 51](#_Toc233829417)

[References 69](#_Toc233829418)

**Supplementary Methods**

**Method A. Observed outbreak scenarios for model validation**

We examined three major COVID-19 outbreaks that occurred in mainland China between 2021 and 2022, originating in Shanghai, Nanjing, and northwestern China.

- The Omicron outbreak in Shanghai began with the first confirmed case on 1 March 2022. Localised and phased lockdown measures were introduced from 28 March (Pudong) and 1 April (Puxi). Shanghai officially announced the continuation and consolidation of strict city-wide static management on 5 April, marking the point at which population mobility dropped to uniformly low levels across the entire municipality. Our analysis focuses on the period prior to the implementation of full-scale city-wide static management, so we defined the outbreak period from 1 March to 5 April 2022. During this time, infections spread to 121 cities.
- The Delta outbreak linked to Nanjing Lukou Airport started on 20 July 2021 and affected 28 cities. The last confirmed case occurred on 5 August, so the study period was defined as from 20 July to 5 August 2021.
- The origin of the Delta outbreak in northwestern China was less clear because a tour group travelled across multiple cities during the National Day holiday. The first confirmed case was reported in Xi’an on 17 October 2021, and the outbreak spread to 26 cities by 3 November. The outbreak period was therefore defined as 17 October to 3 November 2021.

**Method B. First confirmed case collection**

Daily confirmed case data for each outbreak were obtained from the National Health Commission and provincial/municipal health authorities. For every affected city, we extracted the date of its first confirmed infection linked to the outbreaks in Shanghai, Nanjing, or northwestern China. Cases were included if (i) health authorities explicitly classified them as outbreak-related, or (ii) epidemiological investigation, such as documented travel history, transportation records, or close-contact tracing, indicated that the infection originated from one of the three source regions. The first arrival time for each city was defined as the interval between the date of the first confirmed case in the origin city and the date of the first epidemiologically linked case in the affected city. This procedure ensured accurate and consistent identification of city-level first arrival times for validating model predictions.

**Method C. Human mobility data**

Human mobility was quantified using the Baidu Migration Index, which is derived from anonymised location-based service signals from over 1.1 billion mobile devices. We obtained both the migration scale index for each city and the proportion of outflow directed to each destination city from the Baidu Qianxi platform. These data were used to characterise intercity mobility as follows:

$$\begin{aligned} {M_{ij,t}=Q}_{i,t,out}\times x_{ij,t}\#\left( \text{1} \right) \end{aligned}$$

$$\begin{aligned} M_{ij}=\frac{\left( \sum_{t=1}^{T} M_{ij,t} \right)}{T}\#\left( \text{2} \right) \end{aligned}$$

$$\begin{aligned} p_{M_{ij}}=\frac{M_{ij}}{\sum_{j} M_{ij}}\#\left( \text{3} \right) \end{aligned}$$

where $Q_{i,t,out}$ denotes the migration scale index representing the total outflow from city $i$ at time $t$, and $x_{ij,t}$ denotes the proportion of that outflow travelling from city $i$ to city $j$. The quantity $M_{ij}$ therefore represents the average outflow volume from city $i$ to city $j$ over the period $T$, and $p_{M_{ij}}$ is the corresponding probability of travel. Using these Baidu Qianxi data, we constructed 366$\times$366 transition matrices for each COVID-19 outbreak scenario, corresponding to the relevant time periods.

Functionally comparable mobility data products are available in other settings, including Meta's Data for Good Movement Range maps, Google's archived Community Mobility Reports, SafeGraph and Cuebiq aggregated mobility data in the United States, and mobile-network-operator origin-destination matrices in several European and Latin American countries, and would support implementation of the same analytical framework with appropriate local adaptation.

**Method D. Migration probability**

A sigmoid function of the city-level migration scale index determined the probability that an infected individual travelled on a given day. For each city $i$, the average outflow index during the outbreak period was calculated as $\bar{Q}_{i}=\sum_{t=1}^{T} Q_{i,t,out}/T$, and $\bar{Q}_{max}=max\left( \left\{ \bar{Q}_{i} \right\} \right)$. To map this mobility intensity to a migration probability $p_{i}$, we defined a sigmoid function:

$$\begin{aligned} S\left( \bar{Q}_{i} \right)=\frac{1}{1+e^{-\bar{Q}_{i}+{\bar{Q}_{max}}/2}}\#\left( \text{4} \right) \end{aligned}$$

and $p_{i}$ was assumed to be proportional to this value $p_{i}\propto S(\bar{Q}_{i})$, $\frac{p_{i}}{p_{j}}=\frac{S(\bar{Q}_{i})}{S(\bar{Q}_{j})}$. We set the migration probability of Shanghai ($p_{Sh}$) at 1%[1] as the baseline. Thus, the migration probability for city $i$ is,

$$\begin{aligned} p_{i}=\frac{S\left( \bar{Q}_{i} \right)}{S\left( \bar{Q}_{Sh} \right)}\times1\%\#\left( \text{5} \right) \end{aligned}$$

To parameterise the migration probability function, we set the baseline daily migration probability for Shanghai at 1%. This value was not arbitrarily assigned. Instead, it was approximately estimated using external commuting-flow data for the Yangtze River Delta region together with the Baidu migration index. Specifically, the commuting report provided the total population moving from Shanghai to other cities in the Yangtze River Delta, and the Baidu migration index provided the proportion of Shanghai’s total outflow directed to those cities. Combining these two sources allowed us to approximately back-calculate Shanghai’s total out-migration volume. Dividing this inferred total outflow by Shanghai’s resident population yielded an estimated daily per-capita out-migration probability close to 1%. Because no publicly available dataset provides a directly observed individual-level daily migration probability for Shanghai, this value should be interpreted as an empirically informed approximation used to anchor the migration probability function rather than as a directly observed quantity. To assess robustness to this assumption, we additionally examined a range of baseline values from 0.5% to 1.5%.

**Method E. Serial interval**

We emphasise that the serial interval is used here because our validation target, the first arrival time in each city, is derived from the timing of the first detected case (i.e., based on symptom onset/reporting), not from the timing of infection. The serial interval, which describes the interval between successive symptom onsets in a transmission chain, is therefore the natural and directly observable temporal scale on which simulated and observed arrival times can be compared. The serial interval is not used as a proxy for the generation time; generation intervals, being infection-to-infection, are not directly observable in routine surveillance data and would introduce additional unobservable parameters (incubation and reporting delays) if used in place of the serial interval.

**Method F. Stochastic simulation procedure**

To quantify stochastic variability, each outbreak-origin scenario was repeated 10,000 times. In each replicate, we tracked whether each destination city was seeded within the simulation horizon and, if so, recorded its first arrival time. Cities not seeded during the simulation period were treated as not reached at the replicate level. If a city did not experience any infected introduction during the simulation period, it was considered not reached within the observation window, and no finite first arrival time was assigned, consistent with right-censoring within the finite simulation horizon. Analyses comparing observed and predicted first arrival times were restricted to cities with both an observed first arrival time and a finite simulated first arrival time within the study period. For the main validation analyses, the predicted first arrival time of each city was summarised as the arithmetic mean across simulations as the primary estimate of expected arrival timing. MAE, RMSE, and correlation coefficients were then computed by comparing these mean predicted arrival times with the observed first arrival times. For risk assessment, $N_{i}^{risk}$ was likewise summarised as the mean across simulations, and cities not seeded in a given replicate were not counted as contributing to transmission risk in that replicate.

**Method G. Validation against cumulative affected cities and temporal growth of spatial spread**

To extend model validation beyond first arrival time ordering, we assessed two complementary targets for the Shanghai outbreak: the total cumulative number of cities affected within a 30-day observation window, and the day-by-day temporal trajectory of cumulative spatial spread. A city was classified as affected on the day it first received at least one imported infection in a given simulation replicate. For each of the 10,000 simulation replicates, we computed the cumulative number of affected cities at each day from day 1 to day 30, and summarised the simulated distribution at each day as the mean and 95% simulation interval across replicates. The total cumulative count at day 30 was summarised across replicates as the mean and 95% simulation interval. The temporal growth trajectories of the observed and mean simulated cumulative affected cities were visualised separately from the observed and mean simulated counts across days. To formally compare the observed and simulated temporal growth patterns, we computed the mean cumulative number of affected cities across all 30 days for the observed data and for each simulation replicate separately. The observed mean was then compared against the distribution of simulated means using a non-parametric test.

**Method H. Arrival time distribution and mixed-effects modelling**

To account for stochastic variability in spatial spread, we analysed arrival times obtained from repeated stochastic simulations of each outbreak scenario. For each simulation run, the first arrival time was defined as the earliest time at which infection was introduced into a given destination city. Analyses were restricted to city–simulation pairs in which the city was reached within the simulation window, consistent with prior simulation-based approaches that condition on successful transmission.

We summarised the empirical distribution of observed first arrival times across cities and compared it with the distribution of simulated arrival times aggregated across stochastic runs. Summary statistics included the mean, median, and interquartile range.

To formally incorporate uncertainty in arrival times, we fitted a mixed-effects model with log-transformed arrival time as the dependent variable:

$$\log\left( T_{\mathrm{ijr}} \right)=\beta_{0}+\beta_{1}\mathrm{Pathogen}_{i}+\beta_{2}\log\left( \mathrm{Migration}_{\mathrm{ij}}+c \right)+\beta_{3}\mathbb{I}\left( \mathrm{Migration}_{\mathrm{ij}}=0 \right)$$

$$\begin{aligned} +\beta_{4}\log\left( {Geodesic distance}_{\mathrm{ij}} \right)+\sum_{k=2}^{6} \gamma_{k}\mathbb{I}\left( \mathrm{Tier}_{j}=k \right)+\beta_{5}\log\left( \mathrm{Population}_{j} \right)+u_{j}+\varepsilon_{\mathrm{ijr}}\#\left( 6 \right) \end{aligned}$$

where, $T_{\mathrm{ijr}}$ denotes the first arrival time for destination city $j$in simulation run $r$under scenario $i$; $\mathrm{Pathogen}_{i}$ indicates the outbreak variant (Shanghai Omicron vs Nanjing Delta); $\mathrm{Migration}_{\mathrm{ij}}$ represents population flow from the origin city to destination city $j$, with a small constant $c$added to allow log transformation; $\mathbb{I}\left( \mathrm{Migration}_{\mathrm{ij}}=0 \right)$ is an indicator for zero-flow connections; ${Geodesic distance}_{\mathrm{ij}}$ is the distance between origin and destination cities; $\mathrm{Tier}_{j}$ denotes the prefecture tier of the destination city; $\mathrm{Population}_{j}$ is the resident population size of the destination city; $u_{j}$ is a random intercept for destination city, assumed to follow a normal distribution; $\varepsilon_{\mathrm{ijr}}$ is the residual error term.

This model allows estimation of associations between arrival time and key structural determinants of spread while accounting for repeated simulations and unobserved city-level heterogeneity.

**Method I. Origin identification using log-transformed Pearson correlation**

To identify the most likely outbreak origin, we compared the simulated first arrival time pattern generated by each candidate origin with the observed first arrival time pattern across affected cities. Our assumption that the first arrival time ($t$) and diffusion distance ($D$) satisfy a generalised power-law relation is motivated by both the physical basis of spatial diffusion and the epidemiological characteristics of COVID-19 spread driven by human mobility. At a coarse-grained level, the geographic spread of COVID-19 can be viewed as a mobility-driven spatial propagation process. In classical diffusion theory[2], the mean squared displacement satisfies $\left\langle D^{2} \right\rangle=2\kappa t$, where $\kappa$ is the diffusion coefficient, implying $t\propto D^{2}$. Thus, the power-law form has a clear physical basis under ideal diffusion. However, real-world COVID-19 transmission does not occur in a homogeneous medium; instead, it unfolds on highly heterogeneous human mobility networks. As a result, epidemic spread may deviate from classical diffusion and exhibit anomalous diffusion-like behaviour. We therefore adopt the more general form $t=\beta D^{\alpha}$. When two variables are related by a nonlinear scaling law such as $t=\beta D^{\alpha}$, applying Pearson correlation directly to the raw data may not adequately capture their relationship. Taking logarithms yields $\ln t=\ln\beta+\alpha\ln D$, which linearises the scaling relation. Therefore, Pearson correlation computed on the log-transformed variables is more appropriate than correlation computed on the raw variables. We computed the Pearson correlation coefficient between the logarithm of the predicted first arrival time ($\lg T_{pre}$) and the logarithm of the observed first arrival time ($\lg T_{obs}$). This log–log correlation quantifies how well the model reproduces the relative ordering and scaling of spatial diffusion across cities, independent of the specific value of α. We used the logarithmic scale to capture similarity in spatial diffusion patterns on a scaling-based time scale, consistent with diffusion-based formulations and previous work on epidemic arrival times in mobility networks.

In the origin-identification analysis, the Pearson correlation on log-transformed arrival times was used primarily as a measure of similarity between observed and predicted arrival time patterns across cities. This choice was made because origin identification required not only agreement in the ordering of arrival times, but also preservation of their relative temporal spacing, which improved discrimination among candidate origins. By contrast, rank-based measures such as Spearman correlation retain ordinal information only and were therefore used for the geodesic-distance comparison, where we do not know the functional relationship between diffusion distance and time in the context of SARS-CoV-2 transmission.

For origin identification, only candidate origins with at least 10 cities included in the correlation analysis were ranked, to ensure that the ranking reflected a meaningful portion of the observed spatial diffusion pattern rather than small-sample artefacts. The empirical distributions of arrival times across cities appear highly skewed. To reduce reliance on classical parametric assumptions, statistical significance of the Pearson correlation was additionally assessed using permutation tests, and uncertainty in the correlation coefficients was quantified using bootstrap 95% confidence intervals.

**Method J. Spearman correlation in geodesic distance analysis**

To assess whether Pearson or Spearman correlation was more appropriate for the geodesic distance analysis, we examined the distributional properties of the variables and the functional form of their relationship (Fig E). The marginal distributions of both observed first arrival time and geodesic distance were right-skewed. To formally evaluate the bivariate normality assumption underlying Pearson correlation, we conducted the Henze–Zirkler multivariate normality test on the joint distribution of observed first arrival time and geodesic distance for each outbreak separately. Bivariate normality was rejected for both the Shanghai outbreak (HZ statistic = 3.59, p < 0.001) and the Nanjing outbreak (HZ statistic = 0.85, p = 0.03). The scatter plot of observed first arrival time against geodesic distance further confirms that the relationship between the two variables is at most weakly monotonic and conforms to no consistent parametric functional form. Additionally, the functional form of the relationship between geographic distance and first arrival time under SARS-CoV-2 transmission is not known a priori. Taken together, these considerations support the use of Spearman rank correlation for geodesic distance analysis, as it assesses monotonic association without requiring bivariate normality, without imposing assumptions about functional form, and with greater robustness to extreme values than Pearson correlation.

**Method K. Additional uncertainty analyses for origin identification**

To further evaluate uncertainty in origin identification, we performed three complementary analyses. First, for each candidate origin, uncertainty in the Pearson correlation estimate was quantified using permutation tests and bootstrap 95% confidence intervals. These analyses assessed whether the correlation between log-transformed observed and predicted first arrival times was stronger than expected under random reassignment of observed arrival times across cities, and quantified the sampling variability of the correlation estimate.

Second, to account for multiplicity arising from ranking all candidate origins and selecting the city with the highest correlation, we performed a max-statistic permutation test. For each outbreak, Pearson correlations between log-transformed observed and predicted first arrival times were first calculated for all eligible candidate origins, and the maximum observed correlation was recorded. We then permuted the observed first arrival times across cities, recalculated the correlations for all eligible candidate origins, and retained the maximum correlation from each permuted dataset. Repeating this procedure generated a null distribution for the origin-selection process itself. The permutation p-value was calculated as the proportion of permutations in which the maximum correlation was at least as large as that observed in the original data.

Third, to assess the stability of candidate-origin ranking, we performed a city-level bootstrap analysis for each outbreak. In each bootstrap replicate, cities included in the outbreak comparison were sampled with replacement while preserving the original sample size. Pearson correlations between log-transformed observed and predicted first arrival times were then recalculated for all eligible candidate origins, and candidate origins were re-ranked accordingly. We summarised the bootstrap distribution of the difference in Pearson correlation between the first- and second-ranked candidate origins to evaluate whether the highest-ranked candidate cities were meaningfully distinguishable.

**Method L. Robustness to an alternative post-zero-COVID mobility regime**

To examine whether the main findings depended strongly on mobility patterns during China’s “dynamic zero-COVID” period, we conducted an additional robustness analysis using intercity mobility data from 2023, after relaxation of that policy. We first constructed mobility matrices for four observed periods: the Nanjing Delta outbreak, the Shanghai Omicron outbreak, the full year 2021, and the full year 2023. Each matrix summarised the proportion of movement from each origin city to each destination city. Structural similarity between matrices was assessed using Pearson correlation.

We then repeated the epidemic risk assessment using the 2023 mobility matrix, while keeping the same epidemiological parameter settings as in the main analysis. Simulations were performed separately for SARS-CoV-2 Omicron and influenza A, assuming 100 initial infections and a 21-day simulation window. As in the primary analysis, epidemic risk was summarised across prefecture tiers on days 7, 14, and 21, and hierarchical spread was characterised by the proportion of affected destination cities in each tier, conditional on the tier of outbreak origin.

**Method M. Sensitivity analysis**

To assess the robustness of our findings, we repeated the risk assessment simulations under a range of alternative assumptions. First, we varied the initial number of infections from 100 to 1000 and adjusted the reference migration probability of Shanghai ($p_{Sh}$) from 0.5% to 1.5%. Second, we examined alternative values of the shape parameter $k$ in the sigmoid function (Fig U), acknowledging that anchoring mobility to Shanghai may overestimate migration probabilities for smaller cities. We further evaluated the impact of these variations at the prefecture-tier level by computing the tier-aggregated epidemic risk:

$$\begin{aligned} N_{u}^{risk}=\frac{1}{\left| u \right|}\sum_{i\in u} N_{i}^{risk}\#\left( \text{7} \right) \end{aligned}$$

where $u$ denotes a prefecture tier, and $N_{i}^{risk}$​ represents the epidemic risk associated with city $i$. This allowed us to assess whether the relative ordering of transmission risk across tiers remained stable under alternative modelling assumptions.

**Method N. Joint probabilistic uncertainty analysis**

To evaluate the robustness of the model to simultaneous uncertainty in key epidemiological and mobility-related inputs, we performed a joint probabilistic uncertainty analysis using Latin hypercube sampling. This analysis was designed to complement the one-way sensitivity analyses by propagating uncertainty in multiple parameters through the full simulation framework simultaneously.

We jointly varied six parameters that could materially affect transmission dynamics and spatial risk estimates: the mean reproduction number ($\mu_{R}$), the overdispersion parameter of the offspring distribution ($k$), the shape and scale parameters of the gamma-distributed serial interval, the sigmoid scaling parameter used in the migration probability function, and the baseline outbound migration probability for Shanghai. The sampling ranges were specified as follows: $\mu_{R}$, 1 to 5; $k$, 0.10–10; serial interval shape, 1 to 5.0; serial interval scale, 0.10 to 2.0; sigmoid scaling parameter, 0.2 to 2.0; and baseline Shanghai migration probability, 0.005 to 0.015.

We generated 28 parameter combinations using Latin hypercube sampling to obtain broad coverage of the multidimensional parameter space. For each sampled parameter set, we reran the transmission simulation and repeated the downstream risk assessment using the same analytical framework as in the main analysis. For comparability with the main text, we summarised the resulting population at risk across prefecture tiers under the same risk definitions and time windows used in Figure 4.

The complete set of sampled parameter combinations is provided in Table D. Pairwise distributions of the sampled values are shown in Fig W, which illustrates the spread of sampled points across the prespecified ranges. Risk estimates across the 28 sampled parameter sets are shown in Supplementary Figs X–Z for the 7-day, 14-day, and 21-day thresholds, respectively.

**Method O. Alternative definitions of** $\mathbf{N}_{\mathbf{i}}^{\mathbf{risk}}$ **based on importation thresholds**

In the main analysis, $N_{i}^{\mathrm{risk}}$ was used to summarise the population living in cities considered at risk under the simulated spatial spread process. To assess the sensitivity of this metric to the treatment of risk as an introduction-based trigger, we conducted additional analyses using stricter importation thresholds for both SARS-CoV-2 (Omicron) and influenza A. Specifically, instead of including a destination city’s population in $N_{i}^{\mathrm{risk}}$ once that city had received at least one imported infection, we redefined $N_{i}^{\mathrm{risk}}$ by including a city only when the cumulative number of imported infections reached at least 5, 10, 50, or 100 cases.

The rationale for this analysis was that, when sustained community transmission is of interest, the scope of transmission within a destination city may be less plausibly represented by a single imported infection and may be more meaningfully related to whether that city receives a sufficient number of introductions, together with its population size. Because the number of imported infections received by destination cities also depends on epidemic size in the origin city, we repeated these analyses under three alternative initial numbers of infections in the origin city (100, 500, and 1000 cases), yielding 12 scenario combinations for each pathogen. For each scenario, we recalculated $N_{i}^{\mathrm{risk}}$ at 7, 14, and 21 days and compared the resulting patterns across prefecture tiers.

**Method P. Permutation-based inference for tier-level comparisons**

Differences in the risk metric across prefecture tiers were originally assessed using the Kruskal–Wallis test, with city-level values treated as the unit of analysis. For each pathogen and time point, city-level values of $N_{i}^{\mathrm{risk}}$ were grouped by prefecture tier and compared using this non-parametric rank-based test.

Because cities are connected through the mobility network and therefore may not be strictly independent observational units, we additionally performed permutation-based analyses to assess the robustness of the cross-tier comparisons. For each pathogen and time point, we first calculated the observed Kruskal–Wallis statistic for the city-level values of $N_{i}^{\mathrm{risk}}$across prefecture tiers. We then randomly permuted prefecture-tier labels across cities while preserving the number of cities in each tier, and recalculated the Kruskal–Wallis statistic for each permuted dataset to generate an empirical null distribution. The permutation-based p-value was calculated as the proportion of permuted Kruskal–Wallis statistics greater than or equal to the observed statistic. This analysis was used as a robustness check to evaluate whether the observed tier gradient was stronger than expected under random reassignment of tier labels, without relying solely on the conventional asymptotic p-value from the Kruskal–Wallis test.

**Method Q. Exploratory post-arrival local transmission analysis**

To examine how factors not explicitly included in the arrival time analysis could affect epidemic dynamics after introduction, we conducted an exploratory post-arrival local transmission sensitivity analysis. We retained the original intercity diffusion framework to determine the first arrival time in each city. Following first arrival, we simulated local transmission in each city using a simplified susceptible–infectious–removed (SIR) model.

For city 𝑖, post-arrival local transmission was described by

$$\begin{aligned} \frac{dS_{i}}{\mathrm{dt}}=-\beta_{i}\frac{S_{i}I_{i}}{N_{i}}, \frac{dI_{i}}{\mathrm{dt}}=\beta_{i}\frac{S_{i}I_{i}}{N_{i}}-\gamma I_{i}, \frac{dR_{i}}{\mathrm{dt}}=\gamma I_{i},\#\left( 8 \right) \end{aligned}$$

Where $N_{i}$ is the population of city $i$, $\gamma$ is the recovery rate, and $\beta_{i}$ is the city-specific transmission rate after introduction. At the time of first arrival, one infectious individual was introduced into city $i$, such that $S_{i}\left( t_{arr,i} \right)=N_{i}-1$, $I_{i}\left( t_{arr,i} \right)=1$, $R_{i}\left( t_{arr,i} \right)=0$.

The city-specific transmission rate was defined as

$$\begin{aligned} \beta_{i}=\beta_{0}\times g_{\mathrm{int}}\times g_{\mathrm{imm}}\times g_{\mathrm{clim}}\left( i \right)\times g_{\mathrm{tier}}\left( i \right),\#\left( 9 \right) \end{aligned}$$

where $\beta_{0}$ is the baseline transmission rate, $g_{\mathrm{int}}$ is an intervention modifier shared across all cities, $g_{\mathrm{imm}}$ is an immunity modifier shared across all cities, $g_{\mathrm{clim}}\left( i \right)$ is a climate modifier based on city-level annual mean temperature and annual mean relative humidity, and $g_{\mathrm{tier}}\left( i \right)$ is a modifier associated with prefecture tier.

We considered three intervention scenarios representing high, moderate, and low control intensity after introduction, with $g_{\mathrm{int}}=$0.6, 0.8, and 1.0, respectively. We also considered three immunity scenarios representing high, moderate, and low pre-existing protection, with $g_{\mathrm{imm}}=$0.6, 0.8, and 1.0, respectively. Prefecture tier was included as a categorical modifier and used as a pragmatic proxy for urban–rural and metropolitan differences in contact opportunities, density, and built environment. Specifically, g_tier (i) was set to 1.15, 1.10, 1.05, 1.00, 0.95, and 0.90 for super-tier, tier-1, tier-2, tier-3, tier-4, and tier-5 cities, respectively.

To represent climatic heterogeneity, we defined

$$\begin{aligned} g_{\mathrm{clim}}\left( i \right)=\exp\left( \eta_{T}Z_{i}^{\mathrm{Temp}}+\eta_{H}Z_{i}^{\mathrm{RH}} \right),\#\left( 10 \right) \end{aligned}$$

where $Z_{i}^{\mathrm{Temp}}$ and $Z_{i}^{\mathrm{RH}}$ are the standardized annual mean temperature and annual mean relative humidity for city$i$. In the main analysis, we set $\eta_{T}=\eta_{H}=-0.08$, such that cooler and drier cities were assigned modestly higher local transmission potential. These coefficients were specified to impose structured climatic heterogeneity rather than to estimate direct causal meteorological effects.

For Omicron, we set the recovery rate to $\gamma=0.194 \mathrm{day}^{-1}$, corresponding to an average infectious period of 5.16 days[3], and set the baseline transmission rate to $\beta_{0}=0.756 \mathrm{day}^{-1}$, derived from a baseline reproduction number of 3.9[4]. These parameter values were informed by published estimates for Omicron BA.2.

For each city and scenario combination, we summarised three post-arrival outcomes: peak size, defined as the maximum daily incidence during the simulation period; and cumulative incidence over the simulation period. These scenario values were chosen to represent plausible contrasts in post-introduction transmission conditions rather than to reproduce observed city-specific immunity or intervention intensity.

**Supplementary Results**

**Result A. Uncertainty analyses for origin identification**

Uncertainty in the Pearson correlation estimates for candidate origins was quantified using permutation p-values and bootstrap 95% confidence intervals (Table A).

For the Shanghai outbreak, the top-ranked origin city was Shanghai, with an observed maximum Pearson correlation of 0.66 and a max-statistic permutation p-value of 0.087 (Fig G). In the bootstrap analysis, Shanghai ranked first in 46.5% of replicates, ranked within the top three in 65.4% of replicates, and had a median bootstrap rank of 2. The observed difference in Pearson correlation between the first- and second-ranked candidate origins was 0.062, with a bootstrap 95% confidence interval of [-0.29,0.32].

For the Nanjing outbreak, the top-ranked origin city was Nanjing, with an observed maximum Pearson correlation of 0.86 and a max-statistic permutation p-value of 0.039 (Fig G). In the bootstrap analysis, Nanjing ranked first in 68% of replicates, ranked within the top three in 97% of replicates, and had a median bootstrap rank of 1. The observed difference in Pearson correlation between the first- and second-ranked candidate origins was 0.27, with a bootstrap 95% confidence interval of [-0.27,0.53].

From the correlation-based ranking, Shanghai and Nanjing emerged as the most likely outbreak origins, although the differences between the top-ranked candidate cities were not always statistically distinguishable.

**Result B. Robustness of tier-specific diffusion patterns under 2023 mobility conditions**

Intercity mobility matrices were highly similar across the four observed periods (Fig H). Pairwise comparisons showed Pearson correlations of 0.930 to 0.995, indicating that the broad organisation of intercity mobility remained stable across these settings.

When the risk assessment was repeated using the 2023 mobility matrix, the main qualitative patterns were preserved (Fig K-M). For Omicron, epidemic risk remained highest in super-tier and tier-1 cities, with broader cross-tier dissemination over time. For influenza A, spread remained more stratified, with less extensive penetration across the urban hierarchy. These differences between pathogens were evident on days 7, 14, and 21.

Taken together, these analyses suggest that the principal qualitative conclusions of the study were robust to an alternative post-zero-COVID mobility regime, although the absolute magnitude of epidemic risk remained context dependent.

**Result C. Validation against cumulative affected cities and temporal growth of spatial spread**

We compared the observed and simulated cumulative number of affected cities within 30 days of the Shanghai outbreak. There were 65 cities observed to have been affected, while our model predicted a mean cumulative total of 60.4 affected cities at day 30 (95% simulation interval [21.0,99.0]). The temporal growth trajectories of cumulative affected cities are shown in Fig C: the simulated fitted curve closely tracks the observed fitted curve across the 30-day window, with both trajectories showing an initial phase of rapid spatial expansion followed by deceleration as the pool of reachable cities within the mobility network becomes increasingly saturated. To formally compare the temporal growth patterns, we computed the mean cumulative number of affected cities across all 30 days for the observed data (23.77) and for each of the 10,000 simulation replicates. The mean of simulated means was 28.90 (95% simulation interval [10.10,53.03]), and the observed mean fell at the 36th percentile of the simulated distribution. A non-parametric comparison found no statistically significant difference between the observed and simulated temporal growth distributions (two-sided p = 0.72), indicating that the model-predicted trajectory of spatial expansion is consistent with the observed data.

**Result D. Arrival time distributions**

In the Shanghai outbreak, the distribution of simulated first arrival times across cities was broadly consistent with observed values (Fig D). However, simulations tended to produce slightly earlier and less variable arrival times. The observed mean arrival time was 23.25 days, with a median of 26.00 days and an interquartile range of 14.00 days. Corresponding simulated values were a mean of 21.80 days, a median of 24.82 days, and an interquartile range of 9.16 days. The overall shape of the simulated distribution aligned with the observed distribution, indicating that the model captures the central tendency of spatial spread but somewhat underestimates dispersion across cities.

**Result E. Mixed-effects model**

In the mixed-effects model, several structural determinants were strongly associated with arrival time. Higher population flow from the origin city was associated with shorter arrival times ($\beta$ = −0.059, 95% CI [−0.065,−0.053]), while greater geodesic distance was associated with longer arrival times ($\beta$ = 0.419, 95% CI [0.412,0.427]). Cities with zero recorded population flow experienced delayed arrival ($\beta$ = 0.077, 95% CI [0.067,0.087]). Larger population size was associated with earlier arrival ($\beta$ = −0.125, 95% CI [−0.193,−0.057]).

**Result F. Cross-tier comparisons under alternative baseline migration probabilities for Shanghai**

We further examined how cross-tier comparisons changed when the baseline daily migration probability for Shanghai was varied from 0.5% to 1.5% (Fig V; Table B). Increasing this baseline value increased the estimated population at risk across all prefecture tiers. In absolute terms, at 7 days, the mean population at risk in super-tier cities increased from 32.3 million to 49.2 million, whereas that in tier-5 cities increased from 1.9 million to 2.3 million; the corresponding absolute difference between super-tier and tier-5 cities increased from 30.4 million to 46.9 million. At 14 days, the super-tier mean increased from 53.2 million to 82.8 million and the tier-5 mean from 2.6 million to 4.1 million, with the absolute difference increasing from 50.6 million to 78.7 million. At 21 days, the super-tier mean increased from 78.9 million to 113.4 million and the tier-5 mean from 4.0 million to 7.6 million, with the absolute difference increasing from 74.8 million to 105.8 million. In relative terms, the cross-tier gradient remained similar across the tested range: the ratio of mean population at risk in super-tier versus tier-5 cities ranged from 16.9 to 21.2 at 7 days, 20.0 to 22.0 at 14 days, and 15.0 to 20.1 at 21 days; the corresponding ratio for tier-1 versus tier-5 cities ranged from 9.8 to 11.9, 11.9 to 13.1, and 10.3 to 12.1, respectively. These results indicate that varying the Shanghai baseline migration probability changed the numerical magnitude of the estimated risk, but did not materially alter the main cross-tier comparison pattern.

**Result G. Sensitivity of** $\mathbf{N}_{\mathbf{i}}^{\mathbf{risk}}$ **to alternative importation thresholds and initial seeding sizes**

For SARS-CoV-2 (Omicron), using stricter importation thresholds reduced the estimated magnitude of $N_{i}^{\mathrm{risk}}$, particularly at earlier time points and when the initial number of infections in the origin city was small. Under thresholds of 5 and 10 imported cases (Fig P, Fig Q), $N_{i}^{\mathrm{risk}}$ still increased markedly with larger initial seeding sizes, and the tier gradient remained clearly visible across 7, 14, and 21 days. Under stricter thresholds of 50 and 100 imported cases (Fig R, Fig S), the reduction in $N_{i}^{\mathrm{risk}}$ became more pronounced, especially for lower-tier cities and at 7 days, indicating that these cities were less likely to accumulate large numbers of imported infections early in the spread process. Even under these stricter definitions, however, super-tier and tier-1 cities continued to account for the largest at-risk populations, while lower-tier cities remained substantially lower.

Influenza A showed the same qualitative pattern (Figs EE-HH). Increasing the importation threshold again reduced the magnitude of $N_{i}^{\mathrm{risk}}$, with the strongest attenuation under the 50- and 100-case thresholds. Under the strictest threshold of 100 imported cases, $N_{i}^{\mathrm{risk}}$ changed little across initial seeding sizes for most tiers within the examined time windows, indicating that relatively few cities accumulated that many imported infections. Nonetheless, the hierarchical ordering across prefecture tiers remained stable: super-tier cities consistently had the largest $N_{i}^{\mathrm{risk}}$, followed by tier-1 cities, whereas lower-tier cities remained substantially lower.

Overall, the absolute value of $N_{i}^{\mathrm{risk}}$ depended on both the importation threshold and the initial number of infections in the origin city: larger thresholds reduced $N_{i}^{\mathrm{risk}}$, whereas larger origin seeding sizes generally increased it. However, the main cross-tier comparison pattern remained qualitatively unchanged for both pathogens across all tested scenarios, indicating that the principal conclusions were not dependent on defining a city as at risk after only a single imported infection.

**Result H. Permutation-based assessment of cross-tier differences in** $\mathbf{N}_{\mathbf{i}}^{\mathbf{risk}}$

Permutation-based analyses supported the original Kruskal-Wallis comparisons of $N_{i}^{\mathrm{risk}}$ across prefecture tiers (Table C). For SARS-CoV-2 (Omicron), the observed Kruskal-Wallis statistics were 142.58 at 7 days, 175.50 at 14 days, and 169.51 at 21 days, with permutation-based p<0.001 for all three time points. The corresponding Kruskal-Wallis p-values were 5.06×10^−29^, 4.90×10^−36^, and 9.29×10^−35^, respectively. For influenza A, the observed Kruskal-Wallis statistics were 198.32, 159.55, and 133.52 at 7, 14, and 21 days, respectively, and the permutation-based p-values were again <0.001 at all three time points; the corresponding Kruskal-Wallis p-values were 6.51×10^−41^, 1.23×10^−32^, and 4.26×10^−27^. These results indicate that the observed hierarchical differences in $N_{i}^{\mathrm{risk}}$ across prefecture tiers were substantially stronger than expected under random reassignment of tier labels.

Taken together, these analyses indicate that the observed hierarchical differences in $N_{i}^{\mathrm{risk}}$ across prefecture tiers are robust to the choice of statistical framework and do not rely solely on independence assumptions. Although cities in the mobility network are not strictly independent, the cross-tier gradient in $N_{i}^{\mathrm{risk}}$ remained strongly supported when assessed against an empirical null distribution based on permuted tier labels.

**Result I. Robustness of tier-specific risk patterns under joint parameter uncertainty**

The joint probabilistic uncertainty analysis showed that the main conclusions were robust to simultaneous variation in the reproduction number distribution, serial interval distribution, and migration probability parameters. The 28 Latin hypercube parameter sets provided broad coverage of the prespecified uncertainty space, without obvious clustering in pairwise projections of sampled values (Fig W).

Across the 28 sampled parameter sets, uncertainty in model inputs changed the absolute magnitude and spread of the estimated population at risk, but the broad tier-specific structure of the results remained similar to that in the baseline analysis. In particular, the contrast between higher-tier and lower-tier cities remained apparent across simulations (Figs X-Z).

These findings indicate that the central inference of the study is not dependent on a single fixed choice of epidemiological or migration parameters. Rather, the association between urban hierarchy and spatial transmission risk remained stable under joint uncertainty in the major model inputs considered here.

**Result J. Exploratory post-arrival local transmission analysis**

In the exploratory post-arrival local transmission analysis, introducing intervention, immunity, climate, and tier modifiers had a limited effect on the interpretation of first arrival timing but a much larger effect on post-arrival epidemic outcomes. Across scenarios, peak size and cumulative incidence varied substantially with the assumed levels of control and pre-existing immunity, and with climatic conditions and prefecture tier, further modulating these differences (Figs AA-BB).

In general, stronger interventions and higher pre-existing immunity were associated with lower peak size and cumulative incidence across scenarios. Climatic conditions and prefecture tier further modified the magnitude of local outbreaks. Under several scenario combinations, the ranking of prefecture tiers based on post-arrival outcomes differed from the ranking implied by the early diffusion analysis alone, indicating that local susceptibility, intervention intensity, and environmental suitability can become increasingly important once transmission is established.

These findings support the rationale for using first arrival timing as the primary outcome in the main analysis: arrival time-based metrics are comparatively robust for studying intercity diffusion, whereas post-arrival outcomes such as peak size and cumulative incidence are more sensitive to local epidemiological and environmental heterogeneity.

**Supplementary Figures**

**Fig A. Extended validation of first arrival time predictions incorporating pathogen-specific reproduction numbers and serial intervals.**

(A) Comparison between observed and mean predicted first arrival times for cities affected by the Shanghai outbreak, based on simulations incorporating outbreak-specific reproduction number and serial interval values. (B) Comparison between observed and mean predicted first arrival times for cities affected by the Nanjing outbreak, based on simulations incorporating outbreak-specific reproduction number and serial interval values. Each dot represents a city, and symbol size reflects its population. The dashed line indicates the 1:1 line. For the Shanghai outbreak, the Pearson correlation was 0.66 (p<0.001), with a mean absolute error (MAE) of 6.28 days and a root mean squared error (RMSE) of 7.99 days. For the Nanjing outbreak, the corresponding values were 0.86 (p<0.001), 1.93 days, and 2.56 days, respectively.

**

**Fig B. Origin identification after incorporating pathogen-specific transmission parameters.**

Correlations between observed and predicted first arrival times, estimated from simulations incorporating pathogen-specific transmission parameters (reproduction number and serial interval), were compared with correlations between observed arrival times and geodesic distance for the outbreaks in Shanghai (a), Nanjing (b), and northwestern China (c). Each dot represents a candidate city, and symbol size corresponds to the resident population. Dashed lines indicate thresholds for the top 10% of absolute correlation coefficients based on predicted arrival times (orange) or geodesic distance (blue). Shaded areas highlight cities within these top 10% thresholds. Shanghai and Nanjing remained strongly supported as the outbreak origins, and Xi’an remained the most probable origin of the northwestern China outbreak. Overall, the model-data correlation-based approach provided more reliable origin identification than the distance-based method after incorporating pathogen-specific transmission parameters.

**Fig C. Cumulative number of cities affected by the Shanghai outbreak over the 30-day observation window.**

The blue bars represent the observed daily cumulative count of affected cities, and the blue fitted curve shows observed growth trajectory. The red bars represent the mean simulated cumulative count of affected cities across 10,000 stochastic replicates at each day, and the red fitted curve shows the simulated growth trajectory. A city was counted as affected on the day it first received at least one imported infection in the simulation, and was included in the analysis if it met the 5% simulation-frequency inclusion threshold. The close agreement between the observed and simulated fitted curves across the observation period, together with the non-parametric comparison (two-sided p = 0.72), indicates that the model reproduces the temporal trajectory of spatial expansion without systematic bias.

**Fig D. Distribution of observed and simulated first arrival times in the Shanghai outbreak.**

Density distributions of first arrival times across cities for the Shanghai outbreak. The observed distribution is derived from reported city-level first-detection dates, whereas the simulated distribution is aggregated from repeated stochastic simulations. The simulated distribution shows a similar central tendency to the observed data, with slightly earlier and less dispersed arrival times. Summary statistics are as follows: observed mean 23.25 days, median 26.00 days, interquartile range 14.00 days; simulated mean 21.80 days, median 24.82 days, interquartile range 9.16 days.

**Fig E. Scatter plot of observed first arrival time and geodesic distance from Shanghai and Nanjing outbreaks.**

Scatter plots of observed first arrival time against geodesic distance from the outbreak origin for cities affected by the Shanghai outbreak (upper panel, blue) and the Nanjing outbreak (lower panel, red). Each point represents one affected city.

**Fig F.** **Kernel density estimates of correlation coefficients using predicted arrival times and geodesic distance.**

Kernel probability density estimates of correlation coefficients between observed first arrival times and either predicted arrival times (orange) or geodesic distance (blue) for the outbreaks in Shanghai (A), Nanjing (B), and northwestern China (C). Dashed vertical lines indicate the thresholds corresponding to the top 10% of absolute correlation coefficients based on predicted arrival times (orange) and geodesic distance (blue). Shaded regions denote the ranges of these top 10% absolute correlation values for each method. Overall, predicted arrival time correlations exhibit stronger, more distinct peaks than those from the distance-based approach, illustrating the superior discriminatory ability of the mobility-driven framework.

**
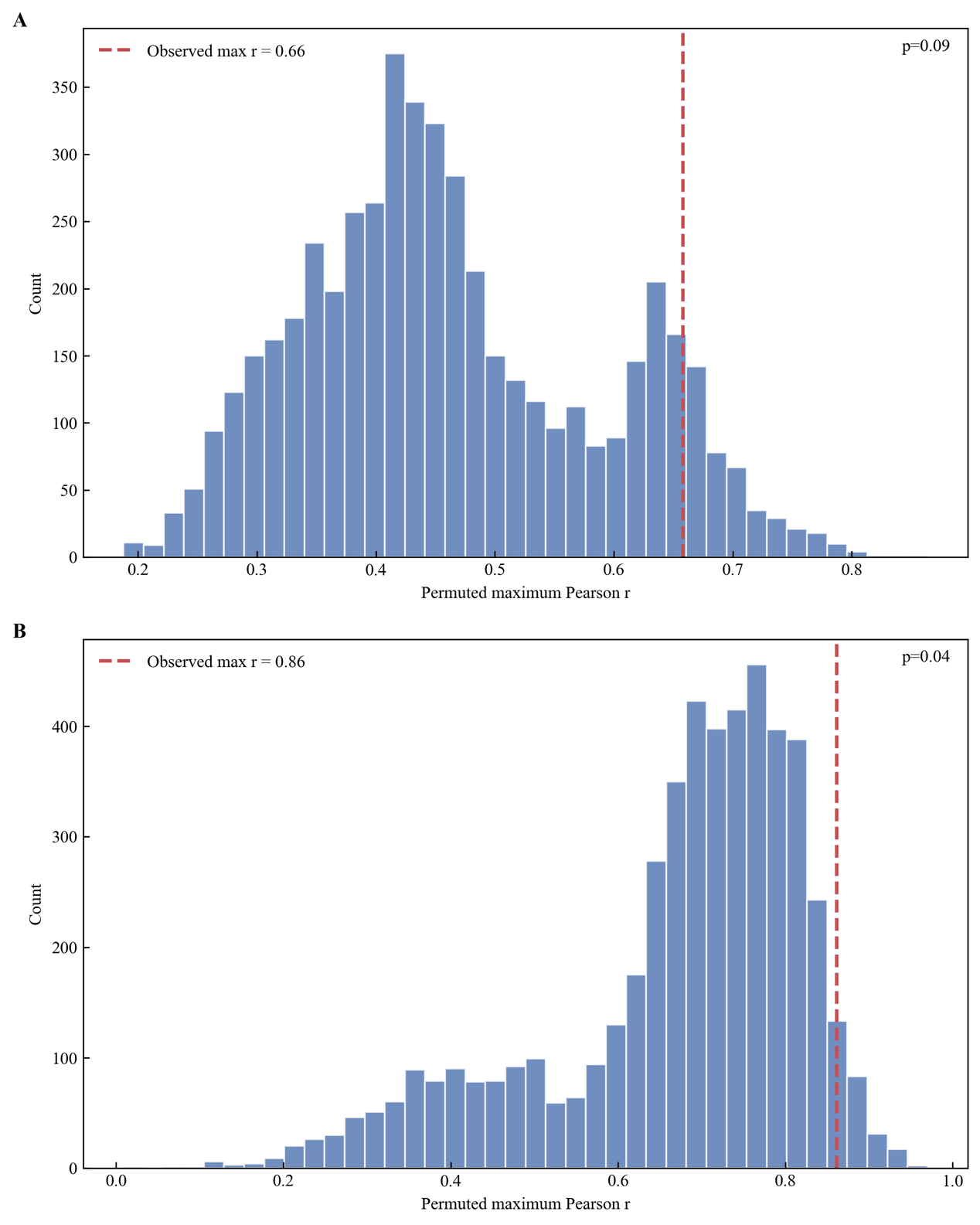
**

**Fig G. Max-statistic permutation test for origin identification in the Shanghai and Nanjing outbreaks.**

Null distributions of the maximum Pearson correlation across all candidate origins obtained from the max-statistic permutation test for the Shanghai outbreak (A) and the Nanjing outbreak (B). The vertical dashed line indicates the observed maximum correlation in each outbreak.

**Fig H. Intercity mobility matrices across four periods.**

(A-D) Heatmaps show the intercity mobility matrices for the Nanjing Delta outbreak, the Shanghai Omicron outbreak, Year 2021, and Year 2023. Each cell represents the proportion of movement from an origin city to a destination city, with darker colours indicating higher mobility intensity. Pairwise comparisons showed high similarity across matrices, with Pearson correlations ranging from 0.930 to 0.995.

**Fig I.** **Epidemic risk across prefecture tiers for SARS-CoV-2 and Influenza A transmission within 7 days.**

Raincloud plots show the distribution of epidemic risk across prefecture tiers for SARS-CoV-2 (Omicron) (A) and Influenza A (B), assuming 100 initial cases and a 7-day simulation period. Significant differences in risk were observed across tiers for both pathogens (Kruskal–Wallis test, p < 0.001). Panels (C) and (D) present heatmaps of the proportion of cities affected in each destination tier, conditional on outbreaks originating from cities in a given source tier. Darker colours indicate higher proportions, illustrating tier-specific patterns of within-tier and cross-tier transmission.

**Fig J.** **Epidemic risk across prefecture tiers for SARS-CoV-2 and Influenza A transmission within 21 days.**

(A–B) Raincloud plots show the distribution of epidemic risk across prefecture tiers for SARS-CoV-2 (Omicron) and Influenza A, assuming 100 initial cases and a 21-day simulation window. Risk differences across tiers were significant for both pathogens (Kruskal–Wallis test, p < 0.001). (C–D) Heatmaps depict the proportion of affected cities in each destination tier, conditional on outbreaks originating from source tiers. Darker colours indicate a higher likelihood of transmission, illustrating tier-specific patterns of within-tier and cross-tier spread.

**Fig K. Epidemic risk across prefecture tiers for SARS-CoV-2 and Influenza A transmission on Day 7, based on 2023 population mobility data.**

(A-B) Raincloud plots show the distribution of epidemic risk across prefecture tiers for SARS-CoV-2 (Omicron) and Influenza A on Day 7, assuming 100 initial cases and a 21-day simulation window. (C-D) Heatmaps depict the proportion of affected cities in each destination tier, conditional on outbreaks originating from source tiers, for SARS-CoV-2 (Omicron) and Influenza A on Day 7. Darker colours indicate a higher likelihood of transmission, illustrating tier-specific patterns of within-tier and cross-tier spread.

**Fig L. Epidemic risk across prefecture tiers for SARS-CoV-2 and Influenza A transmission on Day 14, based on 2023 population mobility data.**

(A-B) Raincloud plots show the distribution of epidemic risk across prefecture tiers for SARS-CoV-2 (Omicron) and Influenza A on Day 14, assuming 100 initial cases and a 21-day simulation window. (C-D) Heatmaps depict the proportion of affected cities in each destination tier, conditional on outbreaks originating from source tiers, for SARS-CoV-2 (Omicron) and Influenza A on Day 14. Darker colours indicate a higher likelihood of transmission, illustrating tier-specific patterns of within-tier and cross-tier spread.

**Fig M. Epidemic risk across prefecture tiers for SARS-CoV-2 and Influenza A transmission on Day 21, based on 2023 population mobility data.**

(A-B) Raincloud plots show the distribution of epidemic risk across prefecture tiers for SARS-CoV-2 (Omicron) and Influenza A on Day 21, assuming 100 initial cases and a 21-day simulation window. (C-D) Heatmaps depict the proportion of affected cities in each destination tier, conditional on outbreaks originating from source tiers, for SARS-CoV-2 (Omicron) and Influenza A on Day 21. Darker colours indicate a higher likelihood of transmission, illustrating tier-specific patterns of within-tier and cross-tier spread.

**Fig N.** **Sensitivity analysis of initial infection numbers for SARS-CoV-2 (Omicron) transmission using an importation threshold of 1 case.**

The raincloud plots illustrate the distribution of epidemic risk across prefecture tiers for SARS-CoV-2 (Omicron) under three initial infection scenarios (100, 500, and 1000 cases), evaluated at 7, 14, and 21 days after seeding. Higher initial case numbers increase risk across all tiers while preserving the overall hierarchical pattern of risk distribution. The bottom panels summarise the mean epidemic risk for each prefecture tier as a function of the number of initial infections (100-1000), showing consistent tier ordering and demonstrating that increasing the initial seeding size does not alter the relative vulnerability structure across tiers.

**Fig O.** **Sensitivity analysis of initial infection numbers for Influenza A transmission using an importation threshold of 1 case.**

The raincloud plots illustrate the distribution of epidemic risk across prefecture tiers for Influenza A under three initial seeding scenarios (100, 500, and 1000 cases), evaluated at 7, 14, and 21 days after introduction. Increasing the number of initial cases raises overall epidemic risk but preserves the characteristic stratified diffusion pattern observed for Influenza A. The bottom panels summarise the mean epidemic risk for each prefecture tier as a function of the number of initial infections (100-1000), demonstrating consistent tier ordering across time windows and confirming that larger seeding sizes amplify risk magnitude without altering the relative risk hierarchy.

**Fig P. Sensitivity analysis of alternative importation thresholds for** $\mathbf{N}_{\mathbf{i}}^{\mathbf{risk}}$ **for SARS-CoV-2 (Omicron): threshold of 5 imported cases.**

The raincloud plots illustrate the distribution of $N_{i}^{\mathrm{risk}}$ across prefecture tiers for SARS-CoV-2 (Omicron) under three initial infection scenarios (100, 500, and 1000 cases), evaluated at 7, 14, and 21 days after seeding. In this analysis, a destination city’s population was included in $N_{i}^{\mathrm{risk}}$ only when the cumulative number of imported infections into that city reached at least 5 cases. Higher initial case numbers increased $N_{i}^{\mathrm{risk}}$ across all tiers while preserving the overall hierarchical pattern of risk distribution. The bottom panels summarise the mean $N_{i}^{\mathrm{risk}}$ for each prefecture tier as a function of the number of initial infections (100–1000), showing consistent tier ordering and indicating that increasing the initial seeding size did not alter the relative vulnerability structure across tiers.

**

**Fig Q. Sensitivity analysis of alternative importation thresholds for** $\mathbf{N}_{\mathbf{i}}^{\mathbf{risk}}$ **for SARS-CoV-2 (Omicron): threshold of 10 imported cases.**

The raincloud plots illustrate the distribution of $N_{i}^{\mathrm{risk}}$ across prefecture tiers for SARS-CoV-2 (Omicron) under three initial infection scenarios (100, 500, and 1000 cases), evaluated at 7, 14, and 21 days after seeding. In this analysis, a destination city’s population was included in $N_{i}^{\mathrm{risk}}$ only when the cumulative number of imported infections into that city reached at least 10 cases. Higher initial case numbers increased $N_{i}^{\mathrm{risk}}$ across all tiers while preserving the overall hierarchical pattern of risk distribution. The bottom panels summarise the mean $N_{i}^{\mathrm{risk}}$ for each prefecture tier as a function of the number of initial infections (100–1000), showing consistent tier ordering and indicating that increasing the initial seeding size did not alter the relative vulnerability structure across tiers.

**Fig R. Sensitivity analysis of alternative importation thresholds for** $\mathbf{N}_{\mathbf{i}}^{\mathbf{risk}}$ **for SARS-CoV-2 (Omicron): threshold of 50 imported cases.**

The raincloud plots illustrate the distribution of $N_{i}^{\mathrm{risk}}$ across prefecture tiers for SARS-CoV-2 (Omicron) under three initial infection scenarios (100, 500, and 1000 cases), evaluated at 7, 14, and 21 days after seeding. In this analysis, a destination city’s population was included in $N_{i}^{\mathrm{risk}}$ only when the cumulative number of imported infections into that city reached at least 50 cases. Compared with lower thresholds, $N_{i}^{\mathrm{risk}}$ was reduced in absolute magnitude, particularly in lower-tier cities and at earlier time points, while the overall hierarchical pattern across prefecture tiers remained evident. The bottom panels summarise the mean $N_{i}^{\mathrm{risk}}$ for each prefecture tier as a function of the number of initial infections (100–1000), showing that larger initial seeding sizes increased $N_{i}^{\mathrm{risk}}$ without materially changing the relative tier ordering.

**Fig S. Sensitivity analysis of alternative importation thresholds for** $\mathbf{N}_{\mathbf{i}}^{\mathbf{risk}}$ **for SARS-CoV-2 (Omicron): threshold of 100 imported cases.**

The raincloud plots illustrate the distribution of $N_{i}^{\mathrm{risk}}$ across prefecture tiers for SARS-CoV-2 (Omicron) under three initial infection scenarios (100, 500, and 1000 cases), evaluated at 7, 14, and 21 days after seeding. In this analysis, a destination city’s population was included in $N_{i}^{\mathrm{risk}}$ only when the cumulative number of imported infections into that city reached at least 100 cases. This stricter definition further reduced the absolute magnitude of $N_{i}^{\mathrm{risk}}$, especially for lower-tier cities and in the early phase after seeding, but the overall cross-tier gradient remained qualitatively similar. The bottom panels summarise the mean $N_{i}^{\mathrm{risk}}$ for each prefecture tier as a function of the number of initial infections (100–1000), indicating that higher initial seeding sizes increased $N_{i}^{\mathrm{risk}}$ while preserving the overall hierarchical pattern of risk distribution.

**Fig T.** **Sensitivity analysis of reference migration probability (**$\boldsymbol{p}_{\boldsymbol{Sh}}$**) for Influenza A transmission.**

The raincloud plots illustrate epidemic risk across prefecture tiers under three reference mobility settings ($p_{Sh}$ = 0.5%, 0.9%, and 1.3%), evaluated at 7, 14, and 21 days. Although increasing $p_{Sh}$ elevates overall epidemic risk, the characteristic tier-stratified diffusion pattern of Influenza A remains stable across all mobility assumptions. The bottom panels summarise the mean epidemic risk for each prefecture tier across a broader range of $p_{Sh}$ values (0.5%-1.5%), showing gradual scaling of risk magnitude while preserving the relative ordering between tiers.

**Fig U.** **Sigmoid migration function under different values of** $\boldsymbol{k}$**.**

Larger $k$ values produce sharper transitions between low and high migration probabilities, whereas smaller $k$ values yield more gradual changes. This sensitivity analysis evaluates how assumptions about the responsiveness of migration probability to the migration scale index may influence simulated mobility-driven transmission patterns.

**Fig V.** **Sensitivity analysis of the sigmoid migration function (**$\boldsymbol{k}$**) for Influenza A transmission.**

The raincloud plots show the epidemic risk in cities across each prefecture tier under Influenza A transmission, with sigmoid steepness parameters $k$ = 0.6, 1.2, and 1.8. The bottom three panels present the mean epidemic risk for each prefecture tier across a broader range of $k$ values (0.2–2.0) over 7, 14, and 21 days. Higher $k$ values yield steeper sigmoid transitions, leading to lower inferred mobility probabilities for smaller cities. This analysis evaluates how assumptions about the responsiveness of migration probability to the migration scale index affect tier-based transmission risk.

**

**Fig W. Latin hypercube samples for the joint probabilistic uncertainty analysis.**

Pairwise scatter matrix showing the 28 Latin hypercube-sampled parameter sets used in the joint uncertainty analysis. Parameters jointly sampled were the mean reproduction number ($\mu_{R}$), overdispersion parameter ($k$), serial interval shape, serial interval scale, sigmoid scaling parameter for migration probability, and baseline outbound migration probability for Shanghai. Histograms on the diagonal show the marginal distribution of sampled values for each parameter, and off-diagonal panels show pairwise projections of the sampled combinations. Together, these plots illustrate broad coverage of the prespecified multidimensional parameter space.

**

**Fig X. Population at risk across prefecture tiers under joint parameter uncertainty: 7-day threshold.**

Raincloud plots showing the estimated population at risk across prefecture tiers for each of the 28 Latin hypercube-sampled parameter sets when risk was defined using the 7-day threshold. Each numbered panel corresponds to one sampled parameter set listed in Table D. The figure is presented using the same analytical framework in the main text to facilitate direct comparison with the baseline analysis. Across parameter sets, the absolute magnitude of risk varied, whereas the broad tier-structured spatial pattern remained similar.

**

**Fig Y. Population at risk across prefecture tiers under joint parameter uncertainty: 14-day threshold.**

Raincloud plots showing the estimated population at risk across prefecture tiers for each of the 28 Latin hypercube-sampled parameter sets when risk was defined using the 14-day threshold. Each numbered panel corresponds to one sampled parameter set listed in Table D. The overall contrast between higher-tier and lower-tier cities remained apparent across sampled parameter sets despite variation in absolute risk estimates.

**

**Fig Z. Population at risk across prefecture tiers under joint parameter uncertainty: 21-day threshold.**

Raincloud plots showing the estimated population at risk across prefecture tiers for each of the 28 Latin hypercube-sampled parameter sets when risk was defined using the 21-day threshold. Each numbered panel corresponds to one sampled parameter set listed in Table D. Although uncertainty in the jointly sampled parameters affected the magnitude of estimated risk, the overall tier-specific pattern remained broadly consistent with that in the baseline analysis.

**

**Fig AA. Exploratory post-arrival local transmission analysis of peak daily incidence under alternative intervention, immunity, climate, and prefecture-tier scenarios.**

For each city, the first arrival time was determined using the main intercity diffusion model, after which local transmission was simulated using a simplified city-level SIR model. Panels show variation in peak daily incidence across combinations of three intervention scenarios ($g_{int}$=0.6,0.8,1.0), three immunity scenarios ($g_{imm}$=0.6,0.8,1.0), climatic modulation based on standardized temperature and relative humidity, and prefecture-tier modifiers. This analysis was intended to illustrate how factors that were not explicitly included in the arrival time framework could influence post-introduction epidemic trajectories, rather than to reconstruct observed city-specific epidemic burden.

**

**Fig BB. Exploratory post-arrival local transmission analysis of cumulative incidence under alternative intervention, immunity, climate, and prefecture-tier scenarios.**

For each city, the first arrival time was determined using the main intercity diffusion model, after which local transmission was simulated using a simplified city-level SIR model. Panels show variation in cumulative incidence across combinations of three intervention scenarios ($g_{int}$=0.6,0.8,1.0), three immunity scenarios ($g_{int}$=0.6,0.8,1.0), climatic modulation based on standardized temperature and relative humidity, and prefecture-tier modifiers. This analysis was intended to illustrate how factors that were not explicitly included in the arrival time framework could influence post-introduction epidemic trajectories, rather than to reconstruct observed city-specific epidemic burden.

**Fig CC.** **Prefecture-tier classification across mainland China.**

Cities were grouped into hierarchical tiers following the classification system developed by Yicai Media Group, which evaluates five dimensions: concentration of commercial resources, transportation hub capacity, urban residential activity, lifestyle diversity, and long-term development potential. The super-tier consists of four major cities: Beijing, Shanghai, Guangzhou, and Shenzhen. The remaining cities are categorised into Tier-1 (15 cities), Tier-2 (30 cities), Tier-3 (70 cities), Tier-4 (90 cities), and Tier-5 (128 cities). The maps illustrate the spatial distribution of cities in each tier. The basemap shapefiles were downloaded from the Chinese Resource and Environmental Science Data Platform (http://www.resdc.cn/, DOI:10.12078/2023010102).

**Fig DD.** **Distribution of population size across prefecture tiers.**

The raincloud plots show the distribution of permanent resident populations for cities in each prefecture tier. Transparent gradients in population size are observed across tiers, with statistically significant differences between groups (Kruskal–Wallis test, p < 0.001).

**Fig EE. Sensitivity analysis of alternative importation thresholds for** $\mathbf{N}_{\mathbf{i}}^{\mathbf{risk}}$ **for influenza A: threshold of 5 imported cases.**

The raincloud plots illustrate the distribution of $N_{i}^{\mathrm{risk}}$ across prefecture tiers for influenza A when a city’s population was included only after the cumulative number of imported infections reached 5 cases. Results are shown under three initial infection scenarios in the origin city (100, 500, and 1000 cases), evaluated at 7, 14, and 21 days after seeding. The bottom panels summarise the mean $N_{i}^{\mathrm{risk}}$ for each prefecture tier as a function of the number of initial infections (100–-1000). Increasing the initial seeding size increased $N_{i}^{\mathrm{risk}}$ across tiers while preserving the overall hierarchical pattern.

**Fig FF. Sensitivity analysis of alternative importation thresholds for** $\mathbf{N}_{\mathbf{i}}^{\mathbf{risk}}$ **for influenza A: threshold of 10 imported cases.**

The raincloud plots illustrate the distribution of $N_{i}^{\mathrm{risk}}$ across prefecture tiers for influenza A when a city’s population was included only after the cumulative number of imported infections reached 10 cases. Results are shown under three initial infection scenarios in the origin city (100, 500, and 1000 cases), evaluated at 7, 14, and 21 days after seeding. The bottom panels summarise the mean $N_{i}^{\mathrm{risk}}$ for each prefecture tier as a function of the number of initial infections (100–-1000). The stricter threshold reduced the absolute magnitude of $N_{i}^{\mathrm{risk}}$, but the tier ordering remained stable.

**Fig GG. Sensitivity analysis of alternative importation thresholds for** $\mathbf{N}_{\mathbf{i}}^{\mathbf{risk}}$ **for influenza A: threshold of 50 imported cases.**

The raincloud plots illustrate the distribution of $N_{i}^{\mathrm{risk}}$ across prefecture tiers for influenza A when a city’s population was included only after the cumulative number of imported infections reached 50 cases. Results are shown under three initial infection scenarios in the origin city (100, 500, and 1000 cases), evaluated at 7, 14, and 21 days after seeding. The bottom panels summarise the mean $N_{i}^{\mathrm{risk}}$ for each prefecture tier as a function of the number of initial infections (100–-1000). Under this threshold, $N_{i}^{\mathrm{risk}}$ was further attenuated, particularly in lower-tier cities, while the hierarchical pattern across tiers remained unchanged.

**Fig HH. Sensitivity analysis of alternative importation thresholds for** $\mathbf{N}_{\mathbf{i}}^{\mathbf{risk}}$ **for influenza A: threshold of 100 imported cases.**

The raincloud plots illustrate the distribution of $N_{i}^{\mathrm{risk}}$ across prefecture tiers for influenza A when a city’s population was included only after the cumulative number of imported infections reached 100 cases. Results are shown under three initial infection scenarios in the origin city (100, 500, and 1000 cases), evaluated at 7, 14, and 21 days after seeding. The bottom panels summarise the mean $N_{i}^{\mathrm{risk}}$ for each prefecture tier as a function of the number of initial infections (100–-1000). Under this strictest threshold, $N_{i}^{\mathrm{risk}}$ changed little across initial seeding sizes for most tiers within the examined time windows, indicating that few cities accumulated 100 imported infections, but the overall tier hierarchy remained stable.

**Supplementary Tables**

**Table A. Top five candidate origin cities with the highest absolute Pearson correlations between observed and predicted first arrival times in the Shanghai and Nanjing outbreaks.**

| **Outbreak** | **Rank** | **City** | **Pearson r** | **Permutation p-value** | **Bootstrap 95% CI** |
| --- | --- | --- | --- | --- | --- |
|  | 1 | Shanghai | 0.66 | <0.001 | [0.34,0.84] |
|  | 2 | Nantong | 0.60 | <0.001 | [0.41,0.73] |
| Shanghai | 3 | Suzhou | 0.54 | <0.001 | [0.35,0.73] |
|  | 4 | Taizhou | 0.49 | <0.001 | [0.35,0.67] |
|  | 5 | Yangzhou | 0.49 | <0.001 | [0.36,0.63] |
|  | 1 | Nanjing | 0.86 | 0.001 | [0.49,0.98] |
|  | 2 | Ma’anshan | 0.59 | 0.063 | [0.38,0.96] |
| Nanjing | 3 | Jingzhou | -0.46 | 0.091 | [-0.80,-0.37] |
|  | 4 | Suqian | 0.41 | 0.12 | [-0.069,0.91] |
|  | 5 | Wuhan | -0.35 | 0.08 | [-0.64,-0.28] |

Pearson r was computed between log-transformed observed and predicted first arrival times for each candidate origin city. Candidate origins were ranked by the absolute value of Pearson r, so both positive and negative correlations could be included among the top five. Permutation p-values and bootstrap 95% confidence intervals are reported to assess the robustness of the correlation estimates. For the Shanghai outbreak, Shanghai ranked first among eligible candidate origins (Pearson r=0.66, permutation p<0.001, bootstrap 95% CI [0.34,0.84]). For the Nanjing outbreak, Nanjing ranked first among eligible candidate origins (Pearson r=0.86, permutation p=0.001, bootstrap 95% CI [0.49,0.98]).

**Table B. Cross-tier comparisons of estimated population at risk in absolute and relative terms under alternative baseline migration probabilities for Shanghai.**

| **Day 7** | **People at risk (million)** | | | | | | | | | |
| --- | --- | --- | --- | --- | --- | --- | --- | --- | --- | --- |
| $p_{Sh}$(%) | Super-tier | Tier-1 | Tier-2 | Tier-3 | Tier-4 | Tier-5 | Super-tier − Tier-5 | Tier-1 − Tier-5 | Super-tier / Tier-5 | Tier-1 / Tier-5 |
| 0.5 | 32.3 | 18.7 | 8.9 | 6.1 | 3.8 | 1.9 | 30.4 | 16.8 | 16.9 | 9.8 |
| 0.6 | 31.3 | 20.5 | 9.0 | 6.0 | 3.8 | 1.9 | 29.3 | 18.5 | 16.1 | 10.6 |
| 0.7 | 36.0 | 21.3 | 9.1 | 6.1 | 3.9 | 2.0 | 34.0 | 19.3 | 17.7 | 10.5 |
| 0.8 | 36.0 | 21.8 | 9.6 | 6.4 | 3.9 | 2.1 | 33.9 | 19.7 | 17.5 | 10.6 |
| 0.9 | 37.5 | 22.6 | 9.6 | 6.6 | 4.0 | 2.1 | 35.4 | 20.5 | 18.1 | 10.9 |
| 1.0 | 41.0 | 23.3 | 10.0 | 6.6 | 4.2 | 2.1 | 38.9 | 21.2 | 19.3 | 11.0 |
| 1.1 | 42.6 | 24.2 | 10.4 | 6.8 | 4.3 | 2.1 | 40.5 | 22.1 | 19.9 | 11.3 |
| 1.2 | 42.6 | 24.6 | 10.7 | 6.8 | 4.2 | 2.2 | 40.4 | 22.5 | 19.5 | 11.3 |
| 1.3 | 44.6 | 26.5 | 10.5 | 7.0 | 4.4 | 2.2 | 42.3 | 24.3 | 20.1 | 11.9 |
| 1.4 | 46.1 | 26.5 | 11.1 | 7.0 | 4.5 | 2.2 | 43.8 | 24.2 | 20.6 | 11.8 |
| 1.5 | 49.2 | 27.7 | 10.6 | 7.2 | 4.5 | 2.3 | 46.9 | 25.3 | 21.2 | 11.9 |
| **Day 14** | **People at risk (million)** | | | | | | | | | |
| $p_{Sh}$(%) | Super-tier | Tier-1 | Tier-2 | Tier-3 | Tier-4 | Tier-5 | Super-tier − Tier-5 | Tier-1 − Tier-5 | Super-tier / Tier-5 | Tier-1 / Tier |
| 0.5 | 53.2 | 30.3 | 12.7 | 8.1 | 5.0 | 2.6 | 50.6 | 27.8 | 20.8 | 11.9 |
| 0.6 | 57.1 | 33.7 | 12.8 | 8.2 | 5.3 | 2.6 | 54.4 | 31.1 | 21.5 | 12.7 |
| 0.7 | 62.8 | 35.9 | 14.1 | 8.7 | 5.6 | 2.9 | 59.9 | 33.0 | 22.0 | 12.6 |
| 0.8 | 65.5 | 37.2 | 14.7 | 9.1 | 5.8 | 3.1 | 62.4 | 34.1 | 21.4 | 12.1 |
| 0.9 | 66.5 | 40.1 | 15.6 | 9.7 | 5.9 | 3.1 | 63.4 | 37.1 | 21.7 | 13.1 |
| 1.0 | 69.8 | 41.5 | 15.6 | 9.9 | 6.6 | 3.3 | 66.6 | 38.2 | 21.2 | 12.6 |
| 1.1 | 75.9 | 44.7 | 16.9 | 10.5 | 6.8 | 3.5 | 72.4 | 41.2 | 21.7 | 12.8 |
| 1.2 | 76.9 | 45.5 | 17.7 | 11.0 | 7.1 | 3.6 | 73.2 | 41.8 | 21.2 | 12.6 |
| 1.3 | 76.4 | 47.8 | 18.2 | 11.4 | 7.1 | 3.7 | 72.6 | 44.0 | 20.4 | 12.8 |
| 1.4 | 81.4 | 48.6 | 19.0 | 11.5 | 7.7 | 3.8 | 77.6 | 44.8 | 21.2 | 12.6 |
| 1.5 | 82.8 | 51.2 | 18.7 | 12.1 | 7.8 | 4.1 | 78.7 | 47.0 | 20.0 | 12.4 |
| **Day 21** | **People at risk (million)** | | | | | | | | | |
| $p_{Sh}$(%) | Super-tier | Tier-1 | Tier-2 | Tier-3 | Tier-4 | Tier-5 | Super-tier − Tier-5 | Tier-1 − Tier-5 | Super-tier / Tier-5 | Tier-1 / Tier |
| 0.5 | 78.9 | 47.6 | 19.2 | 12.1 | 7.7 | 4.0 | 74.8 | 43.6 | 19.6 | 11.8 |
| 0.6 | 85.9 | 51.6 | 20.0 | 12.6 | 8.5 | 4.3 | 81.6 | 47.3 | 20.1 | 12.1 |
| 0.7 | 91.6 | 55.5 | 22.7 | 14.4 | 9.2 | 4.6 | 87.0 | 50.9 | 19.8 | 12.0 |
| 0.8 | 93.3 | 59.2 | 24.0 | 14.6 | 9.8 | 5.2 | 88.1 | 54.0 | 18.0 | 11.4 |
| 0.9 | 98.8 | 62.3 | 26.0 | 16.1 | 10.3 | 5.4 | 93.4 | 56.9 | 18.4 | 11.6 |
| 1.0 | 100.4 | 64.4 | 26.8 | 17.0 | 11.7 | 5.7 | 94.7 | 58.7 | 17.6 | 11.3 |
| 1.1 | 109.9 | 69.2 | 28.9 | 18.0 | 12.3 | 6.3 | 103.6 | 62.9 | 17.4 | 11.0 |
| 1.2 | 108.5 | 70.6 | 29.9 | 18.6 | 12.9 | 6.5 | 102.0 | 64.1 | 16.6 | 10.8 |
| 1.3 | 111.4 | 72.7 | 31.5 | 19.8 | 13.4 | 6.7 | 104.7 | 66.0 | 16.7 | 10.9 |
| 1.4 | 114.9 | 75.6 | 32.6 | 20.2 | 14.0 | 7.1 | 107.9 | 68.5 | 16.2 | 10.7 |
| 1.5 | 113.4 | 78.0 | 32.3 | 21.6 | 14.8 | 7.6 | 105.8 | 70.4 | 15.0 | 10.3 |

Mean population at risk was estimated for each prefecture tier across alternative baseline daily migration probabilities for Shanghai ($p_{Sh}$=0.5% to 1.5%). To facilitate cross-tier comparisons, both absolute and relative measures are reported. Absolute comparisons are shown as differences in mean population at risk between tiers, whereas relative comparisons are shown as ratios between tiers. Results are presented for 7, 14, and 21 days after introduction, and population at risk is expressed in millions.

**Table C. Kruskal-Wallis and permutation-based p-values for cross-tier comparisons of** $\mathbf{N}_{\mathbf{i}}^{\mathbf{risk}}$**.**

| **pathogen** | **Time (days)** | **Kruskal–Wallis statistic** | **Kruskal–Wallis p-value** | **Permutation p-value** |
| --- | --- | --- | --- | --- |
|  | 7 | 142.58 | 5.06E-29 | <0.001 |
| Omicron | 14 | 175.4958 | 4.9E-36 | <0.001 |
|  | 21 | 169.5093 | 9.29E-35 | <0.001 |
|  | 7 | 198.3158 | 6.51E-41 | <0.001 |
| influenza | 14 | 159.5531 | 1.23E-32 | <0.001 |
|  | 21 | 133.518 | 4.26E-27 | <0.001 |

City-level values of $N_{i}^{\mathrm{risk}}$ were compared across prefecture tiers using the Kruskal–Wallis test. Because cities connected through the mobility network are not strictly independent, permutation-based p-values were additionally calculated by randomly permuting prefecture-tier labels across cities while preserving the number of cities in each tier. Results are shown for SARS-CoV-2 (Omicron) and influenza A at 7, 14, and 21 days.

**Table D. Latin hypercube-sampled parameter sets used in the joint probabilistic uncertainty analysis.**

| Param_id | Mean reproduction number, $\mu_{R}$ | Overdispersion,  𝑘 | Serial interval shape | Serial interval scale | Sigmoid scaling parameter | Baseline Shanghai migration probability |
| --- | --- | --- | --- | --- | --- | --- |
| 1 | 1.10 | 7.36 | 4.41 | 1.32 | 0.34 | 1.40% |
| 2 | 1.17 | 1.23 | 1.50 | 1.90 | 1.85 | 1.00% |
| 3 | 1.34 | 7.20 | 4.33 | 1.65 | 1.60 | 1.22% |
| 4 | 1.46 | 5.26 | 2.20 | 0.40 | 1.39 | 1.27% |
| 5 | 1.53 | 6.95 | 4.92 | 1.77 | 1.94 | 0.55% |
| 6 | 1.71 | 5.71 | 2.41 | 0.13 | 1.40 | 0.93% |
| 7 | 1.82 | 2.58 | 3.23 | 0.76 | 1.08 | 0.85% |
| 8 | 2.05 | 2.22 | 3.75 | 1.07 | 0.91 | 1.48% |
| 9 | 2.15 | 6.53 | 4.67 | 0.64 | 1.72 | 0.97% |
| 10 | 2.31 | 1.48 | 3.93 | 0.21 | 0.82 | 0.60% |
| 11 | 2.42 | 3.72 | 1.10 | 1.29 | 1.89 | 0.81% |
| 12 | 2.57 | 1.99 | 1.18 | 0.99 | 0.24 | 1.13% |
| 13 | 2.62 | 9.93 | 2.67 | 0.26 | 0.97 | 0.88% |
| 14 | 2.81 | 9.29 | 3.56 | 0.53 | 1.16 | 1.25% |
| 15 | 2.96 | 8.69 | 3.91 | 0.58 | 0.68 | 1.31% |
| 16 | 3.10 | 0.57 | 4.81 | 0.44 | 1.16 | 0.75% |
| 17 | 3.26 | 8.31 | 1.71 | 1.16 | 1.68 | 1.46% |
| 18 | 3.34 | 3.05 | 1.98 | 0.83 | 0.64 | 1.36% |
| 19 | 3.53 | 4.13 | 1.27 | 1.22 | 0.55 | 0.51% |
| 20 | 3.62 | 6.15 | 2.90 | 1.70 | 0.31 | 1.08% |
| 21 | 3.77 | 3.83 | 3.08 | 0.93 | 1.77 | 0.79% |
| 22 | 3.94 | 8.01 | 2.55 | 0.89 | 1.22 | 1.04% |
| 23 | 4.08 | 0.94 | 3.42 | 1.62 | 0.45 | 0.94% |
| 24 | 4.31 | 8.50 | 3.36 | 1.39 | 1.57 | 1.17% |
| 25 | 4.34 | 5.46 | 4.52 | 1.84 | 1.29 | 0.67% |
| 26 | 4.53 | 3.10 | 1.81 | 1.96 | 0.77 | 1.41% |
| 27 | 4.73 | 4.57 | 2.84 | 1.51 | 1.50 | 1.14% |
| 28 | 4.91 | 4.74 | 2.17 | 1.47 | 0.38 | 0.58% |

Twenty-eight parameter combinations generated by Latin hypercube sampling for the joint probabilistic uncertainty analysis. The jointly sampled parameters were the mean reproduction number ($\mu_{R}$), the overdispersion parameter of the offspring distribution ($k$), the shape and scale parameters of the gamma-distributed serial interval, the sigmoid scaling parameter used in the migration probability function, and the baseline outbound migration probability for Shanghai. The panel_id identifies the corresponding numbered panel in Figs X–Z.

**Table E. Cities reporting first confirmed cases linked to the Shanghai outbreak (n = 121).**

| City | Date | URL |
| --- | --- | --- |
| Shanghai | 2022/3/1 | <https://sh.bendibao.com/news/202231/248989.shtm> |
| Xuzhou | 2022/3/3 | <https://web.shobserver.com/wx/detail.do?id=457268> |
| Nantong | 2022/3/4 | <http://www.qidong.gov.cn/qdswjw/zytg/content/6995d633-3aeb-4b1f-b973-34b49f76e98b.html> |
| Jiaxing | 2022/3/5 | <https://www.chinanews.com/sh/2022/03-05/9693476.shtml> |
| Xi’an | 2022/3/5 | <https://baijiahao.baidu.com/s?id=1726463709777618522> |
| Jincheng | 2022/3/6 | <https://baijiahao.baidu.com/s?id=1726628466845322305> |
| Beijing | 2022/3/8 | <https://baijiahao.baidu.com/s?id=1726722523506205208> |
| Harbin | 2022/3/8 | <https://baijiahao.baidu.com/s?id=1726735168699019294> |
| Huzhou | 2022/3/8 | <https://huzhou.bendibao.com/news/202238/5108.shtm> |
| Baoji | 2022/3/8 | <https://www.workercn.cn/c/2022-03-08/6769037.shtml> |
| Suzhou (Jiangsu) | 2022/3/9 | <https://suzhou.bendibao.com/news/2022310/98264.shtm> |
| Yangzhou | 2022/3/9 | <https://doi.org/10.5281/zenodo.17852176> |
| Suzhou (Anhui) | 2022/3/9 | <https://baijiahao.baidu.com/s?id=1726866563573522195> |
| Nanjing | 2022/3/10 | <https://baijiahao.baidu.com/s?id=1726885363396126062> |
| Hangzhou | 2022/3/10 | <https://www.thepaper.cn/newsDetail_forward_17049020> |
| Sanya | 2022/3/11 | <https://doi.org/10.5281/zenodo.17852176> |
| Qiongzhong | 2022/3/11 | <https://baijiahao.baidu.com/s?id=1727006164868278491> |
| Suqian | 2022/3/12 | <https://www.sohu.com/a/529171613_114988> |
| Changzhou | 2022/3/13 | <https://cz.bendibao.com/news/2022313/58003.shtm> |
| Taizhou (Jiangsu) | 2022/3/13 | <https://news.sina.com.cn/c/2022-03-13/doc-imcwiwss5781649.shtml> |
| Nanchang | 2022/3/13 | <https://baijiahao.baidu.com/s?id=1727237243167707752> |
| Shangrao | 2022/3/13 | <https://doi.org/10.5281/zenodo.17852176> |
| Shangqiu | 2022/3/14 | <https://news.sina.com.cn/c/2022-03-14/doc-imcwiwss5976373.shtml> |
| Wuxi | 2022/3/15 | <https://baijiahao.baidu.com/s?id=1727411815007381215> |
| Zhenjiang | 2022/3/15 | <https://baijiahao.baidu.com/s?id=1727377302193798882> |
| Shiyan | 2022/3/15 | <https://m.gmw.cn/baijia/2022-03/16/1302847749.html> |
| Tongling | 2022/3/16 | <https://www.tl.gov.cn/openness/OpennessContent/show/589013.html> |
| Zhoushan | 2022/3/17 | <https://doi.org/10.5281/zenodo.17852176> |
| Yueyang | 2022/3/17 | <https://china.huanqiu.com/article/47ECNTAG2Cd> |
| Lishui | 2022/3/18 | <https://news.hangzhou.com.cn/gnxw/content/2022-03/18/content_8202268.htm> |
| Jiaozuo | 2022/3/18 | <https://baijiahao.baidu.com/s?id=1727995778105587590> |
| Bozhou | 2022/3/19 | <https://www.sohu.com/a/532365454_121230613> |
| Zhengzhou | 2022/3/19 | <https://m.gmw.cn/baijia/2022-03/19/1302853483.html> |
| Xinyang | 2022/3/19 | <https://baijiahao.baidu.com/s?id=1727698351769714286> |
| Yancheng | 2022/3/21 | <https://m.thepaper.cn/baijiahao_17223171> |
| Quanzhou | 2022/3/21 | <https://china.huanqiu.com/article/47J8jfMTo9Y> |
| Kaifeng | 2022/3/21 | <https://weibo.com/1702883112/LkJJourEf> |
| Changsha | 2022/3/21 | <https://m.gmw.cn/baijia/2022-03/22/1302857898.html> |
| Cangzhou | 2022/3/22 | <https://baijiahao.baidu.com/s?id=1728051298646907835> |
| Fuzhou | 2022/3/22 | <https://fz.bendibao.com/news/2022325/61802.shtm> |
| Jinan | 2022/3/22 | <https://m.thepaper.cn/baijiahao_17245040> |
| Yiyang | 2022/3/22 | <https://doi.org/10.5281/zenodo.17852176> |
| Longnan | 2022/3/22 | <https://baijiahao.baidu.com/s?id=1728076025009221966> |
| Linyi | 2022/3/23 | <https://linyi.bendibao.com/news/2022323/12172.shtm> |
| Xianning | 2022/3/23 | <https://www.gxnews.com.cn/staticpages/20220327/newgx62406314-20695183.shtml> |
| Hefei | 2022/3/24 | <https://www.163.com/dy/article/H38P34RF0534ISLK.html> |
| Wuhu | 2022/3/24 | <https://baijiahao.baidu.com/s?id=1728142277443751841> |
| Luohe | 2022/3/24 | <https://baijiahao.baidu.com/s?id=1728275384023254368> |
| Zhoukou | 2022/3/24 | <https://k.sina.com.cn/article_6192937794_17120bb4202001sko7.html> |
| Qiandongnan | 2022/3/24 | <https://baijiahao.baidu.com/s?id=1728150326577000387> |
| Shanwei | 2022/3/25 | <https://baijiahao.baidu.com/s?id=1728408588129013816> |
| Jinhua | 2022/3/26 | <https://mp.weixin.qq.com/s?__biz=MzA4ODY3MjkxNA==&mid=2651183379&idx=2&sn=9b84ece78850b174508f08cf59d2a0d6> |
| Qingyuan | 2022/3/26 | <https://m.gmw.cn/baijia/2022-03/28/1302869465.html> |
| Chongqing | 2022/3/26 | <https://www.163.com/dy/article/H3DFJLUP0514R9OJ.html> |
| Huaibei | 2022/3/27 | <https://doi.org/10.5281/zenodo.17852176> |
| Chuzhou | 2022/3/27 | <https://news.66wz.com/system/2022/04/03/105453790.shtml> |
| Nanyang | 2022/3/27 | <https://baijiahao.baidu.com/s?id=1728472271748468367> |
| Zhumadian | 2022/3/27 | <https://china.huanqiu.com/article/47N7DwE05tN> |
| Ezhou | 2022/3/27 | <https://baijiahao.baidu.com/s?id=1728578412362487908> |
| Maoming | 2022/3/27 | <https://doi.org/10.5281/zenodo.17852176> |
| Gannan | 2022/3/27 | <https://lz.bendibao.com/news/2022328/55194.shtm> |
| Baoding | 2022/3/28 | <https://baijiahao.baidu.com/s?id=1728676632892542367> |
| Taizhou (Zhejiang) | 2022/3/29 | <https://china.huanqiu.com/article/47OMKmmo1sg> |
| Ma’anshan | 2022/3/29 | <https://finance.sina.com.cn/jjxw/2022-03-30/doc-imcwipii1310783.shtml> |
| Putian | 2022/3/29 | <https://baijiahao.baidu.com/s?id=1728850064331876887> |
| Yantai | 2022/3/29 | <https://www.muping.gov.cn/art/2022/3/30/art_104096_2932813.html> |
| Taian | 2022/3/29 | <https://baijiahao.baidu.com/s?id=1728631067704870732> |
| Loudi | 2022/3/29 | <https://baijiahao.baidu.com/s?id=1728599238532605090> |
| Wanning | 2022/3/29 | <https://baijiahao.baidu.com/s?id=1728642816955688946> |
| Guiyang | 2022/3/29 | <https://doi.org/10.5281/zenodo.17852176> |
| Urumqi | 2022/3/29 | <https://baijiahao.baidu.com/s?id=1728719761943421652> |
| Changzhi | 2022/3/30 | <https://m.gmw.cn/baijia/2022-03/30/1302874317.html> |
| Suihua | 2022/3/30 | <https://m.gmw.cn/baijia/2022-03/30/1302874418.html> |
| Shaoxing | 2022/3/30 | <https://baijiahao.baidu.com/s?id=1728713786033418263> |
| Xiamen | 2022/3/30 | <https://www.xm.gov.cn/jdhy/xwfbh/xmsyqfkqk_20220330/> |
| Ningde | 2022/3/30 | <https://m.gmw.cn/baijia/2022-03/31/1302874729.html> |
| Weifang | 2022/3/30 | <https://baijiahao.baidu.com/s?id=1728717686059285508> |
| Heze | 2022/3/30 | <https://baijiahao.baidu.com/s?id=1728782805440063906> |
| Xiaogan | 2022/3/30 | <https://baijiahao.baidu.com/s?id=1728782388568478854> |
| Huanggang | 2022/3/30 | <https://doi.org/10.5281/zenodo.17852176> |
| Hengyang | 2022/3/30 | <https://news.sina.com.cn/c/2022-03-30/doc-imcwiwss9053002.shtml> |
| Xiangxi | 2022/3/30 | <https://baijiahao.baidu.com/s?id=1728777897563483541> |
| Nanning | 2022/3/30 | <https://m.gmw.cn/baijia/2022-03/30/1302873410.html> |
| Haikou | 2022/3/30 | <https://doi.org/10.5281/zenodo.17852176> |
| Leshan | 2022/3/30 | <https://www.sc.gov.cn/10462/10464/13722/2022/3/31/946eb487a4a14fb187666033c2994b2f.shtml> |
| Liupanshui | 2022/3/30 | <https://doi.org/10.5281/zenodo.17852176> |
| Kunming | 2022/3/30 | <https://www.sohu.com/a/533797411_162522> |
| Qujing | 2022/3/30 | <https://m.gmw.cn/baijia/2022-03/31/1302874713.html> |
| Pu’er | 2022/3/30 | <https://baijiahao.baidu.com/s?id=1728782290709961571> |
| Huaian | 2022/3/31 | <https://ha.bendibao.com/news/2022331/8728.shtm> |
| Ningbo | 2022/3/31 | <https://nb.bendibao.com/news/2022331/68031.shtm> |
| Quzhou | 2022/3/31 | <http://zj.people.com.cn/n2/2022/0401/c186327-35203462.html> |
| Jining | 2022/3/31 | <http://www.qufu.gov.cn/art/2022/3/31/art_18886_2728088.html> |
| Hebi | 2022/3/31 | <https://baijiahao.baidu.com/s?id=1728889615641009918> |
| Huangshi | 2022/3/31 | <https://china.huanqiu.com/article/47PwlLKVnl1> |
| Shantou | 2022/3/31 | <http://gd.people.com.cn/n2/2022/0401/c123932-35203298.html> |
| Huizhou | 2022/3/31 | <https://doi.org/10.5281/zenodo.17852176> |
| Zhongshan | 2022/3/31 | <https://china.huanqiu.com/article/47QQxmGtwzB> |
| Qionghai | 2022/3/31 | <https://doi.org/10.5281/zenodo.17852176> |
| Deyang | 2022/3/31 | <https://news.sina.com.cn/c/2022-03-31/doc-imcwipii1667494.shtml> |
| Handan | 2022/4/1 | <http://he.people.com.cn/n2/2022/0416/c192235-35226237.html> |
| Rizhao | 2022/4/1 | <https://china.huanqiu.com/article/47Qkb0Jqo9V> |
| Zhuhai | 2022/4/1 | <https://wsjkj.zhuhai.gov.cn/zwgk/ztzl/yqfkzt/yqxx/content/post_3126901.html> |
| Foshan | 2022/4/1 | <https://weibo.com/1668589317/LmsESrgBB> |
| Yulin | 2022/4/1 | <https://baijiahao.baidu.com/s?id=1729017196057631225> |
| Lingshui | 2022/4/1 | <https://news.sina.cn/2022-04-01/detail-imcwipii1775291.d.html> |
| Haidong | 2022/4/1 | <https://news.sina.com.cn/o/2022-04-01/doc-imcwiwss9433688.shtml> |
| Datong | 2022/4/2 | <https://baijiahao.baidu.com/s?id=1729032344371963648> |
| Wenzhou | 2022/4/2 | <https://china.huanqiu.com/article/47RhvHI0LpC> |
| Zibo | 2022/4/2 | <https://www.sohu.com/a/534722241_695881> |
| Jieyang | 2022/4/2 | <https://doi.org/10.5281/zenodo.17852176> |
| Qianxinan | 2022/4/2 | <https://www.guizhou.gov.cn/ztzl/fkxxgzbdgrdfy/qzblzyhdqy/qxnz_5633592/202204/t20220402_73239037.html> |
| Ziyang | 2022/4/3 | <https://news.sina.com.cn/c/2022-04-03/doc-imcwipii2203092.shtml> |
| Liangshan | 2022/4/3 | <https://news.sina.com.cn/c/2022-04-03/doc-imcwiwss9759694.shtml> |
| Xining | 2022/4/3 | <https://china.huanqiu.com/article/47SCZ5boloP> |
| Linfen | 2022/4/4 | <https://china.huanqiu.com/article/47TkOp69HM7> |
| Shenyang | 2022/4/4 | <https://baijiahao.baidu.com/s?id=1729254624208199808> |
| Zhaotong | 2022/4/4 | <http://yn.people.com.cn/n2/2022/0404/c378439-35207841.html> |
| Shijiazhuang | 2022/4/5 | <https://doi.org/10.5281/zenodo.17852176> |
| Xuchang | 2022/4/5 | <https://china.huanqiu.com/article/47U8fzUxmlx> |
| Nanchong | 2022/4/5 | <https://news.sina.com.cn/c/2022-04-10/doc-imcwiwst0996229.shtml> |

**Table F. Cities reporting first confirmed cases linked to the Nanjing outbreak (n = 28).**

| City | Date | URL |
| --- | --- | --- |
| Nanjing | 2021/7/20 | <https://nj.bendibao.com/news/2021721/111007.shtm> |
| Shenyang | 2021/7/22 | <https://sy.bendibao.com/news/2021722/63713.shtm> |
| Suqian | 2021/7/22 | <https://baijiahao.baidu.com/s?id=1706044396018256776> |
| Ma’anshan | 2021/7/22 | <https://www.thepaper.cn/newsDetail_forward_13722683> |
| Zhongshan | 2021/7/22 | <https://zh.bendibao.com/news/2021728/61252.shtm> |
| Mianyang | 2021/7/23 | <https://baijiahao.baidu.com/s?id=1706138486907362301> |
| Wuhu | 2021/7/25 | <https://wuhu.bendibao.com/news/2021725/13366.shtm> |
| Zhuhai | 2021/7/25 | <https://zh.bendibao.com/news/2021726/61135.shtm> |
| Dalian | 2021/7/27 | <https://baijiahao.baidu.com/s?id=1706351982960983326> |
| Chengdu | 2021/7/27 | <https://baijiahao.baidu.com/s?id=1706492776133266083> |
| Beijing | 2021/7/28 | <https://bj.bendibao.com/news/2021729/297072.shtm> |
| Yangzhou | 2021/7/28 | <https://yz.bendibao.com/news/2021426/11867.shtm> |
| Changde | 2021/7/28 | <https://cs.bendibao.com/news/2021730/77100.shtm> |
| Huaian | 2021/7/29 | <https://3w.huanqiu.com/a/6fd472/44BhZG9prFI?p_a6885ef14ddf7d919a31cad87a81a7c87> |
| Changsha | 2021/7/29 | <https://www.thepaper.cn/newsDetail_forward_13854902> |
| Zhuzhou | 2021/7/29 | <https://www.thepaper.cn/newsDetail_forward_13804238> |
| Zhangjiajie | 2021/7/29 | <https://m.thepaper.cn/baijiahao_13804030> |
| Xiangtan | 2021/7/30 | <https://baijiahao.baidu.com/s?id=1706713531580402856> |
| Xiangxi | 2021/7/30 | <https://baijiahao.baidu.com/s?id=1706698357735460726> |
| Chongqing | 2021/7/30 | <https://china.huanqiu.com/article/44EWWEYARYI> |
| Yantai | 2021/7/31 | <https://baijiahao.baidu.com/s?id=1706850411733508395> |
| Huanggang | 2021/7/31 | <https://wh.bendibao.com/news/202182/129760.shtm> |
| Yinchuan | 2021/7/31 | <http://nx.people.com.cn/GB/n2/2021/0805/c192493-34853220.html> |
| Jingzhou | 2021/8/1 | <https://baijiahao.baidu.com/s?id=1706940897995293679> |
| Yiyang | 2021/8/1 | <https://baijiahao.baidu.com/s?id=1706872735295507617> |
| Haikou | 2021/8/1 | <https://news.sina.com.cn/o/2021-08-01/doc-ikqcfncc0232553.shtml> |
| Wuhan | 2021/8/2 | <https://baijiahao.baidu.com/s?id=1707168311407113760> |
| Hulunbuir | 2021/8/5 | <https://www.thepaper.cn/newsDetail_forward_13904919> |

**Table G. Cities reporting first confirmed cases linked to the northwestern China outbreak (n = 26).**

| City | Date | URL |
| --- | --- | --- |
| Xi’an | 2021/10/17 | <https://m.thepaper.cn/baijiahao_14952140> |
| Changsha | 2021/10/18 | <https://baijiahao.baidu.com/s?id=1713994354578756969> |
| Yinchuan | 2021/10/18 | <https://baijiahao.baidu.com/s?id=1713917546772606961> |
| Beijing | 2021/10/19 | <https://baijiahao.baidu.com/s?id=1714032055586817140> |
| Alashan | 2021/10/19 | <http://www.mnw.cn/news/china/2530264.html> |
| Zunyi | 2021/10/19 | <https://gy.bendibao.com/news/20211026/56296.shtm> |
| Lanzhou | 2021/10/19 | <https://www.wuhan.com/xinwen/78210.html> |
| Wuzhong | 2021/10/19 | <https://baijiahao.baidu.com/s?id=1714100448884931195> |
| Xingtai | 2021/10/20 | <https://doi.org/10.5281/zenodo.17852176> |
| Tianmen | 2021/10/20 | <https://www.thepaper.cn/newsDetail_forward_15002571> |
| Zhangye | 2021/10/20 | <https://baijiahao.baidu.com/s?id=1714123699738251566> |
| Baoding | 2021/10/21 | <https://m.thepaper.cn/baijiahao_15005663> |
| Jiayuguan | 2021/10/21 | <https://news.cctv.com/2021/10/22/ARTIvkWiyFUCeXOJAC1t3WuZ211022.shtml> |
| Haidong | 2021/10/21 | <https://baijiahao.baidu.com/s?id=1714260806358192720> |
| Longnan | 2021/10/22 | <https://baijiahao.baidu.com/s?id=1714472762737332223> |
| Hohhot | 2021/10/23 | <https://baijiahao.baidu.com/s?id=1714378957617877124> |
| Zhuzhou | 2021/10/23 | <https://baijiahao.baidu.com/s?id=1714384992741896525> |
| Zigong | 2021/10/24 | <https://cd.bendibao.com/news/2021824/127942.shtm> |
| Rizhao | 2021/10/25 | <https://m.thepaper.cn/baijiahao_15072427> |
| Zhongwei | 2021/10/25 | <https://baijiahao.baidu.com/s?id=1714669827973966842> |
| Xining | 2021/10/28 | <https://xa.bendibao.com/news/20211028/89491.shtm> |
| Shijiazhuang | 2021/10/31 | <https://www.tsr.he.cn/xwtj/2021-11/02/cms130090article.shtml> |
| Chongqing | 2021/11/2 | <https://finance.sina.com.cn/jjxw/2021-11-03/doc-iktzscyy3482705.shtml> |
| Chengdu | 2021/11/2 | <https://www.sohu.com/a/498800177_121123732> |
| Changzhou | 2021/11/3 | <https://wan.cncn.com/guide_29913.htm> |
| Jiaxing | 2021/11/3 | <https://baijiahao.baidu.com/s?id=1715515796926889533> |

**Table H. List of super-tier cities in China, 2023 (n = 4).**

| City name | City tier | City name | City tier |
| --- | --- | --- | --- |
| Beijing | Super tier | Shanghai | Super tier |
| Guangzhou | Super tier | Shenzhen | Super tier |

**Table I. List of tier-1 cities in China, 2023 (n = 15).**

| City name | City tier | City name | City tier | City name | City tier |
| --- | --- | --- | --- | --- | --- |
| Chengdu | First-tier | Chongqing | First-tier | Hangzhou | First-tier |
| Wuhan | First-tier | Suzhou | First-tier | Xi’an | First-tier |
| Nanjing | First-tier | Changsha | First-tier | Tianjin | First-tier |
| Zhengzhou | First-tier | Dongguan | First-tier | Qingdao | First-tier |
| Kunming | First-tier | Ningbo | First-tier | Hefei | First-tier |

**Table J. List of tier-2 cities in China, 2023 (n = 30).**

| City name | City tier | City name | City tier | City name | City tier |
| --- | --- | --- | --- | --- | --- |
| Foshan | Second-tier | Shenyang | Second-tier | Wuxi | Second-tier |
| Jinan | Second-tier | Xiamen | Second-tier | Fuzhou | Second-tier |
| Wenzhou | Second-tier | Harbin | Second-tier | Shijiazhuang | Second-tier |
| Dalian | Second-tier | Nanning | Second-tier | Quanzhou | Second-tier |
| Jinhua | Second-tier | Guiyang | Second-tier | Changzhou | Second-tier |
| Changchun | Second-tier | Nanchang | Second-tier | Nantong | Second-tier |
| Jiaxing | Second-tier | Xuzhou | Second-tier | Huizhou | Second-tier |
| Taiyuan | Second-tier | Taizhou | Second-tier | Shaoxing | Second-tier |
| Baoding | Second-tier | Zhongshan | Second-tier | Weifang | Second-tier |
| Linyi | Second-tier | Zhuhai | Second-tier | Yantai | Second-tier |

**Table K. List of tier-3 cities in China, 2023 (n = 70).**

| City name | City tier | City name | City tier | City name | City tier |
| --- | --- | --- | --- | --- | --- |
| Lanzhou | Third-tier | Haikou | Third-tier | Huzhou | Third-tier |
| Yangzhou | Third-tier | Luoyang | Third-tier | Shantou | Third-tier |
| Yancheng | Third-tier | Ganzhou | Third-tier | Tangshan | Third-tier |
| Urumqi | Third-tier | Jining | Third-tier | Zhenjiang | Third-tier |
| Langfang | Third-tier | Xianyang | Third-tier | Taizhou | Third-tier |
| Wuhu | Third-tier | Handan | Third-tier | Jieyang | Third-tier |
| Nanyang | Third-tier | Hohhot | Third-tier | Fuyang | Third-tier |
| Jiangmen | Third-tier | Yinchuan | Third-tier | Zunyi | Third-tier |
| Huaian | Third-tier | Zhangzhou | Third-tier | Guilin | Third-tier |
| Zibo | Third-tier | Xinxiang | Third-tier | Lianyungang | Third-tier |
| Cangzhou | Third-tier | Mianyang | Third-tier | Hengyang | Third-tier |
| Shangqiu | Third-tier | Heze | Third-tier | Xinyang | Third-tier |
| Xiangyang | Third-tier | Chuzhou | Third-tier | Shangrao | Third-tier |
| Jiujiang | Third-tier | Yichang | Third-tier | Putian | Third-tier |
| Zhanjiang | Third-tier | Lizhou | Third-tier | Anqing | Third-tier |
| Suqian | Third-tier | Zhaoqing | Third-tier | Zhoukou | Third-tier |
| Xingtai | Third-tier | Jingzhou | Third-tier | Sanya | Third-tier |
| Yueyang | Third-tier | Bengbu | Third-tier | Zhumadian | Third-tier |
| Taian | Third-tier | Chaozhou | Third-tier | Zhuzhou | Third-tier |
| Weihai | Third-tier | Lu’an | Third-tier | Changde | Third-tier |
| Anyang | Third-tier | Suzhou | Third-tier | Huanggang | Third-tier |
| Dezhou | Third-tier | Ningde | Third-tier | Liaocheng | Third-tier |
| Yichun | Third-tier | Weinan | Third-tier | Qingyuan | Third-tier |
| Nanchong | Third-tier |  |  |  |  |

**Table L. List of tier-4 cities in China, 2023 (n = 90).**

| City name | City tier | City name | City tier | City name | City tier |
| --- | --- | --- | --- | --- | --- |
| Ma’anshan | Fourth-tier | Kaifeng | Fourth-tier | Bozhou | Fourth-tier |
| Lishui | Fourth-tier | Chenzhou | Fourth-tier | Anshan | Fourth-tier |
| Xuancheng | Fourth-tier | Xuchang | Fourth-tier | Quzhou | Fourth-tier |
| Xiaogan | Fourth-tier | Zaozhuang | Fourth-tier | Yibin | Fourth-tier |
| Shaoyang | Fourth-tier | Qinhuangdao | Fourth-tier | Luzhou | Fourth-tier |
| Zhoushan | Fourth-tier | Nanping | Fourth-tier | Jilin | Fourth-tier |
| Longyan | Fourth-tier | Sanming | Fourth-tier | Qujing | Fourth-tier |
| Meizhou | Fourth-tier | Deyang | Fourth-tier | Xiangtan | Fourth-tier |
| Baoji | Fourth-tier | Maoming | Fourth-tier | Jiaozuo | Fourth-tier |
| Yuncheng | Fourth-tier | Pingdingshan | Fourth-tier | Yulin | Fourth-tier |
| Shanwei | Fourth-tier | Huaihua | Fourth-tier | Ji'an | Fourth-tier |
| Rizhao | Fourth-tier | Huangshi | Fourth-tier | Huainan | Fourth-tier |
| Ordos | Fourth-tier | Qiandongnan | Fourth-tier | Baotou | Fourth-tier |
| Shaoguan | Fourth-tier | Yongzhou | Fourth-tier | Leshan | Fourth-tier |
| Dongying | Fourth-tier | Jinzhong | Fourth-tier | Binzhou | Fourth-tier |
| Yingkou | Fourth-tier | Zhangjiakou | Fourth-tier | Enshi | Fourth-tier |
| Daqing | Fourth-tier | Xianning | Fourth-tier | Bijie | Fourth-tier |
| Heyuan | Fourth-tier | Shiyan | Fourth-tier | Loudi | Fourth-tier |
| Fuzhou | Fourth-tier | Hanzhong | Fourth-tier | Jinzhou | Fourth-tier |
| Jingdezhen | Fourth-tier | Meishan | Fourth-tier | Dali | Fourth-tier |
| Xining | Fourth-tier | Yulin | Fourth-tier | Panjin | Fourth-tier |
| Linfen | Fourth-tier | Hengshui | Fourth-tier | Huangshan | Fourth-tier |
| Honghe | Fourth-tier | Qiannan | Fourth-tier | Chengde | Fourth-tier |
| Puyang | Fourth-tier | Yiyang | Fourth-tier | Yangjiang | Fourth-tier |
| Changzhi | Fourth-tier | Qiqihar | Fourth-tier | Datong | Fourth-tier |
| Tongren | Fourth-tier | Chifeng | Fourth-tier | Neijiang | Fourth-tier |
| Dazhou | Fourth-tier | Beihai | Fourth-tier | Jingmen | Fourth-tier |
| Tongling | Fourth-tier | Xishuangbanna | Fourth-tier | Suining | Fourth-tier |
| Ezhou | Fourth-tier | Jiamusi | Fourth-tier | Huaibei | Fourth-tier |
| Luohe | Fourth-tier | Mudanjiang | Fourth-tier | Yanbian | Fourth-tier |

**Table M. List of tier-5 cities in China, 2023 (n = 128).**

| City name | City tier | City name | City tier | City name | City tier |
| --- | --- | --- | --- | --- | --- |
| Zhaotong | Fifth-tier | Liangshan | Fifth-tier | Guang’an | Fifth-tier |
| Dandong | Fifth-tier | Liupanshui | Fifth-tier | Lhasa | Fifth-tier |
| Liaoyang | Fifth-tier | Yan’an | Fifth-tier | Tieling | Fifth-tier |
| Huludao | Fifth-tier | Fushun | Fifth-tier | Yuxi | Fifth-tier |
| Baise | Fifth-tier | Baoshan | Fifth-tier | Pingxiang | Fifth-tier |
| Guangyuan | Fifth-tier | Sanmenxia | Fifth-tier | Xinyu | Fifth-tier |
| Xiangxi | Fifth-tier | Jincheng | Fifth-tier | Tianshui | Fifth-tier |
| Lvliang | Fifth-tier | Suizhou | Fifth-tier | Xinzhou | Fifth-tier |
| Chaoyang | Fifth-tier | Chizhou | Fifth-tier | Zigong | Fifth-tier |
| Suihua | Fifth-tier | Zhangjiajie | Fifth-tier | Hulunbuir | Fifth-tier |
| Lijiang | Fifth-tier | Yunfu | Fifth-tier | Pu’er | Fifth-tier |
| Wuzhou | Fifth-tier | Yingtan | Fifth-tier | Anshun | Fifth-tier |
| Guigang | Fifth-tier | Dehong | Fifth-tier | Siping | Fifth-tier |
| Hebi | Fifth-tier | Qingyang | Fifth-tier | Benxi | Fifth-tier |
| Ankang | Fifth-tier | Tonghua | Fifth-tier | Qinzhou | Fifth-tier |
| Chuxiong | Fifth-tier | Wenshan | Fifth-tier | Tongliao | Fifth-tier |
| Ziyang | Fifth-tier | Fuxin | Fifth-tier | Songyuan | Fifth-tier |
| Qianxinan | Fifth-tier | Yili | Fifth-tier | Kashgar | Fifth-tier |
| Bayingolin | Fifth-tier | Jiuquan | Fifth-tier | Aksu | Fifth-tier |
| Bazhong | Fifth-tier | Xilingol | Fifth-tier | Hechi | Fifth-tier |
| Dingxi | Fifth-tier | Bayan Nur | Fifth-tier | Jixi | Fifth-tier |
| Panzhihua | Fifth-tier | Heihe | Fifth-tier | Hezhou | Fifth-tier |
| Baishan | Fifth-tier | Laibin | Fifth-tier | Ya’an | Fifth-tier |
| Ulanqab | Fifth-tier | Lincang | Fifth-tier | Yangquan | Fifth-tier |
| Karamay | Fifth-tier | Jiayuguan | Fifth-tier | Wuhai | Fifth-tier |
| Baiyin | Fifth-tier | Zhangye | Fifth-tier | Changji | Fifth-tier |
| Fangchenggang | Fifth-tier | Shangluo | Fifth-tier | Wuzhong | Fifth-tier |
| Baicheng | Fifth-tier | Pingliang | Fifth-tier | Shuangyashan | Fifth-tier |
| Hetian | Fifth-tier | Tongchuan | Fifth-tier | Longnan | Fifth-tier |
| Hegang | Fifth-tier | Shuozhou | Fifth-tier | Liaoyuan | Fifth-tier |
| Qitaihe | Fifth-tier | Jinchang | Fifth-tier | Hami | Fifth-tier |
| Alashan | Fifth-tier | Shizuishan | Fifth-tier | Xing’an | Fifth-tier |
| Yichun | Fifth-tier | Chongzuo | Fifth-tier | Linzhi | Fifth-tier |
| Guyuan | Fifth-tier | Linxia | Fifth-tier | Haixi | Fifth-tier |
| Danzhou | Fifth-tier | Nujiang | Fifth-tier | Wuwei | Fifth-tier |
| Haidong | Fifth-tier | Aletai | Fifth-tier | Zhongwei | Fifth-tier |
| Daxing'anling | Fifth-tier | Tacheng | Fifth-tier | Bortala | Fifth-tier |
| Changdu | Fifth-tier | Diqing | Fifth-tier | Shigatse | Fifth-tier |
| Ganzi | Fifth-tier | Turpan | Fifth-tier | Ngawa | Fifth-tier |
| Shannan | Fifth-tier | Gannan | Fifth-tier | Ali | Fifth-tier |
| Golog | Fifth-tier | Naqu | Fifth-tier | Sansha | Fifth-tier |
| Hainan | Fifth-tier | Haibei | Fifth-tier | Yushu | Fifth-tier |
| Kizilsu | Fifth-tier | Huangnan | Fifth-tier |  |  |

**References**

1. School of Architecture and Urban Planning, Tongji University. Yangtze River Delta Inter-city Commuting Annual Report, 2022 Shanghai2022 [cited 2023 June 30]. Available from: <https://www.shplanning.com.cn/information/detail/id/288.html>.

2. Einstein A. On the motion of small particles suspended in liquids at rest required by the molecular-kinetic theory of heat. Ann Phys (Berl). 1905;17:549-60.

3. Wu Y, Guo Z, Yuan J, Cao G, Wang Y, Gao P, et al. Duration of viable virus shedding and polymerase chain reaction positivity of the SARS-CoV-2 Omicron variant in the upper respiratory tract: a systematic review and meta-analysis. Int J Infect Dis. 2023;129:228-35.

4. Cai J, Deng X, Yang J, Sun K, Liu H, Chen Z, et al. Modeling transmission of SARS-CoV-2 Omicron in China. Nat Med. 2022;28(7):1468-75.
