# Supplementary material for: Assessing spatial transmission risk of respiratory infectious diseases across cities of different socioeconomic tiers in China: A modelling study
Source: PLoS Med. 2026 Jul 20;23(7):e1005172. doi: 10.1371/journal.pmed.1005172 (PMC13384292; doi:10.1371/journal.pmed.1005172)
Supplement: Attachment — Submitted filename: Response to Reviewers R2_submission.pdf [file pmed.1005172.s002.pdf]

**Manuscript Number: PMEDICINE-D-25-04489**

**Assessing spatial transmission risk of respiratory infectious diseases across urban tiers in China: a modelling study**

**Wenjie Li, Wei Yang, Yang Liu, Ye Yao**

**PLOS Medicine**

---

**Response to Reviewers**

We thank the editor and the anonymous reviewers for their careful and constructive evaluation of our manuscript. We greatly appreciate the insightful comments, which have helped us to substantially improve the clarity, rigour, and positioning of the study.

In the revised manuscript, we have clarified the conceptual contribution of our work, particularly the generalisable mechanism linking transmissibility, mobility, and urban hierarchy. We have strengthened the discussion of generalisability beyond the specific study setting and expanded the validation framework by incorporating additional complementary metrics. We have also provided further methodological details to improve transparency and reproducibility. We have carefully addressed all comments and provided a detailed, point-by-point response below. All revisions are clearly indicated in the marked-up manuscript.

## Response to Editorial Comment on Novelty and Positioning

We thank the editor for this direct and important feedback. We fully agree that the use of mobility data in modelling respiratory disease transmission is well established, and that our original submission did not sufficiently distinguish the specific advance of our work from this existing body of literature.

In the revised manuscript, we have therefore undertaken a substantial reframing to clarify both the study's conceptual contribution and its broader relevance. In particular, we now position the work around three complementary contributions:

(1) high-fidelity empirical validation against infection data collected under exceptional surveillance conditions;

(2) generation of sub-national, tier-stratified evidence to inform regional public health management;

(3) a direct empirical comparison of how mobility shapes spatial diffusion across respiratory pathogens with contrasting transmission profiles.

These contributions are now explicitly articulated in the Introduction and Discussion, and are further elaborated in our detailed responses to the reviewers' comments below.

## Reviewer 2

This manuscript presents a mobility-informed, agent-based branching process framework to characterise the spatial transmission risk of respiratory infectious diseases across urban tiers in China. By integrating large-scale Baidu Migration data with a city-level branching process model, the authors examine tier-specific diffusion patterns and validate their approach using three well-documented COVID-19 outbreaks. The comparison between SARS-CoV-2 (Omicron) and Influenza A provides a contrast between highly transmissible and more moderately transmissible pathogens that is potentially relevant for public-health preparedness.

Overall, the study addresses an important question in spatial epidemiology. However, similar mobility-based modelling approaches have been extensively explored in the literature, and the conceptual novelty of the current work is limited. Moreover, it is unclear whether the primary outcome "first arrival time" has sufficient public-health relevance to justify the complexity of the proposed framework or to serve as a robust validation target. In practice, outbreak origins are typically identified through epidemiological and outbreak investigation efforts, raising the question of what additional actionable insight this model provides beyond existing approaches.

While the use of large-scale mobile phone-derived mobility data is a strength, I remain unconvinced that the framework, as currently presented, offers clear or substantial added value for public-health decision-making. Given these concerns, I am hesitant to recommend publication in PLOS Medicine. My specific comments are outlined below.

**Response**: We thank the reviewer for the careful evaluation of our manuscript and for raising important concerns regarding novelty, validation, and policy relevance. We also appreciate the recognition of the importance of the research question and the strengths of the data and comparative design.

We acknowledge that, in the original submission, the study's conceptual contribution and practical implications were not sufficiently clearly articulated. We agree that the modelling framework's conceptual novelty is incremental. In the revised manuscript, we have undertaken a substantial reframing to better distinguish our work from existing mobility-based modelling studies, clarify the role of first arrival time as an early-phase and policy-

relevant metric, and strengthen the discussion of how the framework can inform public health decision-making. The central question we address is not whether mobility matters for epidemic spread, which is well established, but whether early spatial diffusion can be quantified in a way that is both empirically testable and operationally relevant for sub-national public health decision-making.

**Novelty**

1. **High-fidelity empirical validation against infection data collected under exceptional surveillance conditions**

   *This contribution lies in enabling high-fidelity empirical validation of mobility-driven models using directly observed intercity transmission events, rather than relying on statistically inferred proxies. This contribution is therefore empirical rather than methodological in nature.*

   A central difficulty in empirical validation of mobility-driven models of respiratory disease spread is that the underlying ground truth, specifically the sequence and timing of intercity infector-infectee transmission events, is rarely observed directly. For endemic respiratory pathogens such as seasonal influenza, validation of predicted geographic spread typically relies on aggregated surveillance data that are themselves filtered through healthcare-seeking behaviour, case definitions, and laboratory confirmation practices that vary across settings. Under these conditions, the apparent first-arrival time of a pathogen in a given city reflects a combination of the true introduction time and the local detection probability, and the infector-infectee relationships underlying any observed spread must be reconstructed through statistical inference rather than observed directly.

   The three COVID-19 outbreaks analysed here offer a rare exception to this general pattern. They occurred under conditions of near-exhaustive population testing, comprehensive contact tracing, and centralised case reporting associated with China's dynamic zero-COVID strategy. For each confirmed case, epidemiological investigation recorded travel history, contact networks, and documented transportation records; population-wide nucleic acid amplification testing was deployed in affected areas, often within days of case

detection; and local authorities published case lists linking infections to specific outbreak chains. Crucially, SARS-CoV-2 was a novel pathogen for which population-level immunity was minimal at the time of the outbreaks, meaning that each detected case represented a genuine recent infection rather than a reactivation or asymptomatic seropositive finding. The combination of a novel pathogen, a fully susceptible population, and an unprecedented surveillance response produced a dataset in which long-distance intercity transmission events, including the infector-infectee links that seeded new city-level outbreaks, could be identified with a degree of confidence that is unusual in respiratory epidemiology.

This data environment enables a validation strategy that would be difficult to replicate for other respiratory pathogens in real-world settings. Rather than relying on regression-based inference of likely transmission routes from aggregate case counts, we directly compare model-predicted first-arrival times with observed first-arrival times documented through outbreak investigation. The validation, therefore, tests the model's ability to reproduce real transmission events rather than its ability to fit an inferred statistical quantity. The high correlations obtained (r=0.68 for Shanghai, r=0.76 for Nanjing, r=0.56 for northwestern China), together with the successful retrospective identification of outbreak origins, indicate that the framework captures genuine diffusion patterns rather than artefacts of surveillance coverage. For other respiratory pathogens, where the ground truth must itself be inferred, claims about mobility-driven diffusion rest on a weaker empirical foundation. Our framework is not intended to replace epidemiological investigation, but to provide a rapid, data-driven approximation in settings where detailed investigation is delayed, incomplete, or unavailable.

The advance here is therefore not simply the use of a mobility-informed branching-process model, which, as several reviewers noted, has been applied to respiratory disease in various forms [1-3]. The advance lies in the ability to validate such a model against directly observed transmission events in a national urban network, using a dataset whose epidemiological reliability is unlikely to be reproducible under ordinary surveillance conditions. This strengthens confidence that mobility structure, independently of

pathogen-specific epidemiological parameters, governs the ordering of intercity seeding during the earliest phase of spread.

2. **Sub-national, tier-stratified evidence to inform regional public health management**
*This contribution lies in quantifying how early transmission risk is structured across urban hierarchies within a country, providing a basis for regionally differentiated public health strategies.*

Existing empirical evidence on the effectiveness of internal movement restrictions for respiratory infectious disease control has primarily been derived from between-country comparisons [4]. Such analyses have been instrumental in establishing the effectiveness of non-pharmaceutical interventions at the national level. However, they are not designed to characterise how transmission risk is distributed within countries, where intercity connectivity, population size, and other contextual factors vary substantially. A complementary line of inquiry concerns the sub-national distribution of early transmission risk: within a country, where early introduction risk is concentrated across cities and how it propagates through the mobility network. While these questions are central to practical public health decision-making, quantitative evidence addressing them remains relatively limited, particularly at high spatial resolution and in settings with empirically characterised mobility networks.

Our study builds on this body of work by addressing these questions through a tier-stratified analysis of intercity diffusion across 366 Chinese cities. By grouping cities into six tiers reflecting shared demographic, socioeconomic, and connectivity profiles, and by simulating intercity spread under standardised conditions, we quantify how early transmission risk is distributed across the urban hierarchy. The analysis shows that super-tier and tier-1 cities experience substantially greater early importation risk, that this gradient is robust across parameter assumptions, and that the pace at which lower-tier cities become affected varies with pathogen transmissibility.

These findings translate directly into policy-relevant insights: which groups of cities should be prioritised for early surveillance, how much lead time may be available before lower-tier areas are affected, and how risk distribution evolves during the early phase of spread. Rather than implying that each city requires a distinct response, a tier-based framework allows cities with similar structural characteristics to share common protocols, while preserving meaningful differentiation across tiers. In this sense, tier-stratified approaches offer a practical balance between uniform national measures and highly localised planning. Our work, therefore, complements existing evidence on non-pharmaceutical interventions by extending the analysis from the national to the sub-national scale, providing quantitative support for tier-based public health strategies that have been implemented in multiple settings but have not previously been informed by high-resolution mobility-based diffusion modelling.

3. **Direct empirical comparison of how mobility shapes diffusion across respiratory pathogens with contrasting transmission profiles**

*This contribution lies in explicitly incorporating a direct empirical comparison of how a shared mobility network shapes early spatial diffusion across respiratory pathogens with contrasting transmission characteristics, thereby informing an important and previously unresolved policy question of whether internal movement restrictions and other regionally targeted measures are likely to exert comparable effects across different respiratory pathogens.*

Previous studies have demonstrated the importance of human mobility in shaping the spread of respiratory infectious diseases, examining associations between travel volume and seeding probability [5], commuting intensity and spatial spread [6], and air travel and international importation risk [2]. These analyses have largely focused on individual pathogens, providing important insights into how mobility influences transmission dynamics within specific epidemiological contexts. An important complementary question is whether and how these mobility-transmission relationships differ across pathogens with distinct epidemiological characteristics.

Respiratory pathogens differ substantially in key epidemiological parameters, including generation interval, serial interval, the contribution of presymptomatic transmission, and the degree of transmission heterogeneity (overdispersion). These differences are expected to influence how sensitively an epidemic responds to mobility structure. For example, pathogens with shorter generation intervals and higher overdispersion may traverse mobility networks more rapidly via stochastic superspreading events. In contrast, pathogens with longer generation intervals and more homogeneous transmission may exhibit slower, more structured spatial diffusion. Our study builds on this literature by directly comparing SARS-CoV-2 (Omicron BA.2) and influenza A within a shared intercity mobility system. This comparison reveals marked differences in how the two pathogens propagate across the urban hierarchy: Omicron exhibits rapid cross-tier dissemination, with the tier-level risk gradient eroding within approximately two weeks of seeding, whereas influenza A remains largely tier-bounded over the same period. These differences arise from the interaction between transmissibility, transmission heterogeneity, and mobility network topology, rather than from mean transmissibility alone.

By providing direct empirical evidence that the role of mobility in shaping spatial diffusion is pathogen-dependent, our findings inform an important and previously unresolved policy question: whether internal movement restrictions and other regionally targeted measures are likely to have comparable effects across respiratory pathogens. While our analysis does not directly estimate intervention effects, the observed divergence in cross-tier diffusion patterns suggests that the expected impact and timing of such measures may differ across pathogens, and that response strategies may need to be adapted to pathogen-specific transmission characteristics.

To our knowledge, this represents one of the first empirical comparisons of how a shared intercity mobility network translates into differential early spatial diffusion across pathogens with contrasting transmission profiles. More broadly, our findings are consistent with and extend prior work [7] by demonstrating that the role of mobility depends not only on behavioural patterns but also on pathogen-specific transmission characteristics and spatial scale.

**Content Added to Introduction (Page 6, Lines 126-141)**

*A central limitation in validating mobility-driven models of respiratory disease spread is the difficulty of directly observing intercity infector-infectee relationships, such that validation typically relies on statistically inferred transmission patterns. Here, we leverage a uniquely detailed dataset from three COVID-19 outbreaks in China to enable validation against directly observed transmission events. More broadly, this study makes three contributions. First, it provides high-fidelity empirical validation of mobility-driven diffusion models using directly observed transmission events, strengthening the empirical grounding of such approaches. Second, it quantifies how early transmission risk is structured across urban hierarchies within a country, generating sub-national, tier-stratified evidence to inform regionally differentiated public health strategies. Third, it explicitly compares the empirical effects of a shared mobility network on spatial diffusion across respiratory pathogens with contrasting transmission characteristics, thereby informing an important and previously unresolved policy question: whether internal movement restrictions and other regionally targeted measures are likely to exert comparable effects across different respiratory pathogens.*

**Major Comments:**

1. Dependence on mobility patterns during China's "dynamic zero-COVID" period

   The mobility data used in this study are drawn from a period characterised by substantial but structured mobility restrictions under China's "dynamic zero-COVID" policy. Although this context is acknowledged, the implications for generalisability are not sufficiently discussed. How might the observed tier-specific diffusion patterns differ under post-2022 mobility regimes, or in settings without comparable intervention intensity? The authors should more clearly frame China as a specific case study or proof-of-concept and discuss the extent to which the findings and the proposed framework can be transferred to other epidemiological and policy contexts.

   <u>**Response**</u>: We thank the reviewer for this important comment. We agree that the mobility data used in our main analysis were collected during China's "dynamic zero-COVID" period, when substantial but structured public health interventions influenced intercity travel. We

have therefore revised the manuscript to state this more explicitly and to clarify that the main-text estimates should first be interpreted within this specific epidemiological and policy context, rather than as representing unconstrained mobility conditions.

To address the reviewer's question regarding post-2022 mobility regimes, we conducted an additional robustness analysis using mobility data from 2023, after the end of the "dynamic zero-COVID" strategy. We first compared intercity mobility matrices across four observed periods: the Nanjing Delta outbreak, the Shanghai Omicron outbreak, the full year 2021, and the full year 2023. Visual comparison of the matrices showed very similar overall structure, including persistent concentration of mobility along a limited set of highly connected origin-destination pairs, and pairwise quantitative comparisons confirmed high similarity across periods, with Pearson correlations ranging from 0.930 to 0.995. These results are consistent with prior evidence that China's intercity transportation network exhibits structural stability even following large shocks such as the initial COVID-19 outbreak [8], and suggest that, despite policy differences across periods, the large-scale organisation of intercity mobility remained broadly stable in the observed data.

We then repeated the epidemic risk assessment using the 2023 mobility matrix. This supplementary analysis showed that the main qualitative tier-specific diffusion patterns were preserved. For Omicron, epidemic risk remained highest in super-tier and tier-1 cities and markedly lower in lower-tier cities by day 7, with a clear gradient across the hierarchy; by days 14 and 21, the overall scale of risk increased substantially, but the hierarchical pattern remained evident. The corresponding heatmaps further showed sustained cross-tier dissemination, especially toward tier-3 and tier-2 destination cities, indicating continued downward hierarchical spread from higher-tier origins.

For influenza A, the 2023 analysis again showed a more stratified pattern. Although super-tier and tier-1 origins still generated substantial risk, the overall spread remained more limited than for Omicron, and the destination-tier heatmaps showed a stronger tendency toward within-tier or adjacent-tier spread rather than broad hierarchical penetration across the full urban system. This contrast between Omicron and influenza A was apparent

on days 7, 14, and 21, supporting the conclusion that the more rapid and broader cross-tier dissemination of Omicron is not solely an artefact of the 2021-2022 mobility regime used in the main analysis.

The reviewer also asks how the findings might differ in settings without comparable intervention intensity. We agree that this remains an important consideration. Our additional 2023 analysis suggests that the principal qualitative conclusions are robust to an alternative, post-zero-COVID mobility regime within China. At the same time, we have revised the Discussion to make it clear that the quantitative magnitude and timing of spatial diffusion are likely to depend on the prevailing mobility regime, intervention environment, travel behaviour, and transport system. We therefore do not interpret the China-specific numerical estimates as directly transferable to all settings without recalibration.

Following the reviewer’s suggestion, we now frame China more explicitly as a specific case study and proof-of-concept. In the revised Discussion, we describe China as a large, socioeconomically heterogeneous, tier-structured urban system observed under a distinctive policy regime. In this sense, the present study is intended to demonstrate how a mobility-informed branching-process framework can be used to quantify and compare hierarchical spatial diffusion, rather than to claim universal quantitative generalisability.

We have also clarified the extent to which the framework, rather than the China-specific results, can be transferred to other epidemiological and policy contexts. In our view, the framework is transferable because it requires three ingredients that are available in many settings: city-to-city or area-to-area mobility data supporting the construction of an origin-destination matrix; a functional or socioeconomic stratification of cities that groups units with shared structural characteristics; and pathogen-specific transmission parameters (mean reproductive number, overdispersion, generation time, or serial interval). For example, in the United States, the Brookings Institution's Metro Monitor [9] provides a well-established empirical foundation for deriving a socioeconomic stratification of cities. And commuting flow data can be used to quantify inter-area mobility. In such settings, the same analytical approach could be implemented using locally observed mobility patterns,

locally appropriate urban-system definitions, and pathogen-specific epidemiological parameters. What would need to be adapted are the parameterisation and the operational definition of urban tiers, not the general modelling logic itself.

In response to this comment, we therefore revised the manuscript in three ways. First, we strengthened the Discussion of the policy-conditioned nature of the mobility data used in the main analysis. Second, we added a supplementary robustness analysis based on 2023 post-zero-COVID mobility data, which showed high structural similarity of mobility matrices across observed periods and qualitatively consistent tier-specific diffusion patterns for both Omicron and influenza A. Third, we revised the study's framing to define China more explicitly as a case study/proof-of-concept, while clarifying that the transferable component is the analytical framework rather than the direct portability of China-specific quantitative estimates.

**Supplementary Material (Page 8, Lines 222-235)**

***Robustness to an alternative post-zero-COVID mobility regime***

*To examine whether the main findings were strongly dependent on mobility patterns during China's "dynamic zero-COVID" period, we conducted an additional robustness analysis using intercity mobility data from 2023, after the policy was relaxed. We first constructed mobility matrices for four observed periods: the Nanjing Delta outbreak, the Shanghai Omicron outbreak, the full year 2021, and the full year 2023. Each matrix summarised the proportion of movement from each origin city to each destination city. Structural similarity between matrices was assessed using Pearson correlation.*

*We then repeated the epidemic risk assessment using the 2023 mobility matrix, while keeping the same epidemiological parameter settings as in the main analysis. Simulations were performed separately for SARS-CoV-2 Omicron and influenza A, assuming 100 initial infections and a 21-day simulation window. As in the primary analysis, epidemic risk was summarised across prefecture tiers on days 7, 14, and 21, and hierarchical spread was characterised by the proportion of affected destination cities in each tier, conditional on the tier of outbreak origin.*

**Supplementary Material (Page 12, Lines 377-390)**

***Robustness of tier-specific diffusion patterns under 2023 mobility conditions***

*Intercity mobility matrices were highly similar across the four observed periods (Fig H). Pairwise comparisons showed Pearson correlations of 0.930 to 0.995, indicating that the broad organisation of intercity mobility remained stable across these settings.*

*When the risk assessment was repeated using the 2023 mobility matrix, the main qualitative patterns were preserved (Fig K-M). For Omicron, epidemic risk remained highest in super-tier and tier-1 cities, with broader cross-tier dissemination over time. For influenza A, spread remained more stratified, with less extensive penetration across the urban hierarchy. These differences between pathogens were evident on days 7, 14, and 21.*

*Taken together, these analyses suggest that the principal qualitative conclusions of the study were robust to an alternative post-zero-COVID mobility regime. However, the absolute magnitude of epidemic risk remained context dependent.*

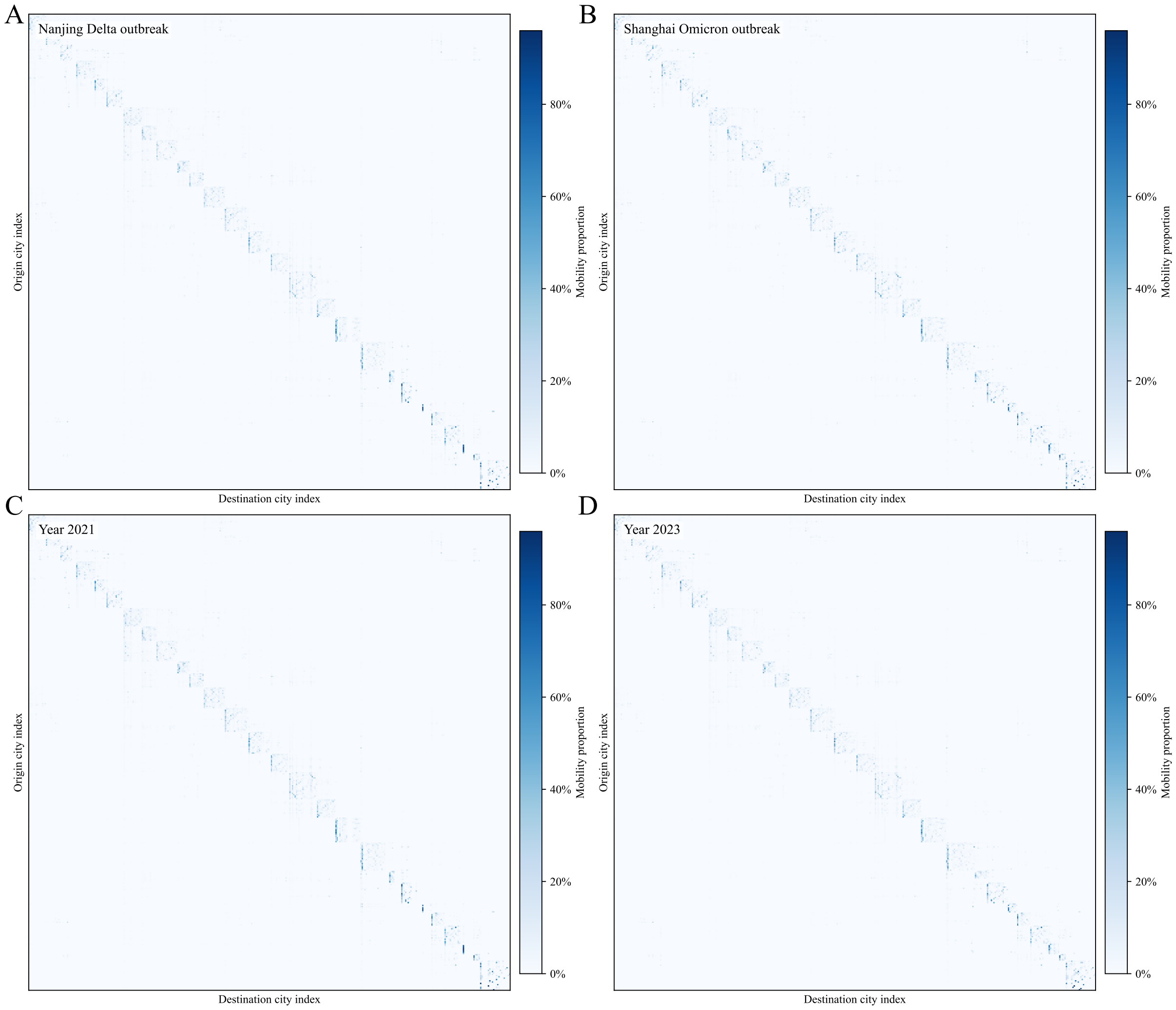


***Fig H. Intercity mobility matrices across four periods.***

*(A-D) Heatmaps show intercity mobility matrices for the Nanjing Delta outbreak, the Shanghai Omicron outbreak, and for 2021 and 2023. Each cell represents the proportion of movement from an origin city to a destination city, with darker colours indicating higher mobility intensity. Pairwise comparisons showed high similarity across matrices, with Pearson correlations ranging from 0.930 to 0.995.*

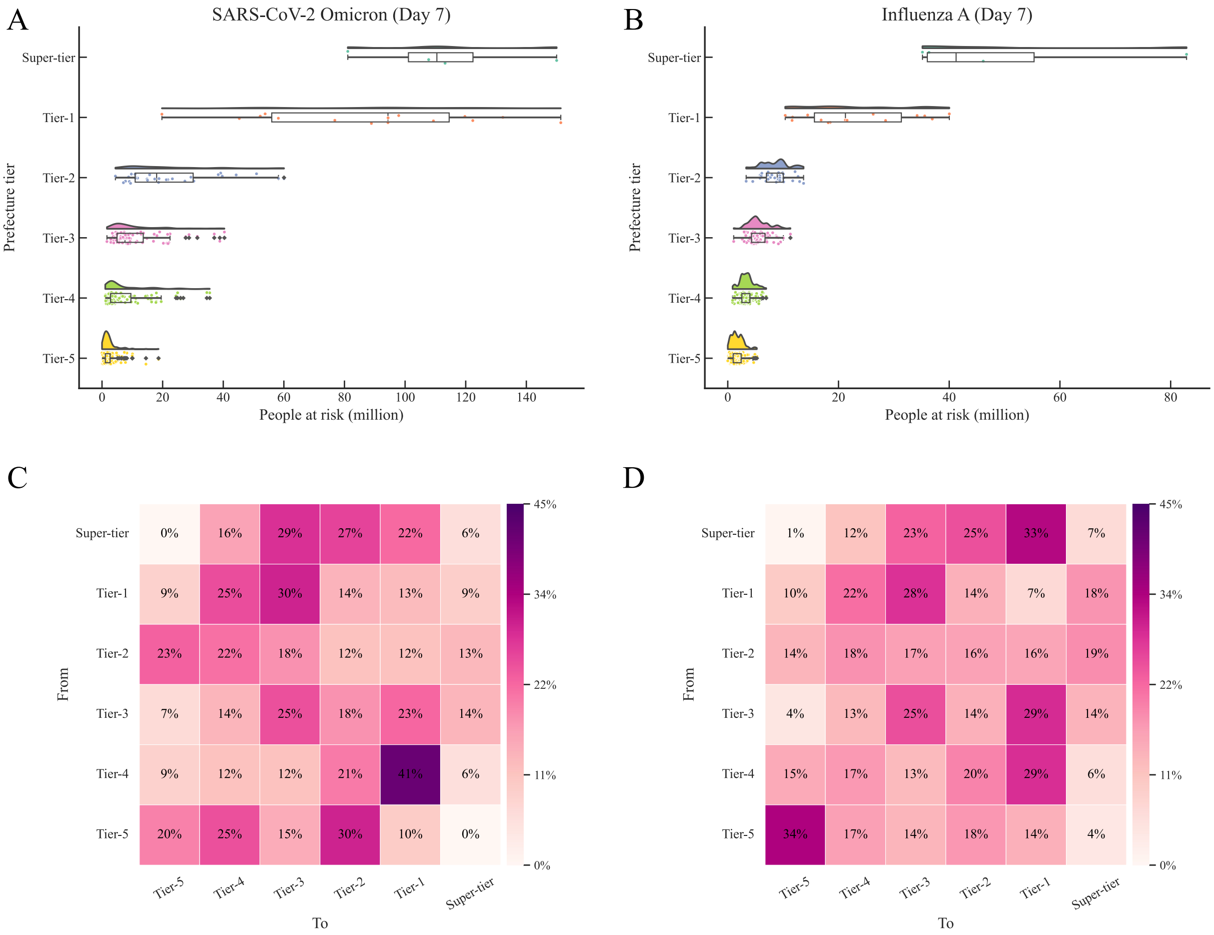


***Fig K. Epidemic risk across prefecture tiers for SARS-CoV-2 and Influenza A transmission on Day 7, based on 2023 population mobility data.***

*(A-B) Raincloud plots show the distribution of epidemic risk across prefecture tiers for SARS-CoV-2 (Omicron) and Influenza A on Day 7, assuming 100 initial cases and a 21-day simulation window. (C-D) Heatmaps depict the proportion of affected cities in each destination tier, conditional on outbreaks originating from source tiers, for SARS-CoV-2 (Omicron) and Influenza A on Day 7. Darker colours indicate a higher likelihood of transmission, illustrating tier-specific patterns of within-tier and cross-tier spread.*

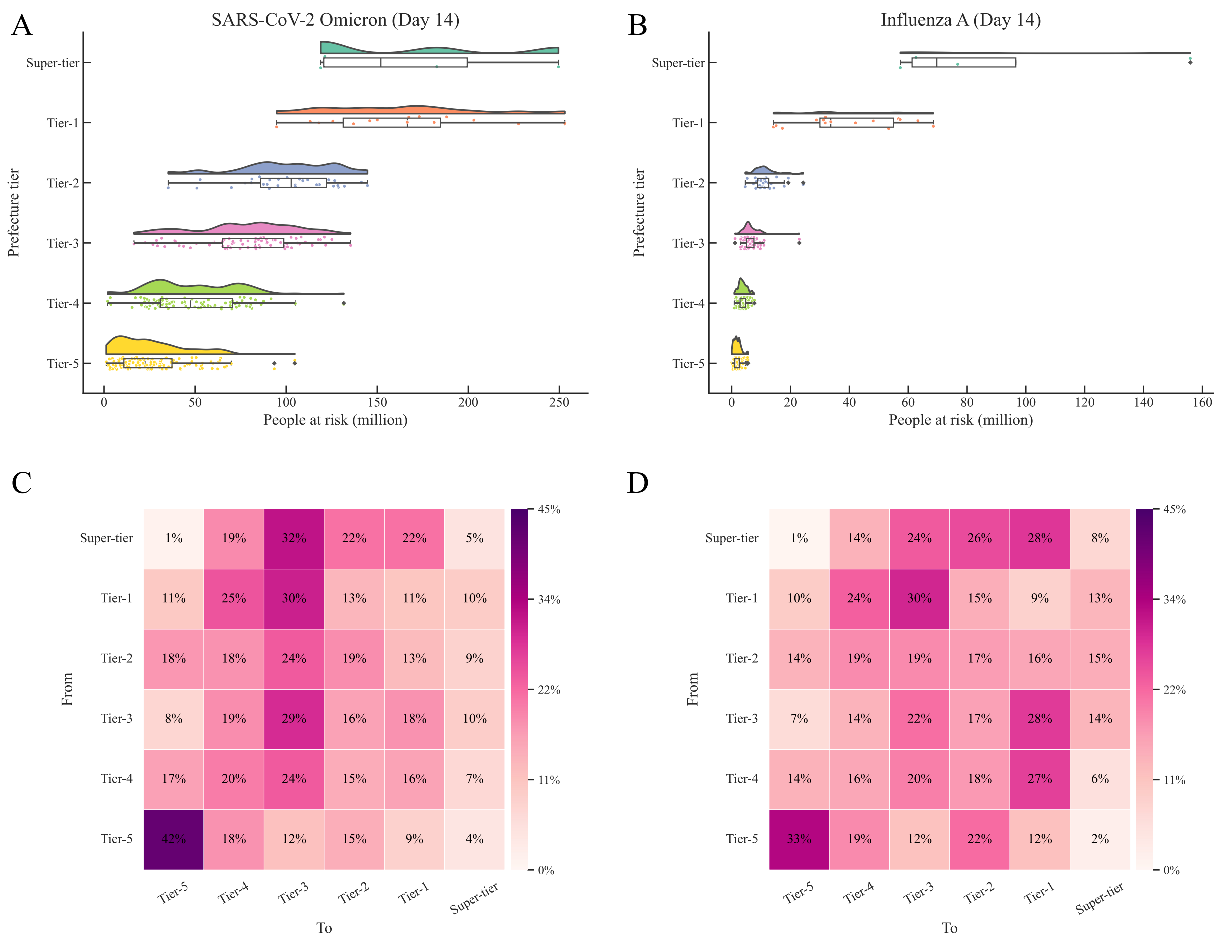


***Fig L. Epidemic risk across prefecture tiers for SARS-CoV-2 and Influenza A transmission on Day 14, based on 2023 population mobility data.***

*(A-B) Raincloud plots show the distribution of epidemic risk across prefecture tiers for SARS-CoV-2 (Omicron) and Influenza A on Day 14, assuming 100 initial cases and a 21-day simulation window. (C-D) Heatmaps depict the proportion of affected cities in each destination tier, conditional on outbreaks originating from source tiers, for SARS-CoV-2 (Omicron) and Influenza A on Day 14. Darker colours indicate a higher likelihood of transmission, illustrating tier-specific patterns of within-tier and cross-tier spread.*

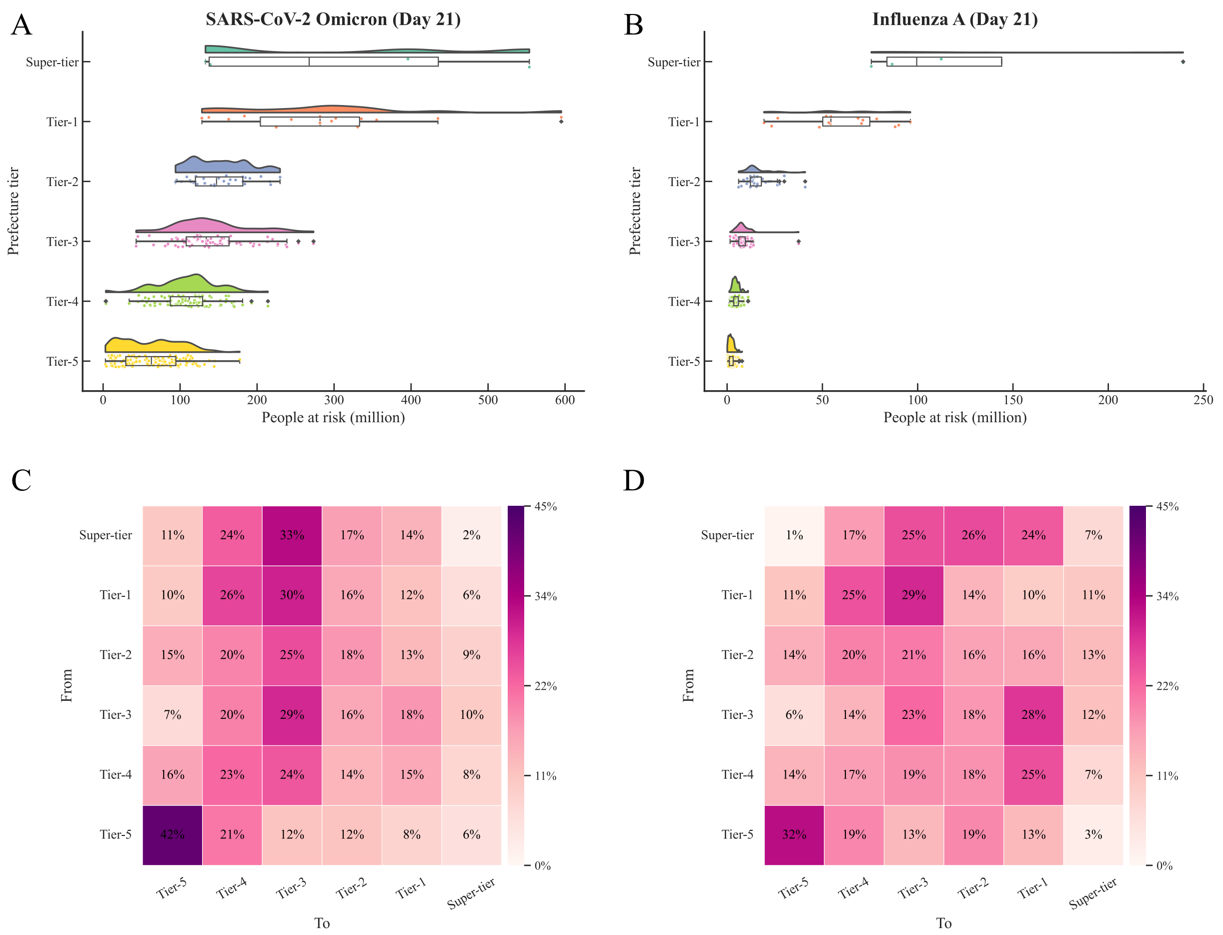


***Fig M. Epidemic risk across prefecture tiers for SARS-CoV-2 and Influenza A transmission on Day 21, based on 2023 population mobility data.***

*(A-B) Raincloud plots show the distribution of epidemic risk across prefecture tiers for SARS-CoV-2 (Omicron) and Influenza A on Day 21, assuming 100 initial cases and a 21-day simulation window. (C-D) Heatmaps depict the proportion of affected cities in each destination tier, conditional on outbreaks originating from source tiers, for SARS-CoV-2 (Omicron) and Influenza A on Day 21. Darker colours indicate a higher likelihood of transmission, illustrating tier-specific patterns of within-tier and cross-tier spread.*

## Discussion (Page 28, Lines 669-683)

*The mobility data used in the main analysis were derived from China's "dynamic zero-COVID" period and therefore reflect a policy-conditioned mobility regime. In supplementary analyses, however, intercity mobility matrices were highly similar across the Nanjing Delta outbreak, the Shanghai Omicron outbreak, the full year*

*2021, and the full year 2023, consistent with prior evidence that China's intercity transportation network exhibits structural stability even following large shocks such as the initial COVID-19 outbreak. And repeating the risk assessment using post-zero-COVID mobility data from 2023 yielded qualitatively consistent results. In particular, Omicron continued to show faster and broader cross-tier dissemination, whereas influenza A remained more stratified within the urban hierarchy. These findings suggest that the main qualitative conclusions are not solely specific to the mobility conditions of 2021 to 2022. We therefore interpret China as a large-scale case study and the present analysis as a proof-of-concept for a transferable framework, rather than as implying direct quantitative generalisability of the China-specific estimates.*

2. Model validation beyond first arrival times

   Model validation focuses primarily on correlations between predicted and observed first arrival times. While this metric may be relevant for early spatial diffusion, arrival-time ordering alone does not fully capture outbreak dynamics or public-health impact. The authors should justify why first arrival time was prioritised over alternative or complementary validation targets, such as the cumulative number of affected cities, the temporal growth of spatial spread, or measures more directly linked to intervention decision-making.

   **Response**: We thank the reviewer for this constructive comment. First-arrival time remains our primary validation target because it directly defines the actionable window for early detection and intervention before local transmission becomes established. We fully agree, however, that complementary metrics enrich the assessment of model performance along other dimensions, and we have extended the validation framework accordingly to include the two targets suggested by the reviewer: the cumulative number of affected cities and the temporal growth of spatial spread.

   The first arrival time was chosen as the primary validation target for reasons related to the policy frame our evidence is intended for, regional rather than national policymakers, in the context of regional movement restriction as a public health intervention. In China, policies for respiratory epidemics are enacted at the city level rather than the national

level, given the size of the population. Public health interventions are often initiated in response to a trigger. For local public health practitioners, it is therefore more valuable to know "has the outbreak propagated towards my city" than "what proportion of the country is now affected" or "how fast is the outbreak spreading spatially." This local signal, whether an outbreak has arrived, is the operationally meaningful metric when the decision being modelled is a city-level response trigger, not a national resource allocation or a macro epidemic trajectory.

We compared the observed and simulated cumulative number of affected cities within 30 days of the Shanghai outbreak. There were 65 cities observed to have been affected, while our model predicted a mean cumulative total of 60.4 affected cities at day 30 (95% simulation interval: 21.0-99.0). To assess the temporal growth of spatial spread, we compared the observed and simulated cumulative number of affected cities over time across the 30-day observation window, as shown in the supplementary figure. The growth curves show that the model captures the general trajectory of spatial expansion, with the simulated fitted curve tracking the observed fitted curve across the observation period. To formally compare the two distributions, we conducted a non-parametric test comparing the observed mean cumulative number of affected cities across days (23.77) against the distribution of simulated means (mean 28.90, 95% CI: 10.10-53.03). The two-sided empirical p-value was 0.72, indicating no statistically significant difference between the observed and simulated temporal growth patterns. The observed value falls at the 36th percentile of the simulated distribution, well within the range of stochastic outcomes produced by the model.

These results extend the validation evidence beyond the first arrival time ordering in two important respects. First, the model reproduces not only the sequence in which cities were affected but also the outbreak's total geographic footprint within 30 days, with the observed cumulative count of 65 cities comfortably falling within the simulated 95% interval. Second, the temporal trajectory of spatial spread, the rate at which new cities were reached over time, is statistically indistinguishable from the observed trajectory under a non-parametric comparison. Together with the first arrival time correlations reported in the main analysis, these results indicate that the model captures multiple

complementary dimensions of spatial diffusion dynamics rather than optimising performance on a single metric. These analyses are described in full in Supplementary Methods, and the corresponding results are reported in Supplementary Results.

**Supplementary Material (Page 5, Lines 99-112)**

***Validation against cumulative affected cities and the temporal growth of the spatial spread***

*To extend model validation beyond the first arrival time ordering, we assessed two complementary targets for the Shanghai outbreak: the total cumulative number of cities affected within a 30-day observation window, and the day-by-day temporal trajectory of cumulative spatial spread. A city was classified as affected on the day it first received at least one imported infection in a given simulation replicate. For each of the 10,000 simulation replicates, we computed the cumulative number of affected cities from day 1 to day 30. We summarised the simulated distribution on each day as the mean and 95% simulation interval across replicates. The total cumulative count at day 30 was summarised across replicates as the mean and 95% simulation interval. The temporal growth trajectories of the observed and mean simulated cumulative affected cities were visualised separately from the observed and mean simulated counts across days. To formally compare the observed and simulated temporal growth patterns, we computed the mean cumulative number of affected cities over all 30 days for the observed data and for each simulation replicate. The observed mean was then compared against the distribution of simulated means using a non-parametric test.*

**Supplementary Material (Page 12, Lines 392-405)**

***Validation against cumulative affected cities and the temporal growth of the spatial spread***

*We compared the observed and simulated cumulative number of affected cities within 30 days of the Shanghai outbreak. There were 65 cities observed to have been affected, while our model predicted a mean cumulative total of 60.4 affected cities at day 30 (95% simulation interval: 21.0-99.0). The temporal growth trajectories of cumulative affected cities are shown in Supplementary Fig C: the*

simulated fitted curve closely tracks the observed fitted curve across the 30-day window, with both trajectories showing an initial phase of rapid spatial expansion followed by deceleration as the pool of reachable cities within the mobility network becomes increasingly saturated. To formally compare the temporal growth patterns, we computed the mean cumulative number of affected cities across all 30 days for the observed data (23.77) and for each of the 10,000 simulation replicates. The mean of simulated means was 28.90 (95% simulation interval: 10.10-53.03), and the observed mean fell at the 36th percentile of the simulated distribution. A non-parametric comparison found no statistically significant difference between the observed and simulated temporal growth distributions (two-sided $p = 0.72$), indicating that the model-predicted trajectory of spatial expansion is consistent with the observed data.*

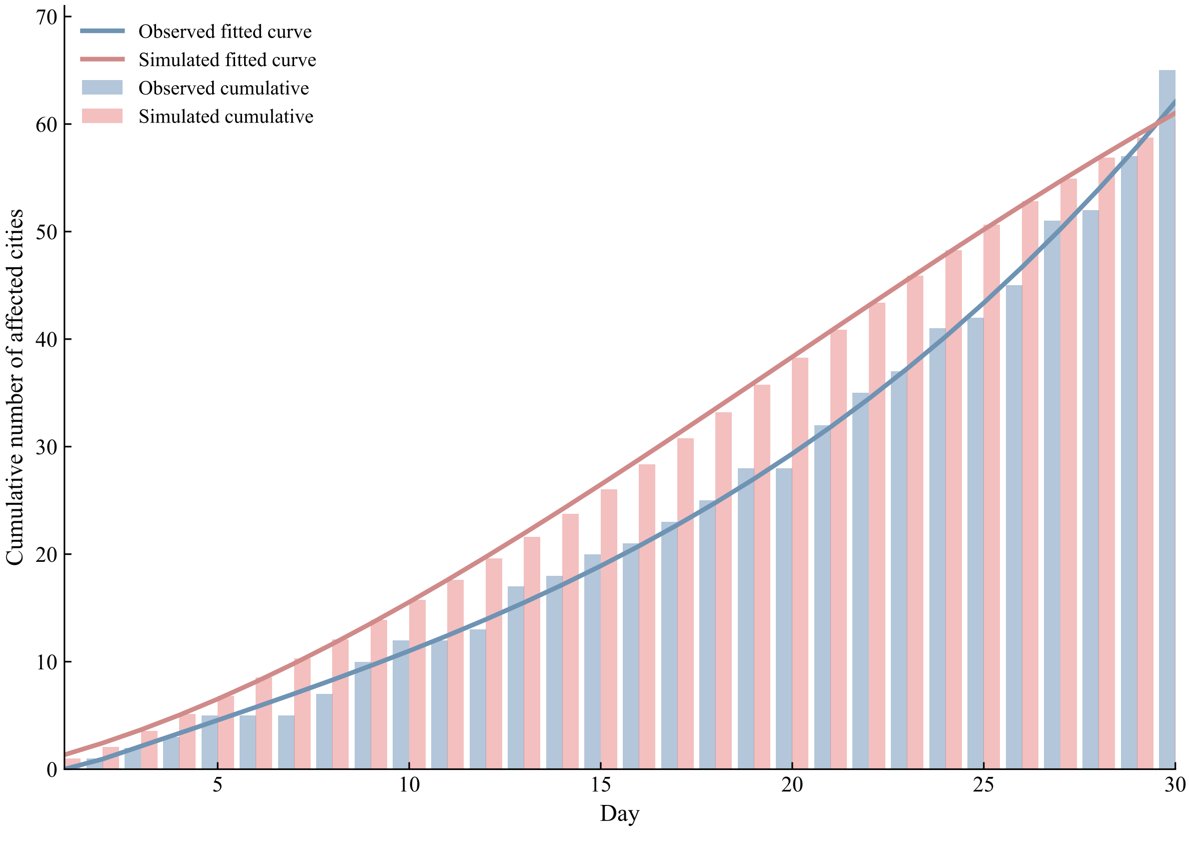


***Fig C. Cumulative number of cities affected by the Shanghai outbreak over the 30-day observation window.***

*The blue bars represent the observed daily cumulative count of affected cities, and the blue fitted curve shows the observed growth trajectory. The red bars represent*

*the mean simulated cumulative count of affected cities across 10,000 stochastic replicates at each day, and the red fitted curve shows the corresponding simulated growth trajectory. A city was counted as affected on the day it first received at least one imported infection in the simulation, and was included in the analysis if it met the 5% simulation-frequency inclusion threshold. The close agreement between the observed and simulated fitted curves across the observation period, together with the non-parametric comparison (two-sided p = 0.72), indicates that the model reproduces the temporal trajectory of spatial expansion without systematic bias.*

**Minor Comments:**

1. Lines 93-94: The statement "In countries with substantial socioeconomic heterogeneity and hierarchical urban systems" is vague. Please specify which countries or settings are being referred to, beyond China.

   **Response**: We thank the reviewer for highlighting this imprecision. The original phrasing invoked two conceptually distinct properties, socioeconomic heterogeneity among cities and the presence of a structured urban hierarchy, without naming comparator settings or identifying classification schemes that operationalise these properties. The revised sentence addresses both. The Yicai ranking used in the present study assesses Chinese cities at the prefectural level and above on five dimensions, namely concentration of commercial resources, urban hub function, urban resident activity, new economy competitiveness, and future potential, producing an ordering that reflects socioeconomic development, commercial maturity, and connectivity rather than population size or administrative type alone. Analogous classifications constructed on socioeconomic criteria, rather than on administrative boundaries or commuting integration alone, are available for several other settings, and a non-exhaustive list is given below together with representative applications in health or epidemiological research.

   In Brazil, the Brazilian Institute of Geography and Statistics (IBGE) conducts the Regiões de Influência das Cidades (REGIC) survey, most recently in 2018, which classifies Brazilian cities into a hierarchy reflecting socioeconomic centrality: Metropolises, Regional Capitals, Sub-regional Centres, Zonal Centres, and Local Centres[10]. Unlike administrative

definitions of metropolitan areas, REGIC classifies cities according to the breadth and intensity of their socioeconomic influence over surrounding regions, indicated by goods and service flows, commercial activity, and institutional presence. Lowe and colleagues used the REGIC hierarchy as a proxy for human movement to model the expansion of dengue across 21st-century Brazil, demonstrating that the socioeconomic hierarchy captures epidemiologically relevant connectivity beyond geographic distance [11].

In Indonesia, Statistics Indonesia (Badan Pusat Statistik, BPS) classifies regencies and cities by development status using the Human Development Index, which combines life expectancy, education, and income components. The scheme distinguishes very high (HDI $\geq$ 80), high (70 $\leq$ HDI < 80), medium (60 $\leq$ HDI < 70), and low (HDI < 60) categories, and the data are published annually for all 514 regencies and cities [12]. This classification produces an explicit socioeconomic stratification of Indonesian urban units, typically concentrating very high HDI status in major municipalities such as Surabaya and Medan. At the same time, rural and peripheral regencies remain in lower tiers.

In India, the Ministry of Finance classifies cities into three tiers under the House Rent Allowance scheme, designated X, Y, and Z (commonly referred to as Tier 1, Tier 2, and Tier 3), based on population size and cost-of-living indicators following the recommendations of the Seventh Central Pay Commission [13]. Although originally designed for administrative wage-setting, the scheme functions as a widely used proxy for socioeconomic stratification in research, reflecting systematic differences in economic development, infrastructure, and service capacity across the urban hierarchy. It’s a logic directly analogous to the Yicai classification used here. The scheme has been explicitly applied in epidemiological research: Vasudevan et al. (2022) used the Tier 1 and Tier 2 classification to compare air quality changes across Indian cities during COVID-19 lockdowns, demonstrating that tier membership predicted broadly similar patterns of urban responsiveness to public health restriction[14], and Sunilkumar et al. (2023) used Tier 1 cities as the primary analytical unit when examining the relationship between air pollutant concentrations and COVID-19 infection rates[15].

In the United States, the Brookings Institution's Metro Monitor[9] provides a well-established empirical foundation for deriving such a stratification. Published annually since 2015, the Metro Monitor assesses approximately 196 metropolitan areas with populations above 250,000 residents across composite dimensions of growth, prosperity, and inclusion, assigning each metropolitan area a composite score and a rank within its size class. Although the Metro Monitor does not assign metropolitan areas to fixed named tiers, discrete performance categories can be derived by applying quantile or cluster-based divisions to the composite scores, a methodologically straightforward step that would produce a socioeconomically grounded urban stratification broadly analogous in logic to the Yicai classification used here, in that cities are ordered by structural socioeconomic position rather than by administrative boundaries or population size alone.

We have revised the sentence at lines 118-124 to name comparator settings and cite the classification schemes that capture socioeconomic heterogeneity in each.

**Introduction (Page 6, Lines 118-124):**

> *In countries with substantial socioeconomic heterogeneity and hierarchical urban systems, as characterised, for example, by Brazil's IBGE Regiões de Influência das Cidades[19], Indonesia's BPS Human Development Index-based classification[20], India's Ministry of Finance tier classification[21], and the United States' Brookings Metro Monitor rankings[22], integrating mobility-informed risk assessment into epidemic preparedness frameworks provides a practical pathway toward achieving precision and efficiency in outbreak response.*

2. Line 112: Please provide a clearer explanation of the migration index. Are comparable mobility indices available in other countries, and if so, could this framework be realistically implemented elsewhere?

   **<u>Response</u>**: We thank the reviewer for raising this point. The original manuscript introduced the Baidu migration index without adequately describing its data sources, construction, or interpretation, and without addressing whether analogous indices exist in other settings. We have revised line 112 and the associated Methods subsection to clarify these points.

The Baidu migration index (Baidu Qianxi) is derived from anonymised location data generated by over 1.1 billion active users of Baidu's map and location-based services in mainland China, which together account for approximately 80% of the smartphone map-service market in the country. Two complementary components are used in the present study. The first is a daily city-level migration scale index, which represents the relative intensity of outbound travel from each origin city on each day, normalised against a platform-wide baseline. The second is a daily directional outflow proportion, which specifies the share of each city's total outbound travel that is directed to each of the other cities. Combining these two components yields directed daily city-to-city mobility flows for all prefecture-level units in mainland China, which we then averaged over the outbreak period and row-normalised to construct the origin-destination transition matrices used in the model.

Functionally comparable mobility data products capable of supporting origin-destination matrix construction are available in several other settings. In the United States, SafeGraph has been used to compute dynamic daily and weekly county-to-county and state-to-state population flow matrices [16], and Cuebiq has been used alongside other platforms to build mobility matrices for epidemic modelling [17]. In Europe, Meta's Data for Good programme publishes a Movement Between Tiles dataset that records directed inter-tile population flows and has been used to characterise multiscale mobility patterns during the COVID-19 pandemic [18]. In sub-Saharan Africa, call detail records from mobile network operators have been used to construct district-level origin-destination matrices [19]. These products differ in their spatial resolution, population coverage, and temporal granularity. Still, all provide the directed mobility flow information required to construct the origin-destination transition matrices used in our framework.

Implementation of the framework in other settings, therefore, does not require access to the Baidu platform specifically. It requires a mobility data source to construct a directed origin-destination matrix among the urban units of interest, a functional or socioeconomic stratification of those units, and pathogen-specific transmission parameters. Where these inputs are available, the analytical logic transfers directly. However, numerical estimates would reflect the specific mobility regime and urban system of each setting and should

not be assumed to replicate those reported here. We have revised the Supplementary to include this clarification and to point readers to the relevant data sources for several comparator settings.

**Supplementary Material (Page 3, Lines 32-50)**

*Human mobility was quantified using the Baidu Migration Index, which is derived from anonymised location-based service signals from over 1.1 billion mobile devices. We obtained both the migration scale index for each city and the proportion of outflow directed to each destination city from the Baidu Qianxi platform. These data were used to characterise intercity mobility as follows:*

$$M_{ij,t} = Q_{i,t,out} \times x_{ij,t} \tag{1}$$

$$M_{ij} = \frac{\left(\sum_{t=1}^{T} M_{ij,t}\right)}{T} \tag{2}$$

$$p_{M_{ij}} = \frac{M_{ij}}{\sum_j M_{ij}} \tag{3}$$

*where $Q_{i,t,out}$ denotes the migration scale index representing the total outflow from city $i$ at time $t$, and $x_{ij,t}$ denotes the proportion of that outflow travelling from city $i$ to city $j$. The quantity $M_{ij}$ therefore represents the average outflow volume from city $i$ to city $j$ over the period $T$, and $p_{M_{ij}}$ is the corresponding probability of travel. Using these Baidu Qianxi data, we constructed 366×366 transition matrices for each COVID-19 outbreak scenario, corresponding to the relevant time periods.*

*Functionally comparable mobility data products are available in other settings, including Meta's Movement Between Tiles dataset, aggregated mobility data from SafeGraph and Cuebiq, and mobile-network-operator origin-destination matrices. They would support the implementation of the same analytical framework with appropriate local adaptation.*

3. Line 124: Please spell out the abbreviation PHSM at first use.

**Response**: Thank you for noting this omission. We have now spelt out PHSM at first use as "public health and social measures".

**Methods (Page 8, Lines 174-176)**

*In the context of this study, we define the start of an outbreak as the detection of the first case, and the end of an outbreak as the last confirmed case or the introduction of substantial public health and social measures (PHSM).*

4. Line 136: The serial interval is used in the model—please clarify whether this is being treated as a proxy for the generation time, and discuss any implications.
**Response**: We thank the reviewer for raising this point, which allows us to clarify an aspect of the model specification that we agree deserves an explicit statement.

The serial interval (SI) in our model is not used as a proxy for the generation time (GT). Rather, the use of the SI is a deliberate choice that aligns with the nature of the empirical data against which the model is validated.

Our validation quantity is the first arrival time in each city, defined as the interval between the first reported/observed case in the origin city and the first reported/observed case in the destination city. These arrival times are therefore derived from case-detection (symptom-onset/reporting) timestamps, not from unobservable infection timestamps. Infection times (and hence generation time intervals) were not directly observed in any of the three validation outbreaks (Shanghai-Omicron, Nanjing-Delta, and the northwestern Delta outbreak).

The SI describes the interval between symptom onsets of successive cases in a transmission chain, whereas the GT describes the interval between successive infection events. Because the observed first arrival time in a destination city is indexed to the detection of the first case there (not to the unobserved infection event that seeded the city), the simulated inter-case interval that enters our model must also be indexed to detection/onset events to remain on the same time scale as the observations. Using the SI ensures this alignment between the simulated and observed quantities. Using a GT-

based interval, by contrast, would yield simulated infection times that would need to be convolved with an incubation period and a reporting delay before being comparable to the observed detection-based arrival times, introducing additional unobservable parameters and a mismatched reference time.

Assuming the distribution of incubation periods is approximately stationary across cities and outbreaks, the SI provides an empirically observable and internally consistent temporal scale for inter-city transmission within our framework. We acknowledge, however, that (i) the SI can be biased by non-pharmaceutical interventions, case ascertainment practices, and right-truncation, particularly in the early phase of an outbreak, and (ii) the variance of the SI is generally larger than that of the GT because it incorporates incubation-period variation on both sides of a transmission pair. For these reasons, we did not attempt to recover infection-time dynamics; instead, we restricted the comparison to detection-based first arrival times, which is the quantity the SI directly parameterises. The joint probabilistic uncertainty analysis (now referenced in the revised Supplementary methods and results) further shows that our spatial risk patterns are robust to plausible variation in the SI distribution.

To make this reasoning explicit for readers, we have added the following clarification to the Supplementary methods. **(Page 4, Lines 75-82).**

*We emphasise that the serial interval is used here because our validation target, the first arrival time in each city, is derived from the timing of the first detected case (i.e., based on symptom onset/reporting), not from the timing of infection. The serial interval, which describes the interval between successive symptom onsets in a transmission chain, is therefore the natural and directly observable temporal scale on which simulated and observed arrival times can be compared. The serial interval is not used as a proxy for the generation time; generation intervals, being infection-to-infection, are not directly observable in routine surveillance data and would introduce additional unobservable parameters (incubation and reporting delays) if used in place of the serial interval.*

5. Line 205: Please explain why Spearman correlation was used instead of Pearson correlation in this analysis.

   **Response**: We thank the reviewer for this question. Line 205 refers to the geodesic distance analysis, in which we assess whether raw geographic distance alone can reproduce the observed ordering of first arrival times.

   Spearman correlation was used for the geodesic distance for two reasons. First, the functional form of the relationship between geographic distance and first-arrival time under SARS-CoV-2 transmission is unknown a priori. Geographic distance and arrival time may be monotonically related without following any specific parametric form, and Spearman correlation is appropriate precisely when one wishes to assess whether a monotonic association exists without imposing assumptions about its functional shape. Second, geodesic distances and first arrival times are both right-skewed distributions. We conducted the Henze-Zirkler multivariate normality test on the joint distribution of observed first arrival time and geodesic distance for both outbreaks. The assumption of bivariate normality was rejected for both the Shanghai outbreak (HZ statistic = 3.59, $p<0.001$) and the Nanjing outbreak (HZ statistic = 0.85, $p = 0.03$). Spearman correlation is more robust to the influence of extreme values and departures from bivariate normality than Pearson correlation. The scatter plot of observed first arrival time against geodesic distance, now provided as a supplementary figure, makes the empirical motivation for Spearman correlation clear.

   We have revised the Supplementary Methods section accordingly.

   **Supplementary Material (Page 7, Lines 180-194)**

   ***Spearman correlation in geodesic distance analysis***

   *To assess whether Pearson or Spearman correlation was more appropriate for the geodesic distance analysis, we examined the distributional properties of the variables and the functional form of their relationship (Fig E). The marginal distributions of both observed first arrival time and geodesic distance were right-skewed. To formally evaluate the bivariate normality assumption underlying Pearson correlation, we conducted the Henze-Zirkler multivariate normality test on the joint distribution of observed first arrival time and geodesic distance for each*

*outbreak separately. Bivariate normality was rejected for both the Shanghai outbreak (HZ statistic = 3.59, $p < 0.001$) and the Nanjing outbreak (HZ statistic = 0.85, $p = 0.03$). The scatter plot of observed first arrival time against geodesic distance further confirms that the relationship between the two variables is at most weakly monotonic and does not conform to any consistent parametric functional form. Additionally, the functional form of the relationship between geographic distance and first arrival time during SARS-CoV-2 transmission is unknown a priori. Taken together, these considerations support the use of Spearman rank correlation for geodesic distance analysis, as it assesses monotonic association without requiring bivariate normality, without imposing assumptions about functional form, and with greater robustness to extreme values than Pearson correlation.*

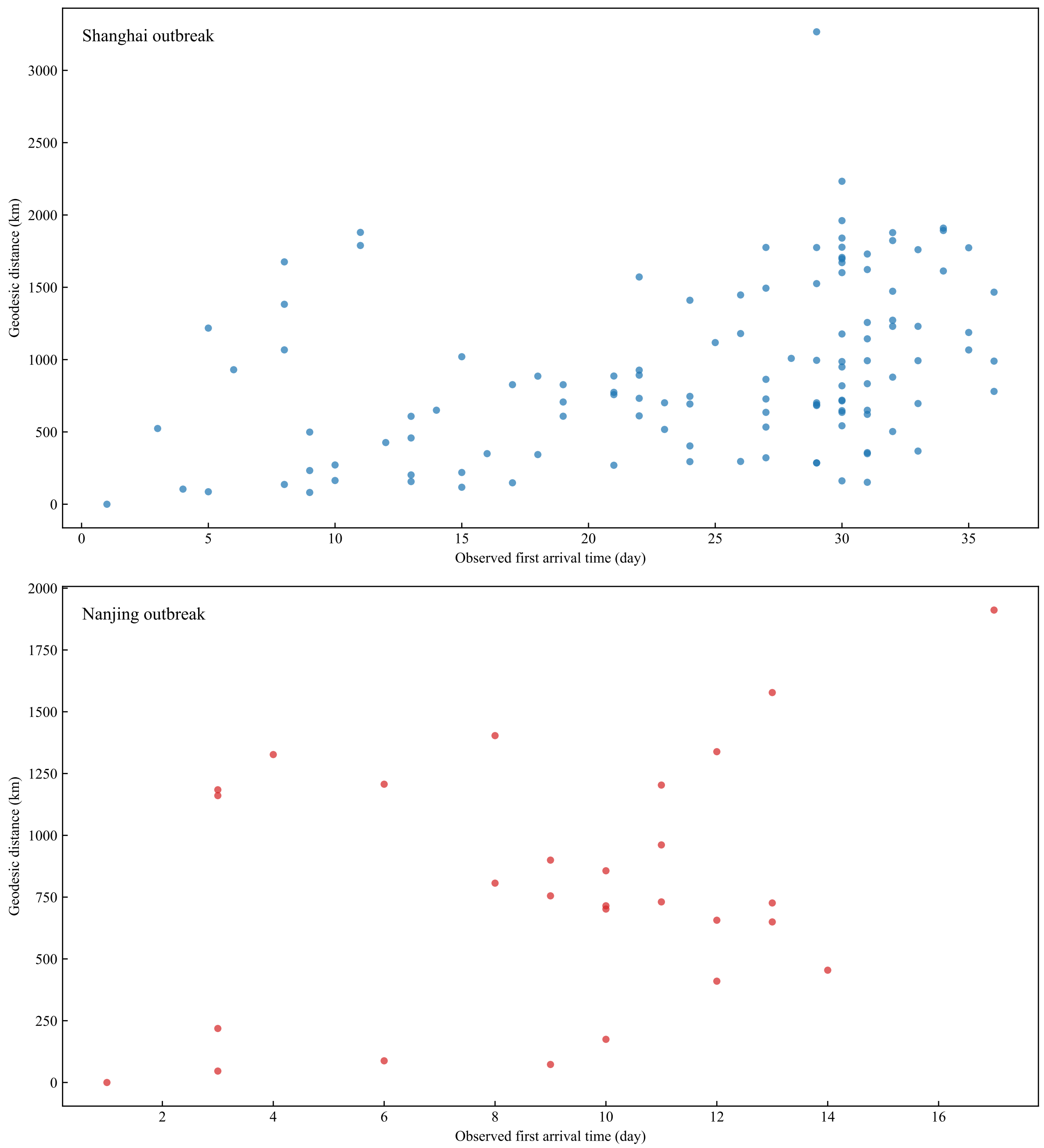


**Fig E. Scatter plot of observed first arrival time and geodesic distance from Shanghai and Nanjing outbreaks.**

Scatter plots of observed first arrival time against geodesic distance from the outbreak origin for cities affected by the Shanghai outbreak (upper panel, blue) and the Nanjing outbreak (lower panel, red). Each point represents one affected city.

**Reviewer 3**

**Major Comments:**

1. The agent-based branching process model is central to the analysis, yet several key implementation details required for reproducibility remain insufficiently specified. In particular, the manuscript does not clearly report the total number of stochastic simulations per scenario, whether results are based on means, medians, or specific quantiles of the simulated distributions, nor how Monte Carlo uncertainty was propagated into the reported outcomes. Given the stochastic nature of the model, this information is essential.

   **Response**: Thank you for this important comment. We have revised the Methods subsection "Simulated first arrival time" to clarify three points requested by the reviewer: the number of stochastic simulations run for each outbreak-origin scenario, how Monte Carlo variability was handled, and how the simulated results were used in the validation analyses.

   Specifically, we now state that each outbreak-origin scenario was simulated 10,000 times, that cities not seeded within the simulation horizon were classified as not reached, and that the predicted first arrival time for each city in the main analysis was summarised as the mean across simulations, which provides a stable estimate of expected arrival timing under stochastic variability. We also clarify that the correlations shown in Fig 2C-D and the additional validation metrics, including MAE and RMSE, were all calculated by comparing the observed first arrival times with these mean predicted first arrival times. We note that the primary objective of the validation is to assess expected arrival timing rather than to estimate uncertainty intervals; however, variability across simulations is reflected in replicate-level outputs and was examined in supplementary analyses to confirm robustness. **(Page 5, Lines 84-97)**.

   > *Each outbreak-origin scenario was simulated 10,000 times to account for stochastic variation in transmission and travel processes. For each simulation, we recorded the first arrival time for every city that was seeded within the simulation horizon. Cities that were not seeded during the simulation period were classified as not reached. For each city, the predicted first arrival time used in the main analysis was*

*summarised as the mean across simulations, which provides a stable estimate of expected arrival timing under stochastic variability. Model performance was evaluated by comparing the observed first arrival times with the mean predicted first arrival times using Pearson correlation, mean absolute error (MAE), and root mean square error (RMSE).*

We have revised the Methods subsection "Risk potential assessment" to clarify how the simulation results were propagated into the reported risk metric. We now specify that the risk metric for each origin city was estimated as the mean across 10,000 stochastic simulations, and that destination cities not seeded within the simulation horizon in a given simulation were excluded from the corresponding risk calculation. **(Page 14, Lines 325-328)**.

*For each origin city, the risk metric* $N_i^{risk}$ *was estimated as the mean across 10,000 stochastic simulations. Destination cities that were not seeded within the simulation horizon in a given simulation were not counted as contributing to transmission risk in that replicate.*

We have also expanded the Supplementary Material to provide additional implementation details to improve reproducibility. In particular, we now describe the replicate-level recording of first arrival times, the treatment of cities not seeded during the simulation period as not reached, and the use of the mean across simulations for both validation and risk assessment. Variability across simulations is captured through these replicate-level outputs and was examined in supplementary analyses to confirm that the results are robust to stochastic variation.

**Supplementary Material (Page 5, Lines 85-97)**

*To quantify stochastic variability, each outbreak-origin scenario was repeated 10,000 times. In each replicate, we tracked whether each destination city was seeded within the simulation horizon and, if so, recorded its first arrival time. Cities not seeded during the simulation period were treated as not reached at the replicate level. For the main validation analyses, the predicted first arrival time of each city was summarised as the arithmetic mean across simulations as the primary estimate of expected arrival timing. MAE, RMSE, and correlation*

*coefficients were then computed by comparing these mean predicted arrival times with the observed first arrival times. For risk assessment, $N_i^{risk}$ was likewise summarised as the mean across simulations, and cities not seeded in a given replicate were not counted as contributing to transmission risk in that replicate.*

2. The decision to omit pathogen-specific parameters (i.e., reproduction number and serial interval) when simulating first arrival times for validation purposes requires stronger justification. While the intention to isolate mobility-driven diffusion is understandable, this assumption implicitly treats all infections as temporally equivalent across pathogens and outbreaks. The potential bias introduced by ignoring heterogeneity in incubation and serial intervals—especially when comparing Omicron and Delta outbreaks—should be more explicitly discussed and, ideally, assessed via sensitivity analysis.

**Response**: We thank the reviewer for this important comment. We agree that the decision to omit pathogen-specific parameters in the original validation analysis required stronger justification. Our primary objective in the original validation was to evaluate the broad spatial ordering of first-arrival times, independent of outbreak-specific transmission dynamics. However, as the reviewer points out, ignoring heterogeneity in reproduction number and serial interval may introduce bias, particularly when comparing outbreaks caused by different variants such as Omicron and Delta.

In response, we performed additional sensitivity analyses that incorporated pathogen-specific transmission parameters into the first-arrival simulations for the Shanghai Omicron and Nanjing Delta outbreaks. We repeated the origin-identification analysis within this extended framework.

**Methods (Page 12, Line 278)**

Table 1. Parameter values for the simulation model

| **Pathogen/ parameter** | **Value used** | **References** |
|---|---|---|
| **SARS-CoV-2 (Omicron BA.2)** | | |
| $R_e$ (Effective reproduction number) | Negative binomial distribution<br>mean = 3.40, overdispersion ($k$ = 0.33) | Cai, J. et al [20]; Guo, Z. et al [21] |
| Serial interval | Gamma distribution<br>mean = 2.72 days, shape = 3.25, scale = 0.84 | Mefsin, Y.M. et al [22] |
| **SARS-CoV-2 (Delta)** | | |
| $R_e$ (Effective reproduction number) | Negative binomial distribution<br>mean = 3.20, overdispersion ($k$ = 0.85) | Zhang, M. et al [23]; Ryu S. et al [24] |
| Serial interval | Gamma distribution | Xu, X. et al [25] |

| | | |
|---|---|---|
| | mean = 3.59 days, shape = 2.06, scale = 1.74 | |

The results showed that the overall agreement between observed and predicted first arrival times remained strong. For the Shanghai outbreak, the Pearson correlation between observed and predicted first arrival times was 0.66 (p<0.001), with an MAE of 6.28 days and an RMSE of 7.99 days. For the Nanjing outbreak, the corresponding Pearson correlation was 0.86 (p<0.001), with an MAE of 1.93 days and an RMSE of 2.56 days. These results indicate that the main validation conclusions were robust after incorporating pathogen-specific reproduction number and serial interval assumptions. This suggests that mobility structure plays a major role in determining the relative ordering of intercity arrival times. At the same time, pathogen-specific parameters primarily affect the temporal scale rather than the spatial pattern of diffusion.

**Supplementary Figure**

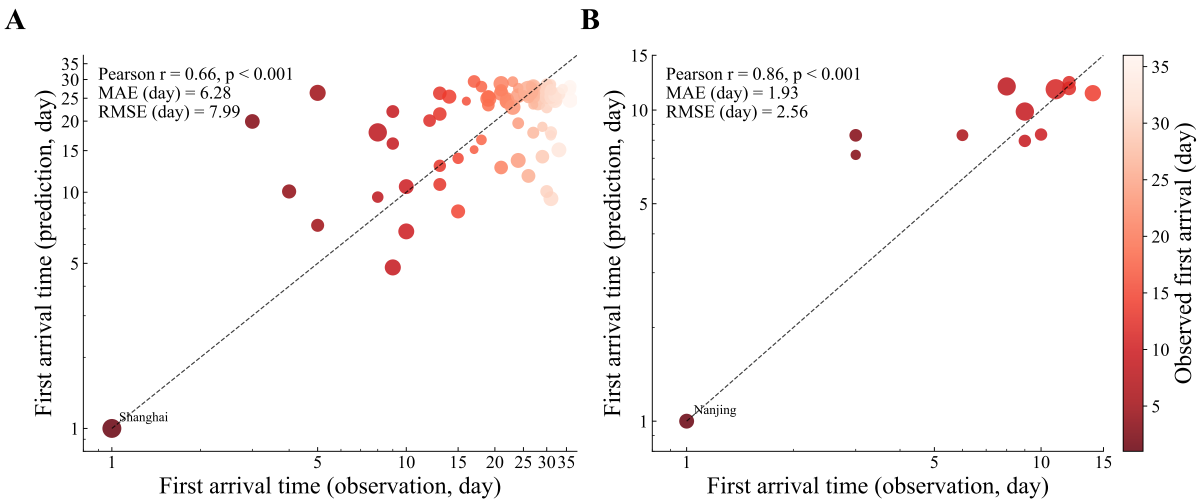


***Fig A. Extended validation of first arrival-time predictions incorporating pathogen-specific reproduction numbers and serial intervals.***

*(A) Comparison between observed and mean predicted first arrival times for cities affected by the Shanghai outbreak, based on simulations incorporating outbreak-specific reproduction number and serial interval values. (B) Comparison between observed and mean predicted first arrival times for cities affected by the Nanjing outbreak, based on simulations incorporating outbreak-specific reproduction number and serial interval values. Each dot represents a city, and the symbol size reflects its population. The dashed line indicates the 1:1 line. For the Shanghai outbreak, the Pearson correlation was 0.66 (p<0.001), with a mean absolute error*

*(MAE) of 6.28 days and a root mean squared error (RMSE) of 7.99 days. For the Nanjing outbreak, the corresponding values were 0.86 (p<0.001), 1.93 days, and 2.56 days, respectively.*

We also repeated the origin-identification analysis with pathogen-specific parameters. The origin-identification results were broadly consistent with the main analysis in these supplementary analyses, indicating that the framework's ability to identify likely origins was not driven solely by the simplifying assumption of temporally equivalent infections across outbreaks, but remained stable under pathogen-specific transmission dynamics.

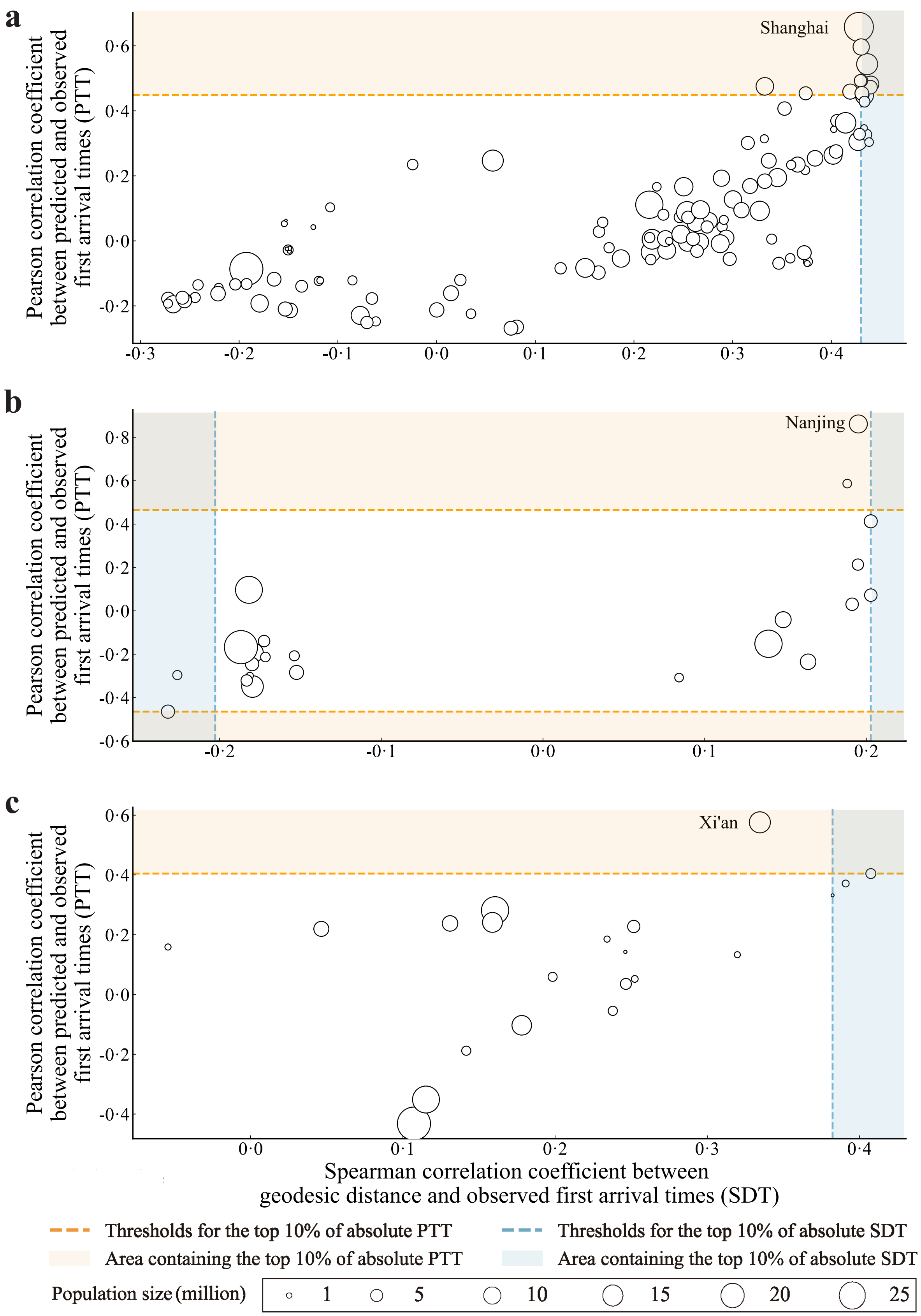


***Fig B. Origin identification after incorporating pathogen-specific transmission parameters.***

*Correlations between observed and predicted first arrival times, estimated from simulations incorporating pathogen-specific transmission parameters (reproduction number and serial interval), were compared with correlations between observed arrival times and geodesic distance for the outbreaks in Shanghai (a), Nanjing (b), and northwestern China (c). Each dot represents a candidate city, and symbol size corresponds to the resident population. Dashed lines indicate thresholds for the top 10% of absolute correlation coefficients based on predicted arrival times (orange) or geodesic distance (blue). Shaded areas highlight cities within these top 10% thresholds. Shanghai and Nanjing remained strongly supported as the outbreak's origins, and Xi'an remained the most probable origin in northwestern China. Overall, the model-data correlation-based approach provided more reliable origin identification than the distance-based method after incorporating pathogen-specific transmission parameters.*

We have also revised the manuscript to clarify this rationale, report the additional sensitivity analyses, and explicitly distinguish between (i) mobility-only validation aimed at isolating structural diffusion patterns and (ii) extended analyses incorporating pathogen-specific parameters to assess robustness. **(Page 10, Lines 232-235).**

*To provide a more comprehensive validation framework, we additionally conducted an extended validation analysis that incorporated outbreak-specific reproduction numbers and serial intervals, with the results presented in the Supplementary Material.*

3. The validation strategy relies almost exclusively on correlation between predicted and observed first arrival times. Correlation, however, measures rank or linear association rather than calibration or absolute predictive accuracy. High correlation can coexist with substantial systematic bias. The authors should justify why correlation alone is an adequate validation metric, or complement it with additional measures (e.g., mean absolute error, root mean squared error, or calibration plots).

   **Response**: We thank the reviewer for highlighting that correlation alone does not fully capture absolute predictive accuracy and may overlook systematic bias. In our framework, correlation was initially used to assess the model's ability to reproduce the relative spatial

ordering of first arrival times, which is the primary quantity of interest in early-phase diffusion. In response, we revised the Methods subsection "Simulated first arrival time" to clarify that the validation framework included the mean absolute error (MAE) and root mean square error (RMSE) in addition to Pearson correlation. **(Page 11, Lines 240-245).**

> *For each city, the predicted first arrival time used in the main analysis was summarised as the mean across simulations. Model performance was evaluated by comparing the observed first arrival times with the mean predicted first arrival times using Pearson correlation, mean absolute error (MAE), and root mean square error (RMSE).*

We also revised the Results section describing model validation to note that it no longer relied solely on correlation and that additional error-based metrics were provided. **(Page 16, Lines 379-384).**

> *To complement the correlation-based assessment, absolute prediction error was additionally evaluated using the mean absolute error (MAE) and root mean square error (RMSE), with detailed results provided in the Supplementary Material (Fig A). The consistency between correlation and error-based metrics suggests that the model captures both the relative ordering and the magnitude of arrival times without substantial systematic bias.*

We further added an extended validation analysis to the Supplementary Material, reporting the corresponding MAE and RMSE values, along with the observed-predicted first arrival  time plots. This extended analysis was based on simulations incorporating outbreak-specific reproduction numbers and serial intervals, thereby complementing the correlation-based assessment with measures of absolute predictive error.

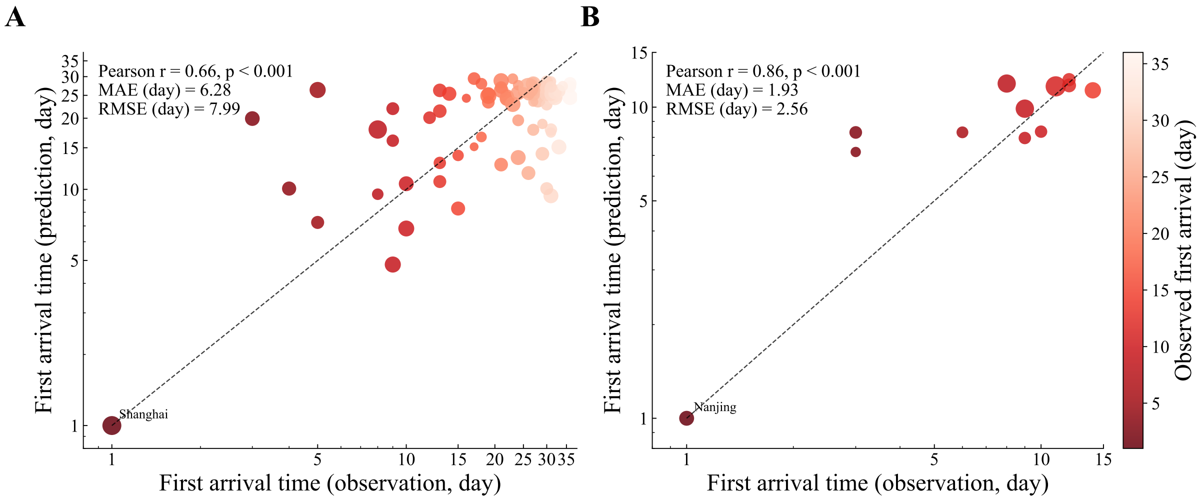


*Fig A. Extended validation of first arrival-time predictions incorporating pathogen-specific reproduction numbers and serial intervals.*

*(A) Comparison between observed and mean predicted first arrival times for cities affected by the Shanghai outbreak, based on simulations incorporating outbreak-specific reproduction number and serial interval values. (B) Comparison between observed and mean predicted first arrival times for cities affected by the Nanjing outbreak, based on simulations incorporating outbreak-specific reproduction number and serial interval values. Each dot represents a city, and the symbol size reflects its population. The dashed line indicates the 1:1 line. For the Shanghai outbreak, the Pearson correlation was 0.66 (p<0.001), with a mean absolute error (MAE) of 6.28 days and a root mean squared error (RMSE) of 7.99 days. For the Nanjing outbreak, the corresponding values were 0.86 (p<0.001), 1.93 days, and 2.56 days, respectively.*

4. The use of Pearson correlation for origin identification assumes approximate linearity and normality of log-transformed arrival times, yet the empirical distributions of arrival times across cities appear highly skewed and heterogeneous. Although a log-log correlation is mentioned in the Supplementary Methods, this transformation is not consistently described or clearly reflected in the main Methods section. Greater clarity is needed regarding which correlation metric (raw vs log-transformed) was used in each analysis and why.

**Response**: We thank the reviewer for this important comment. We agree that the original manuscript did not explain the correlation analyses with sufficient clarity, particularly regarding which correlation metrics were used, the rationale for analysing arrival times across different scales, and why different correlation metrics were used in different parts of the study. We have therefore substantially revised both the main Methods and the Supplementary Methods to clarify the analytical rationale.

Our origin-identification analysis was designed primarily to determine which candidate origin generated a simulated first arrival time pattern most similar to the observed outbreak pattern across cities. In this setting, the correlation analysis was not intended mainly as a classical parametric test of association under strict distributional assumptions. Rather, it was used as a measure of pattern similarity and as a discrimination statistic for comparing candidate origins. This distinction is important because the scientific objective here was not only to assess whether cities were reached in approximately the same order but also to distinguish among candidate origins based on the overall structure of the arrival-time pattern across cities.

For this reason, we compared observed and predicted first arrival times on the logarithmic scale in the mobility-based analyses. This choice was motivated by both theory and prior literature. Prior work on epidemic arrival times in mobility networks has shown that such arrival times are naturally analysed within logarithmic or scaling-based frameworks. Brockmann and Helbing showed that, in network-driven contagion, effective distance reliably predicts disease arrival times. That epidemic spread is more naturally characterised within a transformed network-distance framework than by raw geographic distance alone [26]. Iannelli et al. further described logarithmic metrics as a general framework for predicting disease arrival times on complex networks [27]. More recently, Klamser et al. showed that disease arrival time correlates with the logarithm of estimated import probabilities and evaluated arrival-time agreement within a log-based goodness-of-fit framework [28]. Taken together, these studies support the use of logarithmic or scaling-based formulations when analysing epidemic arrival times, rather than treating raw arrival times as the only natural comparison scale. Theoretically, as described in the Supplementary Methods, diffusion-like spread can be represented more naturally on a

scaling form. Our assumption that the first arrival time ($t$) and diffusion distance ($D$ ) satisfy a generalised power-law relation is motivated by both the physical basis of spatial diffusion and the epidemiological characteristics of COVID-19 spread driven by human mobility. At a coarse-grained level, the geographic spread of COVID-19 can be viewed as a mobility-driven spatial propagation process. In classical diffusion theory, the mean squared displacement satisfies $\langle D^2 \rangle = 2\kappa t$, where $\kappa$ is the diffusion coefficient, implying $t \propto D^2$. Thus, the power-law form has a clear physical basis under ideal diffusion. However, real-world COVID-19 transmission does not occur in a homogeneous medium; instead, it unfolds on highly heterogeneous human mobility networks. As a result, epidemic spread may deviate from classical diffusion and exhibit anomalous diffusion-like behaviour. We therefore adopt the more general form $t = \beta D^{\alpha}$. As noted by the reviewer, Pearson correlation quantifies linear association. When two variables are related by a nonlinear scaling law such as $t = \beta D^{\alpha}$, applying Pearson correlation directly to the raw data may not adequately capture their relationship. Taking logarithms yields $\ln t = \ln \beta + \alpha \ln D$, which linearizes the scaling relation. Therefore, Pearson correlation computed on the log-transformed variables is more appropriate than correlation computed on the raw variables. We have revised the manuscript to clarify this point and to explain better the physical and epidemiological rationale for the power-law assumption.

Within this log-transformed framework, we used Pearson correlation for the mobility-based analyses because the purpose was not merely to recover ordinal concordance but also to preserve information about the relative temporal spacing of arrival times across cities. This was particularly important for origin identification. A purely rank-based statistic would retain only the ordering of arrival times and discard information on how far apart the arrival times were on the transformed scale. In our setting, this loss of spacing information reduced discrimination among candidate origins. By contrast, Pearson correlation on log-transformed arrival times preserved both the broad ordering and the relative spacing of city-level arrival times, making it more informative for identifying the candidate origin whose simulated diffusion pattern most closely matched the observed one.

We used a different correlation metric for the geodesic-distance comparator because the inferential goal there was different. For the geodesic analysis, we did not know the functional relationship between diffusion distance and time in the context of SARS-CoV-2 transmission. Instead, we were interested in whether more geographically distant cities tended to be reached later in a broadly monotonic sense. Under that objective, the Spearman correlation between raw observed first arrival time and geodesic distance was the more appropriate comparator.

At the same time, we agree with the reviewer that the empirical distributions of arrival times are heterogeneous, and that it would therefore be undesirable to rely only on the classical parametric inference associated with Pearson correlation. We therefore retained the log-transformed Pearson correlation as the primary similarity and discrimination metric for the mobility-based analyses, but strengthened the inference by additionally calculating permutation-based p-values and bootstrap 95% confidence intervals. In this way, the analysis no longer depends solely on normality-based parametric inference, while still preserving the greater discriminatory value of Pearson correlation for origin identification.

Using this clarified framework, the true origin city ranked first among eligible candidate origins in both outbreaks. For the Shanghai outbreak, Shanghai ranked first with a log-transformed Pearson correlation of 0.66, a permutation p-value of 0.0005, and a bootstrap 95% confidence interval of 0.34-0.84. For the Nanjing outbreak, Nanjing also ranked first among eligible candidate origins with a log-transformed Pearson correlation of 0.86, a permutation p-value of 0.0015, and a bootstrap 95% confidence interval of 0.49-0.98. These results support the use of log-transformed Pearson correlation as an informative and discriminating measure of similarity in outbreak arrival-time patterns, while the permutation and bootstrap analyses reduce dependence on strict parametric assumptions.

To make this rationale explicit, we revised the main text and Supplementary Material as follows.

We first added the following explanation in the main Methods to clarify why the mobility-based analyses were conducted on the logarithmic scale and why Pearson correlation was used in that setting. **(Page 13, Lines 290-299).**

*We compared the simulated first arrival time pattern generated by each candidate origin with the observed first arrival time pattern across affected cities. This comparison was performed using the Pearson correlation between the logarithm of the predicted first arrival time ($lg\, T_{pre}$) and the logarithm of the observed first arrival time ($lg\, T_{obs}$). We used logarithmically transformed first arrival times for the mobility-based analyses because the relationship between the diffusion time ($t$) and diffusion distance ($D$) follows a power-law form $t \sim D^{\alpha}$. In the origin-identification analysis, Pearson correlation on log-transformed arrival times was used primarily to compare the similarity between observed and predicted arrival time patterns across cities and to preserve information about their relative temporal spacing.*

We then reorganised the Supplementary Methods so that the rationale for origin identification is presented in a single subsection. **(Supplementary Material, Page 6, Lines 141-178).**

***Origin identification using log-transformed Pearson correlation***

*To identify the most likely outbreak origin, we compared the simulated first arrival time pattern generated by each candidate origin with the observed first arrival time pattern across affected cities. Our assumption that the first arrival time ($t$) and diffusion distance ($D$) satisfy a generalized power-law relation is motivated by both the physical basis of spatial diffusion and the epidemiological characteristics of COVID-19 spread driven by human mobility. At a coarse-grained level, the geographic spread of COVID-19 can be viewed as a mobility-driven spatial propagation process. In classical diffusion theory, the mean squared displacement satisfies $\langle D^2 \rangle = 2\kappa t$, where $\kappa$ is the diffusion coefficient, implying $t \propto D^2$. Thus, the power-law form has a clear physical basis under ideal diffusion. However, real-world COVID-19 transmission does not occur in a homogeneous medium; instead, it unfolds on highly heterogeneous human mobility networks. As a result, epidemic*

*spread may deviate from classical diffusion and exhibit anomalous diffusion-like behavior. We therefore adopt the more general form* $t = \beta D^{\alpha}$*. When two variables are related by a nonlinear scaling law such as* $t = \beta D^{\alpha}$*, applying Pearson correlation directly to the raw data may not adequately capture their relationship. Taking logarithms yields* $ln\, t = ln\, \beta + \alpha\, ln\, D$*, which linearizes the scaling relation. Therefore, Pearson correlation computed on the log-transformed variables is more appropriate than correlation computed on the raw variables. We computed the Pearson correlation coefficient between the logarithm of the predicted first arrival time (*$lg\ T_{pre}$*) and the logarithm of the observed first arrival time (*$lg\ T_{obs}$*). This log-log correlation measures how well the model reproduces the relative ordering and scaling of spatial diffusion across cities, independent of the value of α. We used the logarithmic scale to capture similarity in spatial diffusion patterns on a scaling-based time scale, consistent with diffusion-based formulations and previous work on epidemic arrival times in mobility networks.*

*In the origin-identification analysis, the Pearson correlation on log-transformed arrival times was used primarily as a measure of similarity between observed and predicted arrival-time patterns across cities. This choice was made because origin identification required not only agreement in the ordering of arrival times, but also preservation of their relative temporal spacing, which improved discrimination among candidate origins. By contrast, rank-based measures such as Spearman correlation retain ordinal information only and were therefore used for the geodesic-distance comparison, where the functional relationship between distance and arrival time is not specified.*

*For origin identification, only candidate origins with at least 10 cities included in the correlation analysis were ranked, to ensure that the ranking reflected a meaningful portion of the observed spatial diffusion pattern rather than small-sample artefacts. To reduce reliance on classical parametric assumptions, statistical significance of the Pearson correlation was additionally assessed using permutation tests, and uncertainty in the correlation coefficients was quantified using bootstrap 95% confidence intervals.*

We further added a Supplementary Table to report Pearson-based origin-identification results, together with permutation-based p-values and bootstrap confidence intervals. **(Supplementary Table A).**

***Table A. Top five candidate origin cities with the highest absolute Pearson correlations between observed and predicted first arrival times in the Shanghai and Nanjing outbreaks.***

| *Outbreak* | *Rank* | *City* | *Pearson r* | *Permutation p-value* | *Bootstrap 95% CI* |
|---|---|---|---|---|---|
| *Shanghai* | *1* | *Shanghai* | *0.66* | *<0.001* | *[0.34,0.84]* |
| | *2* | *Nantong* | *0.60* | *<0.001* | *[0.41,0.73]* |
| | *3* | *Suzhou* | *0.54* | *<0.001* | *[0.35,0.73]* |
| | *4* | *Taizhou* | *0.49* | *<0.001* | *[0.35,0.67]* |
| | *5* | *Yangzhou* | *0.49* | *<0.001* | *[0.36,0.63]* |
| *Nanjing* | *1* | *Nanjing* | *0.86* | *0.001* | *[0.49,0.98]* |
| | *2* | *Ma'anshan* | *0.59* | *0.063* | *[0.38,0.96]* |
| | *3* | *Jingzhou* | *-0.46* | *0.091* | *[-0.80,-0.37]* |
| | *4* | *Suqian* | *0.41* | *0.12* | *[-0.069,0.91]* |
| | *5* | *Wuhan* | *-0.35* | *0.08* | *[-0.64,-0.28]* |

*Pearson $r$ was computed between log-transformed observed and predicted first arrival times for each candidate origin city. Candidate origins were ranked by the absolute value of the log-transformed Pearson correlation coefficient, so both positive and negative correlations could be included among the top five. Permutation p-values and bootstrap 95% confidence intervals are reported to assess the robustness of the correlation estimates. For the Shanghai outbreak, Shanghai ranked first among eligible candidate origins (Pearson r=0.66, permutation p<0.001, bootstrap 95% CI 0.34-0.84). For the Nanjing outbreak, Nanjing ranked first among eligible candidate origins (Pearson r=0.86, permutation p=0.001, bootstrap 95% CI 0.49-0.98).*

5. The approach to origin identification involves testing all 366 cities as potential sources and selecting the city with the maximum correlation. This raises concerns about multiple testing and overfitting, particularly given the relatively small number of affected cities in some outbreaks (e.g., n = 13 for Nanjing). The manuscript does not address how statistical

uncertainty in correlation estimates was handled, nor whether differences between top-ranked candidate cities were meaningfully distinguishable.

**<u>Response</u>**:

We thank the reviewer for this important comment. We agree that evaluating all candidate cities and selecting the one with the highest correlation introduces multiple sources of uncertainty, including variability in correlation estimates, selection bias due to multiple testing, and uncertainty in the ranking of candidate origins. We have revised the manuscript to address each of these issues explicitly.

To address uncertainty in the correlation estimates themselves, we now explicitly report permutation-based p-values and bootstrap 95% confidence intervals for the Pearson correlations of candidate origins. These results are presented in a new supplementary table listing the five candidate origin cities with the highest absolute Pearson correlations between observed and predicted first arrival times for the Shanghai and Nanjing outbreaks. This addition makes the uncertainty in the correlation estimates explicit, rather than reporting only point estimates. In this way, the manuscript now shows not only which candidate cities yielded strong agreement with the observed arrival-time pattern, but also how stable and statistically supported those correlation estimates were.

To address the reviewer's concern about multiple testing and overfitting at the selection stage, we additionally performed a max-statistic permutation test. The rationale for this analysis is that origin identification was based on evaluating all candidate origins and selecting the city with the highest correlation. Accordingly, the relevant null question is not whether a single pre-specified city has a high correlation, but whether the maximum observed correlation across all candidate origins exceeds what would be expected if the same search procedure were applied under the null. For each outbreak, we therefore repeatedly permuted the observed first arrival times across cities, recalculated the Pearson correlations for all eligible candidate origins in each permuted dataset, and retained the maximum correlation from that permutation. This generated a null distribution for the origin-selection procedure itself. We then compared the observed maximum correlation with this null distribution to obtain a max-statistic permutation p-

value. This approach allows us to evaluate the significance of the selected origin under the same selection procedure applied in the analysis.

To assess whether the top-ranked candidate cities were meaningfully distinguishable, we further performed a bootstrap ranking-stability analysis. For each outbreak, cities included in the correlation analysis were resampled with replacement while preserving the original sample size. For each bootstrap replicate, Pearson correlations between log-transformed observed and predicted first arrival times were recalculated for all eligible candidate origins, and candidate origins were re-ranked accordingly. From these bootstrap replicates, we summarised the frequency with which the true origin ranked first, the frequency with which it appeared within the top three, and its median bootstrap rank. We also quantified the distinguishability of the top-ranked candidate cities by calculating, for each bootstrap replicate, the difference in Pearson correlation ($\Delta r$) between the first- and second-ranked candidate origins and summarising this difference using a bootstrap 95% confidence interval. This analysis allowed us to assess whether the highest-ranked candidate city was stably supported and whether the gap between the top-ranked cities was meaningful rather than an artefact of sampling variability.

Taken together, these additions provide a more complete treatment of uncertainty in the origin-identification analysis. The permutation p-values and bootstrap confidence intervals quantify uncertainty in the correlation estimates themselves. The max-statistic permutation test quantifies uncertainty introduced by searching across all candidate origins and selecting the maximum correlation. The bootstrap ranking-stability analysis quantifies uncertainty in the ranking itself and evaluates whether the highest-ranked candidate cities were meaningfully distinguishable.

Using these additional analyses, we found that for the Shanghai outbreak, the top-ranked origin city was Shanghai, with an observed maximum Pearson correlation of 0.66 and a max-statistic permutation p-value of 0.087, indicating moderate evidence that the observed correlation exceeds that expected under the null given the selection procedure. In the bootstrap analysis, Shanghai ranked first in 46.5% of replicates, ranked within the top three in 65.4% of replicates, and had a median bootstrap rank of 2. The observed

difference in Pearson correlation between the first- and second-ranked candidate origins was 0.062, with a bootstrap 95% confidence interval of -0.29 to 0.32, indicating that the distinction between the top-ranked candidates was not statistically sharp.

For the Nanjing outbreak, the top-ranked origin city was Nanjing, with an observed maximum Pearson correlation of 0.86 and a max-statistic permutation p-value of 0.039. In the bootstrap analysis, Nanjing ranked first in 68% of replicates, ranked within the top three in 97% of replicates, and had a median bootstrap rank of 1. The observed difference in Pearson correlation between the first- and second-ranked candidate origins was 0.27, with a bootstrap 95% confidence interval of -0.27 to 0.53.

Taken together, these analyses indicate that while the correlation-based ranking captures meaningful origin information, the distinction between the top-ranked candidate cities is not always statistically definitive. We have revised the manuscript to reflect this more cautious interpretation.

We added the following sentence in the Supplementary methods to clarify how uncertainty in the correlation estimates themselves was handled. **(Supplementary Material, Page 7, Lines 196-220).**

> ***Additional uncertainty analyses for origin identification***
>
> *To further evaluate uncertainty in origin identification, we performed three complementary analyses. First, for each candidate origin, uncertainty in the Pearson correlation estimate was quantified using permutation tests and bootstrap 95% confidence intervals. These analyses assessed whether the correlation between log-transformed observed and predicted first arrival times was stronger than expected under random reassignment of observed arrival times across cities. They quantified the sampling variability of the correlation estimate.*
>
> *Second, to account for multiplicity arising from ranking all candidate origins and selecting the city with the highest correlation, we performed a max-statistic permutation test. For each outbreak, Pearson correlations between log-transformed observed and predicted first arrival times were first calculated for all*

*eligible candidate origins, and the maximum observed correlation was recorded. We then permuted the observed first arrival times across cities, recalculated the correlations for all eligible candidate origins, and retained the maximum correlation from each permuted dataset. Repeating this procedure generated a null distribution for the origin-selection process itself. The permutation p-value was calculated as the proportion of permutations in which the maximum correlation was at least as large as that observed in the original data.*

*Third, to assess the stability of candidate-origin ranking, we performed a city-level bootstrap analysis for each outbreak. In each bootstrap replicate, the cities included in the outbreak comparison were sampled with replacement, preserving the original sample size. Pearson correlations between log-transformed observed and predicted first arrival times were then recalculated for all eligible candidate origins, and candidate origins were re-ranked accordingly. We summarised the bootstrap distribution of the difference in Pearson correlation between the first- and second-ranked candidate origins to evaluate whether the highest-ranked candidate cities were meaningfully distinguishable.*

We added the following subsection in the Supplementary Results to report the results of these additional uncertainty analyses. **(Supplementary Material, Page 12, Lines 357-375).**

***Uncertainty analyses for origin identification***

*Uncertainty in the Pearson correlation estimates for candidate origins was quantified using permutation p-values and bootstrap 95% confidence intervals (Table A).*

*For the Shanghai outbreak, the top-ranked origin city was Shanghai, with an observed maximum Pearson correlation of 0.66 and a max-statistic permutation p-value of 0.087 (Fig G). In the bootstrap analysis, Shanghai ranked first in 46.5% of replicates, ranked within the top three in 65.4% of replicates, and had a median bootstrap rank of 2. The observed difference in Pearson correlation between the first- and second-ranked candidate origins was 0.062, with a bootstrap 95% confidence interval of -0.29 to 0.32.*

*For the Nanjing outbreak, the top-ranked origin city was Nanjing, with an observed maximum Pearson correlation of 0.86 and a max-statistic permutation p-value of 0.039 (Fig G), indicating stronger evidence than for the Shanghai outbreak. In the bootstrap analysis, Nanjing ranked first in 68% of replicates, ranked within the top three in 97% of replicates, and had a median bootstrap rank of 1. The observed difference in Pearson correlation between the first- and second-ranked candidate origins was 0.27, with a bootstrap 95% confidence interval of -0.27 to 0.53.*

*From the correlation-based ranking, Shanghai and Nanjing emerged as the most likely outbreak origins, although the differences between the top-ranked candidate cities were not always statistically distinguishable.*

We added the following table in the Supplementary Material to present the uncertainty in the Pearson correlation estimates for the leading candidate origins.

***Table A. The top five candidate origin cities with the highest absolute Pearson correlations between observed and predicted first arrival times in the Shanghai and Nanjing outbreaks.***

| *Outbreak* | *Rank* | *City* | *Pearson r* | *Permutation p-value* | *Bootstrap 95% CI* |
|---|---|---|---|---|---|
| *Shanghai* | *1* | *Shanghai* | *0.66* | *<0.001* | *[0.34,0.84]* |
| | *2* | *Nantong* | *0.60* | *<0.001* | *[0.41,0.73]* |
| | *3* | *Suzhou* | *0.54* | *<0.001* | *[0.35,0.73]* |
| | *4* | *Taizhou* | *0.49* | *<0.001* | *[0.35,0.67]* |
| | *5* | *Yangzhou* | *0.49* | *<0.001* | *[0.36,0.63]* |
| *Nanjing* | *1* | *Nanjing* | *0.86* | *0.001* | *[0.49,0.98]* |
| | *2* | *Ma'anshan* | *0.59* | *0.063* | *[0.38,0.96]* |
| | *3* | *Jingzhou* | *-0.46* | *0.091* | *[-0.80,-0.37]* |
| | *4* | *Suqian* | *0.41* | *0.12* | *[-0.069,0.91]* |
| | *5* | *Wuhan* | *-0.35* | *0.08* | *[-0.64,-0.28]* |

*Pearson's r was computed between the log-transformed observed and predicted first arrival times for each candidate origin city. The absolute value of Pearson r ranked candidate origins, so both positive and negative correlations could be included among the top five. Permutation p-values and bootstrap 95% confidence intervals are reported to assess the robustness of the correlation estimates. For the*

*Shanghai outbreak, Shanghai ranked first among eligible candidate origins (Pearson r=0.66, permutation p<0.001, bootstrap 95% CI 0.34-0.84). For the Nanjing outbreak, Nanjing ranked first among eligible candidate origins (Pearson r=0.86, permutation p=0.001, bootstrap 95% CI 0.49-0.98).*

We added the following figure in the Supplementary Material to present the null distributions from the max-statistic permutation test.

A

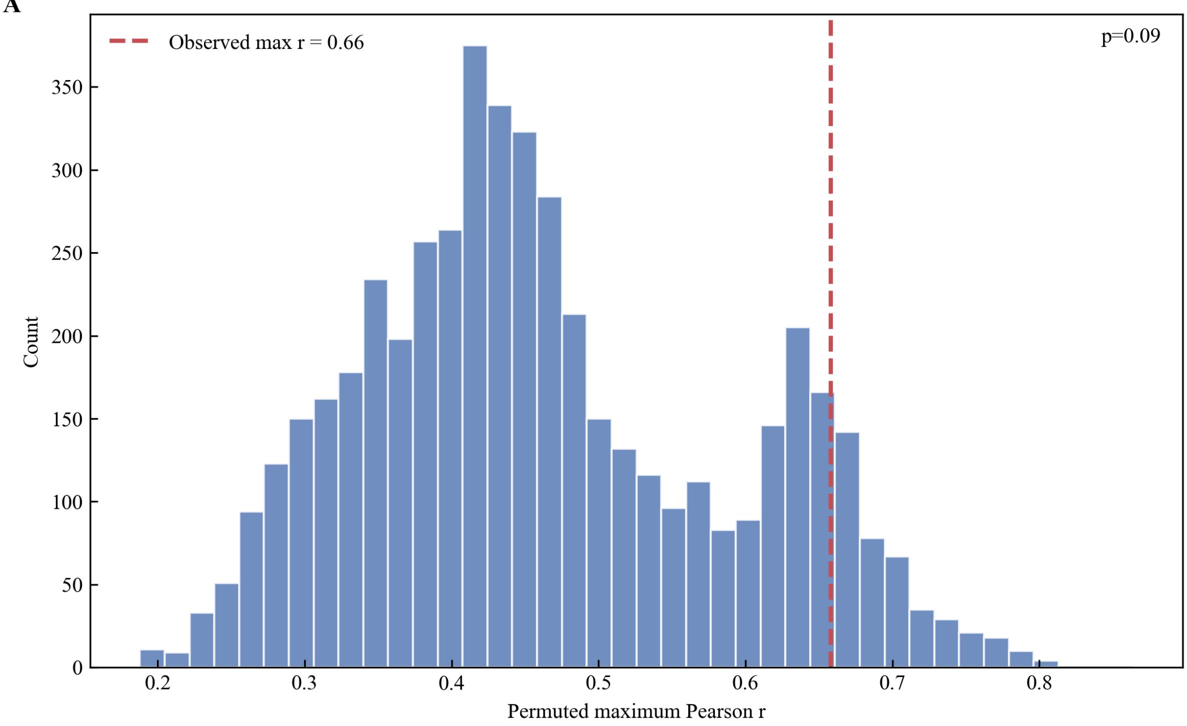


B

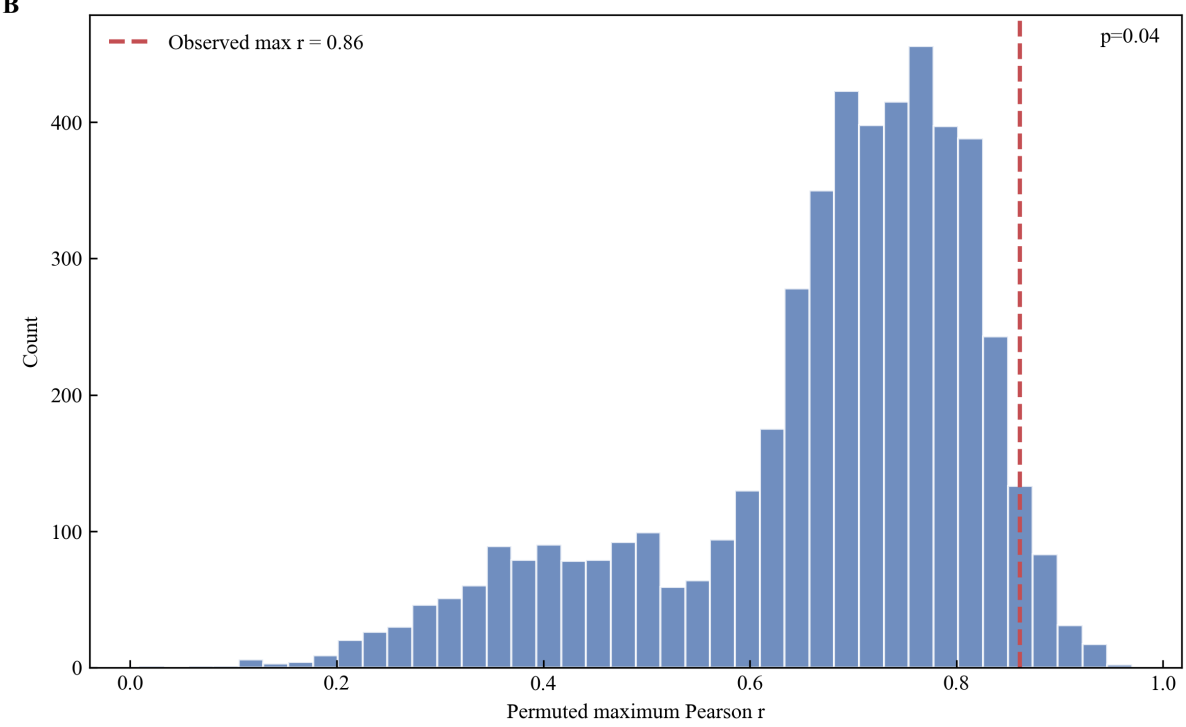


***Fig G. Max-statistic permutation test for origin identification in the Shanghai and Nanjing outbreaks.***

*Null distributions of the maximum Pearson correlation across all candidate origins obtained from the max-statistic permutation test for the Shanghai outbreak (A) and*

*the Nanjing outbreak (B). The vertical dashed line indicates the observed maximum correlation in each outbreak.*

We added the following sentence in the Results to clarify how the origin-identification results should be interpreted in light of these additional uncertainty analyses.

**Results (Page 18, Lines 418-421)**

*From the correlation-based ranking, Shanghai and Nanjing emerged as the most likely outbreak origins, although the differences between the top-ranked candidate cities were not always statistically distinguishable.*

6. The specification of the migration probability function relies on anchoring Shanghai's daily travel probability at 1%, which is treated as a fixed reference value. Although sensitivity analyses are presented, the empirical basis for selecting this baseline remains unclear. It is not evident whether this value reflects observed per-capita travel behaviour, nor how sensitive cross-tier comparisons are to this assumption in absolute (not only relative) terms.

   **<u>Response</u>**: We thank the reviewer for this important comment. We agree that the empirical basis for the 1% baseline daily migration probability for Shanghai was not sufficiently explained in the original Supplementary Methods, and we have now clarified this point. In our framework, this parameter serves as a scaling anchor for the mobility process rather than a precisely estimated individual-level quantity.

   Specifically, the 1% value was not arbitrarily assigned but was approximately derived by combining external commuting-flow data for the Yangtze River Delta with the Baidu migration index. The commuting report provides total intercity movement volumes. In contrast, the Baidu migration index provides relative outflow proportions, allowing us to back-calculate Shanghai's total out-migration and approximate a per-capita daily migration probability close to 1%.

   We would also like to clarify that this 1% value should not be interpreted as a directly observed individual-level daily travel probability, because no publicly available dataset

provides such an observed quantity for Shanghai. Instead, it is an empirically informed approximation that anchors the migration probability function. For this reason, and to evaluate the robustness of the results to this assumption, we further examined a range of baseline values from 0.5% to 1.5%.

These additional results show that changing the baseline migration probability affected the numerical magnitude of the estimated population at risk in each prefecture tier, as expected, but did not materially alter the main cross-tier comparison pattern. In absolute terms, at 7 days, the mean population at risk in the super-tier cities increased from 32.3 million to 49.2 million as the Shanghai baseline migration probability increased from 0.5% to 1.5%, while the corresponding value for tier-5 cities increased from 1.9 million to 2.3 million. Over the same range, the absolute difference between super-tier and tier-5 cities increased from 30.4 million to 46.9 million. At 14 days, the super-tier mean increased from 53.2 million to 82.8 million, whereas tier-5 increased from 2.6 million to 4.1 million, and the absolute difference increased from 50.6 million to 78.7 million. At 21 days, the super-tier mean increased from 78.9 million to 113.4 million, while tier-5 increased from 4.0 million to 7.6 million, and the absolute difference increased from 74.8 million to 105.8 million.

In relative terms, the cross-tier gradient was also preserved across the tested range. The ratio of the mean population at risk in super-tier versus tier-5 cities remained high throughout, ranging from 16.9 to 21.2 at 7 days, 20.0 to 22.0 at 14 days, and 15.0 to 20.1 at 21 days. Likewise, the ratio of tier-1 versus tier-5 remained consistently elevated, ranging from 9.8 to 11.9 at 7 days, 11.9 to 13.1 at 14 days, and 10.3 to 12.1 at 21 days. Thus, although the exact risk values changed with the baseline migration probability, the qualitative conclusion that higher-tier cities experienced substantially greater early spread risk than lower-tier cities remained unchanged.

We have revised the Supplementary Methods to clarify the derivation of the 1% baseline.

**Supplementary Material (Page 4, Lines 61-72)**

*To parameterise the migration probability function, we set the baseline daily migration probability for Shanghai to 1%. This value was not arbitrarily assigned.*

*Instead, it was approximately estimated using external commuting-flow data for the Yangtze River Delta region together with the Baidu migration index. Specifically, the commuting report provided the total population moving from Shanghai to other cities in the Yangtze River Delta, and the Baidu migration index provided the proportion of Shanghai's total outflow directed to those cities. Combining these two sources allowed us to approximately back-calculate Shanghai's total out-migration volume. Dividing this inferred total outflow by Shanghai's resident population yielded an estimated daily per-capita out-migration probability close to 1%. Because no publicly available dataset provides a directly observed individual-level daily migration probability for Shanghai, this value should be interpreted as an empirically informed approximation used to anchor the migration probability function rather than as a directly observed quantity. To assess robustness to this assumption, we additionally examined a range of baseline values from 0.5% to 1.5%.*

We have also added a subsection to the Supplementary Material that presents cross-tier comparisons of estimated population at risk in both absolute and relative terms under alternative baseline migration probabilities for Shanghai. Supplementary Fig V illustrates the same sensitivity pattern graphically.

**Supplementary Material (Page 13, Lines 424-440)**

***Cross-tier comparisons under alternative baseline migration probabilities for Shanghai***

*We further examined how cross-tier comparisons changed when the baseline daily migration probability for Shanghai was varied from 0.5% to 1.5% (Fig V; Table B). Increasing this baseline value increased the estimated population at risk across all prefecture tiers. In absolute terms, at 7 days, the mean population at risk in super-tier cities increased from 32.3 million to 49.2 million, whereas that in tier-5 cities increased from 1.9 million to 2.3 million; the corresponding absolute difference between super-tier and tier-5 cities increased from 30.4 million to 46.9 million. At 14 days, the super-tier mean increased from 53.2 million to 82.8 million and the tier-5 mean from 2.6 million to 4.1 million, with the absolute difference increasing from 50.6 million to 78.7 million. At 21 days, the super-tier mean increased from*

*78.9 million to 113.4 million and the tier-5 mean from 4.0 million to 7.6 million, with the absolute difference increasing from 74.8 million to 105.8 million. In relative terms, the cross-tier gradient remained similar across the tested range: the ratio of mean population at risk in super-tier versus tier-5 cities ranged from 16.9 to 21.2 at 7 days, 20.0 to 22.0 at 14 days, and 15.0 to 20.1 at 21 days; the corresponding ratio for tier-1 versus tier-5 cities ranged from 9.8 to 11.9, 11.9 to 13.1, and 10.3 to 12.1, respectively. These results indicate that varying the Shanghai baseline migration probability changed the numerical magnitude of the estimated risk, but did not materially alter the main cross-tier comparison pattern.*

## Supplementary Material

***Table B. Cross-tier comparisons of estimated population at risk in absolute and relative terms under alternative baseline migration probabilities for Shanghai.***

| Day 7 | People at risk (million) | | | | | | | | | |
|---|---|---|---|---|---|---|---|---|---|---|
| $p_{Sh}$(%) | Super-tier | Tier-1 | Tier-2 | Tier-3 | Tier-4 | Tier-5 | Super-tier − Tier-5 | Tier-1 − Tier-5 | Super-tier / Tier-5 | Tier-1 / Tier-5 |
| 0.5 | 32.3 | 18.7 | 8.9 | 6.1 | 3.8 | 1.9 | 30.4 | 16.8 | 16.9 | 9.8 |
| 0.6 | 31.3 | 20.5 | 9.0 | 6.0 | 3.8 | 1.9 | 29.3 | 18.5 | 16.1 | 10.6 |
| 0.7 | 36.0 | 21.3 | 9.1 | 6.1 | 3.9 | 2.0 | 34.0 | 19.3 | 17.7 | 10.5 |
| 0.8 | 36.0 | 21.8 | 9.6 | 6.4 | 3.9 | 2.1 | 33.9 | 19.7 | 17.5 | 10.6 |
| 0.9 | 37.5 | 22.6 | 9.6 | 6.6 | 4.0 | 2.1 | 35.4 | 20.5 | 18.1 | 10.9 |
| 1.0 | 41.0 | 23.3 | 10.0 | 6.6 | 4.2 | 2.1 | 38.9 | 21.2 | 19.3 | 11.0 |
| 1.1 | 42.6 | 24.2 | 10.4 | 6.8 | 4.3 | 2.1 | 40.5 | 22.1 | 19.9 | 11.3 |
| 1.2 | 42.6 | 24.6 | 10.7 | 6.8 | 4.2 | 2.2 | 40.4 | 22.5 | 19.5 | 11.3 |
| 1.3 | 44.6 | 26.5 | 10.5 | 7.0 | 4.4 | 2.2 | 42.3 | 24.3 | 20.1 | 11.9 |
| 1.4 | 46.1 | 26.5 | 11.1 | 7.0 | 4.5 | 2.2 | 43.8 | 24.2 | 20.6 | 11.8 |
| 1.5 | 49.2 | 27.7 | 10.6 | 7.2 | 4.5 | 2.3 | 46.9 | 25.3 | 21.2 | 11.9 |
| **Day 14** | **People at risk (million)** | | | | | | | | | |
| $p_{Sh}$(%) | Super-tier | Tier-1 | Tier-2 | Tier-3 | Tier-4 | Tier-5 | Super-tier − Tier-5 | Tier-1 − Tier-5 | Super-tier / Tier-5 | Tier-1 / Tier |
| 0.5 | 53.2 | 30.3 | 12.7 | 8.1 | 5.0 | 2.6 | 50.6 | 27.8 | 20.8 | 11.9 |
| 0.6 | 57.1 | 33.7 | 12.8 | 8.2 | 5.3 | 2.6 | 54.4 | 31.1 | 21.5 | 12.7 |
| 0.7 | 62.8 | 35.9 | 14.1 | 8.7 | 5.6 | 2.9 | 59.9 | 33.0 | 22.0 | 12.6 |
| 0.8 | 65.5 | 37.2 | 14.7 | 9.1 | 5.8 | 3.1 | 62.4 | 34.1 | 21.4 | 12.1 |
| 0.9 | 66.5 | 40.1 | 15.6 | 9.7 | 5.9 | 3.1 | 63.4 | 37.1 | 21.7 | 13.1 |
| 1.0 | 69.8 | 41.5 | 15.6 | 9.9 | 6.6 | 3.3 | 66.6 | 38.2 | 21.2 | 12.6 |
| 1.1 | 75.9 | 44.7 | 16.9 | 10.5 | 6.8 | 3.5 | 72.4 | 41.2 | 21.7 | 12.8 |
| 1.2 | 76.9 | 45.5 | 17.7 | 11.0 | 7.1 | 3.6 | 73.2 | 41.8 | 21.2 | 12.6 |
| 1.3 | 76.4 | 47.8 | 18.2 | 11.4 | 7.1 | 3.7 | 72.6 | 44.0 | 20.4 | 12.8 |
| 1.4 | 81.4 | 48.6 | 19.0 | 11.5 | 7.7 | 3.8 | 77.6 | 44.8 | 21.2 | 12.6 |

| | | | | | | | | | | |
|---|---|---|---|---|---|---|---|---|---|---|
| *1.5* | *82.8* | *51.2* | *18.7* | *12.1* | *7.8* | *4.1* | *78.7* | *47.0* | *20.0* | *12.4* |
| ***Day21*** | | | | | ***People at risk (million)*** | | | | | |
| *$p_{Sh}$(%)* | *Super-tier* | *Tier-1* | *Tier-2* | *Tier-3* | *Tier-4* | *Tier-5* | *Super-tier – Tier-5* | *Tier-1 – Tier-5* | *Super-tier / Tier-5* | *Tier-1 / Tier* |
| *0.5* | *78.9* | *47.6* | *19.2* | *12.1* | *7.7* | *4.0* | *74.8* | *43.6* | *19.6* | *11.8* |
| *0.6* | *85.9* | *51.6* | *20.0* | *12.6* | *8.5* | *4.3* | *81.6* | *47.3* | *20.1* | *12.1* |
| *0.7* | *91.6* | *55.5* | *22.7* | *14.4* | *9.2* | *4.6* | *87.0* | *50.9* | *19.8* | *12.0* |
| *0.8* | *93.3* | *59.2* | *24.0* | *14.6* | *9.8* | *5.2* | *88.1* | *54.0* | *18.0* | *11.4* |
| *0.9* | *98.8* | *62.3* | *26.0* | *16.1* | *10.3* | *5.4* | *93.4* | *56.9* | *18.4* | *11.6* |
| *1.0* | *100.4* | *64.4* | *26.8* | *17.0* | *11.7* | *5.7* | *94.7* | *58.7* | *17.6* | *11.3* |
| *1.1* | *109.9* | *69.2* | *28.9* | *18.0* | *12.3* | *6.3* | *103.6* | *62.9* | *17.4* | *11.0* |
| *1.2* | *108.5* | *70.6* | *29.9* | *18.6* | *12.9* | *6.5* | *102.0* | *64.1* | *16.6* | *10.8* |
| *1.3* | *111.4* | *72.7* | *31.5* | *19.8* | *13.4* | *6.7* | *104.7* | *66.0* | *16.7* | *10.9* |
| *1.4* | *114.9* | *75.6* | *32.6* | *20.2* | *14.0* | *7.1* | *107.9* | *68.5* | *16.2* | *10.7* |
| *1.5* | *113.4* | *78.0* | *32.3* | *21.6* | *14.8* | *7.6* | *105.8* | *70.4* | *15.0* | *10.3* |

*Mean population at risk was estimated for each prefecture tier under alternative baseline daily migration probabilities for Shanghai ($p_{Sh}$ = 0.5% to 1.5%). To facilitate cross-tier comparisons, both absolute and relative measures are reported. Absolute comparisons are shown as differences in mean population at risk between tiers, whereas relative comparisons are shown as ratios between tiers. Results are presented for 7, 14, and 21 days after introduction, and the population at risk is expressed in millions.*

This robustness in both absolute differences and relative ratios indicates that the main conclusions are not sensitive to the specific choice of baseline migration probability.

7. The definition of the epidemic risk metric (R*) as the total population of cities that receive at least one infected individual is a coarse, binary measure that ignores infection intensity, timing, and secondary transmission within destination cities. While the authors note this simplification, the implications for interpreting "risk" as a public health metric should be more carefully articulated, especially when comparing pathogens with very different transmission dynamics.

   **Response**: We appreciate the reviewer's thoughtful comment on the definition and interpretation of this risk metric. We agree that R*, defined as the total population of cities that have received at least one infected individual, is a simplified measure and does not capture all dimensions of epidemic risk.

Our intention was not to use this metric as a measure of local epidemic intensity, outbreak size, or healthcare burden within destination cities. In the context of early-phase spatial diffusion, our objective is to characterise whether transmission has plausibly reached a city, rather than to model downstream epidemic dynamics within that city. In this sense, it reflects the potential geographic extent of early spread rather than the magnitude of subsequent local transmission.

We also agree that the original definition does not account for the number of imported infections, the exact timing of importation within the time window, or differences in secondary transmission after introduction. These limitations are especially relevant when comparing pathogens with different transmissibility or local transmission dynamics. We have therefore revised the manuscript to clarify that this metric should be interpreted as a coarse indicator of early geographic spread potential, not as a comprehensive metric of public health burden.

At the same time, we believe this measure remains useful for this study because our primary focus was to compare how mobility structure shapes the early spatial reach of transmission across city tiers. Under this objective, an introduction-based metric provides a transparent and comparable summary of whether transmission has plausibly reached a city, while avoiding stronger assumptions about pathogen-specific local epidemic growth in each destination.

To avoid confusion with epidemiological quantities such as the basic or effective reproduction number, we have renamed the risk metric from R* to $N_i^{risk}$. In addition, we agreed with the reviewer that classifying a city as "at risk" immediately after a single imported infection may be too permissive, because it treats risk as a binary trigger and counts the entire population of that city once only one importation occurs. We therefore conducted additional analyses using more stringent importation thresholds for both SARS-CoV-2 (Omicron) and influenza A.

To assess the sensitivity of this definition, we conducted additional analyses using more stringent importation thresholds (5, 10, 50, and 100 cases), reflecting increasing plausibility of sustained local transmission. Because the number of imported infections received by destination cities also depends on the epidemic size in the origin city, we evaluated these four thresholds across three initial numbers of infections in the origin city (100, 500, and 1000 cases), yielding 12 scenario combinations in total.

For SARS-CoV-2 (Omicron), increasing the importation threshold reduced the absolute magnitude of $N_i^{risk}$, particularly at earlier time points and under smaller initial seeding sizes. This attenuation became more pronounced at the stricter thresholds of 50 and 100 imported cases, for which lower-tier cities contributed little to $N_i^{risk}$ at 7 days and remained substantially lower than higher-tier cities at 14 and 21 days. Nevertheless, the overall cross-tier pattern was preserved across all tested scenarios: super-tier cities consistently had the largest $N_i^{risk}$, followed by tier-1 cities. In contrast, lower-tier cities remained markedly lower, especially in the early phase.

We observed the same qualitative conclusion for influenza A. Applying stricter thresholds again reduced the absolute magnitude of $N_i^{risk}$, with the strongest attenuation under the 50- and 100-case thresholds. Under the strictest threshold of 100 imported cases, $N_i^{risk}$ changed little across initial seeding sizes for most tiers, indicating that few cities accumulated that many imported infections within the examined time windows. However, even under these stricter definitions, the hierarchical pattern across prefecture tiers remained unchanged: super-tier cities consistently accounted for the largest at-risk population, tier-1 cities ranked second, and lower-tier cities remained substantially lower.

Across all scenarios, although the absolute magnitude of $N_i^{risk}$ varied with the threshold and initial seeding size, the cross-tier ranking and hierarchical pattern remained consistent. Taken together, these analyses indicate that while $N_i^{risk}$ is a simplified, introduction-based metric, the principal findings regarding cross-tier differences in early spatial reach are robust to alternative, more stringent definitions of importation. We have revised the

manuscript to emphasise that $N_i^{risk}$ should be interpreted as an indicator of early geographic exposure rather than a measure of epidemic intensity or public health burden.

**Supplementary Material (Page 9, Lines 276-292)**

***Alternative definitions of $N_i^{risk}$ based on importation thresholds***

*In the main analysis, $N_i^{risk}$ was used to summarise the population living in cities considered at risk under the simulated spatial spread process. To assess the sensitivity of this metric to the treatment of risk as an introduction-based trigger, we conducted additional analyses using stricter importation thresholds for both SARS-CoV-2 (Omicron) and influenza A. Specifically, instead of including a destination city's population in $N_i^{risk}$ once that city had received at least one imported infection, we redefined $N_i^{risk}$ by including a city only when the cumulative number of imported infections reached at least 5, 10, 50, or 100 cases.*

*The rationale for this analysis was that, when sustained community transmission is of interest, the scope of transmission within a destination city may be more plausibly represented by a single imported infection and more meaningfully related to whether that city receives a sufficient number of introductions, together with its population size. Because the number of imported infections received by destination cities also depends on epidemic size in the origin city, we repeated these analyses under three alternative initial numbers of infections in the origin city (100, 500, and 1000 cases), yielding 12 scenario combinations for each pathogen. For each scenario, we recalculated $N_i^{risk}$ at 7, 14, and 21 days and compared the resulting patterns across prefecture tiers.*

**Supplementary Material (Page 14, Lines 442-465)**

***Sensitivity of $N_i^{risk}$ to alternative importation thresholds and initial seeding sizes***

*For SARS-CoV-2 (Omicron), using stricter importation thresholds reduced the estimated magnitude of $N_i^{risk}$, particularly at earlier time points and when the initial number of infections in the origin city was small. Under thresholds of 5 and 10 imported cases (Fig P, Fig Q), $N_i^{risk}$ still increased markedly with larger initial*

*seeding sizes, and the tier gradient remained clearly visible across 7, 14, and 21 days. Under stricter thresholds of 50 and 100 imported cases (Fig R, Fig S), the reduction in* $N_i^{risk}$ *became more pronounced, especially for lower-tier cities and at 7 days, indicating that these cities were less likely to accumulate large numbers of imported infections early in the spread process. Even under these stricter definitions, however, super-tier and tier-1 cities continued to account for the largest at-risk populations, while lower-tier cities remained substantially lower.*

*Influenza A showed the same qualitative pattern (Figs EE-HH). Increasing the importation threshold again reduced the magnitude of* $N_i^{risk}$*, with the strongest attenuation under the 50- and 100-case thresholds. Under the strictest threshold of 100 imported cases,* $N_i^{risk}$ *changed little across initial seeding sizes for most tiers within the examined time windows, indicating that relatively few cities accumulated that many imported infections. Nonetheless, the hierarchical ordering across prefecture tiers remained stable: super-tier cities consistently had the largest* $N_i^{risk}$ *, followed by tier-1 cities, whereas lower-tier cities remained substantially lower.*

*Overall, the absolute value of* $N_i^{risk}$ *depended on both the importation threshold and the initial number of infections in the origin city: larger thresholds reduced* $N_i^{risk}$*, whereas larger origin seeding sizes generally increased it. However, the main cross-tier comparison pattern remained qualitatively unchanged for both pathogens across all tested scenarios, indicating that the principal conclusions were not dependent on defining a city as at risk after only a single imported infection.*

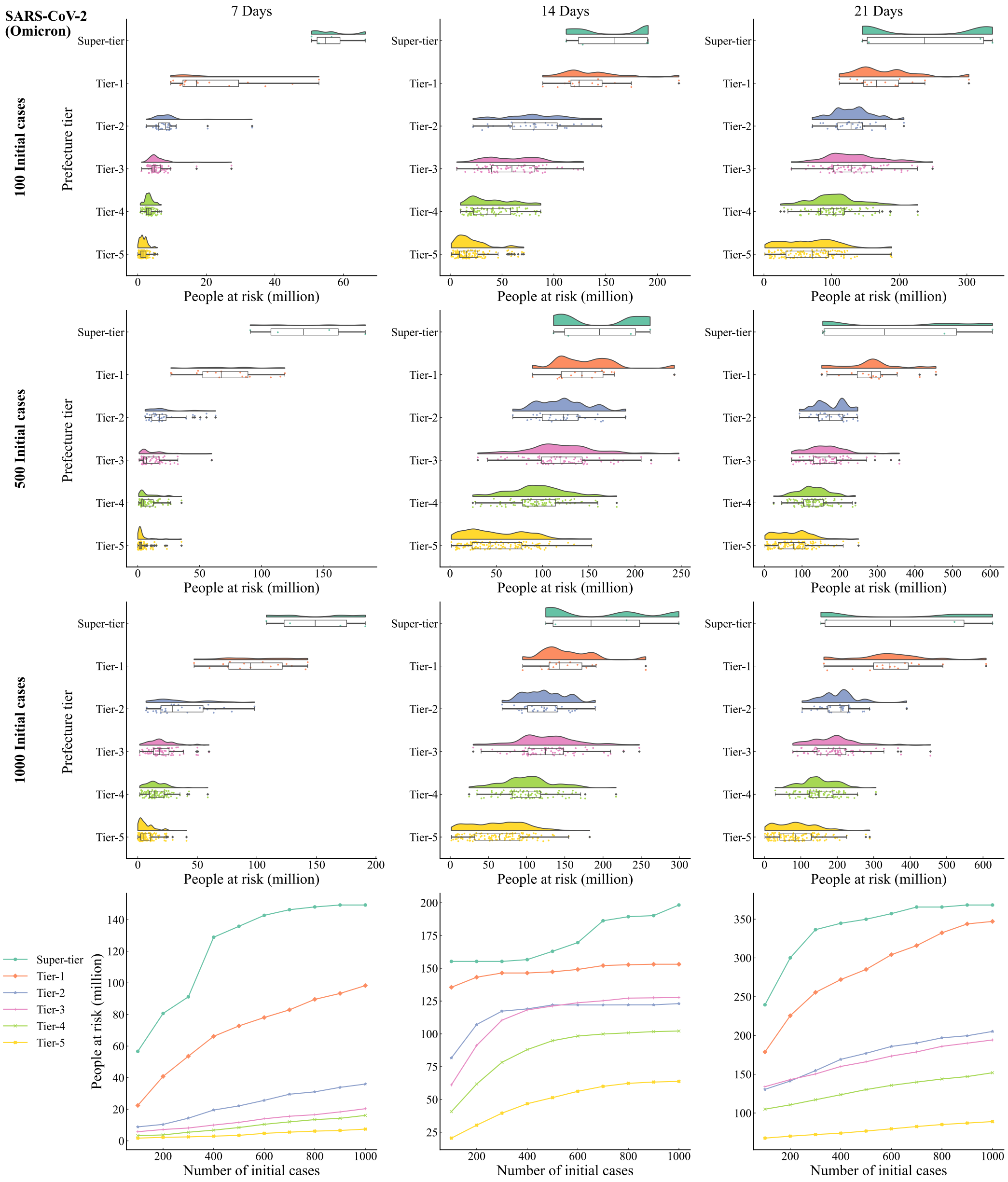


***Fig P. Sensitivity analysis of alternative importation thresholds for $N_i^{risk}$ for SARS-CoV-2 (Omicron): threshold of 5 imported cases.***

*The raincloud plots illustrate the distribution of $N_i^{risk}$ across prefecture tiers for SARS-CoV-2 (Omicron) when a city's population was included only after the cumulative number of imported infections reached 5 cases. Results are shown under three initial infection scenarios in the origin city (100, 500, and 1000 cases), evaluated at 7, 14, and 21 days after seeding. The bottom panels summarise the mean $N_i^{risk}$ for each prefecture tier as a function of the number of initial infections (100-1000). Increasing the initial seeding size increased $N_i^{risk}$ across tiers, while preserving the overall hierarchical pattern of risk distribution.*

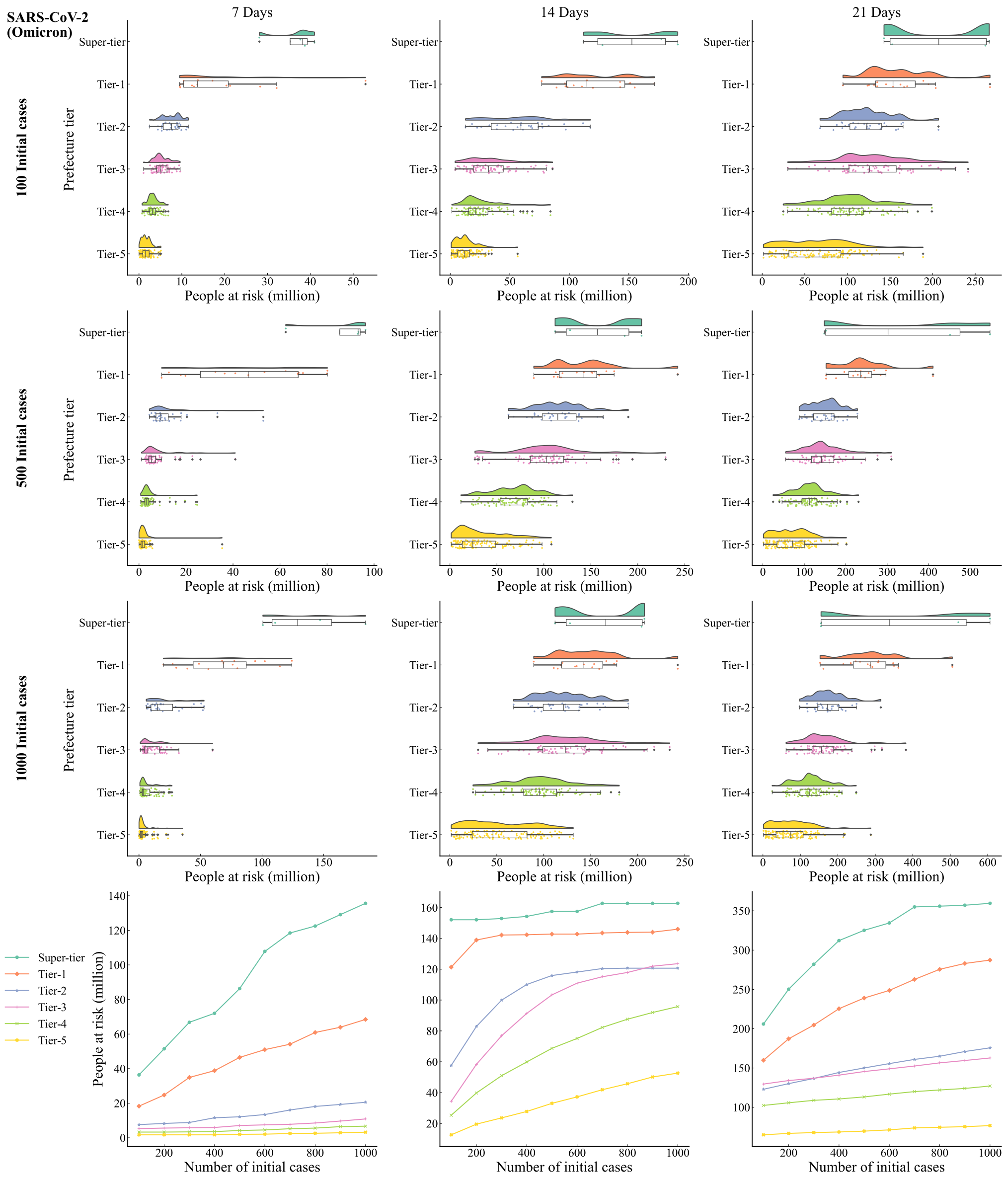


***Fig Q. Sensitivity analysis of alternative importation thresholds for $N_i^{risk}$ for SARS-CoV-2 (Omicron): threshold of 10 imported cases.***

*The raincloud plots illustrate the distribution of $N_i^{risk}$ across prefecture tiers for SARS-CoV-2 (Omicron) when a city's population was included only after the cumulative number of imported infections reached 10 cases. Results are shown under three initial infection scenarios in the origin city (100, 500, and 1000 cases), evaluated at 7, 14, and 21 days after seeding. The bottom panels summarise the mean $N_i^{risk}$ for each prefecture tier as a function of the number of initial infections (100-1000). Although the stricter threshold reduced the absolute magnitude of $N_i^{risk}$, the tier ordering remained unchanged.*

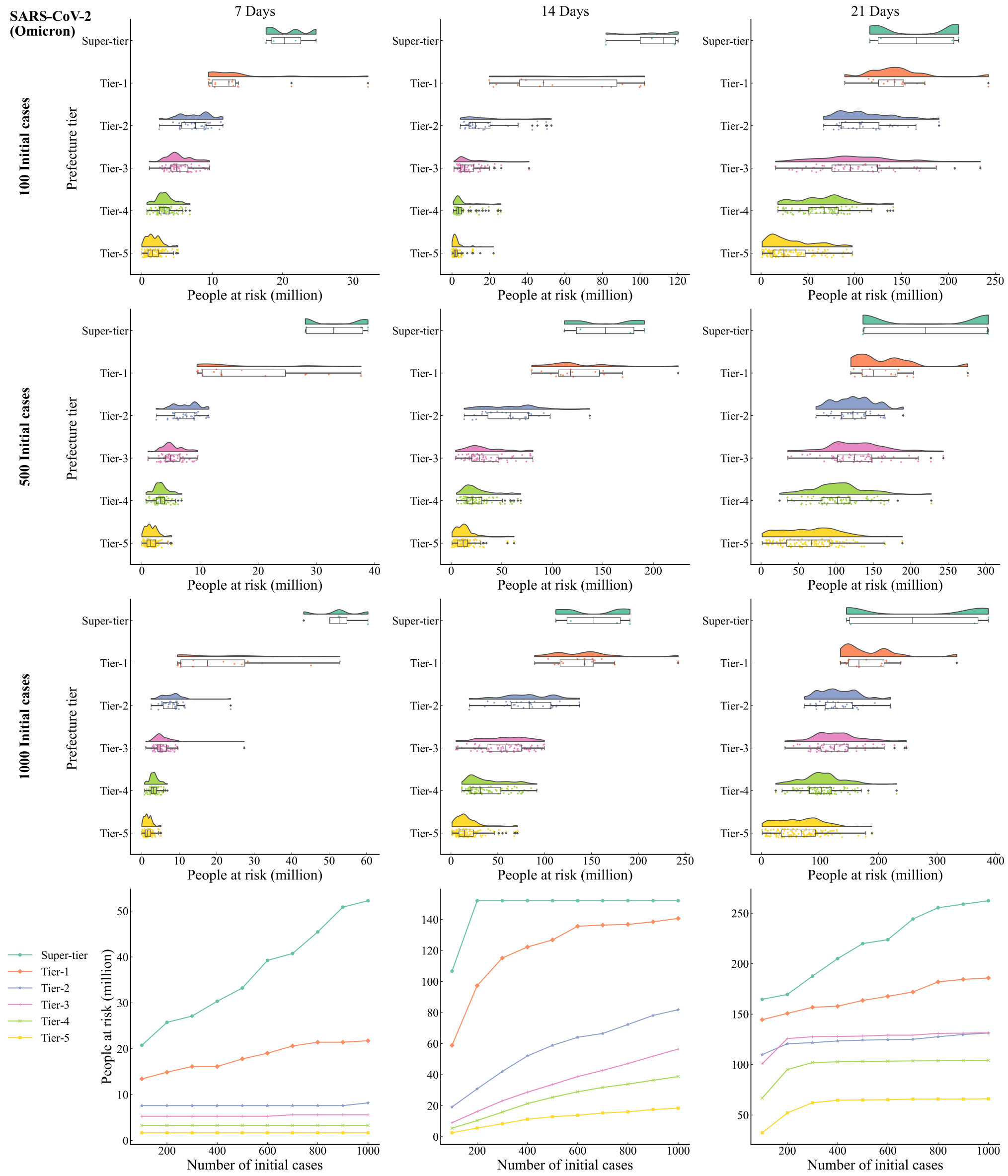


***Fig R. Sensitivity analysis of alternative importation thresholds for $N_i^{risk}$ for SARS-CoV-2 (Omicron): threshold of 50 imported cases.***

*The raincloud plots illustrate the distribution of $N_i^{risk}$ across prefecture tiers for SARS-CoV-2 (Omicron) when a city's population was included only after the cumulative number of imported infections reached 50 cases. Results are shown under three initial infection scenarios in the origin city (100, 500, and 1000 cases), evaluated at 7, 14, and 21 days after seeding. The bottom panels summarise the mean $N_i^{risk}$ for each prefecture tier as a function of the number of initial infections (100-1000). Under this stricter threshold, $N_i^{risk}$ was attenuated more strongly, particularly in lower-tier cities and at earlier time points, but the overall hierarchical pattern across tiers was preserved.*

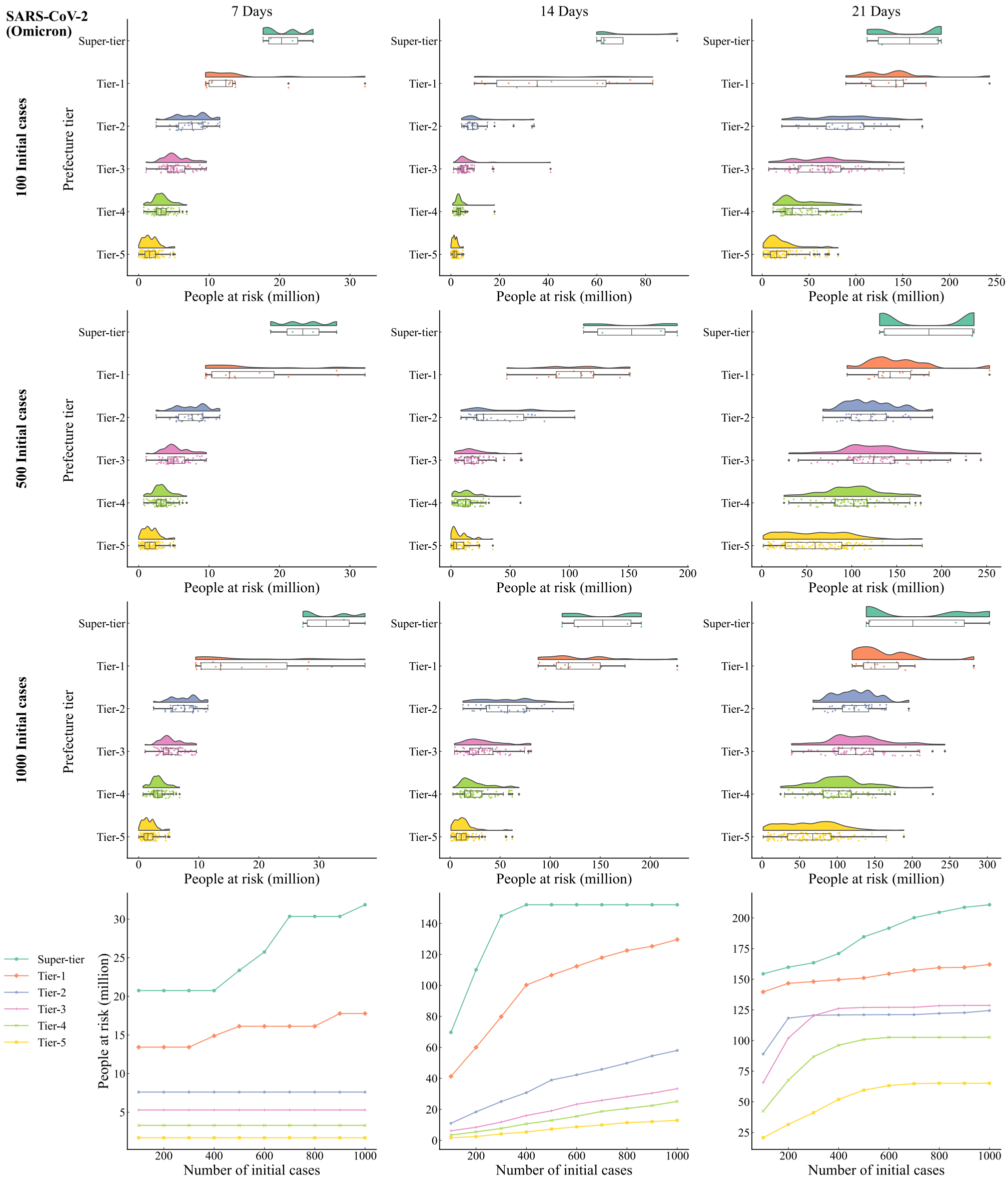


***Fig S. Sensitivity analysis of alternative importation thresholds for $N_i^{risk}$ for SARS-CoV-2 (Omicron): threshold of 100 imported cases.***

*The raincloud plots illustrate the distribution of $N_i^{risk}$ across prefecture tiers for SARS-CoV-2 (Omicron) when a city's population was included only after the cumulative number of imported infections reached 100 cases. Results are shown under three initial infection scenarios in the origin city (100, 500, and 1000 cases), evaluated at 7, 14, and 21 days after seeding. The bottom panels summarise the mean $N_i^{risk}$ for each prefecture tier as a function of the number of initial infections (100-1000). This strictest threshold further reduced the absolute magnitude of $N_i^{risk}$, especially for lower-tier cities, while leaving the relative hierarchy across prefecture tiers unchanged.*

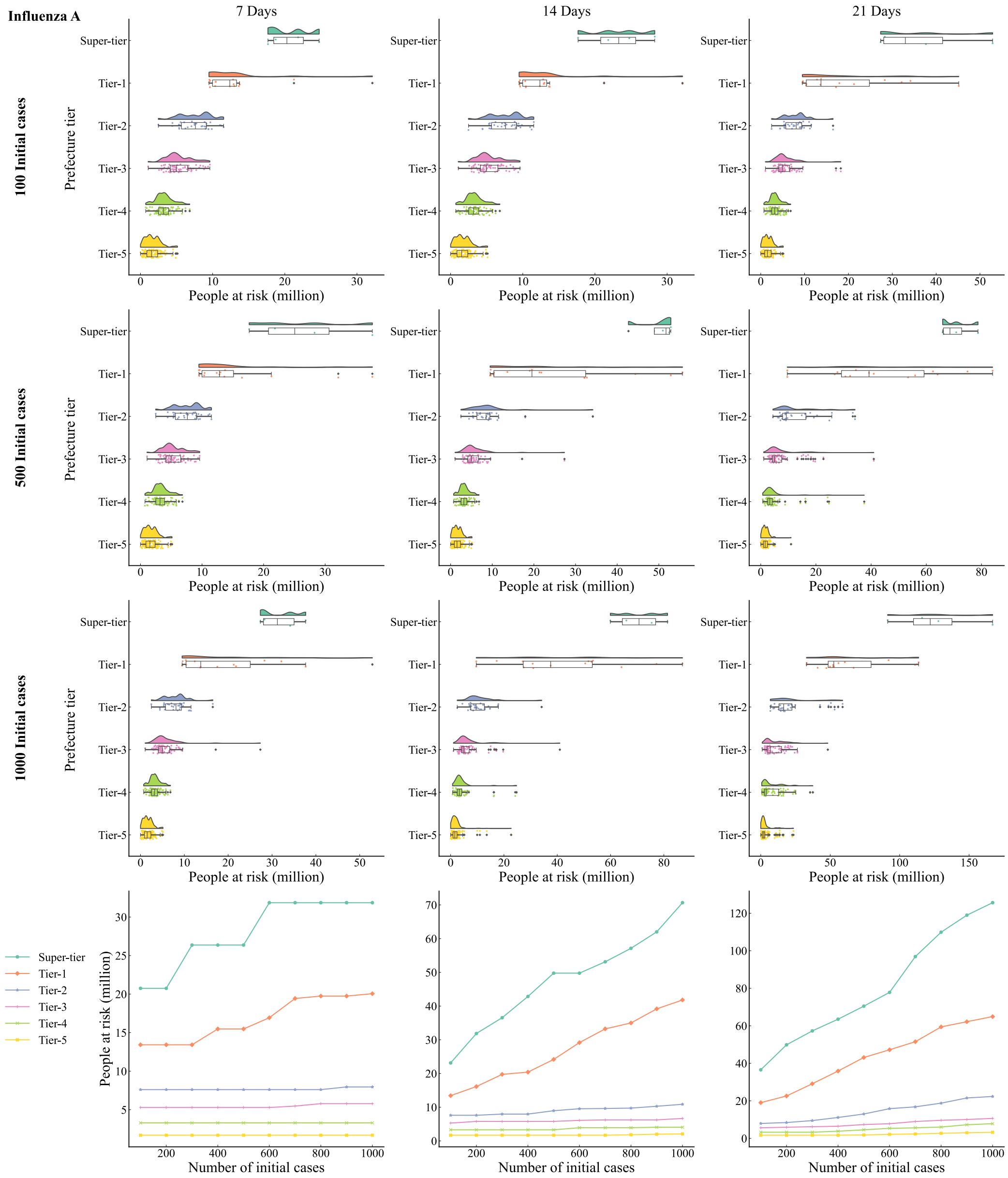


***Fig EE. Sensitivity analysis of alternative importation thresholds for $N_i^{risk}$ for influenza A: threshold of 5 imported cases.***

*The raincloud plots illustrate the distribution of $N_i^{risk}$ across prefecture tiers for influenza A when a city's population was included only after the cumulative number of imported infections reached 5 cases. Results are shown under three initial infection scenarios in the origin city (100, 500, and 1000 cases), evaluated at 7, 14, and 21 days after seeding. The bottom panels summarise the mean $N_i^{risk}$ for each prefecture tier as a function of the number of initial infections (100-1000). Increasing the initial seeding size increased $N_i^{risk}$ across tiers while preserving the overall hierarchical pattern.*

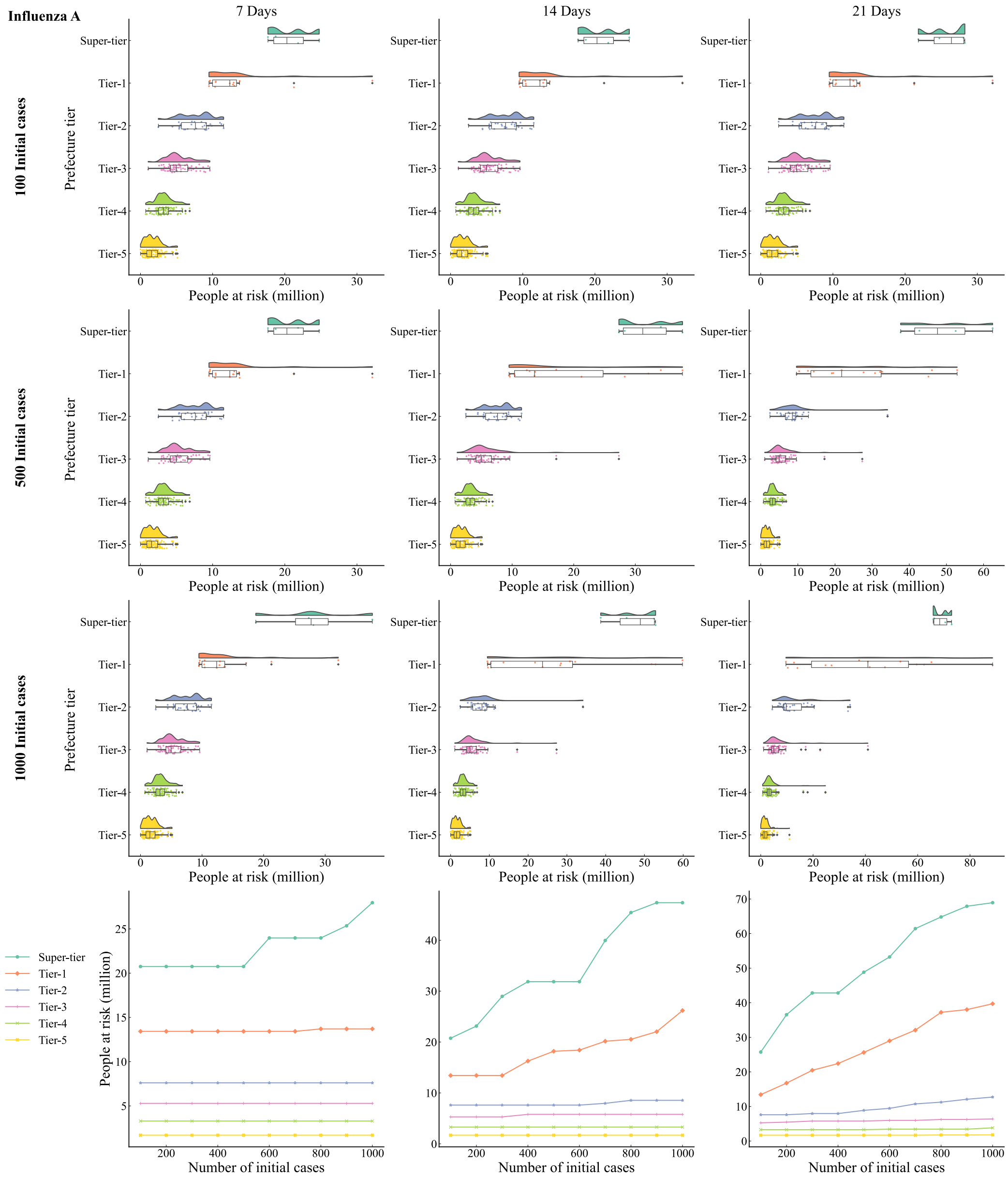


***Fig FF. Sensitivity analysis of alternative importation thresholds for $N_i^{risk}$ for influenza A: threshold of 10 imported cases.***

*The raincloud plots illustrate the distribution of $N_i^{risk}$ across prefecture tiers for influenza A when a city's population was included only after the cumulative number of imported infections reached 10 cases. Results are shown under three initial infection scenarios in the origin city (100, 500, and 1000 cases), evaluated at 7, 14, and 21 days after seeding. The bottom panels summarise the mean $N_i^{risk}$ for each prefecture tier as a function of the number of initial infections (100-1000). The stricter threshold reduced the absolute magnitude of $N_i^{risk}$, but the tier ordering remained stable.*

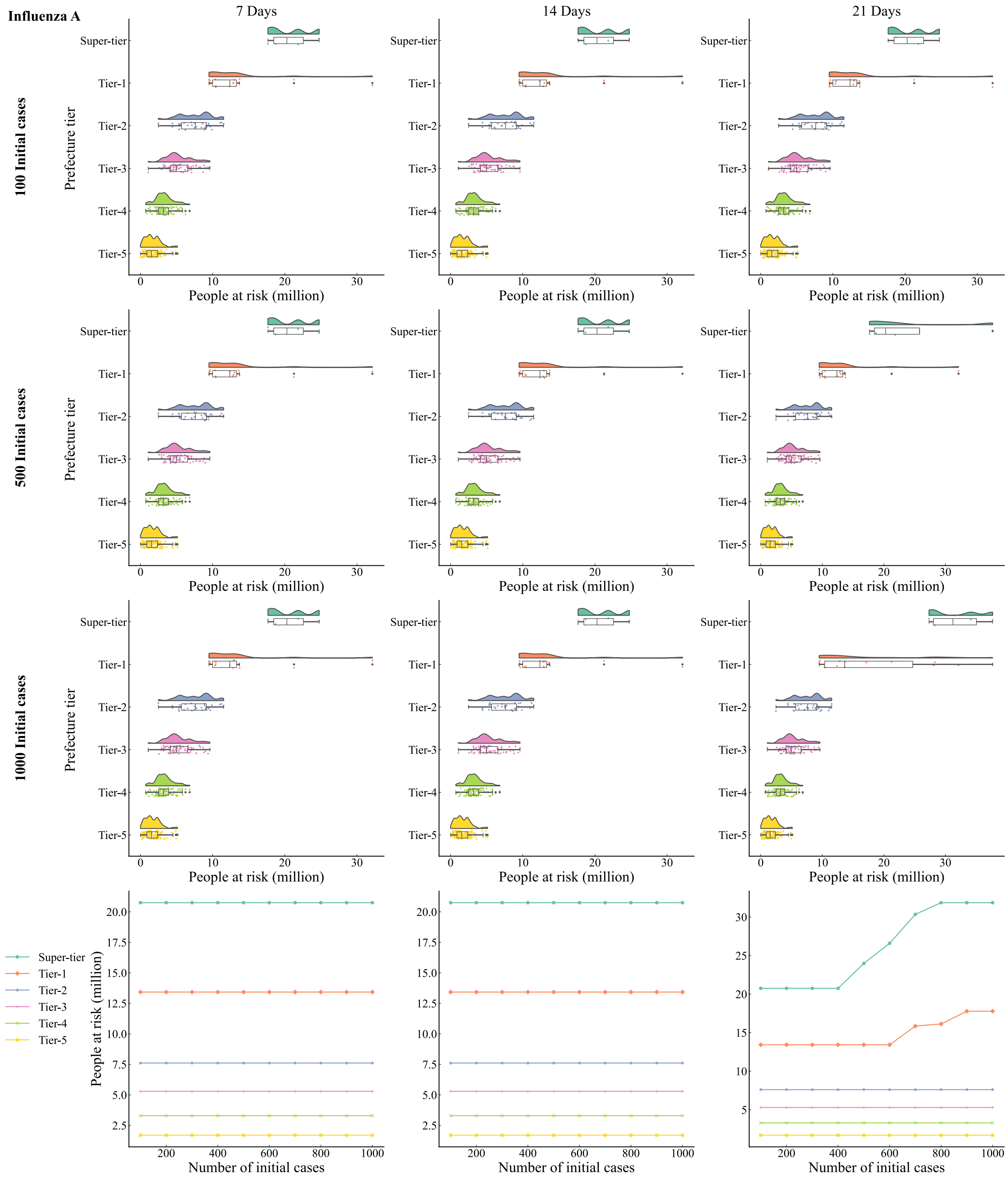


***Fig GG. Sensitivity analysis of alternative importation thresholds for $N_i^{risk}$ for influenza A: threshold of 50 imported cases.***

*The raincloud plots illustrate the distribution of $N_i^{risk}$ across prefecture tiers for influenza A when a city's population was included only after the cumulative number of imported infections reached 50 cases. Results are shown under three initial infection scenarios in the origin city (100, 500, and 1000 cases), evaluated at 7, 14, and 21 days after seeding. The bottom panels summarise the mean $N_i^{risk}$ for each prefecture tier as a function of the number of initial infections (100-1000). Under this threshold, $N_i^{risk}$ was further attenuated, particularly in lower-tier cities, while the hierarchical pattern across tiers remained unchanged.*

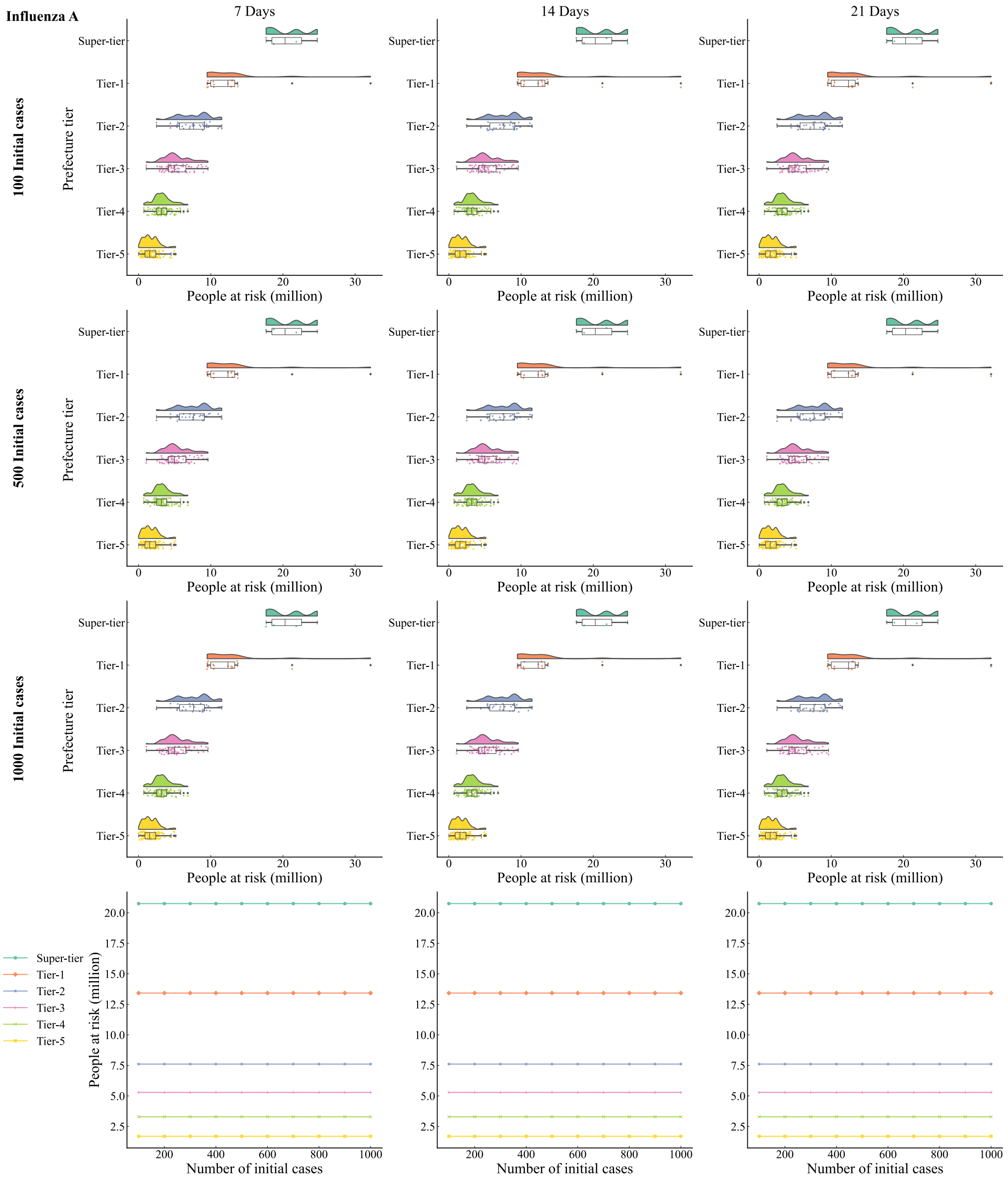


***Fig HH. Sensitivity analysis of alternative importation thresholds for $N_i^{risk}$ for influenza A: threshold of 100 imported cases.***

*The raincloud plots illustrate the distribution of $N_i^{risk}$ across prefecture tiers for influenza A when a city's population was included only after the cumulative number of imported infections reached 100 cases. Results are shown under three initial infection scenarios in the origin city (100, 500, and 1000 cases), evaluated at 7, 14, and 21 days after seeding. The bottom panels summarise the mean $N_i^{risk}$ for each prefecture tier as a function of the number of initial infections (100-1000). Under this strictest threshold, $N_i^{risk}$ changed little across initial seeding sizes for most tiers within the examined time windows, indicating that few cities accumulated 100 imported infections, but the overall tier hierarchy remained stable.*

8. Statistical inference for tier-level comparisons is insufficiently described. The manuscript reports highly significant p-values (e.g., $p < 0.001$) when comparing epidemic risk across prefecture tiers, but does not specify the underlying statistical test, the unit of analysis, or whether assumptions such as independence were met. Given that cities are connected through the mobility network, treating them as independent observations may not be valid.

   **<u>Response</u>**: We thank the reviewer for this important comment and agree that the statistical inference for tier-level comparisons required clearer specification.

   In the original analysis, the unit of analysis was the city, and differences in the risk metric across prefecture tiers were assessed using the Kruskal-Wallis test. We have now revised the manuscript to explicitly state both the statistical test used and the unit of analysis underlying the reported p-values.

   We also agree with the reviewer that cities connected through the mobility network cannot be considered strictly independent observational units. Therefore, although the Kruskal-Wallis test provided an initial non-parametric comparison of tier-level distributions, its p-values should be interpreted cautiously in this setting.

   To address this concern, we conducted additional permutation-based analyses for the tier-level comparisons. This approach relaxes the independence assumption by evaluating whether the observed tier-level differences exceed what would be expected under random reassignment of tier labels. Specifically, for each analysis, we retained the city-level values of the risk metric and the number of cities in each prefecture tier, and randomly permuted the tier labels across cities, then recalculated the Kruskal-Wallis statistic repeatedly to generate an empirical null distribution. We then computed permutation-based p-values by comparing the observed Kruskal-Wallis statistic with this null distribution. These additional analyses confirmed that the observed differences across prefecture tiers remained unlikely to arise under random assignment of tier labels,

supporting the robustness of the hierarchical tier pattern without relying on the same independence assumption as the conventional test.

Accordingly, we have revised the manuscript and Supplementary Methods to clarify the original statistical procedure, the unit of analysis, and the permutation-based robustness analysis. We now frame the tier-level p-values more cautiously in light of network dependence among cities. These findings indicate that the observed cross-tier differences are not an artifact of the independence assumption and remain robust when assessed against an empirical null distribution.

**Supplementary Material (Page 10, Lines 294-308)**

***Permutation-based inference for tier-level comparisons***

*Differences in the risk metric across prefecture tiers were originally assessed using the Kruskal-Wallis test, with city-level values treated as the unit of analysis. For each pathogen and time point, city-level values of* $N_i^{risk}$ *were grouped by prefecture tier and compared using this non-parametric rank-based test.*

*Because cities are connected through the mobility network and therefore may not be strictly independent observational units, we additionally performed permutation-based analyses to assess the robustness of the cross-tier comparisons. For each pathogen and time point, we first calculated the observed Kruskal-Wallis statistic for the city-level values of* $N_i^{risk}$ *across prefecture tiers. We then randomly permuted prefecture-tier labels across cities while preserving the number of cities in each tier, and recalculated the Kruskal-Wallis statistic for each permuted dataset to generate an empirical null distribution. The permutation-based p-value was calculated as the proportion of permuted Kruskal-Wallis statistics greater than or equal to the observed statistic. This analysis was used as a robustness check to evaluate whether the observed tier gradient was stronger than expected under random reassignment of tier labels, without relying solely on the conventional asymptotic p-value from the Kruskal-Wallis test.*

**Supplementary Material (Page 14, Lines 467-482)**

***Permutation-based assessment of cross-tier differences in $N_i^{risk}$***

*Permutation-based analyses supported the original Kruskal-Wallis comparisons of $N_i^{risk}$ across prefecture tiers (Table C). For SARS-CoV-2 (Omicron), the observed Kruskal-Wallis statistics were 142.58 at 7 days, 175.50 at 14 days, and 169.51 at 21 days, with permutation-based p<0.001 for all three time points. The corresponding Kruskal-Wallis p-values were $5.06\times10^{-29}$, $4.90\times10^{-36}$, and $9.29\times10^{-35}$, respectively. For influenza A, the observed Kruskal-Wallis statistics were 198.32, 159.55, and 133.52 at 7, 14, and 21 days, respectively, and the permutation-based p-values were again <0.001 at all three time points; the corresponding Kruskal-Wallis p-values were $6.51\times10^{-41}$, $1.23\times10^{-32}$, and $4.26\times10^{-27}$. These results indicate that the observed hierarchical differences in $N_i^{risk}$ across prefecture tiers were substantially stronger than expected under random reassignment of tier labels.*

*Taken together, these analyses indicate that the observed hierarchical differences in $N_i^{risk}$ across prefecture tiers are robust to the choice of statistical framework and do not rely solely on independence assumptions. Although cities in the mobility network are not strictly independent, the cross-tier gradient in $N_i^{risk}$ remained strongly supported when assessed against an empirical null distribution based on permuted tier labels.*

***Table C. Kruskal-Wallis and permutation-based p-values for cross-tier comparisons of $N_i^{risk}$.***

| *pathogen* | *Time (days)* | *Kruskal-Wallis statistic* | *Kruskal-Wallis p-value* | *Permutation p-value* |
|---|---|---|---|---|
| *Omicron* | *7* | *142.58* | *<0.001* | *<0.001* |
| | *14* | *175.4958* | *<0.001* | *<0.001* |
| | *21* | *169.5093* | *<0.001* | *<0.001* |
| *influenza* | *7* | *198.3158* | *<0.001* | *<0.001* |
| | *14* | *159.5531* | *<0.001* | *<0.001* |
| | *21* | *133.518* | *<0.001* | *<0.001* |

*City-level values of $N_i^{risk}$ were compared across prefecture tiers using the Kruskal-Wallis test. Because cities connected through the mobility network are not strictly independent, permutation-based p-values were additionally calculated by randomly permuting prefecture-tier labels across cities while preserving the*

*number of cities in each tier. Results are shown for SARS-CoV-2 (Omicron) and influenza A at 7, 14, and 21 days.*

**Results (Page 20, Lines 452-453)**

*Differences in* $N_i^{risk}$ *across prefecture tiers were highly significant in Kruskal-Wallis tests and remained robust in permutation-based analyses.*

9. The treatment of uncertainty in model inputs (R, serial interval parameters, migration probabilities) is largely limited to one-way sensitivity analyses. A more coherent probabilistic uncertainty analysis—jointly sampling key parameters—would be more consistent with best practice in stochastic epidemic modelling and would strengthen confidence in the robustness of the conclusions.

   **Response**: We thank the reviewer for this important suggestion. We agree that a joint probabilistic uncertainty analysis is more appropriate than relying only on one-way sensitivity analyses when evaluating the robustness of a stochastic epidemic model. In response, we performed an additional global uncertainty analysis in which key epidemiological and mobility-related parameters were varied jointly rather than individually. Across this analysis, the main cross-tier spatial risk patterns remained qualitatively consistent with the baseline results.

   We jointly sampled six parameters: the mean and overdispersion parameter of the reproduction number distribution, the shape and scale parameters of the serial interval distribution, the sigmoid scaling parameter used to transform migration intensity into movement probability, and the baseline outbound migration probability for Shanghai. The sampling ranges were selected to span plausible values and to encompass the baseline assumptions and sensitivity ranges considered elsewhere in the manuscript: mean reproduction number 1-5, overdispersion 0.10-10, serial interval shape 1-5.0, serial interval scale 0.10-2.0, sigmoid scaling parameter 0.2-2.0, and Shanghai baseline migration probability 0.005-0.015. We generated 28 Latin hypercube parameter sets and reran the full simulation and risk assessment for each set. The sampled parameter combinations spanned the multidimensional uncertainty space well, as indicated by the pairwise scatter matrix of the sampled values.

For each parameter set, we recalculated the population at risk across prefecture tiers using the same framework as in Figure 4 of the main text. Across the joint parameter samples, the overall spatial risk pattern remained qualitatively consistent with the baseline results. Across 7-day, 14-day, and 21-day risk thresholds, the relative spatial pattern of risk remained stable, with consistently greater risk concentrated in higher-tier cities and broadly similar tier-specific distributions across simulations. This indicates that the central inference of the study does not depend on any single parameter configuration but persists under simultaneous variation in key epidemiological and mobility assumptions. Thus, while joint sampling introduced expected variation in the absolute estimated magnitude of risk, it did not alter the core inference that transmission risk is systematically heterogeneous across urban tiers.

We have incorporated this new probabilistic uncertainty analysis into the revised Supplementary Methods and Supplementary Results, and we now refer to it in the main text when discussing robustness. This addition complements the original one-way sensitivity analyses and provides a more coherent assessment of uncertainty propagation through the model. We note that this analysis is intended to assess the structural robustness of the model conclusions rather than to provide a fully probabilistic prediction framework.

We added the following text to the Supplementary Material. **(Page 9, Lines 251-274).**

***Joint probabilistic uncertainty analysis***

*To evaluate the model's robustness to simultaneous uncertainty in key epidemiological and mobility-related inputs, we performed a joint probabilistic uncertainty analysis using Latin hypercube sampling. This analysis was designed to complement the one-way sensitivity analyses by propagating uncertainty in multiple parameters through the full simulation framework simultaneously.*

*We jointly varied six parameters that could materially affect transmission dynamics and spatial risk estimates: the mean reproduction number ($\mu_R$), the overdispersion parameter of the offspring distribution ($k$), the shape and scale parameters of the*

*gamma-distributed serial interval, the sigmoid scaling parameter used in the migration probability function, and the baseline outbound migration probability for Shanghai. The sampling ranges were specified as follows: $\mu_R$, 1 to 5; $k$, 0.10-10; serial interval shape, 1 to 5.0; serial interval scale, 0.10 to 2.0; sigmoid scaling parameter, 0.2 to 2.0; and baseline Shanghai migration probability, 0.005 to 0.015.*

*We generated 28 parameter combinations using Latin hypercube sampling to obtain broad coverage of the multidimensional parameter space (Table D). For each sampled parameter set, we reran the transmission simulation and repeated the downstream risk assessment using the same analytical framework as in the main analysis. For comparability with the main text, we summarised the resulting population at risk across prefecture tiers, using the same risk definitions and time windows as in Figure 4.*

*The complete set of sampled parameter combinations is provided in Supplementary Table D. Pairwise distributions of the sampled values are shown in Supplementary Fig W, illustrating the spread of the sampled points across the prespecified ranges. Risk estimates across the 28 sampled parameter sets are shown in Supplementary Figs X-Z for the 7-day, 14-day, and 21-day thresholds, respectively.*

We added the following text to the Supplementary Material **(Page 15, Lines 484-497)**

***Robustness of tier-specific risk patterns under joint parameter uncertainty***

*The joint probabilistic uncertainty analysis showed that the main conclusions were robust to simultaneous variation in the reproduction number distribution, serial interval distribution, and migration probability parameters. The 28 Latin hypercube parameter sets provided broad coverage of the prespecified uncertainty space, without obvious clustering in pairwise projections of sampled values (Fig W).*

*Across the 28 sampled parameter sets, uncertainty in model inputs changed the absolute magnitude and spread of the estimated population at risk. Still, the broad tier-specific structure of the results remained similar to that in the baseline analysis.*

*In particular, the contrast between higher-tier and lower-tier cities remained apparent across simulations (Figs X-Z).*

*These findings indicate that the central inference of the study is not dependent on a single fixed choice of epidemiological or migration parameters. Rather, the association between urban hierarchy and spatial transmission risk remained stable under joint uncertainty in the major model inputs considered here.*

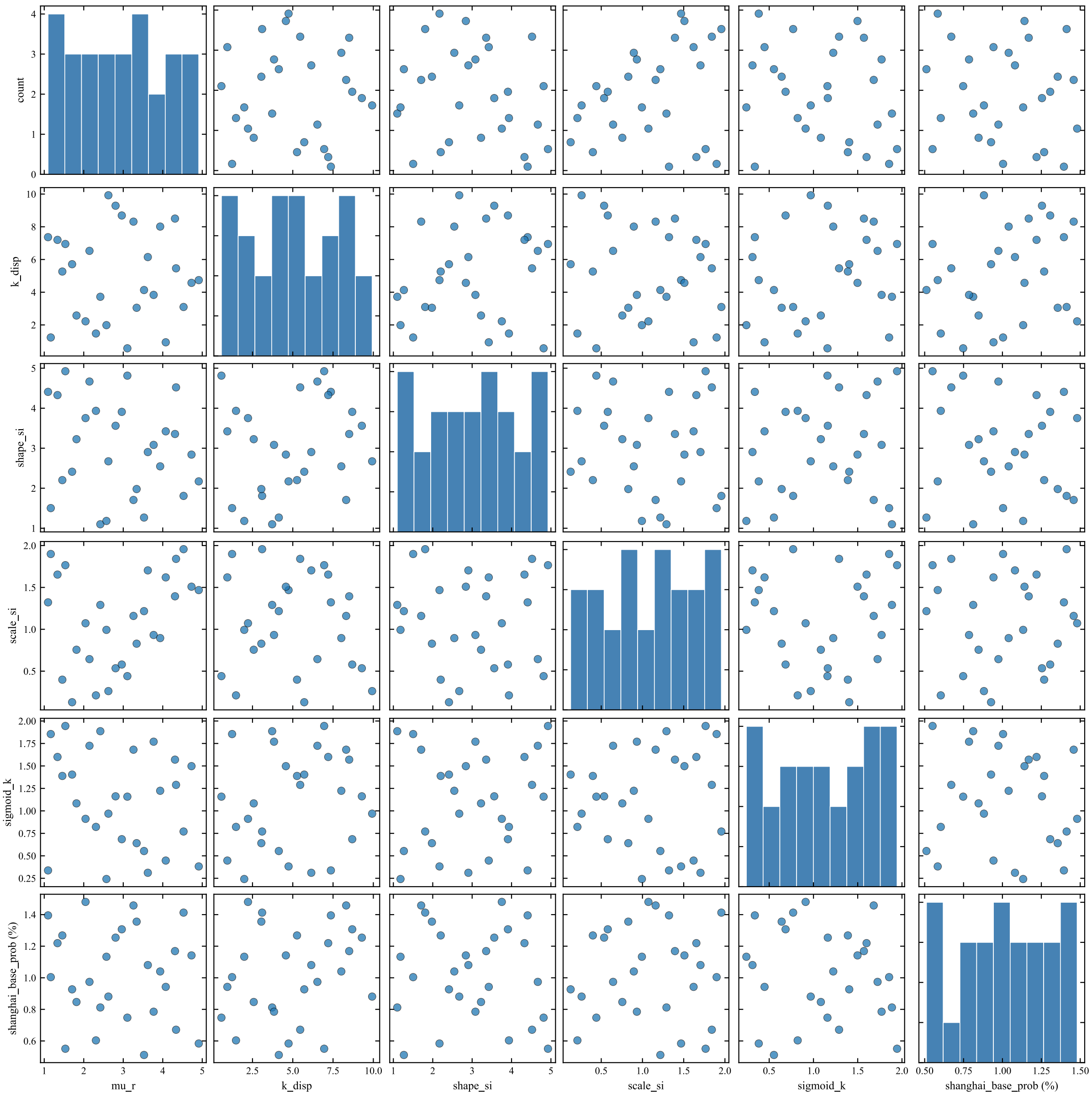


***Fig W. Latin hypercube samples for the joint probabilistic uncertainty analysis.***

*Pairwise scatter matrix showing the 28 Latin hypercube-sampled parameter sets used in the joint uncertainty analysis. Parameters jointly sampled were the mean reproduction number ($\mu_R$), overdispersion parameter ($k$), serial interval shape, serial interval scale, sigmoid scaling parameter for migration probability, and baseline outbound migration probability for Shanghai. Histograms on the diagonal show the marginal distribution of sampled values for each parameter, and off-diagonal panels show pairwise projections of the sampled combinations. Together, these plots illustrate broad coverage of the prespecified multidimensional parameter space.*

***Table D. Latin hypercube-sampled parameter sets used in the joint probabilistic uncertainty analysis.***

| Param_id | Mean reproduction number, $\mu_R$ | Overdispersion, $k$ | Serial interval shape | Serial interval scale | Sigmoid scaling parameter | Baseline Shanghai migration probability |
|---|---|---|---|---|---|---|
| 1 | 1.10 | 7.36 | 4.41 | 1.32 | 0.34 | 1.40% |
| 2 | 1.17 | 1.23 | 1.50 | 1.90 | 1.85 | 1.00% |
| 3 | 1.34 | 7.20 | 4.33 | 1.65 | 1.60 | 1.22% |
| 4 | 1.46 | 5.26 | 2.20 | 0.40 | 1.39 | 1.27% |
| 5 | 1.53 | 6.95 | 4.92 | 1.77 | 1.94 | 0.55% |
| 6 | 1.71 | 5.71 | 2.41 | 0.13 | 1.40 | 0.93% |
| 7 | 1.82 | 2.58 | 3.23 | 0.76 | 1.08 | 0.85% |
| 8 | 2.05 | 2.22 | 3.75 | 1.07 | 0.91 | 1.48% |
| 9 | 2.15 | 6.53 | 4.67 | 0.64 | 1.72 | 0.97% |
| 10 | 2.31 | 1.48 | 3.93 | 0.21 | 0.82 | 0.60% |
| 11 | 2.42 | 3.72 | 1.10 | 1.29 | 1.89 | 0.81% |
| 12 | 2.57 | 1.99 | 1.18 | 0.99 | 0.24 | 1.13% |
| 13 | 2.62 | 9.93 | 2.67 | 0.26 | 0.97 | 0.88% |
| 14 | 2.81 | 9.29 | 3.56 | 0.53 | 1.16 | 1.25% |
| 15 | 2.96 | 8.69 | 3.91 | 0.58 | 0.68 | 1.31% |
| 16 | 3.10 | 0.57 | 4.81 | 0.44 | 1.16 | 0.75% |
| 17 | 3.26 | 8.31 | 1.71 | 1.16 | 1.68 | 1.46% |
| 18 | 3.34 | 3.05 | 1.98 | 0.83 | 0.64 | 1.36% |
| 19 | 3.53 | 4.13 | 1.27 | 1.22 | 0.55 | 0.51% |
| 20 | 3.62 | 6.15 | 2.90 | 1.70 | 0.31 | 1.08% |
| 21 | 3.77 | 3.83 | 3.08 | 0.93 | 1.77 | 0.79% |
| 22 | 3.94 | 8.01 | 2.55 | 0.89 | 1.22 | 1.04% |
| 23 | 4.08 | 0.94 | 3.42 | 1.62 | 0.45 | 0.94% |
| 24 | 4.31 | 8.50 | 3.36 | 1.39 | 1.57 | 1.17% |
| 25 | 4.34 | 5.46 | 4.52 | 1.84 | 1.29 | 0.67% |
| 26 | 4.53 | 3.10 | 1.81 | 1.96 | 0.77 | 1.41% |
| 27 | 4.73 | 4.57 | 2.84 | 1.51 | 1.50 | 1.14% |
| 28 | 4.91 | 4.74 | 2.17 | 1.47 | 0.38 | 0.58% |

*Twenty-eight parameter combinations generated by Latin hypercube sampling for the joint probabilistic uncertainty analysis. The jointly sampled parameters were the mean reproduction number ($\mu_R$), the overdispersion parameter of the offspring distribution ($k$), the shape and scale parameters of the gamma-distributed serial interval, the sigmoid scaling parameter used in the migration probability function, and the baseline outbound migration probability for Shanghai. The panel_id identifies the corresponding numbered panel in Supplementary Figs X-Z.*

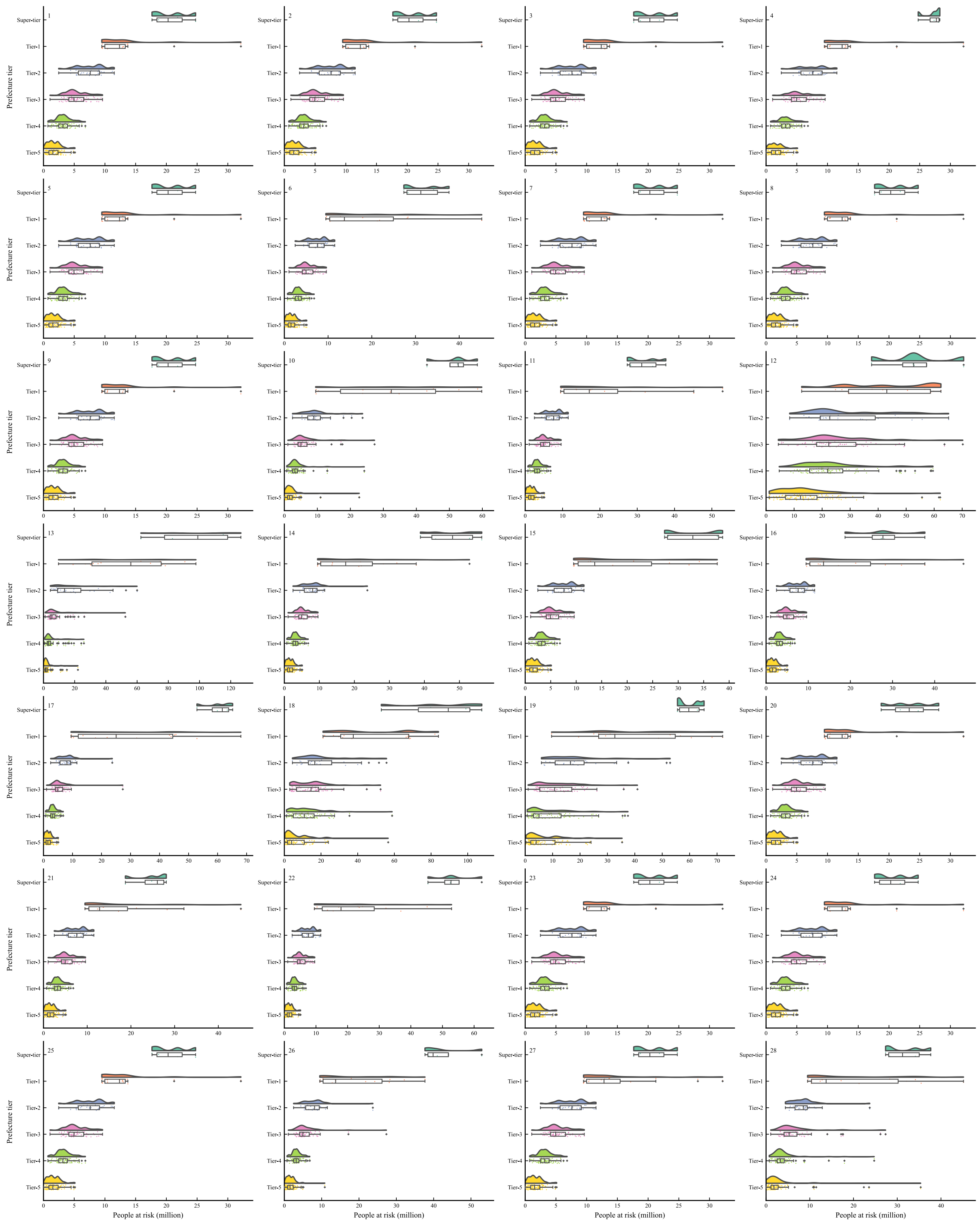


***Fig X. Population at risk across prefecture tiers under joint parameter uncertainty: 7-day threshold.***

*Raincloud plots showing the estimated population at risk across prefecture tiers for each of the 28 Latin hypercube-sampled parameter sets when risk was defined using the 7-day threshold. Each numbered panel corresponds to one sampled*

*parameter set listed in Supplementary Table D. The figure is presented using the same analytical framework in the main text to facilitate direct comparison with the baseline analysis. Across parameter sets, the absolute magnitude of risk varied, whereas the broad tier-structured spatial pattern remained similar.*

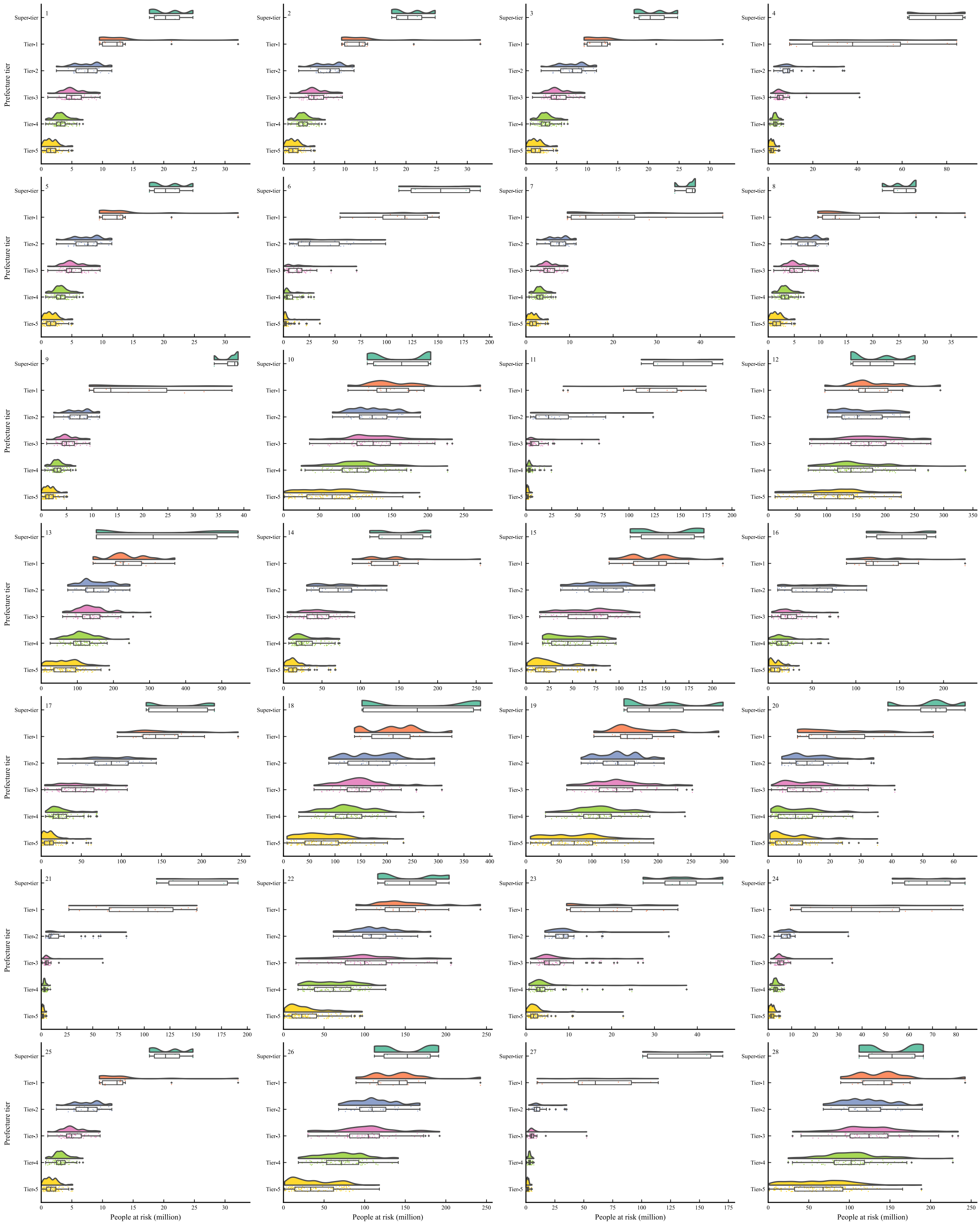

***Fig Y. Population at risk across prefecture tiers under joint parameter uncertainty: 14-day threshold.***

*Raincloud plots showing the estimated population at risk across prefecture tiers for each of the 28 Latin hypercube-sampled parameter sets when risk was defined using the 14-day threshold. Each numbered panel corresponds to one sampled parameter set listed in Supplementary Table D. The overall contrast between higher-tier and lower-tier cities remained apparent across sampled parameter sets despite variation in absolute risk estimates.*

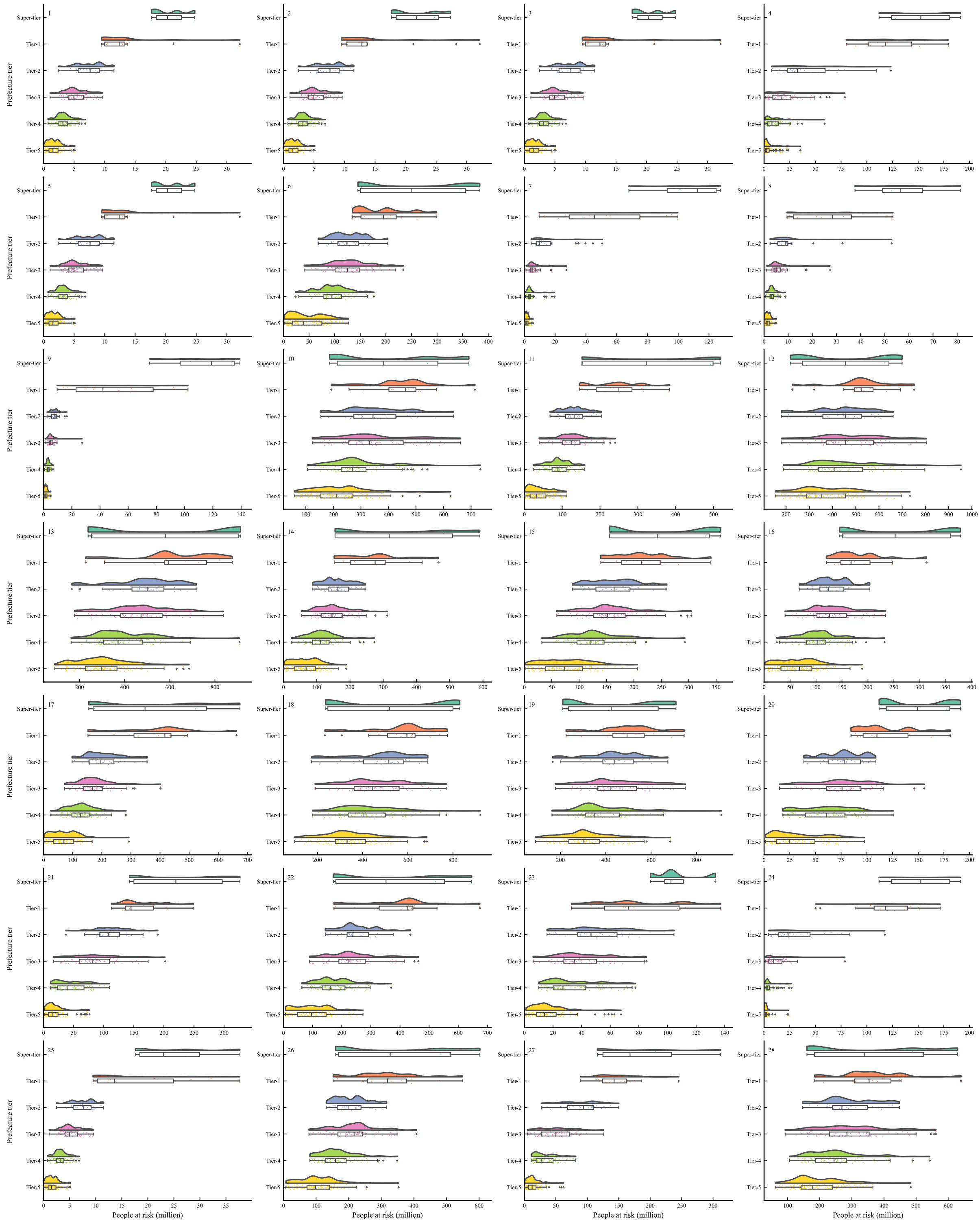


***Fig Z. Population at risk across prefecture tiers under joint parameter uncertainty: 21-day threshold.***

*Raincloud plots showing the estimated population at risk across prefecture tiers for each of the 28 Latin hypercube-sampled parameter sets when risk was defined using the 21-day threshold. Each numbered panel corresponds to one sampled*

*parameter set listed in Supplementary Table D. Although uncertainty in the jointly sampled parameters affected the magnitude of estimated risk, the overall tier-specific pattern remained broadly consistent with that in the baseline analysis.*

10. Although extensive supplementary material is provided, several methodological elements critical to interpretation (e.g., the log-log correlation rationale, the construction of mobility transition matrices, and details of sensitivity analyses) are only described in the supplement. Given their importance, these should be more explicitly summarised in the main Methods section to ensure transparency for readers who may not consult the supplementary files in detail.

    **Response**: We thank the reviewer for this important comment on methodological transparency. We agree that key methodological elements should be sufficiently described in the main Methods section, and we have revised the manuscript accordingly. We agree that several methodological elements that are important for interpreting the results should not rely solely on the Supplementary Material. In response, we have revised the main Methods section to provide a clearer summary of three components that were previously described mainly in the supplement: (1) the rationale for using correlations between log-transformed observed and predicted first arrival times, (2) the construction of the mobility transition matrix from Baidu migration data, and (3) the scope and purpose of the sensitivity and uncertainty analyses.

    Specifically, in the revised main Methods, we now explicitly explain why the comparison between observed and predicted first arrival times was performed on the log scale: to compare the similarity between the observed and predicted arrival time patterns across cities and to preserve information about their relative temporal spacing. We also added a concise description of how daily intercity mobility data were converted into transition probabilities used in the simulation, including the role of the migration matrix and the scaling assumptions applied in the model. In addition, we now summarise the sensitivity analyses in the main text, including both the original one-way sensitivity analyses and the newly added joint probabilistic uncertainty analysis based on Latin hypercube sampling.

The Supplementary Material still contains the full technical details, parameter specifications, and additional figures. Still, the main Methods now include the information readers need to understand the key modelling choices without relying solely on the appendix. We have revised the manuscript accordingly, and these revisions ensure that the main Methods section now contains the essential information required to understand the modelling framework and key assumptions without relying on the Supplementary Material.

**Methods (Page 13, Lines 290-299)**

*We compared the simulated first arrival-time pattern generated by each candidate origin with the observed first arrival-time pattern across affected cities. This comparison was performed using the Pearson correlation between the logarithm of the predicted first arrival time ($lg\,T_{pre}$) and the logarithm of the observed first arrival time ($lg\,T_{obs}$). We used logarithmically transformed first arrival times for the mobility-based analyses because the relationship between the diffusion time ($t$) and diffusion distance ($D$) follows a power-law form $t \sim D^{\alpha}$. In the origin-identification analysis, Pearson correlation on log-transformed arrival times was used primarily to compare the similarity between observed and predicted arrival time patterns across cities and to preserve information about their relative temporal spacing.*

**Methods (Page 7, Lines 159-165)**

*For each city and day, we obtained both the outbound migration scale index and the proportion of outflow directed to each destination city. We combined these two components to estimate city-to-city mobility flows and then normalised these flows to derive travel probabilities between origin-destination pairs. These probabilities were assembled into city-to-city mobility transition matrices, which were used to parameterise intercity movement in the transmission model.*

**Methods (Page 15, Lines 356-362)**

***Sensitivity and uncertainty analyses***

*We assessed robustness through one-way sensitivity analyses and an additional joint probabilistic uncertainty analysis. In the latter, key parameters governing the reproduction number distribution, serial interval distribution, and migration probabilities were jointly sampled using Latin hypercube sampling to evaluate whether the main spatial risk patterns were robust to simultaneous uncertainty in model inputs.*

Overall, these revisions improve transparency while maintaining the Supplementary Material as a resource for full technical detail.

**Minor Comments**

1. The terminology used for reproduction numbers alternates between "effective reproduction number" ($R_e$) and "basic reproduction number" ($R_0$ / R*) across sections and pathogens. A consistent notation and clearer explanation of why different measures are used for Omicron and Influenza A would improve clarity.

   **Response**: We thank the reviewer for this helpful suggestion. We agree that the use of different reproduction number terms across sections could be confusing. We have revised the manuscript to use a more consistent notation and to clarify the epidemiological meaning of each quantity. The choice of reproduction number terminology was aligned with the role of the parameter in each analysis rather than fixed across all scenarios.

   Specifically, we now distinguish clearly between the basic reproduction number ($R_0$), which refers to transmission potential in a fully susceptible population without interventions, and the effective or context-specific reproduction number ($R_e$), which reflects realised transmission under prevailing immunity, behavioural responses, and control measures. In the revised manuscript, we use the same notation consistently throughout and explicitly explain why different quantities are used for different pathogens/scenarios.

   For the COVID-19 Omicron validation analyses, the reproduction number corresponds to transmission under the control context in which the observed outbreaks occurred; therefore, we refer to it as an effective reproduction number rather than a basic

reproduction number. By contrast, for the hypothetical Influenza A simulations, the parameter is used primarily to represent the intrinsic transmissibility assumed in the modelled scenario, and we now define this more explicitly in the Methods to avoid ambiguity.

**Methods (Page 12, Lines 265-271)**

*We defined reproduction numbers according to their epidemiological interpretation:* $R_0$ *denotes the basic reproduction number in a fully susceptible population without interventions, whereas* $R_e$ *denotes the effective reproduction number under prevailing epidemiological and control conditions. For the COVID-19 Omicron validation analyses, we therefore referred to* $R_e$. *For the hypothetical Influenza A simulations, the reproduction parameter represents an assumed baseline transmissibility parameter analogous to* $R_0$ *under the modelled conditions.*

2. The serial interval distributions are specified with point estimates for parameters, but it is unclear whether uncertainty in these parameters was incorporated into the simulations or treated as fixed. Clarifying this would improve transparency.

<u>**Response**</u>: We thank the reviewer for this important comment. In the baseline simulations, serial interval parameters were treated as fixed, while uncertainty in these parameters was explored separately through sensitivity and joint uncertainty analyses.

In the baseline analyses, the serial interval distribution for each pathogen was specified using point estimates for its parameters obtained from the literature. Within each simulation, serial intervals for individual transmission events were randomly drawn from the specified distribution, thereby incorporating stochastic variability in transmission timing. Thus, stochastic variation in individual-level transmission timing was incorporated in every simulation run.

Uncertainty in the parameters of the serial interval distribution was addressed separately in sensitivity analyses. In particular, in the joint uncertainty analysis, we sampled alternative combinations of serial interval parameters over predefined plausible ranges and repeated the simulations under these alternative distributions. We have revised the

Method to make this distinction explicit. This approach ensures that both stochastic variability and parameter uncertainty in serial intervals are accounted for within the overall modelling framework.

**Methods (Page 12, Lines 272-277)**

*For the baseline analyses, serial interval distributions were parameterised using literature-based point estimates. In each simulation, serial intervals for individual transmission events were randomly sampled from these distributions. Parameter uncertainty in the serial interval distributions was examined separately in sensitivity analyses, including the joint uncertainty analysis, in which alternative parameter combinations were sampled over plausible ranges.*

3. The Methods section would benefit from a concise schematic or algorithmic description (e.g., stepwise outline or pseudocode) of the simulation process, particularly for readers less familiar with agent-based branching process models.

   **Response**: We thank the reviewer for this suggestion. In response, we have added a concise pseudocode description of the simulation process. This schematic description clarifies the sequence of stochastic transmission and spatial movement steps and makes the simulation logic more accessible to readers unfamiliar with agent-based branching process models. The added algorithm summarises the branching process used to generate transmission lineages, including the stochastic generation of secondary cases from a negative binomial distribution and the assignment of infection times by sampling serial intervals from a gamma distribution. We have also expanded the description to clarify how spatial spread is incorporated through the mobility transition matrix and how first arrival times are recorded for each city. We believe this addition makes the modelling workflow more transparent and easier to follow without substantially increasing the length of the main text.

**Methods (Page 9, Lines 216-218)**

*To improve transparency, the core steps of the spatial agent-based branching process are summarised in the following pseudocode.*

***Algorithm: Spatial agent-based branching process simulation***

*Input: seed city o, initial infections $N_0$, time window W, offspring distribution, serial interval distribution, mobility matrix P*
*Output: first arrival time FA(c) for each city c*

*Initialise $N_0$ infections in city o on day 1. Set FA(o) = 1 and FA(c) = undefined for all other cities. Add initial infections to queue Q*

```
while Q is not empty do
   Select one infected individual i from Q
   Draw the number of secondary infections r
   for each secondary infection do
      Draw serial interval Δ
      Set t = day(i) + round(Δ)
      Assign destination city by sampling from the mobility transition probabilities P
      if FA(c) is undefined then FA(c) = t
      if t ≤ W then add offspring (t, c) to Q
   end for
end while
```

*Return FA(c)*
*Repeat the above process over multiple stochastic simulations and summarise the resulting first arrival times for each city*

4. The assumption of 100 initial cases for risk simulations is somewhat arbitrary. Although alternative values are explored in sensitivity analyses, a brief justification for this choice in the main text would be helpful.

   **Response**: Thank you for this helpful comment. We agree that the choice of 100 initial cases is a modelling assumption rather than an empirically fixed value. In the main analysis, we assumed 100 initial cases as a standardised seeding condition for the risk simulations to facilitate comparison of relative transmission risk across cities. This choice also avoids excessive stochastic extinction in early simulations while remaining within a realistic order of estimate for early outbreak seeding. Sensitivity analyses using alternative initial case numbers yielded similar overall results. We further assessed the impact of this assumption in sensitivity analyses using alternative numbers of initial cases, and the overall spatial risk patterns and main conclusions remained materially unchanged. We have now added a brief justification for this choice in the main text and referred readers to the sensitivity analyses. **(Methods, Page 14, Lines 328-331)**

   *We assumed 100 initial cases as a standardised seeding condition for the risk simulations to enable stable and comparable estimates of relative transmission risk*

*across cities and to reduce the influence of stochastic extinction in early transmission chains; sensitivity analyses using alternative initial case numbers showed similar overall patterns.*

5. The description of the prefecture-tier classification relies on an externally defined commercial ranking system. While this is reasonable, the potential mismatch between commercial attractiveness and epidemiological vulnerability could be acknowledged more explicitly as a limitation of the stratification approach.

   **Response**: We thank the reviewer for this helpful point. We agree that the prefecture-tier classification reflects a city's commercial and functional hierarchy rather than a direct measure of epidemiological vulnerability. We used this classification as a pragmatic proxy for urban scale and intercity connectivity, which are the primary structural drivers of early spatial diffusion examined in this study. Nevertheless, this approach may not capture other dimensions relevant to vulnerability to epidemic spread, including demographic structure, healthcare capacity, housing conditions, and local response capacity. We have now acknowledged this more explicitly as a limitation in the Discussion.

   **Discussion (Page 29, Lines 684-691)**

   *Additionally, although the prefecture-tier classification captures broad differences in urban scale and connectivity, it is based on commercial and functional characteristics. It may not fully capture epidemiological vulnerability, which depends on additional factors such as demographic structure, healthcare capacity, housing conditions, socioeconomic context, and local public health response capacity. However, because our analysis focuses on mobility-driven early spatial diffusion, the use of this classification is unlikely to materially affect the main conclusions regarding cross-tier differences in transmission risk.*

6. The handling of cities that do not experience any introductions during the simulation period is not clearly described. It would be useful to clarify whether such cities are censored, assigned infinite arrival times, or excluded from certain analyses.

   **Response**: We thank the reviewer for this important comment. We agree that the treatment of cities with no simulated introductions during the analysis period should be described more clearly. In our simulations, if a city received no infected arrivals within the

simulation window, no finite first arrival time was assigned; these cities were treated as not reached within the observation period rather than being assigned an arbitrarily large or infinite value. For analyses that compared observed and predicted first arrival times, we restricted the comparison to cities with a defined observed first arrival time and a finite model-predicted first arrival time within the study period. Cities not reached in the simulation period were therefore excluded from these correlation analyses. This restriction ensures that the validation focuses on the relative timing of observed introductions rather than being influenced by structurally undefined or right-censored arrival times. We have now clarified this point in the Supplementary Material. **(Page 5, Lines 88-92).**

*If a city did not experience any infected introduction during the simulation period, it was considered not reached within the observation window, and no finite first arrival time was assigned, consistent with right-censoring within the finite simulation horizon. Analyses comparing observed and predicted first arrival times were restricted to cities with both an observed first arrival time and a finite simulated first arrival time within the study period.*

7. Minor editorial clarification is needed to distinguish clearly between validation analyses using observed COVID-19 outbreaks and hypothetical simulations for Influenza A, as the transition between these sections may be confusing for some readers.

   **<u>Response</u>**: We thank the reviewer for this helpful suggestion. We agree that the distinction between the validation analyses based on observed COVID-19 outbreaks and the hypothetical simulations for Influenza A should be made clearer. In the revised manuscript, we have strengthened the transition between these sections and clarified their different purposes. We also introduced explicit signposting to distinguish clearly between the validation stage and the subsequent scenario-based analyses. Specifically, we now state more explicitly that the observed COVID-19 outbreaks were used to evaluate the model's ability to reproduce real-world spatial diffusion patterns. In contrast, the Influenza A simulations were intended as hypothetical applications of the validated framework to compare transmission risk across urban tiers under a different respiratory infection

scenario. We have also revised the relevant introductory and transitional sentences to make this distinction clearer for readers.

**Methods (Page 11, Lines 257-261).**

> *We first used observed COVID-19 outbreaks to validate the model's ability to reproduce real-world spatial diffusion patterns. We then applied the validated framework to Influenza A as a hypothetical scenario to illustrate its use in comparing spatial transmission risk across urban tiers under a different respiratory infection context.*

**Reviewer 4**

In this paper, the authors develop an agent-based branching process model that incorporates mobility to study the movement of respiratory pathogens through hierarchical urban/rural areas. They apply their model to SARS-CoV-2 Delta and Omicron variant outbreaks in China, and also influenza outbreaks in China. As the authors highlight, while the use of mobility data to ground dynamic transmission models has advanced, we have few comparative-level analyses looking across different pathogen types and/or variants with meaningfully different epidemiological parameters. The authors identify differences between the two SARS-CoV-2 variants and influenza, and account for them mechanistically by examining how these pathogens spread. The comparisons provided are a real strength, differentiating the work from much of what has been done previously. Additionally, and just as importantly, the authors also move beyond simple single-parameter descriptions of outbreaks, i.e., R0, and include overdispersion in their analysis. I do have four substantive concerns.

**Response**: We thank the reviewer for this constructive and balanced assessment of our work. We are encouraged that the reviewer recognised the value of the comparative framework and of incorporating transmission heterogeneity beyond simple reproduction-number summaries. We have carefully considered the four substantive concerns raised and revised the manuscript accordingly to clarify model assumptions, strengthen validation and uncertainty analyses, and better position the study's contribution within the existing literature. Detailed, point-by-point responses are provided below.

1. First, the approach does not seem to account for the existing immunological landscape or potential climate-related seasonal drivers of influenza transmission. Regarding prior immunity, despite China's largely successful COVID-zero strategy, sporadic outbreaks still occurred. The authors should discuss the landscape of prior infections in the cities under investigation. Relatedly, while I know there were concerns about whether the vaccine would protect against Omicron infection, there was no discussion of how to account for pre-existing vaccination. Regarding COVID-zero, my understanding is that the policy was largely abandoned when Omicron began spreading. How should we compare/contrast the Delta outbreaks (when intensive interventions were used to control transmission,

including mobility restrictions) vs Omicron? Regarding influenza, the landscape of immunity from COVID was quite complicated in many countries, including the US. Were there similarly atypical patterns of influenza in China that would have impacted the immunological landscape? I know aspects like immunity are likely why the authors focused on arrival time. Still, the authors should discuss what we know about how mobility, interventions, immunity, climate, urban/rural, etc., might interact to drastically change the results if we consider final attack rate, peak height, epidemic intensity, or other measures of how the outbreak would unfold.

**Response**: We thank the reviewer for this thoughtful and constructive comment, and we appreciate the reviewer's recognition that these considerations motivated our focus on first-arrival timing. We agree that prior infection, vaccination, intervention intensity, climatic conditions, and urban-rural heterogeneity are all important determinants of epidemic dynamics, and that if the outcome of interest were peak incidence, cumulative incidence, epidemic intensity, or final attack rate after introduction, these factors could substantially influence both the magnitude of local outbreaks and the relative ranking of cities and pathogens. We have expanded the Methods and Discussion to make this distinction more explicit and to address each point below.

Regarding the specific points raised:

1) Prior infection landscape

    We agree that sporadic outbreaks under the dynamic zero-COVID strategy could still have generated some local heterogeneity in infection-acquired immunity. At the same time, available evidence suggests that, before late 2022, cumulative SARS-CoV-2 infection in China remained low overall compared with many high-incidence settings. Serological studies from Chinese cities reported very low SARS-CoV-2 seroprevalence outside the earliest epicentre [29], supporting the view that infection-derived immunity remained limited overall before the large Omicron wave [30]. We have therefore revised the Discussion to acknowledge both points: first, that some local heterogeneity in infection-acquired immunity may still have existed because of sporadic outbreaks; and second, that such heterogeneity was likely more relevant to post-arrival local transmission intensity and outbreak size than to the earliest importation-driven patterns captured by first arrival timing. We also note that we

were unable to identify harmonised city-specific seroprevalence estimates for all study cities and periods, so the available evidence is more informative about the overall national context than about directly comparable infection-acquired immunity levels across all investigated cities.

2) Pre-existing vaccination

We agree that pre-existing vaccination should be discussed more explicitly. Available evidence suggests that COVID-19 vaccine coverage in China was already high during the study period. However, the most directly comparable data are available mainly at the national, provincial, or selected-city levels rather than as harmonised city-level estimates across all study locations. For example, province-level analyses documented high coverage by late 2021, with particularly high uptake in Beijing and Shanghai[31], and official reports indicated that vaccination had already covered about 90% of the population by spring 2022. Studies from Shanghai further reported primary vaccination coverage above 90% during the 2022 Omicron BA.2 outbreak, although booster uptake and vaccination coverage among older adults were lower [32,33]. We have therefore revised the manuscript to clarify that, while vaccination was not explicitly parameterised in the arrival-time framework, this was not because vaccination was unimportant, but because comparable city-specific vaccination data were not available for all study cities and because vaccination would be expected to affect post-introduction local amplification and burden more strongly than the earliest importation event itself.

3) Delta versus Omicron under an intervention context

We appreciate this point and agree that comparisons between Delta and Omicron need to be interpreted in light of the intervention context. We have revised the manuscript to make this clearer. Specifically, the comparison in our study should not be understood as one between outbreaks occurring under intensive control for Delta and a broadly abandoned control strategy for Omicron. Rather, the Delta outbreaks examined here and the Shanghai Omicron outbreak both occurred under the broader dynamic zero-COVID framework. During the Shanghai 2022 Omicron outbreak, China's dynamic zero-COVID strategy remained in place, and extensive movement restrictions and lockdown measures were implemented in response to transmission[33,34]. The more relevant contrast is therefore between pathogens with

different transmissibility and generation characteristics under a broadly comparable containment framework, rather than between entirely different policy regimes. At the same time, the substantially higher transmissibility of Omicron meant that similar containment strategies were likely to face greater pressure and could therefore result in different realised transmission patterns. We now state this more explicitly in the Discussion.

4) Atypical influenza patterns in China

   We agree that this is an important issue. As in other countries, influenza circulation in China was substantially disrupted during the COVID period, indicating that the immunological landscape during the study period was unlikely to reflect a typical pre-pandemic seasonal steady state. Recent studies from China describe unusually low influenza activity during the period of stringent COVID-related measures, followed by a resurgence and altered seasonal patterns, including reports of out-of-season influenza activity [35-37].

5) How mobility, interventions, immunity, climate, and urban-rural heterogeneity might interact if other outcomes were considered

   Beyond first-arrival timing, peak incidence, cumulative incidence, epidemic intensity, and final attack rate, these metrics are more sensitive to local heterogeneity in susceptibility, vaccine-derived protection, intervention intensity, climatic suitability, and urban context. This is especially plausible for influenza, for which studies in China have shown associations with temperature and humidity [38,39], and for which urbanisation has been linked to differences in transmission dynamics [40,41]. We have therefore revised the Discussion to emphasise that our main findings should be interpreted as reflecting comparative early-diffusion potential rather than comparative local epidemic severity.

To illustrate this contrast concretely, we include a simple post-arrival analysis examining how intervention intensity, immunity, climate, and urban context may influence epidemic outcomes after introduction (Supplementary Methods and Results). This analysis was not intended to reproduce observed epidemic dynamics, but to demonstrate that post-arrival outcomes are substantially more sensitive to these factors than first-arrival timing.

We added a clarifying sentence in the Methods to explain why the first arrival timing was used as the primary outcome. **(Methods, Page 11, Lines 246-254).**

*First-arrival time was chosen as the primary validation target because it matches the policy frame our evidence is intended to inform: in China, responses to respiratory epidemics are typically enacted at the city level and initiated by a local trigger, so the operationally meaningful question for city-level practitioners is whether an outbreak has arrived—not what proportion of the country is affected or how fast it is spreading spatially. First-arrival timing also more directly reflects intercity diffusion on the mobility network and is less confounded by city-specific differences in prior infection, vaccination, local interventions, and climatic conditions than metrics based on peak incidence, cumulative incidence, epidemic intensity, or final attack rate.*

We expanded the Discussion to address the likely roles of prior infection, vaccination, intervention context, atypical influenza circulation in China during the COVID period, and the expected influence of climate and urban context on post-arrival epidemic outcomes. **Discussion (Page 27, Lines 620-653)**

*Our findings should be interpreted as reflecting comparative early spatial diffusion potential, rather than comparative local epidemic severity after introduction. We focused on first arrival timing because it is more directly linked to the mobility network, transmission generation characteristics, and stochastic seeding process during the earliest phase of spread, and is therefore less sensitive than outcomes such as peak incidence, cumulative incidence, epidemic intensity, or final attack rate to city-specific heterogeneity in prior infection, vaccination, climatic forcing, and the timing and intensity of local interventions. These factors remain important for epidemic growth after introduction. Available evidence suggests that widespread prior SARS-CoV-2 infection in China remained limited overall before late 2022, although sporadic outbreaks could still have generated some local heterogeneity in infection-acquired immunity. Likewise, COVID-19 vaccine uptake in China was high during the study period, but harmonised city-specific vaccination*

*data were not available across all study locations and time periods. The comparison between the Delta outbreaks and the Shanghai Omicron outbreak should also be interpreted within the broader dynamic zero-COVID framework, rather than as a contrast between intensive control for Delta and broadly abandoned control for Omicron. Influenza circulation in China was also substantially perturbed during the COVID period, with delayed resurgence and altered seasonal patterns, suggesting that the immunological landscape was unlikely to represent a typical pre-pandemic seasonal steady state. Taken together, these considerations suggest that incorporating the infection landscape, vaccination landscape, climatic drivers, and heterogeneous interventions would be essential for studies of peak size, epidemic intensity, or final attack rate, but is less critical for the arrival-time-based question addressed here.*

*We did not explicitly model city-specific prior infection, vaccination coverage, time-varying intervention intensity, or climatic forcing. This was a deliberate choice because our objective was to study early intercity diffusion and first arrival timing rather than local epidemic burden after introduction. If the framework were extended to outcomes such as peak incidence, cumulative incidence, epidemic intensity, or final attack rate, these factors would need to be incorporated explicitly. They could alter the relative ranking of cities and pathogens. An exploratory post-arrival local transmission analysis was therefore added in the Supplementary Material to illustrate how these factors could influence local epidemic trajectories once introduction had occurred.*

We also added an exploratory supplementary post-arrival local transmission analysis **(Supplementary Material, Page 10, Lines 310-354)** based on a simplified city-level SIR model to illustrate how intervention intensity, immunity, climate, and prefecture tier could affect peak size, cumulative incidence, and epidemic intensity once introduction had occurred.

**Supplementary Material (Page 15, Lines 499-516)**

***Exploratory post-arrival local transmission analysis***

*In the exploratory post-arrival local transmission analysis, introducing intervention, immunity, climate, and tier modifiers had a limited effect on the interpretation of first arrival timing but a much larger effect on post-arrival epidemic outcomes. Across scenarios, peak size and cumulative incidence varied substantially with the assumed levels of control and pre-existing immunity, as well as with climatic conditions and prefecture tier, further modulating these differences.*

*In general, stronger interventions and higher pre-existing immunity were associated with lower peak size and cumulative incidence across scenarios. Climatic conditions and prefecture tier further modified the magnitude of local outbreaks. Under several scenario combinations, the ranking of prefecture tiers based on post-arrival outcomes differed from the ranking implied by the early diffusion analysis alone, indicating that local susceptibility, intervention intensity, and environmental suitability can become increasingly important once transmission is established.*

*These findings support the rationale for using first arrival timing as the primary outcome in the main analysis: arrival-based metrics are comparatively robust for studying intercity diffusion, whereas post-arrival outcomes such as peak size and cumulative incidence are more sensitive to local epidemiological and environmental heterogeneity.*

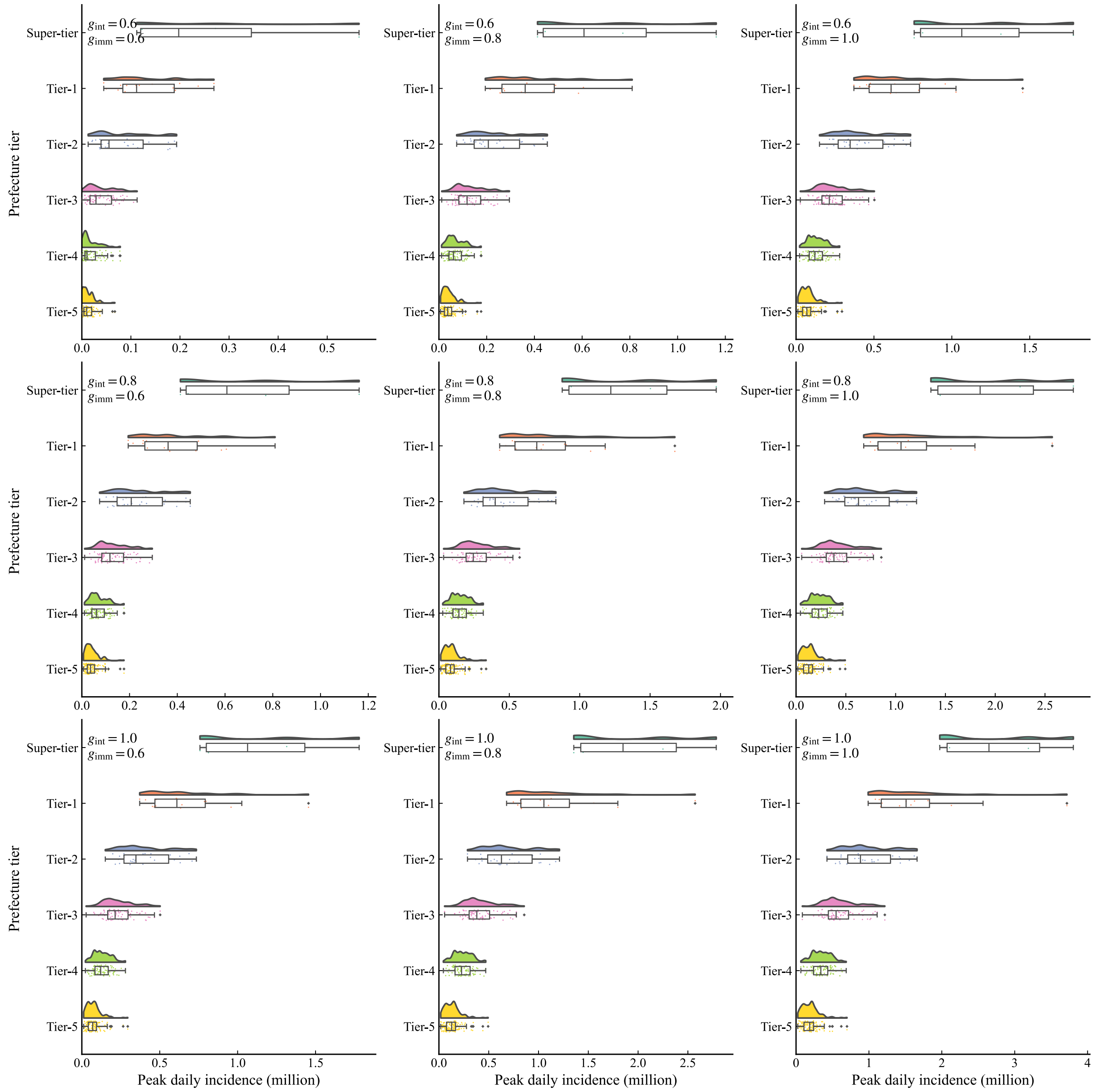


***Fig AA. Exploratory post-arrival local transmission analysis of peak daily incidence under alternative intervention, immunity, climate, and prefecture-tier scenarios.***

*For each city, the first arrival time was determined using the main intercity diffusion model, after which local transmission was simulated using a simplified city-level SIR model. Panels show variation in peak daily incidence across combinations of three intervention scenarios ($g_{int}$=0.6,0.8,1.0), three immunity scenarios ($g_{imm}$ =0.6,0.8,1.0), climatic modulation based on standardised temperature and relative humidity, and prefecture-tier modifiers. This analysis was intended to illustrate how factors that were not explicitly included in the arrival-time framework could*

*influence post-introduction epidemic trajectories, rather than to reconstruct observed city-specific epidemic burden.*

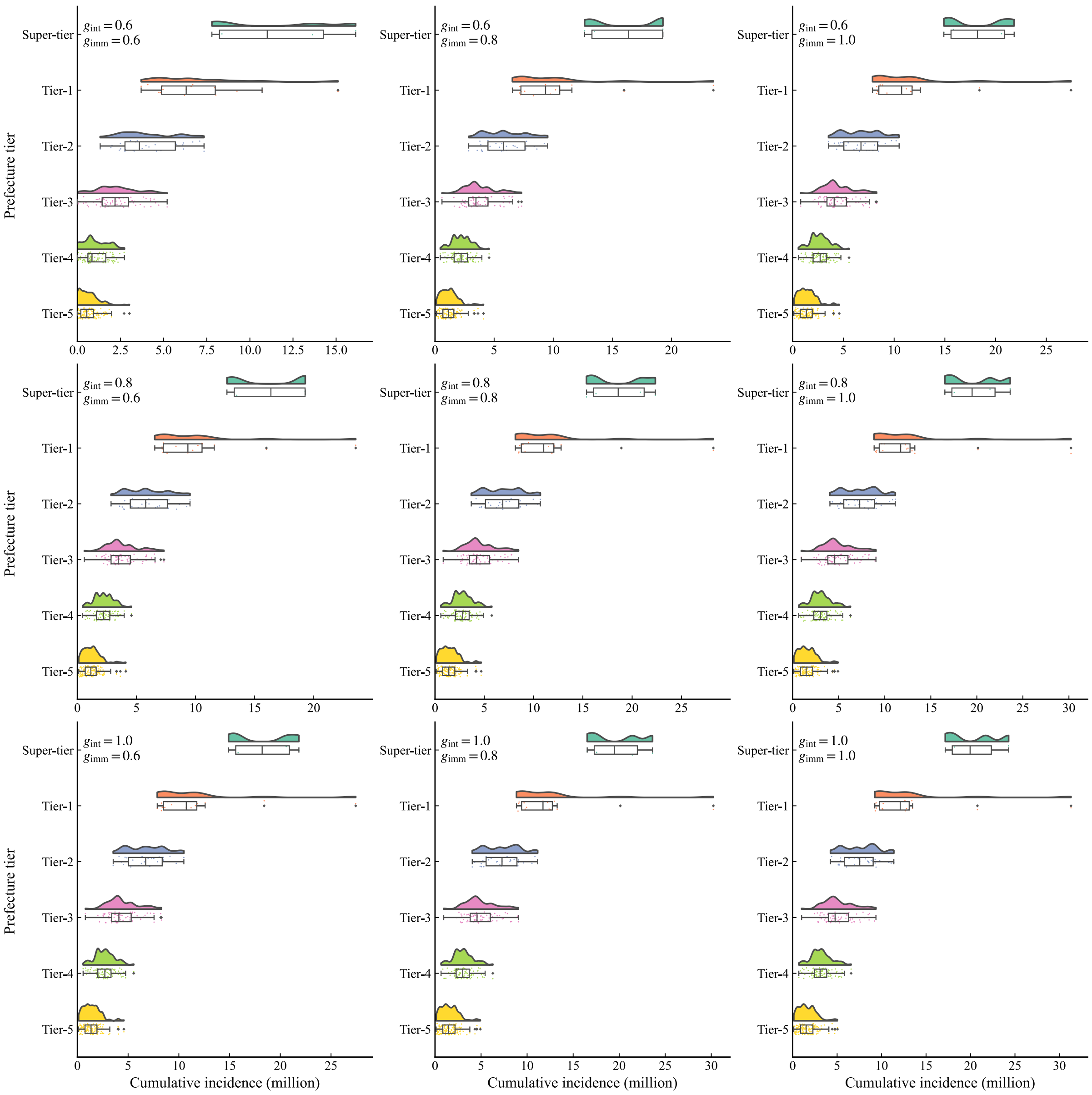


***Fig BB. Exploratory post-arrival local transmission analysis of cumulative incidence under alternative intervention, immunity, climate, and prefecture-tier scenarios.***

*For each city, the first arrival time was determined using the main intercity diffusion model, after which local transmission was simulated using a simplified city-level SIR model. Panels show variation in cumulative incidence across combinations of three intervention scenarios ($g_{int}$=0.6,0.8,1.0), three immunity scenarios ($g_{imm}$*

*=0.6,0.8,1.0), climatic modulation based on standardised temperature and relative humidity, and prefecture-tier modifiers. This analysis was intended to illustrate how factors that were not explicitly included in the arrival-time framework could influence post-introduction epidemic trajectories, rather than to reconstruct observed city-specific epidemic burden.*

2. Second, if it weren't for the overdispersion, a criticism would be that all the results boil down to pathogens with higher R0 and lower generation time spreading faster. I think what the authors are doing is much more interesting than that, but focusing exclusively on arrival time makes it a bit hard to determine what would happen if we looked at some of the measures I mentioned in the previous section. Perhaps most critically, Mercier et al. (2022) showed how ~90% of mobility could be ignored in the US and simulated SIR epidemics could be entirely recreated, not just the arrival times (which they also considered). One possible middle ground (because I agree that moving beyond arrival time would raise more challenges than it would address) would be to examine the distribution of estimated arrival times and fit a hierarchical or mixed-effects model that accounts for uncertainty in arrival times. Mercier et al. (2022) show that distributions of arrival times can be more diagnostic.

   **<u>Response</u>**: We thank the reviewer for this thoughtful suggestion. We agree that reliance on single summary estimates of first arrival times may obscure the stochastic nature of spatial spread and could make differences between pathogens appear primarily driven by transmissibility or generation time.

   Following this suggestion, we extended our analysis by explicitly characterising the distribution of arrival times derived from repeated stochastic simulations. Because each city contributes only one observed arrival time, direct distributional comparison at the city level is limited. We therefore complement the validation framework with distributional summaries of simulated arrival times across stochastic runs.

   In the Shanghai outbreak, the distribution of simulated arrival times was broadly consistent with observed values (observed: mean 23.25 days, median 26.00 days, IQR

14.00; simulated: mean 21.80 days, median 24.82 days, IQR 9.16), with a modest tendency toward earlier, less dispersed arrival times in simulations. In the mixed-effects model, arrival time was strongly associated with mobility connectivity, spatial distance, and city population size, while substantial between-city variability remained. After adjustment for these factors, the Shanghai scenario was associated with systematically longer arrival times relative to the Nanjing scenario, indicating that differences in spatial spread cannot be fully explained by simple transmission parameters alone, but reflect the interaction between pathogen-specific dynamics and the mobility network structure.

To further account for uncertainty in simulated arrival times, we fitted a mixed-effects model in which city-specific arrival times from repeated simulations were treated as stochastic outcomes. This approach allows the distribution of arrival times, rather than a single summary statistic, to inform inference while accounting for between-city heterogeneity.

Together, these additional analyses suggest that our findings are not driven solely by simple transmission parameters but reflect the interaction between pathogen-specific dynamics and the structure of the mobility network. We have revised the supplementary to make this explicit.

**Supplementary Material (Page 5 Lines 114-139)**

***Arrival time distribution and mixed-effects modelling***

*To account for stochastic variability in spatial spread, we analyzed arrival times obtained from repeated stochastic simulations of each outbreak scenario. For each simulation run, the first arrival time was defined as the earliest time at which infection was introduced into a given destination city. Analyses were restricted to city-simulation pairs in which the city was reached within the simulation window, consistent with prior simulation-based approaches that condition on successful transmission.*

*We summarised the empirical distribution of observed first arrival times across cities and compared it with the distribution of simulated arrival times aggregated*

*across stochastic runs. Summary statistics included the mean, median, and interquartile range.*

*To formally incorporate uncertainty in arrival times, we fitted a mixed-effects model with log-transformed arrival time as the dependent variable:*

$$log(T_{ijr}) = \beta_0 + \beta_1 Pathogen_i + \beta_2\, log(Migration_{ij} + c) + \beta_3 \mathbb{I}(Migration_{ij} = 0) + \beta_4 log(Geodesic\ distance_{ij}) + \sum_{k=2}^{6} \gamma_k \mathbb{I}(Tier_j = k) + \beta_5 log(Population_j) + u_j + \varepsilon_{ijr}$$

*where,* $T_{ijr}$ *denotes the first arrival time for destination city* $j$ *in simulation run* $r$ *under scenario* $i$*;* $Pathogen_i$ *indicates the outbreak variant (Shanghai Omicron vs Nanjing Delta);* $Migration_{ij}$ *represents population flow from the origin city to destination city* $j$*, with a small constant* $c$ *added to allow log transformation;* $\mathbb{I}(Migration_{ij} = 0)$ *is an indicator for zero-flow connections;* $Geodesic\ distance_{ij}$ *is the distance between origin and destination cities;* $Tier_j$ *denotes the prefecture tier of the destination city;* $Population_j$ *is the resident population size of the destination city;* $u_j$ *is a random intercept for destination city, assumed to follow a normal distribution;* $\varepsilon_{ijr}$ *is the residual error term.*

*This model allows estimation of associations between arrival time and key structural determinants of spread while accounting for repeated simulations and unobserved city-level heterogeneity.*

**Supplementary Material (Page 13 Lines 407-414)**

***Arrival time distributions***

*In the Shanghai outbreak, the distribution of simulated first arrival times across cities was broadly consistent with observed values. However, simulations tended to produce slightly earlier and less variable arrival times. The observed mean arrival time was 23.25 days, with a median of 26.00 days and an interquartile range of 14.00 days. Corresponding simulated values were a mean of 21.80 days, a median of 24.82 days, and an interquartile range of 9.16 days.*

*The overall shape of the simulated distribution aligned with the observed distribution, indicating that the model captures the central tendency of spatial spread but somewhat underestimates dispersion across cities.*

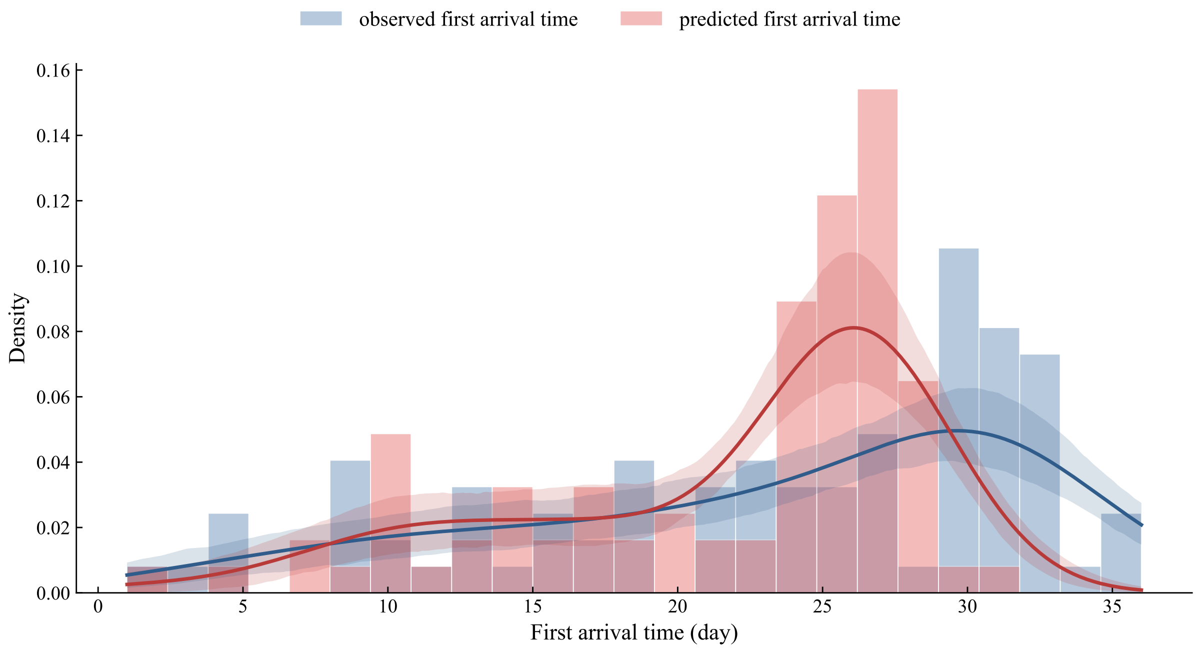


***Fig D. Distribution of observed and simulated first arrival times in the Shanghai outbreak.***

*Density distributions of first arrival times across cities for the Shanghai outbreak. The observed distribution is derived from reported city-level first-detection dates, whereas the simulated distribution is aggregated from repeated stochastic simulations. The simulated distribution shows a similar central tendency to the observed data, with slightly earlier and less dispersed arrival times. Summary statistics are as follows: observed mean 23.25 days, median 26.00 days, interquartile range 14.00 days; simulated mean 21.80 days, median 24.82 days, interquartile range 9.16 days.*

**Supplementary Material (Page 13 Lines 416-422)**

***Mixed-effects model***

*In the mixed-effects model, several structural determinants were strongly associated with arrival time. Higher population flow from the origin city was associated with shorter arrival times (β = −0.059, 95% CI −0.065 to −0.053), while*

*greater geodesic distance was associated with longer arrival times (β = 0.419, 95% CI 0.412 to 0.427). Cities with zero recorded population flow experienced delayed arrival (β = 0.077, 95% CI 0.067 to 0.087). Larger population size was associated with earlier arrival (β = −0.125, 95% CI −0.193 to −0.057).*

3. Third, the use of branching process models to study epidemic transmission across populations with mobility is well established. At least since Colizza and Vespignani (2008), scientists have studied how epidemics unfold in metapopulations using approaches closely related to branching process models. More recently, Jamieson-Lane and Blasius (2020) examined branching processes in the context of pathogen arrival times. The most relevant application is Balcan et al. (2009), who discuss an agent-based branching process model that incorporates mobility. The approach, which they termed GLEAM in Balcan et al. (2010), has been widely used to study many respiratory pathogens, including COVID-19 and influenza. Balcan et al. (2009) specifically used a branching process approximation within their metapopulation framework to derive invasion thresholds—the theoretical relationship between mobility network structure and the probability that a pathogen successfully establishes in a new subpopulation. The authors should carefully discuss how their work extends beyond what has been done by GLEAM (and similar models, see Hunter et al. 2017 for a decent review).

   **<u>Response</u>**: We thank the reviewer for this important comment and for highlighting the substantial literature on mobility-coupled epidemic modelling. We agree that the general framework underlying our study is well-established, and we have revised the manuscript to more clearly acknowledge this work and better position our contribution within this broader modeling tradition.

   More specifically, we distinguish between two levels of positioning. At the level of modelling paradigm, our work builds directly on established mobility-based and branching-process approaches for modelling spatial epidemic spread. At the level of empirical application, however, our study focuses on a more specific inferential setting: reconstructing city-level first arrival patterns, supporting outbreak-origin inference, and comparing early spatial spread across pathogens with different transmission

characteristics. Within this setting, our contributions lie in three complementary dimensions: (1) high-fidelity empirical validation using detailed outbreak data, (2) generation of sub-national, tier-stratified evidence to inform public health decision-making, and (3) direct comparison of spatial diffusion across pathogens with contrasting transmission profiles.

We also appreciate the reviewer's suggestion to discuss the cited studies individually. Below, we clarify how we view each of these contributions in relation to our work and how we have revised the manuscript accordingly.

**Colizza and Vespignani (2008)**

Colizza and Vespignani (2008) provided a foundational theoretical framework for the invasion of epidemics in metapopulation systems, demonstrating how heterogeneous connectivity shapes large-scale spatial spread. Our study builds on this theoretical foundation by moving from abstract invasion dynamics to empirically grounded validation. In particular, by leveraging detailed outbreak data under intensive surveillance conditions, we directly evaluate whether mobility-driven models can reproduce observed first arrival patterns across cities, addressing a gap between theoretical predictions and empirical verification.

**Jamieson-Lane and Blasius (2020)**

Jamieson-Lane and Blasius (2020) characterized the statistical properties of epidemic arrival times within a branching-process framework, highlighting their theoretical relevance for spatial diffusion. We extend this line of work by treating the first arrival time as an empirical validation target and an inferential quantity. Specifically, we use observed arrival patterns to evaluate model performance and to support outbreak-origin identification, thereby linking theoretical arrival-time concepts to practical epidemiological inference.

**Balcan et al. (2009)**

Balcan et al. (2009) demonstrated how multiscale mobility networks shape epidemic spread and established the importance of mobility structure in metapopulation systems. Building on this perspective, our study focuses on the sub-national scale and quantifies how transmission risk is distributed across urban hierarchies within a country. By stratifying cities into tiers and analyzing their differential exposure to early importation, we generate tier-specific evidence directly relevant to regional public health planning.

**Balcan et al. (2010) and the GLEAM framework**

The GLEAM framework (Balcan et al., 2010) represents a major advance in global epidemic simulation, integrating high-resolution population data with multiscale mobility networks to model large-scale spread. While such frameworks are designed for general-purpose epidemic forecasting, our study focuses on a more targeted empirical question. Specifically, we use a mobility-informed stochastic framework to (i) validate predicted first arrival patterns against observed outbreak data, (ii) characterize how transmission risk is structured across urban tiers within a national system, and (iii) compare spatial diffusion dynamics across pathogens with contrasting transmission profiles. This positions our work as an empirically grounded complement to existing large-scale simulation frameworks, with a focus on early-phase spatial inference rather than full epidemic reconstruction.

**Hunter et al. (2017)**

Hunter et al. (2017) emphasize the importance of clearly defining model scope and purpose in agent-based epidemiological modelling. In line with this perspective, we position our framework as a tool for early spatial inference, focusing on empirically grounded validation, sub-national risk stratification, and cross-pathogen comparison rather than general epidemic simulation.

Taken together, these studies establish the theoretical and computational foundations for mobility-coupled epidemic modelling. In the revised manuscript, we therefore clarify that our contribution lies not in proposing a new modelling paradigm, but in extending this framework along three complementary dimensions: (1) high-fidelity empirical validation using detailed outbreak data, (2) generation of sub-national, tier-stratified evidence to inform public health decision-making, and (3) direct comparison of how mobility shapes

spatial diffusion across pathogens with contrasting transmission characteristics. These elements together correspond to the three core contributions of the study: high-fidelity empirical validation, sub-national tier-stratified risk characterization, and cross-pathogen comparison of spatial diffusion.

We have revised the Discussion accordingly and also adjusted relevant wording in the Introduction and Methods to better position our modelling framework builds on established work on epidemic spread in mobility networks and spatial transmission systems.

**Discussion (Page 25, Lines 558-573)**

*Our modelling framework builds on established work on epidemic spread in metapopulation systems and mobility networks. Early theory showed that heterogeneous coupling and host movement can govern invasion across connected subpopulations, while subsequent work examined arrival-time distributions and multiscale mobility effects[1,2,42]. GLEAM further extended this tradition into a global epidemic-simulation platform [3]. We therefore do not consider the general use of mobility-coupled branching-process or metapopulation models to be novel. Instead, our contribution lies in adapting this modelling tradition to a specific empirical and inferential setting: early city-level spread within China, using daily intercity mobility data for 366 cities and validation against observed first arrival times from multiple outbreaks. In this context, our framework is designed not for general epidemic forecasting, but for reconstructing first arrival patterns, supporting origin inference, and comparing how pathogens with different transmission characteristics propagate across the urban hierarchy. We view this as a mobility-coupled stochastic diffusion model, with value in its empirical grounding, interpretability, and direct validation against observed spatial spread [43].*

4. Fourth, while I agree more work is needed, it's been established for over a decade that real-time mobility data are often essential for capturing epidemic dynamics. Early papers to discuss include Wesolowki et al. (2012) and (2015). A more recent position piece is Buckee et al. (2020). Kraemer et al. (2020) similarly used Baidu data to examine how SARS-CoV-2 spread from Wuhan and the effects of interventions. It would be important to

discuss how the results from Kraemer et al. (2020) on pre-Alpha SARS-CoV-2 qualitatively compare to the authors' results on Delta and Omicron. Lastly, Perofsky et al. (2024) recently published an analysis of many different respiratory pathogens in Seattle and explored the shifting role of mobility in explaining their dynamics. The key take-home from Perofsky et al. (2024) is that the type of mobility that matters varies a lot and changes over time. At a minimum, the authors should compare/contrast their results with Perofsky et al. (2024). Overall, the authors should ground their work more firmly in prior publications on real-time mobility data and epidemic spreading.

**Response**: We thank the reviewer for this important suggestion. We agree that the original Discussion did not sufficiently situate our study within the broader literature on the use of real-time or near-real-time mobility data to understand epidemic spread. In the revised manuscript, we have substantially expanded the Discussion to ground our work in prior studies better, using mobility data to infer spatial transmission, evaluate epidemic timing, and assess the effects of interventions, including Wesolowski et al. (2012, 2015), Buckee et al. (2020), Kraemer et al. (2020), Mercier et al. (2022), and Perofsky et al. (2024)

Wesolowski et al. (2012) used mobile phone-derived movement data and malaria prevalence data in Kenya to identify parasite sources and sinks and to quantify the contribution of human travel to malaria importation across regions. This study was foundational in demonstrating that human mobility data can reveal epidemiologically meaningful spatial connectivity beyond what would be inferred from geography alone. However, it focused on a vector-borne disease with a transmission ecology very different from that of directly transmitted respiratory pathogens, and it did not address stochastic intercity epidemic diffusion or compare pathogen-specific spread patterns within a national urban system. Our study builds on the general insight that mobility is epidemiologically informative. Still, it extends it by providing high-fidelity empirical validation of mobility-driven diffusion using observed first arrival times from real outbreaks.

Wesolowski et al. (2015) analyzed dengue epidemics in Pakistan by integrating epidemiological, climate, and mobile phone-based mobility data, and showed that mobility estimates improved the prediction of both the geographic spread and timing of

epidemics in established and emerging locations. This study is especially relevant because it linked mobility not only to where transmission occurred, but also to when epidemics emerged. Nevertheless, it focused on dengue, a vector-borne infection whose spread is strongly constrained by environmental suitability, rather than on stochastic intercity diffusion of respiratory pathogens through a national mobility network. In contrast, our study extends this line of work by examining how pathogen-specific epidemiological characteristics shape intercity diffusion within a shared mobility network, and by quantifying how early transmission risk is structured across urban tiers.

Buckee et al. (2020) was a perspective article arguing that aggregated mobility data collected by private companies could support pandemic response by providing near-real-time information on behavior change and intervention effects, while also highlighting the need for appropriate privacy and governance safeguards. We now cite this paper to clarify that we do not position our study as demonstrating the general importance of mobility data. Rather, our contribution is to embed routinely available intercity mobility data in a stochastic epidemic framework to study spatial diffusion risk, evaluate likely origins of spread using observed arrival times, and compare pathogen-specific dissemination patterns across city tiers.

Kraemer et al. (2020) is the most directly relevant prior study because it also used Baidu mobility data in China. That study showed that, during the initial spread of SARS-CoV-2 from Wuhan, mobility out of Wuhan strongly explained the spatial dissemination of infection across China, but that this association weakened substantially after major control measures were implemented. We now discuss this paper explicitly because it provides an important benchmark for interpreting our own findings. Qualitatively, our results are consistent with and extend those of Kraemer et al., showing that mobility structure is highly informative for the early geographic spread of SARS-CoV-2. In both studies, mobility patterns help explain where infection first appears and how rapidly it disseminates across cities in the early phase of the spread. At the same time, our findings also extend this comparison in important ways. Kraemer et al. focused on pre-Alpha SARS-CoV-2 during the first national wave in China and emphasized the weakening of the mobility-spread relationship after large-scale interventions. By contrast, our results indicate that while

mobility remains highly informative for early spread, the translation of mobility into spatial diffusion is pathogen-specific. By comparing Delta and Omicron within the same mobility system, we further show that differences in transmissibility and generation time lead to distinct diffusion patterns, providing a direct empirical comparison across pathogens.

Perofsky et al. (2024) is highly relevant for a different reason. Rather than analyzing a single pathogen with a single mobility measure, that study examined multiple respiratory viruses in Seattle. It showed that the mobility variables most associated with transmission differed across pathogens and over time. We agree with the reviewer that the key implication of this work is that there is no single universally dominant form of mobility for all respiratory viruses or all periods of transmission. Our findings provide complementary evidence at the intercity scale by showing that, even within a fixed mobility network, realized diffusion patterns differ across pathogens. This directly supports the conclusion that the epidemiological role of mobility is pathogen-specific, and extends it to the context of early spatial invasion across a national urban system.

We also added a discussion of Mercier et al. (2022), which showed that large mobility networks can be sparsified while preserving epidemic dynamics with high fidelity. Although that paper is methodological rather than focused on a particular outbreak, it reinforces the broader point that epidemic spread can be usefully analyzed on explicitly represented mobility networks. It helps position our work within a growing literature that treats mobility networks as central objects for epidemic inference and simulation. Our study differs in that it uses an empirical intercity mobility network to examine observed outbreak arrival patterns and pathogen-specific spatial diffusion in China.

Overall, real-time or near-real-time mobility data have been used for more than a decade to study the spatial spread of infectious diseases. Early studies demonstrated that mobile phone-derived movement data can identify epidemiologically meaningful connectivity beyond geographic distance alone and improve prediction of epidemic spread and timing. During the COVID-19 pandemic, such data were further recognized as valuable for monitoring behavioral change and assessing the effects of interventions. Our contribution is therefore not the use of mobility data itself, which is well established, but the way

mobility is operationalized within a stochastic diffusion framework to (1) enable empirical validation against observed first arrival patterns, (2) characterize sub-national transmission risk across urban hierarchies, and (3) provide a direct comparison of spatial diffusion across pathogens within a shared mobility system.

**Discussion (Page 25, Lines 575-600)**

*Real-time or near-real-time mobility data have been used for more than a decade to study the spatial spread of infectious diseases. Early studies showed that mobile phone-derived movement data could identify sources and sinks of malaria parasite importation and improve the prediction of the geographic spread and timing of dengue epidemics, demonstrating that human mobility can provide epidemiologically meaningful information beyond geographic distance alone [44,45]. During the COVID-19 pandemic, aggregated mobility data were further recognized as valuable for monitoring behavioral change and assessing intervention effects in near-real time [46]. Methodological work has also highlighted the importance of explicitly representing mobility networks for epidemic simulation[5]. In China, Kraemer et al. used Baidu mobility data to show that movement out of Wuhan strongly explained the early spatial dissemination of pre-Alpha SARS-CoV-2 across China, although this relationship weakened after major control measures were introduced; our findings are consistent with this broader conclusion that mobility structure is highly informative for early geographic spread, but extend it by showing that, under the later dynamic zero-COVID period, realised diffusion patterns differed substantially between Delta and Omicron despite being embedded in the same intercity mobility system[6]. Our interpretation is also consistent with the broader conclusion of Perofsky et al., who showed that the mobility measures most closely associated with respiratory virus transmission can vary markedly across pathogens and over time. However, that study focused on multiple local mobility indicators within Seattle, whereas we analyzed intercity flows across mainland China. Both studies suggest that the epidemiological role of mobility is not fixed but depends on pathogen characteristics, epidemic context, and spatial scale [7]. Taken together, these findings suggest that mobility is a crucial determinant of early spread, but that its form and magnitude of epidemiological relevance vary by pathogen and scale.*

## References


1. Colizza V, Vespignani A. Epidemic modeling in metapopulation systems with heterogeneous coupling pattern: theory and simulations. J Theor Biol. 2008;251(3):450-67.
2. Balcan D, Colizza V, Gonçalves B, Hu H, Ramasco JJ, Vespignani A. Multiscale mobility networks and the spatial spreading of infectious diseases. Proc Natl Acad Sci U S A. 2009;106(51):21484-9.
3. Balcan D, Gonçalves B, Hu H, Ramasco JJ, Colizza V, Vespignani A. Modeling the spatial spread of infectious diseases: The GLobal Epidemic and Mobility computational model. J Comput Sci. 2010;1(3):132-45.
4. Liu Y, Morgenstern C, Kelly J, Lowe R, Jit M. The impact of non-pharmaceutical interventions on SARS-CoV-2 transmission across 130 countries and territories. BMC Med. 2021;19(1):40.
5. Mercier A, Scarpino S, Moore C. Effective resistance against pandemics: Mobility network sparsification for high-fidelity epidemic simulations. PLoS Comput Biol. 2022;18(11):e1010650.
6. Kraemer MUG, Yang C-H, Gutierrez B, Wu C-H, Klein B, Pigott DM, et al. The effect of human mobility and control measures on the COVID-19 epidemic in China. Science. 2020;368(6490):493-7.
7. Perofsky AC, Hansen CL, Burstein R, Boyle S, Prentice R, Marshall C, et al. Impacts of human mobility on the citywide transmission dynamics of 18 respiratory viruses in pre- and post-COVID-19 pandemic years. Nat Commun. 2024;15(1):4164.
8. Gibbs H, Liu Y, Pearson CAB, Jarvis CI, Grundy C, Quilty BJ, et al. Changing travel patterns in China during the early stages of the COVID-19 pandemic. Nat Commun. 2020;11(1):5012.
9. Institution B. Metro Monitor 2025: Brookings Institution; 2025 [cited 2026 Apr 24]. Available from: https://www.brookings.edu/articles/metro-monitor-2025/.
10. (IBGE) BIoGaS. Regions of influence of cities (Regiões de Influência das Cidades): IBGE; 2018 [cited 2026 Apr 24]. Available from: https://www.ibge.gov.br/geociencias/cartas-e-mapas/redes-geograficas/15798-regioes-de-influencia-das-cidades.html.
11. Lee SA, Economou T, de Castro Catão R, Barcellos C, Lowe R. The impact of climate suitability, urbanisation, and connectivity on the expansion of dengue in 21st century Brazil. PLoS Negl Trop Dis. 2021;15(12):e0009773.
12. (BPS) SI. Human Development Index 2024 [cited 2026 Apr 24]. Available from: https://www.bps.go.id/en/statistics-table/2/NDEzIzI=/-new-method--human-development-index.html.
13. Department of Expenditure MoF, Government of India. Orders and circulars 2022 [cited 2026 Apr 24]. Available from: https://doe.gov.in/orders-circulars/32.
14. Vasudevan M, Natarajan N, Selvi SM, Ravikumar K, Rajendran AD, Bagavathi AB. Correlating the trends of COVID-19 spread and air quality during lockdowns in Tier-I and Tier-II cities of India-lessons learnt and futuristic strategies. Environ Sci Pollut Res Int. 2022;29(57):86126-55.
15. Sunilkumar A, Singh SK, Mondal AK, Ghosh P, Mondal S. Aggravation of CoVID-19 infections due to air pollutant concentrations in Indian cities. Spat Inf Res. 2023;31(5):525-36.
16. Kang Y, Gao S, Liang Y, Li M, Rao J, Kruse J. Multiscale dynamic human mobility flow dataset in the U.S. during the COVID-19 epidemic. Sci Data. 2020;7(1):390.

17. Gallotti R, Maniscalco D, Barthelemy M, De Domenico M. Distorted insights from human mobility data. Commun Phys. 2024;7(1):421.
18. Schindler DJ, Clarke J, Barahona M. Multiscale mobility patterns and the restriction of human movement. R Soc Open Sci. 2023;10(10):230405.
19. Meredith HR, Giles JR, Perez-Saez J, Mande T, Rinaldo A, Mutembo S, et al. Characterizing human mobility patterns in rural settings of sub-Saharan Africa. eLife. 2021;10:e68441.
20. Cai J, Deng X, Yang J, Sun K, Liu H, Chen Z, et al. Modeling transmission of SARS-CoV-2 Omicron in China. Nat Med. 2022;28(7):1468-75.
21. Guo Z, Zhao S, Lee SS, Mok CKP, Wong NS, Wang J, et al. Superspreading potential of COVID-19 outbreak seeded by Omicron variants of SARS-CoV-2 in Hong Kong. J Travel Med. 2022;29(6).
22. Mefsin YM, Chen D, Bond HS, Lin Y, Cheung JK, Wong JY, et al. Epidemiology of Infections with SARS-CoV-2 Omicron BA.2 Variant, Hong Kong, January-March 2022. Emerg Infect Dis. 2022;28(9):1856-8.
23. Zhang M, Xiao J, Deng A, Zhang Y, Zhuang Y, Hu T, et al. Transmission Dynamics of an Outbreak of the COVID-19 Delta Variant B.1.617.2 – Guangdong Province, China, May – June 2021. China CDC Wkly. 2021;3:584-6.
24. Ryu S, Kim D, Lim J-S, Ali ST, Cowling B. Serial Interval and Transmission Dynamics during SARS-CoV-2 Delta Variant Predominance, South Korea. Emerg Infect Dis. 2022;28(2):407.
25. Xu X, Wu Y, Kummer AG, Zhao Y, Hu Z, Wang Y, et al. Assessing changes in incubation period, serial interval, and generation time of SARS-CoV-2 variants of concern: a systematic review and meta-analysis. BMC Med. 2023;21(1):374.
26. Brockmann D, Helbing D. The hidden geometry of complex, network-driven contagion phenomena. Science. 2013;342(6164):1337-42.
27. Iannelli F, Koher A, Brockmann D, Hövel P, Sokolov IM. Effective distances for epidemics spreading on complex networks. Phys Rev E. 2017;95(1-1):012313.
28. Klamser PP, Zachariae A, Maier BF, Baranov O, Jongen C, Schlosser F, et al. Inferring country-specific import risk of diseases from the world air transportation network. PLoS Comput Biol. 2024;20(1):e1011775.
29. Chang L, Hou W, Zhao L, Zhang Y, Wang Y, Wu L, et al. The prevalence of antibodies to SARS-CoV-2 among blood donors in China. Nat Commun. 2021;12(1):1383.
30. Liu R, Zhang Y, Ma J, Wang H, Lan Y, Tang X. Epidemiological features of SARS-CoV-2 Omicron infection under new control strategy: a cross-sectional study of the outbreak since December 2022 in Sichuan, China. BMC Public Health. 2023;23(1):2463.
31. Zheng W, Yan X, Zhao Z, Yang J, Yu H. COVID-19 vaccination program in the mainland of China: a subnational descriptive analysis on target population size and current progress. Infect Dis Poverty. 2021;10(1):124.
32. Wu Q, Wang H, Cai J, Ai J, Li Y, Zhang H, et al. Vaccination effects on post-infection outcomes in the Omicron BA.2 outbreak in Shanghai. Emerg Microbes Infect. 2023;12(1):e2169197.
33. Liu Q, Tu X. Shanghai's Response to Fast Spreading of the COVID-19 Pandemic-Omicron (B.2). Asia Pac J Public Health. 2022;34(8):843-5.
34. Ge Y, Wu X, Zhang W, Wang X, Zhang D, Wang J, et al. Effects of public-health measures for zeroing out different SARS-CoV-2 variants. Nat Commun. 2023;14(1):5270.
35. Du Z, Shao Z, Zhang X, Chen R, Chen T, Bai Y, et al. Nowcasting and Forecasting Seasonal Influenza Epidemics - China, 2022-2023. China CDC Wkly. 2023;5(49):1100-6.

36. Jiang M, Jia M, Wang Q, Sun Y, Xu Y, Dai P, et al. Changes in the Epidemiological Features of Influenza After the COVID-19 Pandemic in China, the United States, and Australia: Updated Surveillance Data for Influenza Activity. Interact J Med Res. 2024;13:e47370.
37. Zhang L, Duan W, Ma C, Zhang J, Sun Y, Ma J, et al. An Intense Out-of-Season Rebound of Influenza Activity After the Relaxation of Coronavirus Disease 2019 Restrictions in Beijing, China. Open Forum Infect Dis. 2024;11(4):ofae163.
38. Zhang R, Peng Z, Meng Y, Song H, Wang S, Bi P, et al. Temperature and influenza transmission: Risk assessment and attributable burden estimation among 30 cities in China. Environ Res. 2022;215:114343.
39. Yan Z-L, Liu W-H, Long Y-X, Ming B-W, Yang Z, Qin P-Z, et al. Effects of meteorological factors on influenza transmissibility by virus type/subtype. BMC Public Health. 2024;24(1):494.
40. Luo H, Cui Y, Yu W, Li G, Zhao Q, Geng M, et al. The impact of urbanization in China on influenza incidence across neighboring cities. J Infect. 2025;90(1):106370.
41. Lei H, Zhang N, Niu B, Wang X, Xiao S, Du X, et al. Effect of Rapid Urbanization in Mainland China on the Seasonal Influenza Epidemic: Spatiotemporal Analysis of Surveillance Data From 2010 to 2017. JMIR Public Health Surveill. 2023;9.
42. Jamieson-Lane A, Blasius B. Calculation of epidemic arrival time distributions using branching processes. Phys Rev E. 2020;102(4):042301.
43. Hunter E, Mac Namee B, Kelleher JD. A taxonomy for agent-based models in human infectious disease epidemiology. J Artif Soc Soc Simul. 2017;20(3).
44. Wesolowski A, Eagle N, Tatem AJ, Smith DL, Noor AM, Snow RW, et al. Quantifying the Impact of Human Mobility on Malaria. Science. 2012;338(6104):267-70.
45. Wesolowski A, Qureshi T, Boni MF, Sundsøy PR, Johansson MA, Rasheed SB, et al. Impact of human mobility on the emergence of dengue epidemics in Pakistan. Proc Natl Acad Sci U S A. 2015;112(38):11887-92.
46. Sills J, Buckee CO, Balsari S, Chan J, Crosas M, Dominici F, et al. Aggregated mobility data could help fight COVID-19. Science. 2020;368(6487):145-6.
