# Supplementary material for: Assessing spatial transmission risk of respiratory infectious diseases across cities of different socioeconomic tiers in China: A modelling study
Source: PLoS Med. 2026 Jul 20;23(7):e1005172. doi: 10.1371/journal.pmed.1005172 (PMC13384292; doi:10.1371/journal.pmed.1005172)
Supplement: Attachment — Submitted filename: Response to Editors R3_resubmission.docx [file pmed.1005172.s003.docx]

**Manuscript Number: PMEDICINE-D-25-04489**

**Assessing spatial transmission risk of respiratory infectious diseases across cities of different socioeconomic tiers in China: a modelling study**

**Wenjie Li, Wei Yang, Yang Liu, Ye Yao**

**PLOS Medicine**

**Response to Editors**

We thank the editors for the detailed guidance. We have carefully addressed each remaining editorial and reporting request in the revised manuscript, the supplementary material, the figure/table legends, and the submission statements. The corresponding changes are indicated in the marked-up manuscript. Our point-by-point responses are provided below.

1. To improve clarity please change the title to: Assessing spatial transmission risk of respiratory infectious diseases across cities of different socioeconomic tiers in China: a modelling study

**Response**: We have revised the title exactly as requested.

2. Please revise the last sentence in the abstract to tone it down as follows: Because the framework relies on routinely available mobility data and minimal pathogen-specific inputs, it may provide a scalable approach for epidemic preparedness, including potentially future emerging respiratory threats (DiseaseX).

**Response**: We have revised the final sentence of the Abstract as requested: “Because the framework relies on routinely available mobility data and minimal pathogen-specific inputs, it may provide a scalable approach for epidemic preparedness, including potentially future emerging respiratory threats (DiseaseX).”

3. It appears that one or more study authors may be affiliated with one or more of the agencies that funded the study. Thus, the statement “The funders had no role in study design, data collection and analysis, decision to publish, or preparation of the manuscript” would not apply. If applicable, please revise the Financial Disclosure accordingly, as in "[Author name] is [author's role] at [funding agency]. The funders had no other role in study design…..”

**Response**: We have carefully reviewed the authors’ affiliations and the funding sources. None of the authors is employed by, holds an official position at, or has any funding relationship with the agencies that supported this study. In particular, although Dr. Yang Liu is affiliated with PATH, this organization did not provide funding for this work and had no role in study design, data collection and analysis, decision to publish, or preparation of the manuscript. We have therefore retained the Financial Disclosure statement indicating that the funders had no role in the study.

**COMPETING INTERESTS STATEMENT**

1. All authors must declare their relevant competing interests per the PLOS policy, which can be seen here: https://journals.plos.org/plosmedicine/s/competing-interests For authors with ties to industry, please indicate whether any of the interests has a financial stake in the results of the current study.

**Response**: We confirm that all authors have declared their relevant competing interests in accordance with the PLOS policy, and that no author has any financial or non-financial competing interest related to this study. None of the authors has industry ties or a financial stake in the results of the current study.

Regarding the per-author confirmation in Editorial Manager, all co-authors except Dr. Yang Liu have completed the confirmation in the system. Dr. Liu has been unable to complete the confirmation because the confirmation link in the system is not working for her, even after the letter was resent. Dr. Liu has already contacted the editorial office by email to report this technical issue. We would be grateful for the editorial office’s assistance in resolving this matter, and we confirm, on her behalf, that Dr. Liu also declares no competing interests.

**DATA AVAILABILITY**

1. For studies in which a novel model is central to the manuscript's findings, as is the case here, authors are responsible for providing the source code needed to replicate the study's findings in a repository (such as GitHub, SourceForge or Bitbucket) or a cloud computing service (such as Code Ocean). Please include a citable digital object identifier (DOI) or other persistent identifier of the code (such as through linkage with Zenodo). Protection of authors’ intellectual property will not be cause for exception. Please explain in the manuscript’s Data Availability Statement how readers can access the shared code.

**Response**: We have revised the Data sharing statement to clarify how readers can access the source code. **(Page 32 Lines 757-760)**

*All datasets used in this study, including Baidu Migration data, first confirmed case data, and processed analytical inputs, are publicly available in the Zenodo repository (*[*https://doi.org/10.5281/zenodo.17852176*](https://doi.org/10.5281/zenodo.17852176)*). The analysis code is publicly available on GitHub (*[*https://github.com/OOLWJOO/Diffusion_Based_on_Baidu_Migration*](https://github.com/OOLWJOO/Diffusion_Based_on_Baidu_Migration)*).*

2. Please include the statement on code availability in the data availability statement. Please see guidance here:https://journals.plos.org/plosmedicine/s/code-sharing-guidance#loc-data-availability-statement-examples
   **Response**: The Data sharing statement now explicitly includes the availability of the analysis code and provides the Zenodo DOI. The analysis code is publicly available on GitHub (https://github.com/OOLWJOO/Diffusion_Based_on_Baidu_Migration), and this code link is also provided within our Zenodo repository (<https://doi.org/10.5281/zenodo.17852176>).

**FIGURES**

1. Please provide titles and legends for all figures and tables (including those in Supporting Information files). Please define all acronyms used in each figure or table in its corresponding legend.

**Response**: All figures and tables in the main manuscript and the Supporting Information files are provided with titles and legends. We have also reviewed the figures and tables to ensure that acronyms are defined in their corresponding legends. In particular, we revised the legend of Fig 1 to spell out the abbreviations used in the figure: probability mass function (pmf) and probability density function (pdf).

2. When a p value is given, please specify the statistical test used to determine it in the legend.

**Response**: We have specified the statistical test in the relevant figure legends. In the legends of Fig 2 and Fig 3, we now state that the correlation coefficients (r), p values, and 95% confidence intervals were derived from the Pearson correlation test.

3. Please consider avoiding the use of red and green in order to make your figure more accessible

**Response**: We thank the editor for this helpful suggestion regarding accessibility. We have reviewed our figures and confirm that none of them rely on a red-and-green colour combination, which is the principal source of difficulty for readers with red-green colour vision deficiency. In Fig 1, colour is used as a single-hue sequential scheme, in which different shades (intensities) of red encode the speed of first arrival time, rather than contrasting red against green. Because this information is conveyed through differences in lightness and saturation along a single hue rather than through red-versus-green hue discrimination, the gradient remains perceptible to individuals with red-green colour vision deficiency, who can still distinguish the underlying luminance differences. For this reason, we have retained the current colour scheme, as we believe it is both accessible and effective for conveying the intended information.

4. Please confirm that the appropriate usage rights apply to the use of this map. Please see our guidelines for map images: <https://journals.plos.org/plosmedicine/s/figures#loc-maps>

**Response**: We confirm that the appropriate usage rights apply to the maps used in this study. The basemap shapefiles were obtained from the Chinese Resource and Environmental Science Data Platform (http://www.resdc.cn/, DOI: 10.12078/2023010102), and this source is cited in the corresponding figure legends. During the previous revision (R2), the editorial office had already confirmed with us that the usage rights for this map source are acceptable and that maps from this same source have been used in previously published PLOS articles.

5. Please provide the unadjusted comparisons as well as the adjusted comparisons in all relevant Tables

**Response**: This request is not directly applicable to the main modelling results because the manuscript does not present covariate-adjusted regression estimates as primary table results. To improve transparency, we added/retained cross-tier comparisons in absolute and relative terms in the Supplementary Material and provided permutation-based comparisons alongside Kruskal-Wallis tests. Where model-based analyses are reported, the variables included in the model are explicitly described.

6. Please specify the variables controlled for in all relevant Tables

**Response**: For the exploratory mixed-effects arrival-time analysis in the supplementary material, we have specified the variables included in the model: population flows from origin to destination, an indicator for zero-flow connections, geodesic distance, prefecture tier, destination-city resident population size, and a random intercept for destination city. No other tables report adjusted regression estimates.

**MODELING STUDIES**

1. Please provide a diagram that shows the model structure, including how the disease natural history is represented, the process and determinants of disease acquisition, and how the putative intervention could affect the system.

**Response**: We have provided a model structure diagram in Fig 1. The figure illustrates the agent-based branching process, including the disease natural history via the serial interval distribution, secondary infection generation via the reproduction number distribution, travel decisions based on mobility probabilities, and the seeding of infections across cities by infected individuals. We also included pseudocode in the Methods to clarify the simulation process.

2. Please provide a complete list of model parameters, including clear and precise descriptions of the meaning of each parameter, together with the values or ranges for each, with justification or the primary source cited, and important caveats about the use of these values noted.

**Response**: We provide model parameters in Table 1 of the main manuscript, including reproduction number, overdispersion, serial interval distribution, values, and sources for SARS-CoV-2 Omicron and Influenza A. Additional parameter ranges used in uncertainty analyses are provided in Supplementary Table D, and migration-related assumptions are described in the Supplementary Methods.

3. Please provide a clear statement about how the model was fitted to the data including where relevant goodness-of-fit measure, the numerical algorithm used, which parameter varied, constraints imposed on parameter values, and starting conditions.

**Response**: We have clarified in the Methods (and Supplementary Methods) how the model relates to the data, addressing each element raised by the editor. Our framework is a forward stochastic simulation model rather than a model calibrated by statistical optimisation: epidemiological parameters were specified a priori from the literature, and mobility parameters were derived directly from Baidu Migration data, and we then evaluated how well the simulated output reproduced independent observed data.

Specifically:

- Numerical algorithm: The model is a spatial agent-based branching process simulated stochastically. The number of secondary infections per case is drawn from a negative binomial offspring distribution; serial intervals are drawn from pathogen-specific distributions (Gamma for SARS-CoV-2, Weibull for influenza A); and intercity travel is simulated using a sigmoid migration-probability function, with destinations assigned based on observed Baidu outflow proportions. Each outbreak-origin scenario was simulated 10,000 times to capture stochastic variability. The full procedure is provided as pseudocode in the Methods.

- Starting conditions: Each simulation was initialised by seeding a specified number of infections in the origin city on day 1 (100 seed infections for the risk-assessment scenarios), with the simulation run over a fixed time window (e.g., 7, 14, and 21 days).

- Parameters specified vs varied: For the primary first-arrival-time validation, infections propagated through mobility alone, with pathogen-specific reproduction number and serial interval omitted, so that arrival ordering reflected the mobility network rather than tuned epidemiological parameters. For the risk-assessment scenarios, pathogen-specific parameters were fixed at literature-based values (Table 1). In the sensitivity and joint uncertainty analyses, we systematically varied the reproduction number, overdispersion, serial interval shape and scale, the sigmoid scaling parameter, and the baseline Shanghai migration probability (Supplementary Table D).

- Constraints on parameter values: The baseline daily migration probability for Shanghai was anchored at approximately 1%, back-calculated from external commuting-flow data combined with the Baidu migration index, and constrained to a plausible range of 0.5%–1.5% in sensitivity analyses. Other parameters were constrained to literature-based plausible ranges in the uncertainty analyses (Supplementary Table D).

- Goodness-of-fit measures: Model performance was assessed by comparing simulated and observed city-level first arrival times using the Pearson correlation coefficient (on log-transformed values), mean absolute error (MAE), and root mean square error (RMSE). We additionally validated the model against the cumulative number of affected cities (observed 65 vs. simulated mean 60.4 at day 30) and the day-by-day temporal growth trajectory of spatial spread, which showed no statistically significant difference between observed and simulated patterns (two-sided p = 0.72; Supplementary Material).

4. For uncertainty analyses, please state the sources of uncertainties quantified and not quantified this can include parameter, data, and model structure.

**Response**: We have clarified, both in the main text and the Supplementary Material, which sources of uncertainty were formally quantified and where they are described, and how the remaining sources of uncertainty were explored.

Sources of uncertainty quantified, and where they are described:

- Stochastic (Monte Carlo) variability was quantified by running 10,000 simulation replicates per scenario (Supplementary Material S1.6).

- Parameter uncertainty was quantified through one-way sensitivity analyses varying the initial number of infections, the baseline Shanghai migration probability (0.5%–1.5%), the sigmoid migration-function scaling parameter, and the importation threshold defining a city as at risk (Supplementary Material S1.13, S1.15, S2.6, S2.7), and through a joint probabilistic uncertainty analysis that simultaneously varied six key parameters using Latin hypercube sampling (Supplementary Material S1.14, S2.9; Supplementary Table D).

- Uncertainty in origin identification was quantified using permutation tests, bootstrap 95% confidence intervals, a max-statistic permutation test, and a city-level bootstrap (Supplementary Material S1.11, S2.1).

- Uncertainty in the cross-tier statistical comparisons was quantified using permutation-based inference in addition to the Kruskal–Wallis tests (Supplementary Material S1.16, S2.8).

Sources of uncertainty not formally quantified, and the additional analyses we performed to explore them: These sources were previously raised by the reviewers, and although they were not propagated as formal uncertainty distributions, we added dedicated supplementary analyses to examine their potential influence.

- Data uncertainty relating to the mobility regime: Because the main analysis used mobility data from China’s “dynamic zero-COVID” period, we added a robustness analysis using post–zero-COVID mobility data from 2023, together with a structural comparison of intercity mobility matrices across four periods (Supplementary Material S1.12, S2.2).

- Parameter/contextual uncertainty not represented in the main arrival-time model (city-specific prior infection, vaccination coverage, time-varying intervention intensity, and climatic forcing): we added an exploratory post-arrival local transmission analysis based on a city-level SIR model, illustrating how these factors could influence post-introduction epidemic trajectories (Supplementary Material S1.17, S2.10; Figs AA–BB).

5. Please provide sensitivity analyses to identify which parameter values are most important in the model. Uncertainty estimates seek to derive a range of credible results on the basis of an exploration of the range of reasonable parameter values. The choice of method should be presented and justified.

**Response**: Our manuscript already includes a comprehensive set of sensitivity and uncertainty analyses, which we describe more clearly here in relation to the editor’s request.

Methods used and their justification:

- We performed one-way sensitivity analyses, in which individual parameters were varied across plausible ranges while holding others fixed, to isolate the effect of each parameter on model outputs. Parameters examined included the initial number of infections, the baseline Shanghai migration probability (0.5%–1.5%), the sigmoid migration-function scaling parameter, and the importation threshold used to define a city as at risk (Supplementary Material S1.13, S1.15, S2.6, S2.7). This one-at-a-time approach was chosen because it provides a transparent and directly interpretable assessment of the marginal influence of each parameter.

- We complemented this with a joint probabilistic uncertainty analysis using Latin hypercube sampling, in which six key parameters—the mean reproduction number, overdispersion, serial interval shape and scale, the sigmoid scaling parameter, and the baseline Shanghai migration probability—were varied simultaneously across their plausible ranges (Supplementary Material S1.14, S2.9; Supplementary Table D). Latin hypercube sampling was chosen because it efficiently covers a high-dimensional parameter space with far fewer samples than a full factorial grid, and because it allows the joint effects of simultaneous parameter variation to propagate through the full simulation, yielding a range of credible results rather than a single point estimate.

Critically, across all one-way and joint analyses, these parameters affected the magnitude of the estimates but not the qualitative conclusions: the hierarchical ordering of transmission risk across prefecture tiers (with super-tier and higher-tier cities consistently at greatest early risk) remained stable. This indicates that the central conclusions are robust across the explored range of credible parameter values.

6. Please discuss the scientific rationale for this choice of model structure and identify points where this choice could influence conclusions drawn. Please also describe the strength of the scientific basis underlying the key model assumptions.

**Response**: We have addressed all three elements of this request.

(i) Scientific rationale for the model structure: The Discussion explains that our framework builds on established metapopulation and mobility-network modelling theory and clarifies why a mobility-coupled stochastic, agent-based branching-process structure was chosen for our specific aim of reconstructing early city-level first-arrival patterns, supporting origin inference, and comparing pathogens with contrasting transmissibility, rather than for general epidemic forecasting. The Methods and Fig 1 further describe the model structure, including secondary infection generation, serial interval sampling, intercity travel, and how these components jointly determine first arrival times. Because this study did not evaluate a specific intervention, no putative intervention pathway was incorporated into the model diagram.

(ii) Points where this choice could influence conclusions: We explicitly note that focusing on first-arrival timing and omitting city-specific prior infection, vaccination, time-varying intervention intensity, and climatic forcing could alter the relative ranking of cities and pathogens if the framework were extended to post-introduction burden. We have illustrated this issue with an exploratory post-arrival local transmission analysis in the Supplementary Materials S1.17, S2.10.

(iii) Strength of the scientific basis underlying key model assumptions: We have added a dedicated passage to the Discussion that distinguishes assumptions with a strong empirical basis, including mobility as a driver of early spread, the negative-binomial branching-process representation, and the serial interval as the temporal scale, from assumptions resting on a more approximate basis, including the sigmoid travel-probability function anchored to Shanghai and the prefecture-tier classification. We also note that the assumptions with a weaker basis may affect the magnitude of estimates but not the qualitative cross-tier conclusions, consistent with our sensitivity and uncertainty analyses. **(Page 27 Lines 633-651)**

*The key assumptions underlying our model are supported by different levels of empirical evidence. The assumption that intercity human mobility is a primary driver of early city-level spatial spread has a strong empirical basis, supported by extensive prior work using mobile-phone and platform-based mobility data, as well as by our own validation against observed first-arrival times across multiple outbreaks. Representing transmission as a branching process with a negative binomial offspring distribution is also well supported, as it is a standard, empirically grounded approach for capturing individual-level heterogeneity and superspreading. The use of the serial interval as the temporal scale for comparing arrival times is justified by our validation target, which is based on the timing of detected cases rather than unobserved infection events. By contrast, two assumptions rest on a more approximate basis and are therefore treated as sensitivity targets rather than fixed assumptions: the sigmoid travel-probability function anchored to an estimated baseline daily migration probability for Shanghai, which is an empirically informed approximation rather than a directly observed quantity; and the prefecture-tier classification, which captures commercial and functional urban hierarchy rather than epidemiological vulnerability per se. Across these assumptions, our sensitivity and uncertainty analyses suggest that assumptions with weaker empirical support may influence the magnitude of estimates but do not materially alter the qualitative cross-tier conclusions.*
