# Supplementary material for: Assessing spatial transmission risk of respiratory infectious diseases across cities of different socioeconomic tiers in China: A modelling study
Source: PLoS Med. 2026 Jul 20;23(7):e1005172. doi: 10.1371/journal.pmed.1005172 (PMC13384292; doi:10.1371/journal.pmed.1005172)
Supplement: Peer review history

# Editor report: Decision Letter 0

**DOI:** 10.1371/journal.pmed.1005172.r001

**Authors:** Heather Van Epps

24 Dec 2025

Dear Dr Yao,

Thank you for submitting your manuscript entitled "Assessing spatial transmission risk of respiratory infectious diseases across urban tiers in China: a modelling study" for consideration by PLOS Medicine.

Your manuscript has now been evaluated by the PLOS Medicine editorial staff, and I am writing to let you know that we would like to send your submission out for external peer review.

However, before we can send your manuscript to reviewers, we need you to complete your submission by providing the metadata that is required for full assessment. To this end, please login to Editorial Manager where you will find the paper in the ‘submissions Needing Revisions’ folder on your homepage. Please click ‘Revise Submission’ from the Action Links and complete all additional questions in the submission questionnaire.

For clinical studies, please upload a copy of your trial study protocol as a supporting information file. The study protocol should be the version submitted for approval to the institutional review board or ethics committee, should include any amendments to the study protocol, as well as the date of their approval by the institutional review or ethics committee. Please also detail any deviations from the study protocol in the Methods section of your manuscript. The editors will consider the protocol and study conduct prior to a final decision for external review.

Please re-submit your manuscript within two working days, i.e. by Dec 26 2025 11:59PM.

Login to Editorial Manager here: https://www.editorialmanager.com/pmedicine

Once your full submission is complete, your paper will undergo a series of checks in preparation for peer review. Once your manuscript has passed all checks it will be sent out for review.

Feel free to email us at plosmedicine@plos.org if you have any queries relating to your submission.

Kind regards,

Heather Van Epps, PhD

Consulting Editor

PLOS Medicine

# Peer review: Decision Letter 1

**DOI:** 10.1371/journal.pmed.1005172.r002

**Authors:** Andreia Cunha

17 Mar 2026

Dear Dr Yao,

Sincere apologies for the delay in getting back to you with a decision which was due to challenges in securing the necessary advice. Many thanks for submitting your manuscript "Assessing spatial transmission risk of respiratory infectious diseases across urban tiers in China: a modelling study" (PMEDICINE-D-25-04489R1) to PLOS Medicine. The paper has been reviewed by two subject experts and a statistician; their comments are included below and can also be accessed here: [LINK]. Please disregard the report of Reviewer 1.

As you will see, the reviewers have raised some serious concerns that we must ask you to address in full. After discussing the paper with the editorial team and an academic editor with relevant expertise, I’m pleased to invite you to revise the paper in response to the reviewers’ comments. We plan to send the revised paper to some or all of the original reviewers, and we cannot provide any guarantees at this stage regarding publication. We will be reluctant to send the manuscript back to the original Reviewers if the advance over previous work is not made clear as well as the potential for generalizability. Please also revise the text to ensure it is accessible to a broad audience, including defining specialist concepts on first use, and making the discussion of how these results can inform policy decisions or action more accessible.

In addition to these revisions, you may need to complete some formatting changes, which you will receive in a follow up email. A member of our team will be in touch with a set of requests shortly. If you do not receive a separate email within a few days, please assume that checks have been completed, and no additional changes are required.

When you upload your revision, please include a point-by-point response that addresses all of the reviewer and editorial points, indicating the changes made in the manuscript and either an excerpt of the revised text or the location (eg: page and line number) where each change can be found. Please also be sure to check the general editorial comments at the end of this letter and include these in your point-by-point response. When you resubmit your paper, please include a clean version of the paper as the main article file and a version with changes tracked as a marked-up manuscript. It may also be helpful to check the guidelines for revised papers at http://journals.plos.org/plosmedicine/s/revising-your-manuscript for any that apply to your paper.

We ask that you submit your revision by Apr 07 2026 11:59PM. However, if this deadline is not feasible, please contact me by email, and we can discuss a suitable alternative.

Don’t hesitate to contact me directly with any questions (acunha@plos.org).

Best regards,

Andreia

Andreia Cunha, PhD

Senior editor

PLOS Medicine

acunha@plos.org

-----------------------------------------------------------

Comments from the reviewers:

Reviewer #2: This manuscript presents a mobility-informed, agent-based branching process framework to characterise the spatial transmission risk of respiratory infectious diseases across urban tiers in China. By integrating large-scale Baidu Migration data with a city-level branching process model, the authors examine tier-specific diffusion patterns and validate their approach using three well-documented COVID-19 outbreaks. The comparison between SARS-CoV-2 (Omicron) and Influenza A provides a contrast between highly transmissible and more moderately transmissible pathogens that is potentially relevant for public-health preparedness.

Overall, the study addresses an important question in spatial epidemiology. However, similar mobility-based modelling approaches have been extensively explored in the literature, and the conceptual novelty of the current work is limited. Moreover, it is unclear whether the primary outcome "first-arrival time" has sufficient public-health relevance to justify the complexity of the proposed framework or to serve as a robust validation target. In practice, outbreak origins are typically identified through epidemiological and outbreak investigation efforts, raising the question of what additional actionable insight this model provides beyond existing approaches.

While the use of large-scale mobile phone-derived mobility data is a strength, I remain unconvinced that the framework, as currently presented, offers clear or substantial added value for public-health decision-making. Given these concerns, I am hesitant to recommend publication in PLOS Medicine. My specific comments are outlined below.

Major Comments

1. Dependence on mobility patterns during China’s "dynamic zero-COVID" period

The mobility data used in this study are drawn from a period characterised by substantial but structured mobility restrictions under China’s "dynamic zero-COVID" policy. Although this context is acknowledged, the implications for generalisability are not sufficiently discussed. How might the observed tier-specific diffusion patterns differ under post-2022 mobility regimes, or in settings without comparable intervention intensity? The authors should more clearly frame China as a specific case study or proof-of-concept and discuss the extent to which the findings and the proposed framework can be transferred to other epidemiological and policy contexts.

2. Model validation beyond first-arrival times

Model validation focuses primarily on correlations between predicted and observed first-arrival times. While this metric may be relevant for early spatial diffusion, arrival-time ordering alone does not fully capture outbreak dynamics or public-health impact. The authors should justify why first-arrival time was prioritised over alternative or complementary validation targets, such as the cumulative number of affected cities, the temporal growth of spatial spread, or measures more directly linked to intervention decision-making.

Minor Comments

*Lines 93-94: The statement "In countries with substantial socioeconomic heterogeneity and hierarchical urban systems" is vague. Please specify which countries or settings are being referred to, beyond China.

*Line 112: Please provide a clearer explanation of the migration index. Are comparable mobility indices available in other countries, and if so, could this framework be realistically implemented elsewhere?

*Line 124: Please spell out the abbreviation PHSM at first use.

*Line 136: The serial interval is used in the model—please clarify whether this is being treated as a proxy for the generation time, and discuss any implications.

*Line 205: Please explain why Spearman correlation was used instead of Pearson correlation in this analysis.

Reviewer #3: Major Comments

1) The agent-based branching process model is central to the analysis, yet several key implementation details required for reproducibility remain insufficiently specified. In particular, the manuscript does not clearly report the total number of stochastic simulations per scenario, whether results are based on means, medians, or specific quantiles of the simulated distributions, nor how Monte Carlo uncertainty was propagated into the reported outcomes. Given the stochastic nature of the model, this information is essential.

2) The decision to omit pathogen-specific parameters (i.e., reproduction number and serial interval) when simulating first-arrival times for validation purposes requires stronger justification. While the intention to isolate mobility-driven diffusion is understandable, this assumption implicitly treats all infections as temporally equivalent across pathogens and outbreaks. The potential bias introduced by ignoring heterogeneity in incubation and serial intervals—especially when comparing Omicron and Delta outbreaks—should be more explicitly discussed and, ideally, assessed via sensitivity analysis.

3) The validation strategy relies almost exclusively on correlation between predicted and observed first-arrival times. Correlation, however, measures rank or linear association rather than calibration or absolute predictive accuracy. High correlation can coexist with substantial systematic bias. The authors should justify why correlation alone is an adequate validation metric, or complement it with additional measures (e.g., mean absolute error, root mean squared error, or calibration plots).

4) The use of Pearson correlation for origin identification assumes approximate linearity and normality of log-transformed arrival times, yet the empirical distributions of arrival times across cities appear highly skewed and heterogeneous. Although a log-log correlation is mentioned in the Supplementary Methods, this transformation is not consistently described or clearly reflected in the main Methods section. Greater clarity is needed regarding which correlation metric (raw vs log-transformed) was used in each analysis and why.

5) The approach to origin identification involves testing all 366 cities as potential sources and selecting the city with the maximum correlation. This raises concerns about multiple testing and overfitting, particularly given the relatively small number of affected cities in some outbreaks (e.g., n = 13 for Nanjing). The manuscript does not address how statistical uncertainty in correlation estimates was handled, nor whether differences between top-ranked candidate cities were meaningfully distinguishable.

6) The specification of the migration probability function relies on anchoring Shanghai’s daily travel probability at 1%, which is treated as a fixed reference value. Although sensitivity analyses are presented, the empirical basis for selecting this baseline remains unclear. It is not evident whether this value reflects observed per-capita travel behaviour, nor how sensitive cross-tier comparisons are to this assumption in absolute (not only relative) terms.

7) The definition of the epidemic risk metric (R*) as the total population of cities that receive at least one infected individual is a coarse, binary measure that ignores infection intensity, timing, and secondary transmission within destination cities. While the authors note this simplification, the implications for interpreting "risk" as a public health metric should be more carefully articulated, especially when comparing pathogens with very different transmission dynamics.

8) Statistical inference for tier-level comparisons is insufficiently described. The manuscript reports highly significant p-values (e.g., p < 0.001) when comparing epidemic risk across prefecture tiers, but does not specify the underlying statistical test, the unit of analysis, or whether assumptions such as independence were met. Given that cities are connected through the mobility network, treating them as independent observations may not be valid.

9) The treatment of uncertainty in model inputs (R, serial interval parameters, migration probabilities) is largely limited to one-way sensitivity analyses. A more coherent probabilistic uncertainty analysis—jointly sampling key parameters—would be more consistent with best practice in stochastic epidemic modelling and would strengthen confidence in the robustness of the conclusions.

10) Although extensive supplementary material is provided, several methodological elements critical to interpretation (e.g., the log-log correlation rationale, the construction of mobility transition matrices, and details of sensitivity analyses) are only described in the supplement. Given their importance, these should be more explicitly summarised in the main Methods section to ensure transparency for readers who may not consult the supplementary files in detail.

Minor Comments

11) The terminology used for reproduction numbers alternates between "effective reproduction number" (R) and "basic reproduction number" (R₀ / R*) across sections and pathogens. A consistent notation and clearer explanation of why different measures are used for Omicron and Influenza A would improve clarity.

12) The serial interval distributions are specified with point estimates for parameters, but it is unclear whether uncertainty in these parameters was incorporated into the simulations or treated as fixed. Clarifying this would improve transparency.

13) The Methods section would benefit from a concise schematic or algorithmic description (e.g., stepwise outline or pseudocode) of the simulation process, particularly for readers less familiar with agent-based branching process models.

14) The assumption of 100 initial cases for risk simulations is somewhat arbitrary. Although alternative values are explored in sensitivity analyses, a brief justification for this choice in the main text would be helpful.

15) The description of the prefecture-tier classification relies on an externally defined commercial ranking system. While this is reasonable, the potential mismatch between commercial attractiveness and epidemiological vulnerability could be acknowledged more explicitly as a limitation of the stratification approach.

16) The handling of cities that do not experience any introductions during the simulation period is not clearly described. It would be useful to clarify whether such cities are censored, assigned infinite arrival times, or excluded from certain analyses.

17) Minor editorial clarification is needed to distinguish clearly between validation analyses using observed COVID-19 outbreaks and hypothetical simulations for Influenza A, as the transition between these sections may be confusing for some readers.

Reviewer #4: In this paper, the authors develop an agent-based branching process model that incorporates mobility to study the movement of respiratory pathogens through hierarchical urban/rural areas. They apply their model to SARS-CoV-2 Delta and Omicron variant outbreaks in China, and also influenza outbreaks in China. As the authors highlight, while the use of mobility data to ground dynamic transmission models has advanced, we have few comparative-level analyses looking across different pathogen types and/or variants with meaningfully different epidemiological parameters. The authors identify differences between the two SARS-CoV-2 variants and influenza, and account for them mechanistically by examining how these pathogens spread. The comparisons provided are a real strength, differentiating the work from much of what has been done previously. Additionally, and just as importantly, the authors also move beyond simple single-parameter descriptions of outbreaks, i.e., R0, and include overdispersion in their analysis. I do have four substantive concerns.

First, the approach does not seem to account for the existing immunological landscape nor potential seasonal drivers of influenza transmission related to climate. Regarding prior immunity, despite China’s largely successful COVID-zero strategy, sporadic outbreaks still occurred. The authors should discuss the prior infection landscape in the cities under investigation. Relatedly, while I know there were concerns about whether the vaccine would protect against Omicron infection, there was no discussion of how to account for pre-existing vaccination. Regarding COVID-zero, my understanding is that the policy was largely abandoned when Omicron began spreading. How should we compare/contrast the Delta outbreaks (when intensive interventions were used to control transmission, including mobility restrictions) vs. Omicron? Regarding influenza, the landscape of immunity from COVID was quite complicated in many countries, including the US. Were there similarly atypical patterns of influenza in China that would have impacted the immunological landscape? I know aspects like immunity are likely why the authors focused on arrival time, but the authors should discuss what we know about how mobility, interventions, immunity, climate, urban/rural, etc., might interact to drastically change the results if we considered final attack rate, peak height, epidemic intensity, or other measures of how the outbreak would unfold.

Second, if it weren’t for the overdispersion, a criticism would be that all the results boil down to pathogens with higher R0 and lower generation time spreading faster. I think what the authors are doing is much more interesting than that, but focusing exclusively on arrival time makes it a bit hard to determine what would happen if we looked at some of the measures I mentioned in the previous section. Perhaps most critically, Mercier et al. (2022) showed how ~90% of mobility could be ignored in the US and simulated SIR epidemics could be entirely recreated, not just the arrival times (which they also considered). One possible middle ground (because I agree that moving beyond arrival time would raise more challenges than it would address) would be to examine the distribution of estimated arrival times and fit a hierarchical or mixed-effects model that accounts for uncertainty in arrival times. Mercier et al. (2022) show that distributions of arrival times can be more diagnostic.

Third, the use of branching process models to study epidemic transmission across populations with mobility is well established. At least since Colizza and Vespignani (2008), scientists have studied how epidemics unfold in metapopulations using approaches closely related to branching process models. More recently, Jamieson-Lane and Blasius (2020) examined branching processes in the context of pathogen arrival times. The most relevant application would be Balcan et al. (2009) who discuss an agent-based branching process model that incorporates mobility. The approach, which they termed GLEAM in Balcan et al. (2010), has been widely used to study many respiratory pathogens, including COVID-19 and influenza. Balcan et al. (2009) specifically used a branching process approximation within their metapopulation framework to derive invasion thresholds — the theoretical relationship between mobility network structure and the probability of a pathogen successfully establishing in a new subpopulation. The authors should carefully discuss how their work extends beyond what has been done by GLEAM (and similar models, see Hunter et al. 2017 for a decent review).

Fourth, while I agree more work is needed, it’s been established for over a decade that real-time mobility data are often essential for capturing epidemic dynamics. Early papers to discuss include Wesolowki et al. (2012) and (2015). A more recent position piece is Buckee et al. (2020). Kraemer et al. (2020) similarly used Baidu data to examine how SARS-CoV-2 spread from Wuhan and the effects of interventions. It would be important to discuss how the results from Kraemer et al. (2020) on pre-Alpha SARS-CoV-2 qualitatively compare to the authors’ results on Delta and Omicron. Lastly, Perofsky et al. (2024) recently published an analysis of many different respiratory pathogens in Seattle and explored the shifting role of mobility in explaining their dynamics. The key take-home from Perofsky et al. (2024) is that the type of mobility that matters varies a lot and changes over time. At a minimum, the authors should compare/contrast their results with Perofsky et al. (2024). Overall, the authors should better ground their work in prior publications on real-time mobility data and epidemic spreading.

References cited

Balcan, D., Colizza, V., Gonçalves, B., Hu, H., Ramasco, J. J., & Vespignani, A. (2009). Multiscale mobility networks and the spatial spreading of infectious diseases. Proceedings of the national academy of sciences, 106(51), 21484-21489.

Balcan, D., Gonçalves, B., Hu, H., Ramasco, J. J., Colizza, V., & Vespignani, A. (2010). Modeling the spatial spread of infectious diseases: The GLobal Epidemic and Mobility computational model. Journal of computational science, 1(3), 132-145.

Buckee, C. O., Balsari, S., Chan, J., Crosas, M., Dominici, F., Gasser, U., ... & Schroeder, A. (2020). Aggregated mobility data could help fight COVID-19. Science, 368(6487), 145-146.

Colizza, V., & Vespignani, A. (2008). Epidemic modeling in metapopulation systems with heterogeneous coupling pattern: Theory and simulations. Journal of theoretical biology, 251(3), 450-467.

Hunter, E., Mac Namee, B., & Kelleher, J. D. (2017). A taxonomy for agent-based models in human infectious disease epidemiology. Journal of Artificial Societies and Social Simulation, 20(3).

Jamieson-Lane, A., & Blasius, B. (2020). Calculation of epidemic arrival time distributions using branching processes. Physical Review E, 102(4), 042301.

Kraemer, M. U., Yang, C. H., Gutierrez, B., Wu, C. H., Klein, B., Pigott, D. M., ... & Scarpino, S. V. (2020). The effect of human mobility and control measures on the COVID-19 epidemic in China. Science, 368(6490), 493-497.

Mercier, A., Scarpino, S., & Moore, C. (2022). Effective resistance against pandemics: Mobility network sparsification for high-fidelity epidemic simulations. PLOS Computational Biology, 18(11), e1010650.

Perofsky, A. C., Hansen, C. L., Burstein, R., Boyle, S., Prentice, R., Marshall, C., ... & Viboud, C. (2024). Impacts of human mobility on the citywide transmission dynamics of 18 respiratory viruses in pre-and post-COVID-19 pandemic years. Nature communications, 15(1), 4164.

Wesolowski, A., Eagle, N., Tatem, A. J., Smith, D. L., Noor, A. M., Snow, R. W., & Buckee, C. O. (2012). Quantifying the impact of human mobility on malaria. Science, 338(6104), 267-270.

Wesolowski, A., Qureshi, T., Boni, M. F., Sundsøy, P. R., Johansson, M. A., Rasheed, S. B., ... & Buckee, C. O. (2015). Impact of human mobility on the emergence of dengue epidemics in Pakistan. Proceedings of the national academy of sciences, 112(38), 11887-11892.

Any attachments provided with reviews can be seen via the following link: [LINK]

-------------------------------------------------------------

General editorial requests:

*We ask every co-author listed on the manuscript to fill in a contributing author statement, making sure to declare all competing interests. If any of the co-authors have not filled in the statement, we will remind them to do so when the paper is revised. If all statements are not completed in a timely fashion this could hold up the re-review process. If new competing interests are declared later in the revision process, this may also hold up the submission. Should there be a problem getting one of your co-authors to fill in a statement we will be in contact. Please do not add or remove authors without first discussing this with the handling editor. You can see our competing interests policy here: http://journals.plos.org/plosmedicine/s/competing-interests.

*Please upload any figures associated with your paper as individual TIF or EPS files with 300dpi resolution at resubmission; please read our figure guidelines for more information on our requirements: http://journals.plos.org/plosmedicine/s/figures. While revising your submission, we strongly recommend that you use PLOS’s NAAS tool (https://ngplosjournals.pagemajik.ai/artanalysis) to test your figure files. NAAS can convert your figure files to the TIFF file type and meet basic requirements (such as print size, resolution), or provide you with a report on issues that do not meet our requirements and that NAAS cannot fix.

After uploading your figures to PLOS’s NAAS tool - https://ngplosjournals.pagemajik.ai/artanalysis, NAAS will process the files provided and display the results in the "Uploaded Files" section of the page as the processing is complete.

If the uploaded figures meet our requirements (or NAAS is able to fix the files to meet our requirements), the figure will be marked as "fixed" above. If NAAS is unable to fix the files, a red "failed" label will appear above.

When NAAS has confirmed that the figure files meet our requirements, please download the file via the download option, and include these NAAS processed figure files when submitting your revised manuscript.

*Please ensure that the paper adheres to the PLOS Data Availability Policy (see http://journals.plos.org/plosmedicine/s/data-availability), which requires that all data underlying the study’s findings be provided in a repository or as Supporting Information. For data residing with a third party, authors are required to provide instructions with contact information (web or email address) for obtaining the data. Please note that a study author cannot be the contact person for the data. PLOS journals do not allow statements supported by "data not shown" or "unpublished results." For such statements, authors must provide supporting data or cite public sources that include it.

*We expect all researchers with submissions to PLOS in which author-generated code underpins the findings in the manuscript to make all author-generated code available without restrictions upon publication of the work. In cases where code is central to the manuscript, we may require the code to be made available as a condition of publication. Authors are responsible for ensuring that the code is reusable and well documented. Please make any custom code available, either as part of your data deposition or as a supplementary file. Please add a sentence to your data availability statement regarding any code used in the study, e.g. "The code used in the analysis is available from Github [URL] and archived in Zenodo [DOI link]" Please review our guidelines at https://journals.plos.org/plosmedicine/s/materials-software-and-code-sharing and ensure that your code is shared in a way that follows best practice and facilitates reproducibility and reuse. Because Github depositions can be readily changed or deleted, we encourage you to make a permanent DOI’d copy (e.g. in Zenodo) and provide the URL.

FORMATTING - GENERAL

*Abstract: Please structure your abstract using the PLOS Medicine headings (Background, Methods and Findings, Conclusions). Please combine the Methods and Findings sections into one section.

*At this stage, we ask that you include a short, non-technical Author Summary of your research to make findings accessible to a wide audience that includes both scientists and non-scientists. The Author Summary should immediately follow the Abstract in your revised manuscript. This text is subject to editorial change and should be distinct from the scientific abstract. Ideally each sub-heading should contain 2-3 single sentence, concise bullet points containing the most salient points from your study. In the final bullet point of ‘What Do These Findings Mean?’, please include the main limitations of the study in non-technical language. Please see our author guidelines for more information: https://journals.plos.org/plosmedicine/s/revising-your-manuscript#loc-author-summary.

*Please express the main results with 95% CIs as well as p values. When reporting p values please report as p<0.001 and where higher as the exact p value p=0.002, for example. Throughout, suggest reporting statistical information as follows to improve clarity for the reader "22% (95% CI [13%,28%]; p</=)". Please be sure to define all numerical values at first use.

*Please include page numbers and line numbers in the manuscript file. Use continuous line numbers (do not restart the numbering on each page).

*Please cite the reference numbers in square brackets. Citations should precede punctuation.

FIGURES AND TABLES

*Please provide titles and legends for all figures and tables (including those in Supporting Information files).

*Please define all abbreviations used in each figure/table (including those in Supporting Information files).

*Please consider avoiding the use of red and green in order to make your figure more accessible to those with color blindness.

SUPPLEMENTARY MATERIAL

*Please note that supplementary material will be posted as supplied by the authors. Therefore, please amend it according to the relevant comments outlined here.

*Please cite your Supporting Information as outlined here: https://journals.plos.org/plosmedicine/s/supporting-information

REFERENCES

*PLOS uses the numbered citation (citation-sequence) method and first six authors, et al.

*Please ensure that journal name abbreviations match those found in the National Center for Biotechnology Information (NCBI) databases (http://www.ncbi.nlm.nih.gov/nlmcatalog/journals), and are appropriately formatted and capitalised.

*Where website addresses are cited, please include the complete URL and specify the date of access (e.g. [accessed: 12/06/2023]).

*Please also see https://journals.plos.org/plosmedicine/s/submission-guidelines#loc-references for further details on reference formatting.

MODELLING STUDIES

The following list is derived from Geoffrey P Garnett, Simon Cousens, Timothy B Hallett, Richard Steketee, Neff Walker. Mathematical models in the evaluation of health programmes. (2011) Lancet DOI:10.1016/S0140-6736(10)61505-X:

*If pertinent, please provide a diagram that shows the model structure, including how the natural history of the disease is represented, the process and determinants of disease acquisition, and how the putative intervention could affect the system.

*Please provide a complete list of model parameters, including clear and precise descriptions of the meaning of each parameter, together with the values or ranges for each, with justification or the primary source cited and important caveats about the use of these values noted.

*Please provide a clear statement about how the model was fitted to the data, including goodness-of-fit measure, the numerical algorithm used, which parameter varied, constraints imposed on parameter values, and starting conditions.

*For uncertainty analyses, please state the sources of uncertainties quantified and not quantified [can include parameter, data, and model structure].

*Please provide sensitivity analyses to identify which parameter values are most important in the model. Uncertainty estimates seek to derive a range of credible results on the basis of an exploration of the range of reasonable parameter values. The choice of method should be presented and justified.

*Please discuss the scientific rationale for the choice of model structure and identify points where this choice could influence conclusions drawn. Please also describe the strength of the scientific basis underlying the key model assumptions.

*For studies that develop a prediction model or evaluate its performance, please ensure that the study is reported according to the TRIPOD statement (https://www.equator-network.org/reporting-guidelines/tripod-statement) and include the completed checklist as Supporting Information. Please add the following statement, or similar, to the Methods: "This study is reported as per the Transparent Reporting of a Multivariable Prediction Model for Individual Prognosis Or Diagnosis (TRIPOD) statement (S1 Checklist)." For studies using machine learning, please use the TRIPOD-AI checklist. When completing the checklist, please use section and paragraph numbers, rather than page numbers.

# Author comment: Author response to Decision Letter 2

**DOI:** 10.1371/journal.pmed.1005172.r003

29 Apr 2026

Attachment Submitted filename: Response to Reviewers R2_submission.pdf

# Peer review: Decision Letter 2

**DOI:** 10.1371/journal.pmed.1005172.r004

**Authors:** Andreia Cunha

4 Jun 2026

Dear Dr. Yao,

Sincere apologies for the delay in getting back to you with a decision. Thank you very much for re-submitting your manuscript "Assessing spatial transmission risk of respiratory infectious diseases across urban tiers in China: a modelling study" (PMEDICINE-D-25-04489R2) for review by PLOS Medicine.

I have discussed the paper with my colleagues and the academic editor and it was also seen again by the original reviewers. I am pleased to say that provided the remaining editorial and production issues are dealt with we are planning to accept the paper for publication in the journal.

The remaining issues that need to be addressed are listed at the end of this email. Any accompanying reviewer attachments can be seen via the link below. Please take these into account before resubmitting your manuscript:

[LINK]

***Please note while forming your response, if your article is accepted, you may have the opportunity to make the peer review history publicly available. The record will include editor decision letters (with reviews) and your responses to reviewer comments. If eligible, we will contact you to opt in or out.***

In revising the manuscript for further consideration here, please ensure you address the specific points made by each reviewer and the editors. In your rebuttal letter you should indicate your response to the reviewers’ and editors’ comments and the changes you have made in the manuscript. Please submit a clean version of the paper as the main article file. A version with changes marked must also be uploaded as a marked up manuscript file.

Please also check the guidelines for revised papers at http://journals.plos.org/plosmedicine/s/revising-your-manuscript for any that apply to your paper. If you haven’t already, we ask that you provide a short, non-technical Author Summary of your research to make findings accessible to a wide audience that includes both scientists and non-scientists. The Author Summary should immediately follow the Abstract in your revised manuscript. This text is subject to editorial change and should be distinct from the scientific abstract.

In addition to these revisions, you may need to complete some formatting changes, which you will receive in a follow up email. A member of our team will be in touch with a set of requests shortly. If you do not receive a separate email within a few days, please assume that checks have been completed, and no additional changes are required.

We expect to receive your revised manuscript within 1 week. Please email us (plosmedicine@plos.org) if you have any questions or concerns.

We ask every co-author listed on the manuscript to fill in a contributing author statement. If any of the co-authors have not filled in the statement, we will remind them to do so when the paper is revised. If all statements are not completed in a timely fashion this could hold up the re-review process. Should there be a problem getting one of your co-authors to fill in a statement we will be in contact. YOU MUST NOT ADD OR REMOVE AUTHORS UNLESS YOU HAVE ALERTED THE EDITOR HANDLING THE MANUSCRIPT TO THE CHANGE AND THEY SPECIFICALLY HAVE AGREED TO IT.

Please ensure that the paper adheres to the PLOS Data Availability Policy (see http://journals.plos.org/plosmedicine/s/data-availability), which requires that all data underlying the study’s findings be provided in a repository or as Supporting Information. For data residing with a third party, authors are required to provide instructions with contact information for obtaining the data. PLOS journals do not allow statements supported by "data not shown" or "unpublished results." For such statements, authors must provide supporting data or cite public sources that include it.

To enhance the reproducibility of your results, we recommend that you deposit your laboratory protocols in protocols.io, where a protocol can be assigned its own identifier (DOI) such that it can be cited independently in the future. Additionally, PLOS ONE offers an option to publish peer-reviewed clinical study protocols. Read more information on sharing protocols at https://plos.org/protocols?utm_medium=editorial-email&utm_source=authorletters&utm_campaign=protocols

Please review your reference list to ensure that it is complete and correct. If you have cited papers that have been retracted, please include the rationale for doing so in the manuscript text, or remove these references and replace them with relevant current references. Any changes to the reference list should be mentioned in the rebuttal letter that accompanies your revised manuscript.

Please note, when your manuscript is accepted, an uncorrected proof of your manuscript will be published online ahead of the final version, unless you’ve already opted out via the online submission form. If, for any reason, you do not want an earlier version of your manuscript published online or are unsure if you have already indicated as such, please let the journal staff know immediately at plosmedicine@plos.org.

If you have any questions in the meantime, please contact me or the journal staff on plosmedicine@plos.org.

We look forward to receiving the revised manuscript by Jun 11 2026 11:59PM.

Sincerely,

Andreia Cunha, PhD

Senior Editor

PLOS Medicine

plosmedicine.org

------------------------------------------------------------

Requests from Editors:

GENERAL EDITORIAL REQUESTS

* Please confirm that your title complies with PLOS Medicine’s style. Your title must be nondeclarative and not a question. It should begin with main concept if possible. "Effect of" should be used only if causality can be inferred, i.e., for an RCT. Please place the study design ("A randomized controlled trial," "A retrospective study," "A modelling study," etc.) in the subtitle (ie, after a colon).

* Please confirm that your abstract complies with our requirements, including format (three sections: Background, Methods and Findings, and Conclusions) and providing all the information relevant to this study type https://journals.plos.org/plosmedicine/s/submission-guidelines#loc-abstract

* In the last sentence of the Abstract Methods and Findings section, please describe the main limitation(s) of the study’s methodology.

* In the abstract, please include the important dependent variables that are adjusted for in the analyses.

* Please explain the meaning of 'tier' in the Abstract, and also please tone down the following sentence: Mobility

informed, tier-specific risk assessment can strengthen early warning and enable more adaptive public health responses.

* Please ensure that the Introduction ends with a clear description of the study question or hypothesis.

* Please ensure that all abbreviations are defined at first use throughout the text.

* Please confirm that all numbers presented in the abstract are present and identical to numbers presented in the main manuscript text.

GENERAL

* Please disclose any use of AI tools and technologies to the study or article content at the end of the Methods. Please specify the name(s) and version(s) of any tool(s) used and a description of their use, including how the AI-assisted or generated content was evaluated by authors. Please also include a statement specifying who retains responsibility for the final manuscript. Please see our guidelines for more information: https://journals.plos.org/plosmedicine/s/ethical-publishing-practice#loc-artificial-intelligence-tools-and-technologies.

* Statistical reporting: Please revise throughout the manuscript, including tables and figures.

- Please report statistical information as follows to improve clarity for the reader ""22% (95% CI [13,28]; p</=)"".

- Please separate upper and lower bounds with commas instead of hyphens as the latter can be confused with reporting of negative values.

- Please repeat statistical definitions (HR, CI etc.) for each set of parentheses."

FUNDING STATEMENT

* The funding statement should include: specific grant numbers, initials of authors who received each award, URLs to sponsors’ websites. Also, please state whether any sponsors or funders (other than the named authors) played any role in study design, data collection and analysis, the decision to publish, or preparation of the manuscript. If they had no role in the research, include this sentence: “The funders had no role in study design, data collection and analysis, decision to publish, or preparation of the manuscript.”

* It appears that one or more study authors may be affiliated with one or more of the agencies that funded the study. Thus, the statement “The funders had no role in study design, data collection and analysis, decision to publish, or preparation of the manuscript” would not apply. If applicable, please revise the Financial Disclosure accordingly, as in "[Author name] is [author’s role] at [funding agency]. The funders had no other role in study design…..”

COMPETING INTERESTS STATEMENT

* All authors must declare their relevant competing interests per the PLOS policy, which can be seen here: https://journals.plos.org/plosmedicine/s/competing-interests For authors with ties to industry, please indicate whether any of the interests has a financial stake in the results of the current study.

DATA AVAILABILITY

* For studies in which a novel model is central to the manuscript’s findings, as is the case here, authors are responsible for providing the source code needed to replicate the study’s findings in a repository (such as GitHub, SourceForge or Bitbucket) or a cloud computing service (such as Code Ocean). Please include a citable digital object identifier (DOI) or other persistent identifier of the code (such as through linkage with Zenodo). Protection of authors’ intellectual property will not be cause for exception. Please explain in the manuscript’s Data Availability Statement how readers can access the shared code.

* Please include the statement on code availability in the data availability statement. Please see guidance here:https://journals.plos.org/plosmedicine/s/code-sharing-guidance#loc-data-availability-statement-examples

FIGURES

* Please provide titles and legends for all figures and tables (including those in Supporting Information files). Please define all acronyms used in each figure or table in its corresponding legend.

* When a p value is given, please specify the statistical test used to determine it in the legend.

* Please consider avoiding the use of red and green in order to make your figure more accessible

* Please confirm that the appropriate usage rights apply to the use of this map. Please see our guidelines for map images: https://journals.plos.org/plosmedicine/s/figures#loc-maps

* Please provide the unadjusted comparisons as well as the adjusted comparisons in all relevant Tables

* Please specify the variables controlled for in all relevant Tables

ACKNOWLEDGMENTS

* Please include an Acknowledgments section in your manuscript.

MODELING STUDIES

For more details on the requests below please see Geoffrey P Garnett, Simon Cousens, Timothy B Hallett, Richard Steketee, Neff Walker. Mathematical models in the evaluation of health programmes. (2011) Lancet DOI:10.1016/S0140-6736(10)61505-X.

* Please provide a diagram that shows the model structure, including how the disease natural history is represented, the process and determinants of disease acquisition, and how the putative intervention could affect the system.

* Please provide a complete list of model parameters, including clear and precise descriptions of the meaning of each parameter, together with the values or ranges for each, with justification or the primary source cited, and important caveats about the use of these values noted.

* Please provide a clear statement about how the model was fitted to the data including where relevant goodness-of-fit measure, the numerical algorithm used, which parameter varied, constraints imposed on parameter values, and starting conditions.

* For uncertainty analyses, please state the sources of uncertainties quantified and not quantified this can include parameter, data, and model structure.

* Please provide sensitivity analyses to identify which parameter values are most important in the model. Uncertainty estimates seek to derive a range of credible results on the basis of an exploration of the range of reasonable parameter values. The choice of method should be presented and justified.

* Please discuss the scientific rationale for this choice of model structure and identify points where this choice could influence conclusions drawn. Please also describe the strength of the scientific basis underlying the key model assumptions.

Reviewers' comments

Reviewer #2: All the comments are properly addressed in this version of the manuscript.

Reviewer #3: The authors have addressed my major methodological and statistical concerns comprehensively. The revised manuscript now includes additional validation metrics (MAE/RMSE), expanded uncertainty analyses using joint probabilistic sampling, permutation-based robustness checks, clearer justification of modelling assumptions, and improved transparency in the main Methods and Supplementary Material. These revisions substantially strengthen the rigour and interpretability of the study.

While some limitations remain inherent to the modelling framework, particularly regarding validation against first arrival times rather than full epidemic dynamics, the authors now acknowledge these limitations appropriately and provide reasonable justification for their analytical choices.

The manuscript now reflects a robust and valuable contribution to subject, I am happy to recommend this work for publication and look forward to seeing its positive impact in the field.

Reviewer #4: The authors have done a thorough job with the revision.

Any attachments provided with reviews can be seen via the following link:

[LINK]

# Author comment: Author response to Decision Letter 3

**DOI:** 10.1371/journal.pmed.1005172.r005

18 Jun 2026

Attachment Submitted filename: Response to Editors R3_resubmission.docx

# Editor report: Decision Letter 3

**DOI:** 10.1371/journal.pmed.1005172.r006

**Authors:** Heather Van Epps

24 Jun 2026

Dear Dr Yao,

On behalf of my colleagues and the Academic Editor, Dr Simon Cauchemez, I am pleased to inform you that we have agreed to publish your manuscript "Assessing spatial transmission risk of respiratory infectious diseases across cities of different socioeconomic tiers in China: a modelling study" (PMEDICINE-D-25-04489R3) in PLOS Medicine.

Before your manuscript can be formally accepted you will need to complete some formatting changes, which you will receive in a follow up email. Please be aware that it may take several days for you to receive this email; during this time no action is required by you. Once you have received these formatting requests, please note that your manuscript will not be scheduled for publication until you have made the required changes.

In the meantime, please log into Editorial Manager at http://www.editorialmanager.com/pmedicine/, click the "Update My Information" link at the top of the page, and update your user information to ensure an efficient production process.

PRESS

We frequently collaborate with press offices. If your institution or institutions have a press office, please notify them about your upcoming paper at this point, to enable them to help maximise its impact. If the press office is planning to promote your findings, we would be grateful if they could coordinate with medicinepress@plos.org. If you have not yet opted out of the early version process, we ask that you notify us immediately of any press plans so that we may do so on your behalf.

We also ask that you take this opportunity to read our Embargo Policy regarding the discussion, promotion and media coverage of work that is yet to be published by PLOS. As your manuscript is not yet published, it is bound by the conditions of our Embargo Policy. Please be aware that this policy is in place both to ensure that any press coverage of your article is fully substantiated and to provide a direct link between such coverage and the published work. For full details of our Embargo Policy, please visit http://www.plos.org/about/media-inquiries/embargo-policy/.

To enhance the reproducibility of your results, we recommend that you deposit your laboratory protocols in protocols.io, where a protocol can be assigned its own identifier (DOI) such that it can be cited independently in the future. Additionally, PLOS ONE offers an option to publish peer-reviewed clinical study protocols. Read more information on sharing protocols at https://plos.org/protocols?utm_medium=editorial-email&utm_source=authorletters&utm_campaign=protocols

Thank you again for submitting to PLOS Medicine. We look forward to publishing your paper.

Sincerely,

Andreia Cunha, PhD

Senior Editor

PLOS Medicine
